# Chemoselective dehydrogenative esterification of aldehydes and alcohols with a dimeric rhodium(ii) catalyst†

**DOI:** 10.1039/c6sc00145a

**Published:** 2016-03-29

**Authors:** Junjie Cheng, Meijuan Zhu, Chao Wang, Junjun Li, Xue Jiang, Yawen Wei, Weijun Tang, Dong Xue, Jianliang Xiao

**Affiliations:** a Key Laboratory of Applied Surface and Colloid Chemistry, Ministry of Education, School of Chemistry and Chemical Engineering, Shaanxi Normal University Xi'an 710062 China c.wang@snnu.edu.cn; b Department of Chemistry, University of Liverpool Liverpool L69 7ZD UK j.xiao@liverpool.ac.uk

## Abstract

Dehydrogenative cross-coupling of aldehydes with alcohols as well as dehydrogentive cross-coupling of primary alcohols to produce esters have been developed using a Rh-terpyridine catalyst. The catalyst demonstrates broad substrate scope and good functional group tolerance, affording esters highly selectively. The high chemoselectivity of the catalyst stems from its preference for dehydrogenation of benzylic alcohols over aliphatic ones. Preliminary mechanistic studies suggest that the active catalyst is a dimeric Rh(ii) species, operating *via* a mechanism involving metal–base–metal cooperativity.

## Abstract 2

A dimeric rhodium(ii) complex catalyses the chemoselective dehydrogenative esterification of aldehydes and alcohols.

## Graphical abstract

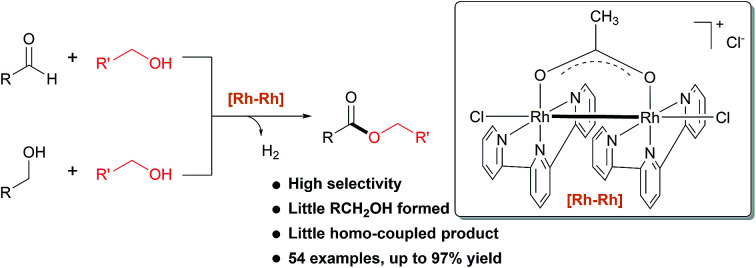

## Introduction

Esters are among the most important and abundant functional groups in chemistry, widely found in food, pharmaceutical, fragrance, flavour, and fine and bulk chemical industries.^1^ There are a number of traditional methods, *e.g.* reaction with carboxylic acid derivatives,^1^ carbonylation^2^ and the Tishchenko reaction,^3^ which could be used for the preparation of ester compounds. The coupling of aldehydes with alcohols^4^ or coupling of alcohols themselves^5^ in the presence of stoichiometric oxidants can also produce esters. An alternative green approach is the dehydrogenative coupling^6^ of alcohols or of aldehydes with alcohols with the release of H_2_.

**Scheme 1:**
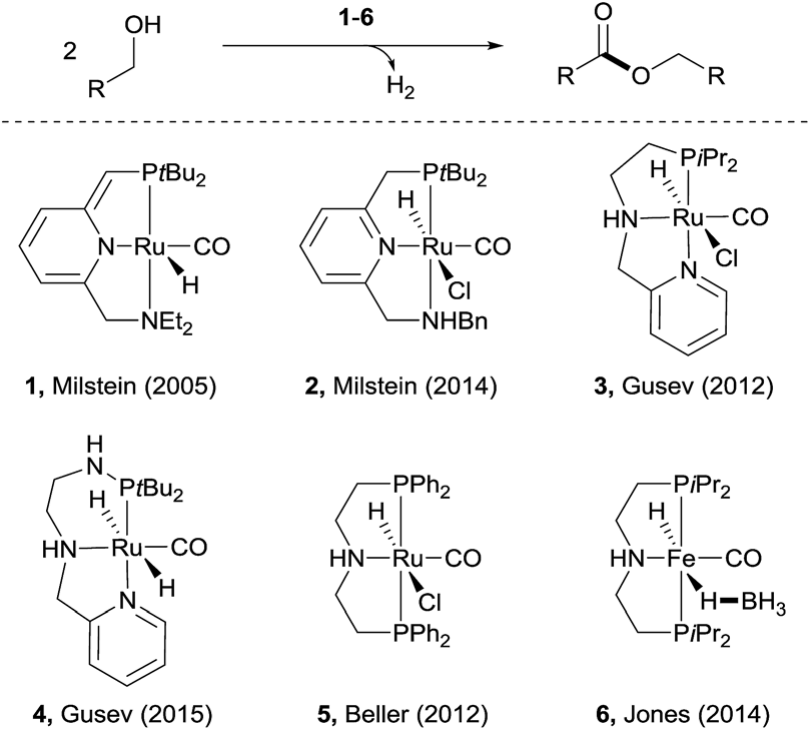
Recent examples of catalysts for acceptorless dehydrogenative coupling of alcohols to form esters.

Examples of acceptorless dehydrogenative *homo-coupling* of alcohols have been reported.^7^ Early in 1981, Murahashi and co-workers reported that the simple RuH_2_(PPh_3_)_4_ could catalyse the formation of esters and lactones from alcohols and diols.^7*a*^ Later in 1985, Shvo and co-workers found that Ru(η^4^-tetraphenylcyclopentadienone)(CO)_3_ could act as catalyst for dehydrogenative homo-coupling of primary alcohols to esters.^7*b*^ The introduction of metal–ligand cooperative catalysts for dehydrogenation reactions by Milstein and co-workers has spurred the development of this area.^8^ Milstein and co-workers reported a metal–ligand bifunctional ruthenium catalysts 1,^7*d*^ which functions through aromatisation/dearomatisation of the PNN ligand, as well as a highly active catalyst 2 (ref. 7*n*) with dual modes of metal–ligand cooperation, for acceptorless dehydrogenative homo-coupling of alcohols (Scheme 1). Gusev and co-workers designed complexes 3 (ref. 7*i* and *j*) and 4 (ref. 7*p*) bearing PNN ligands for ester formation from alcohols with release of H_2_. Beller and co-workers found that the Ru-PNP complex 5 (ref. 7*h*) was highly active for dehydrogenative coupling of ethanol to produce ethyl acetate. The iron complex 6 (ref. 7*o*) with a PNP ligand could also catalyse dehydrogenative coupling of alcohols, as demonstrated by Jones and co-workers.

**Scheme 2:**
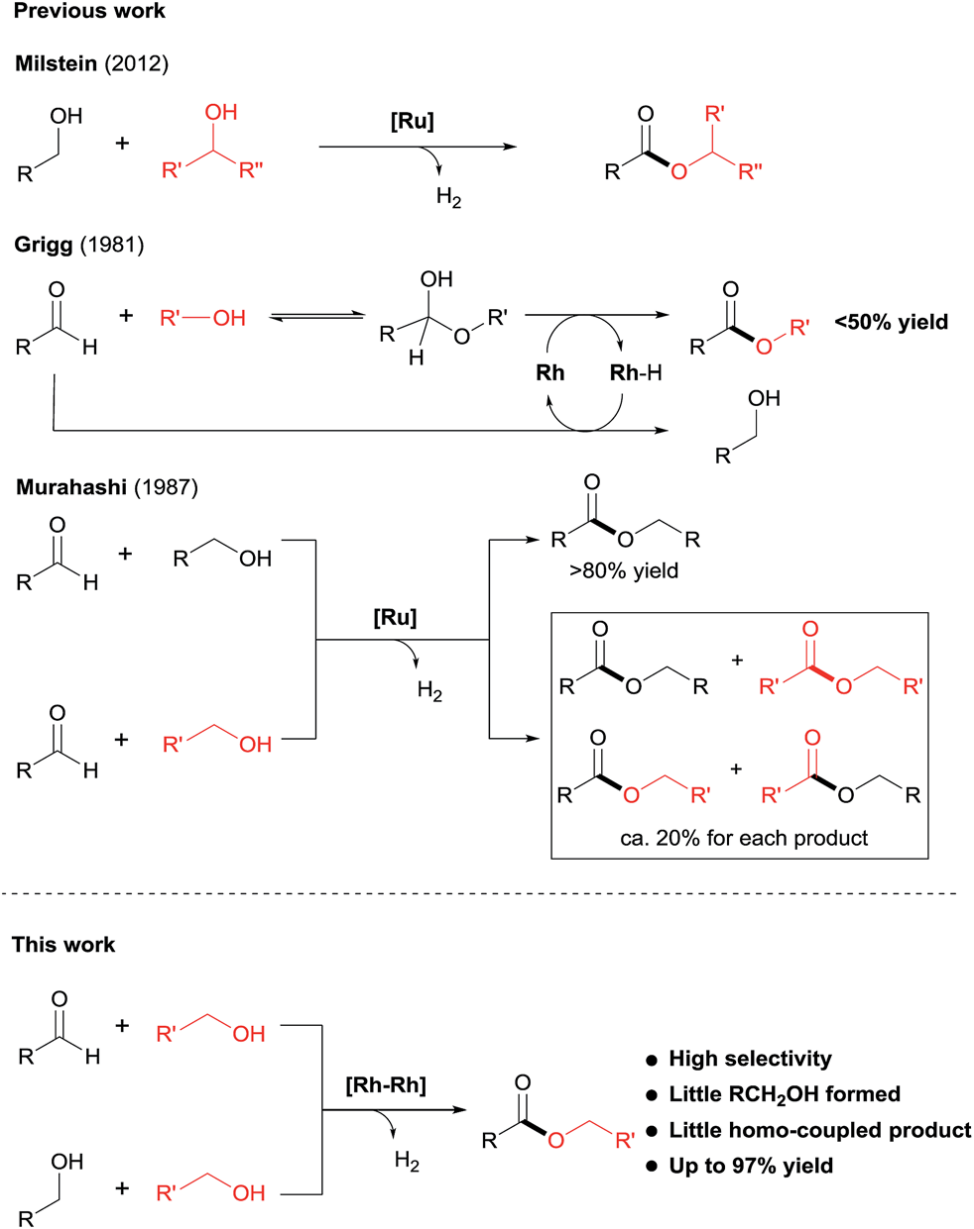
Cross-coupling of alcohols and of aldehydes with alcohols to produce esters.

Despite the progress made in catalyst development, the substrate scope for dehydrogenative coupling of alcohols remains limited, *with most of the catalysts only allowing for homo-coupling or intramolecular coupling of alcohols*. In particular, the acceptorless dehydrogenative *cross-coupling* of alcohols is still challenging. Milstein and co-workers reported an example of dehydrogenative cross-coupling of primary alcohols with secondary alcohols (Scheme 2).^7*k*^ To the best of our knowledge, *the cross-coupling of two different primary alcohols to form esters with evolution of H*_*2*_*has not been reported*.

Likewise, the dehydrogenative *cross-coupling of aldehydes* with alcohols is rare. A critical issue facing such cross-coupling reactions is that the metal hydride intermediate, expected to form during the dehydrogenation step,^6,9^ can easily reduce the aldehydes, instead of undergoing protonation to form H_2_. Indeed, Grigg reported that when aldehydes were reacted with boiling alcohols under the catalysis of RhH(CO)(PPh_3_)_3_, a mixture of esters and alcohols was obtained, with the ester yields generally <50% (Scheme 2).^10^ Later in 1987, Murahashi reported another example of cross-coupling of aldehydes with alcohols. The reaction was selective when a RCHO was coupled with the corresponding RCH_2_OH, but was non-selective with R′CH_2_OH, producing a mixture of homo- and cross-coupled esters in *ca.* 20% yield for each product (Scheme 2).^7*c*^ In both of the examples of cross coupling, reduction of the aldehydes occurred considerably. Given the widespread of aldehydes in natural and synthetic compounds, such coupling could provide an easy way to converting the compound into an ester.

Herein, we disclose a novel catalytic system that allows for highly chemoselective dehydrogenative *cross-coupling of aldehydes* with alcohols to afford esters. The dimeric Rh–tpy (tpy = 2,2′:6′,2′′-terpyridine) catalyst^11^ unearthed also enables the dehydrogenative *cross-coupling of primary alcohols*^7*k*^ to form esters (Scheme 2).^12^

## Results and discussion

### 1. Cross coupling of aldehydes with alcohols

We set out to examine a model reaction with 4-methylbenzaldehyde and MeOH as substrates (Table 1). As expected, in the absence of a catalyst, reacting 4-methylbenzaldehyde with MeOH converted the aldehyde only into a dimethyl acetal in MeOH at 90 °C. Addition of a potential catalyst, [Cp*RhCl_2_]_2_, brought about no ester formation either. Interestingly, in the presence of both [Cp*RhCl_2_]_2_ (1 mol%) and a base NaOAc (5 equivalents), the desired ester 7a was formed in 28% yield alongside an equal amount of the undesired alcohol 8 (Table 1, entry 1). Ligands were next introduced (ligand/Rh = 1.2) and found to affect both the catalytic activity and selectivity. Thus, bidentate nitrogen ligands inhibited the reaction, and when phosphines were used, the selectivity for 8 increased slightly (Table 1, entries 2–5). Surprisingly somehow, when tpy was added,^13^ the ester was formed as the major product, albeit only in 11% yield (Table 1, entry 6). However, deviating from the approximately 1 : 1 tpy/Rh ratio resulted in the loss of either catalytic selectivity or activity (Table 1, entries 7 and 8 *vs.* 6). The choice of base is also critical for the selectivity. Among the bases examined, NaOAc appeared most effective for the selective formation of 7a (Table 1, entries 9–11 *vs.* 6), with 1 equivalent being sufficient.

**Table 1:** Optimisation of conditions for a model coupling^a^

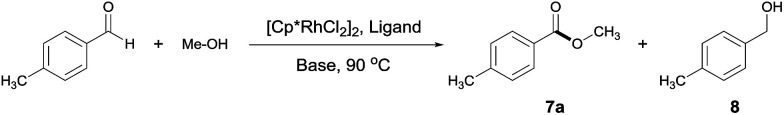				
Entry	Ligand^b^	Base	Yield of 7a^c^ (%)	Yield of 8^c^ (%)
1	—	NaOAc	28	28
2	bipy	NaOAc	<5	<5
3	phen	NaOAc	<5	<5
4	PPh_3_	NaOAc	15	19
5	dppp	NaOAc	28	32
6	tpy	NaOAc	11	1
7^d^	tpy	NaOAc	18	16
8^e^	tpy	NaOAc	<5	<5
9	tpy	NaOH	<5	36
10	tpy	NaHCO_3_	50	26
11	tpy	Et_3_N	17	9
12^f^	tpy	NaOAc	60	—
13^f^^,^^g^	tpy	NaOAc	85	<1
14^f^^,^^g^^,^^h^	tpy	NaOAc	94	<1

a Reaction conditions: aldehyde (0.5 mmol), metal complex (0.005 mmol), ligand (0.012 mmol, except for 0.024 mmol PPh_3_), base (2.5 mmol), MeOH (2 mL), 90 °C in a sealed tube for 6 h.

b bipy = bipyridine; phen = phenanthroline; dppp = 1,3-bis(diphenylphosphino)propane.

c Yields were determined by ^1^H NMR with 1,3,5-trimethoxybenzene as internal standard.

d 0.006 mmol of tpy used.

e 0.024 mmol of tpy used.

f Radleys tube connected to an empty balloon and with 0.5 mmol of NaOAc.

g 0.0125 mmol of NaOH was added.

h 12 h.

The reactions above were performed in a sealed tube. We noted that the reaction was inhibited when placed under an oxygen atmosphere. As there was no oxidant added, the selective formation of ester was expected to generate H_2_.^14^

Thus, to facilitate the release of H_2_, we switched the reaction vessel to a Radleys tube connected to an empty balloon. Delightfully, a dramatic increase of yield from 11 to 60% was observed, with almost 100% selectivity toward the ester as confirmed by both ^1^H NMR and GC-MS analysis (Table 1, entry 12 *vs.* 6). And with the addition of 2.5 mol% of NaOH, the ester was obtained in a satisfactory yield of 94% in a prolonged time of 12 h (Table 1, entries 13 and 14).

It is noted that decreasing the amount of MeOH used does not alter the chemoselectivity but slows the reaction. Thus, when the aldehyde/alcohol molar ratio was lowered to 1 : 3, the ester was obtained in *ca.* 50% yield with the rest of the aldehyde unreacted under the optimized conditions (ESI, Fig. S1†). However, carrying out the reaction in other solvents, such as toluene, DMSO, acetonitrile or dioxane (2 mL plus 0.5 mL MeOH), resulted in no or little product.

**Scheme 3:**
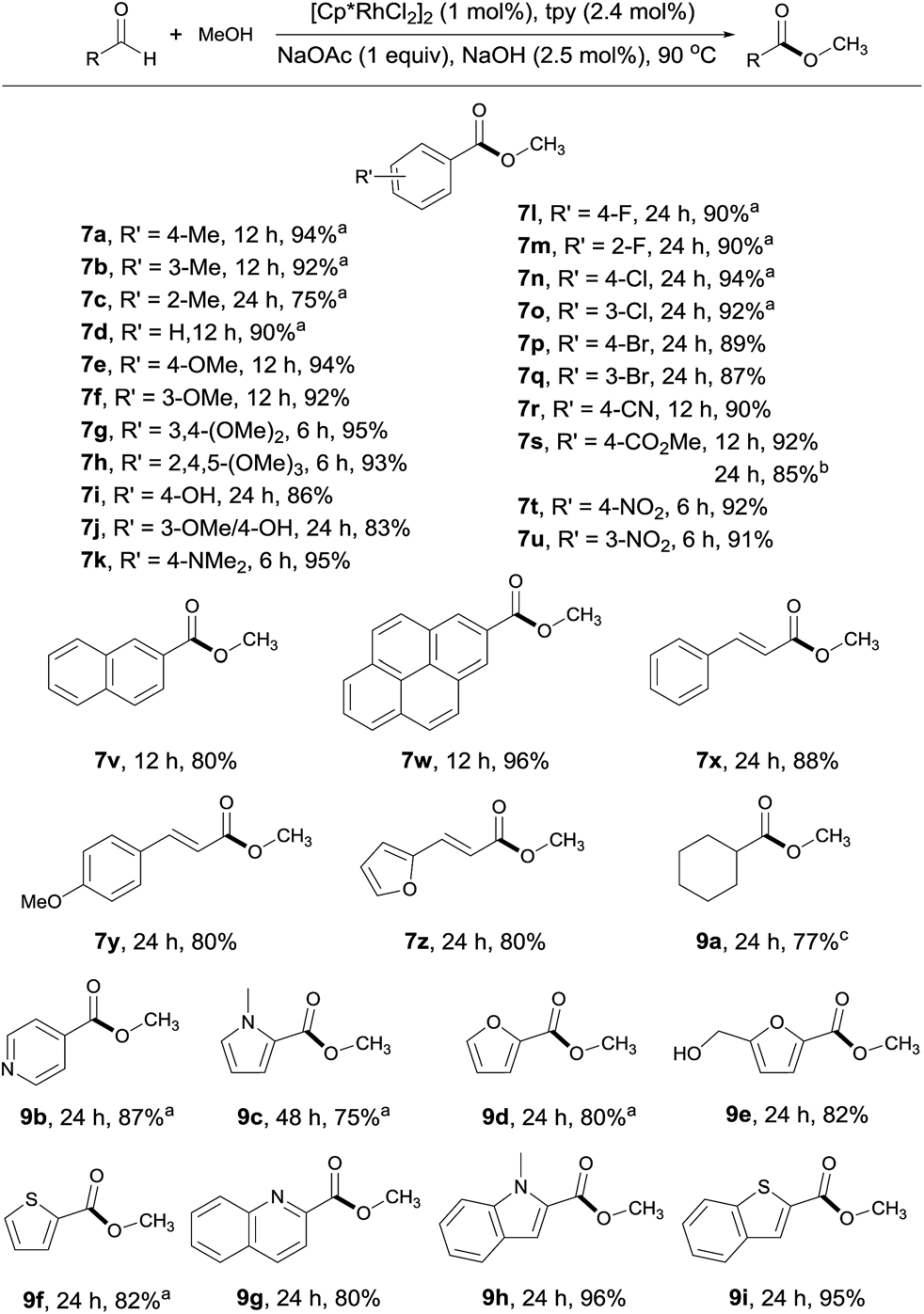
Coupling of MeOH with various aldehydes. Isolated yields are given, 2 mL MeOH; see ESI for details.†^*a*^Yields determined by ^1^H NMR with an internal standard. ^*b*^Terephthalaldehyde used as substrate. ^*c*^Yield determined by GC.

With the optimal conditions in hand, the generality of this catalytic system was examined, first by reacting MeOH with different aldehydes. Both electron rich and deficient aromatic aldehydes reacted well with MeOH to give the corresponding esters with good to excellent yields in 6–24 h (Scheme 3, 7a–7w). Of particular note is that substrates bearing various functional groups, such as –OH, –NMe_2_, –CN, –CO_2_Me and C=C double bonds, all reacted well, with the functional groups being intact (7i, 7k, 7r, 7s, 7x–7z). This is difficult to achieve with traditional esterification methods, as most of these groups are prone to decomposition under or incompatible with the reaction conditions. When terephthalaldehyde was subjected to the coupling, both carbonyl groups were converted to esters (7s). Substrates with multiple aromatic rings are also viable (7v, 7w), so are aliphatic aldehydes as demonstrated by 9a. The substrate scope could also be extended to heterocyclic aldehydes (9b–9i). However, longer reaction time was required for these substrates, probably due to competing coordination of the heteroatom to the rhodium. Worth noting is that 5-hydroxymethylfurfural, a platform molecule derived from biomass,^15^ could be selectively transformed into its ester in 82% yield, with its hydroxyl group intact under the conditions employed (9e).

**Scheme 4:**
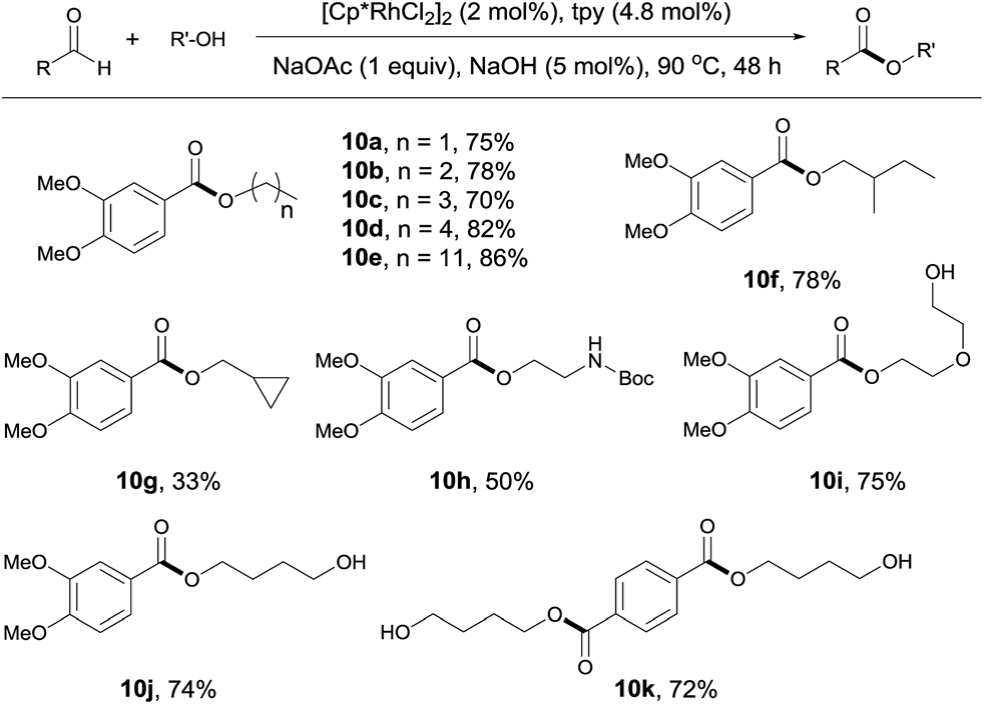
Coupling of aldehydes with different alcohols. Isolated yields are given, 1 mL alcohol; see ESI for details.†

The reaction of aldehydes with other alcohols was next examined (Scheme 4). On switching from methanol to these alcohols, the reaction became slower probably due to the increased steric hindrance hampering β hydrogen elimination or decreased amount of alcohol used (1 mL). Thus, a higher catalyst loading (2 mol%) and a longer time (48 h) were necessary to obtain acceptable yields. Under these conditions, aliphatic alcohols with different chain length all reacted well with 3,4-dimethoxybenzaldehyde (Scheme 4, 10a–10e). Branched aliphatic alcohols could also be used, although a low yield was obtained for cyclopropylmethanol (10f, 10g). Protected amino group survived in the reaction (10h). Interestingly, diols entered the coupling with only one hydroxyl group participating in the esterification. This allows for further functionalization of the free hydroxyl group (10i, 10j). In contrast, the reaction of terephthalaldehyde with 1,4-butanediol resulted in the formation of a diester, with no polymer product observed (10k). Apparently, this contrast results from the use of excess alcohols.

In the reactions above, the catalyst was *in situ* generated from the reaction of [Cp*RhCl_2_]_2_ with tpy, which could lead to a coordinatively saturated and therefore catalytically inactive [Cp*Rh(tpy)]^2+^ complex (*vide infra*). In an attempt to gain insight into what the real active catalyst was, we reacted [Cp*RhCl_2_]_2_ with tpy in MeOH, isolating instead a known compound [RhCl_3_(tpy)] 11 in low yield, in which the Cp* ligand has been displaced (*vide infra*).^16^ This complex showed a higher catalytic activity than the *in situ* formed catalyst in the coupling of 3,4-dimethoxybenzaldehyde with MeOH (ESI, Table S2†), suggesting it might be a pre-catalyst. Complex 11 could be readily prepared from RhCl_3_ and tpy in high yield^16^ and so was subsequently explored for alcohol coupling, while the mechanistic implication of 11 was being explored (*vide infra*).

### 2. Cross coupling of alcohols

Although dehydrogenative homo-coupling of alcohols^7^ to form esters has been reported, the *cross-coupling* of alcohols remains largely challenging.^6*f*,7*k*^ To our delight, in the presence of 1 mol% of 11 and NaOAc, the coupling of 4-methylbenzyl alcohol with MeOH afforded 7a in 22% yield in 6 h at 90 °C (ESI, Table S1†). Further studies revealed NaHCO_3_ (0.5 equivalent) to be a better choice of base. Under these conditions, 4-methylbenzyl alcohol reacted with MeOH to afford 7a in 96% NMR yield in 12 h.

**Scheme 5:**
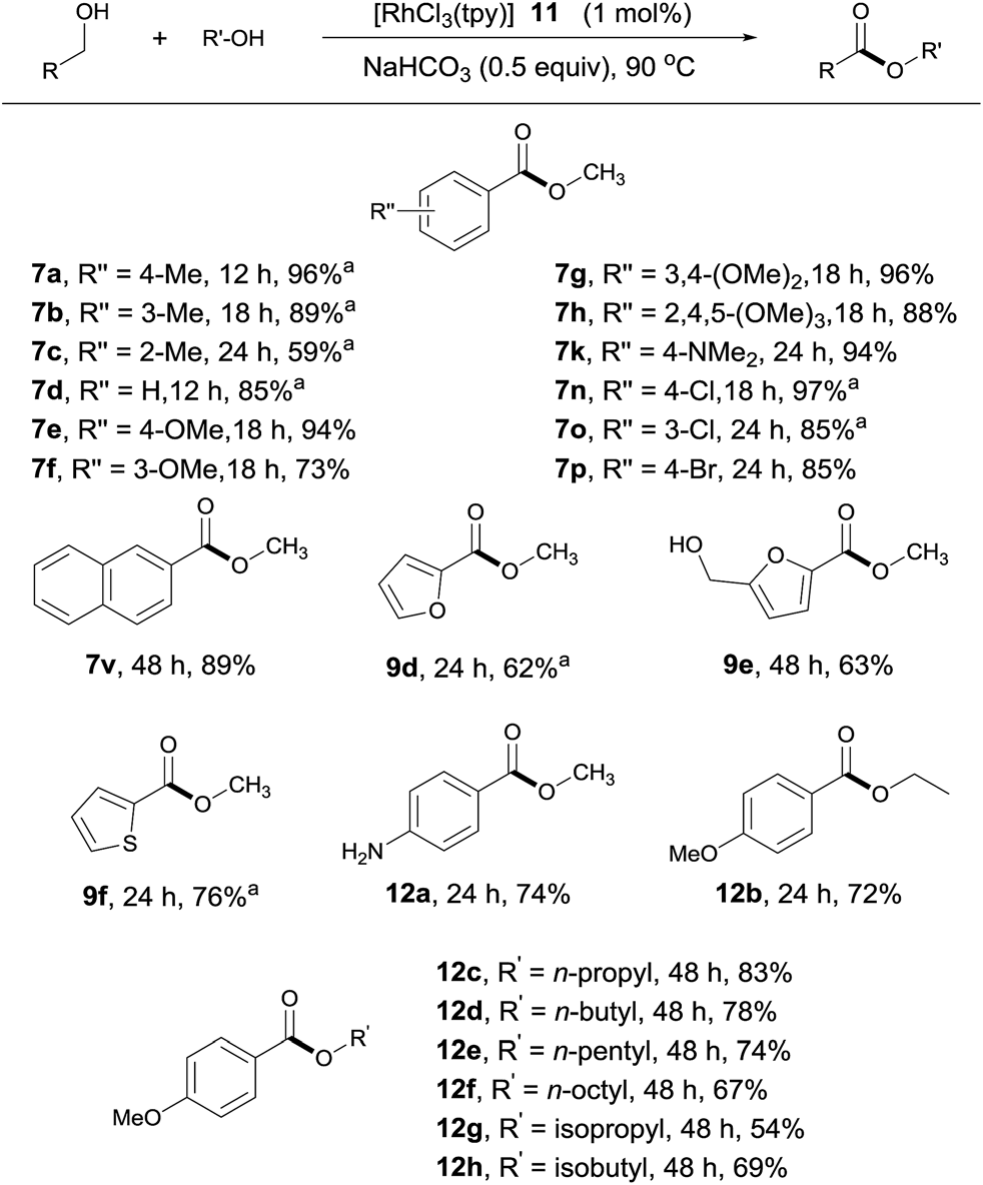
Cross-coupling of alcohols. Isolated yields are given, RCH_2_OH (0.5 mmol), R′OH (1 mL); see ESI for details.†^*a*^Yields determined by ^1^H NMR with an internal standard.

The substrate scope of the catalytic system appears to be quite general (Scheme 5). Thus, benzylic alcohols with various substituents at different positions of the aromatic ring reacted with MeOH, affording their methyl esters with good to excellent yields (Scheme 5, 7a–7h, 7k, 7n–7p, 7v, 12a). In comparison with the aldehyde–alcohol coupling, these reactions tend to be somewhat slower. As with the former, amino and halo substituents were tolerated. Interestingly, when 4-nitrobenzylalcohol was used as substrate, 12a was obtained as the product in 74% yield, with the nitro group being reduced to an amino group, indicative of the generation of metal hydride during the reaction. Other strongly electron-deficient benzyl alcohols, such as methyl 4-(hydroxymethyl)benzoate, showed little activity under the standard conditions, suggesting that the β hydrogen elimination step during dehydrogenation (*vide infra*) might be rate limiting. Heterocycle-containing alcohols also reacted, albeit with lower activities (Scheme 5, 9d–9f). Only one of the two hydroxyl groups reacted in product 9e, as the substrate becomes electron deficient after the first esterification. Likewise, aliphatic alcohols other than methanol were viable; but a longer reaction time or higher catalyst loading was required to obtain acceptable yields (Scheme 5, 12b–12h).

### 3. Identification of active catalyst

**Scheme 6:**
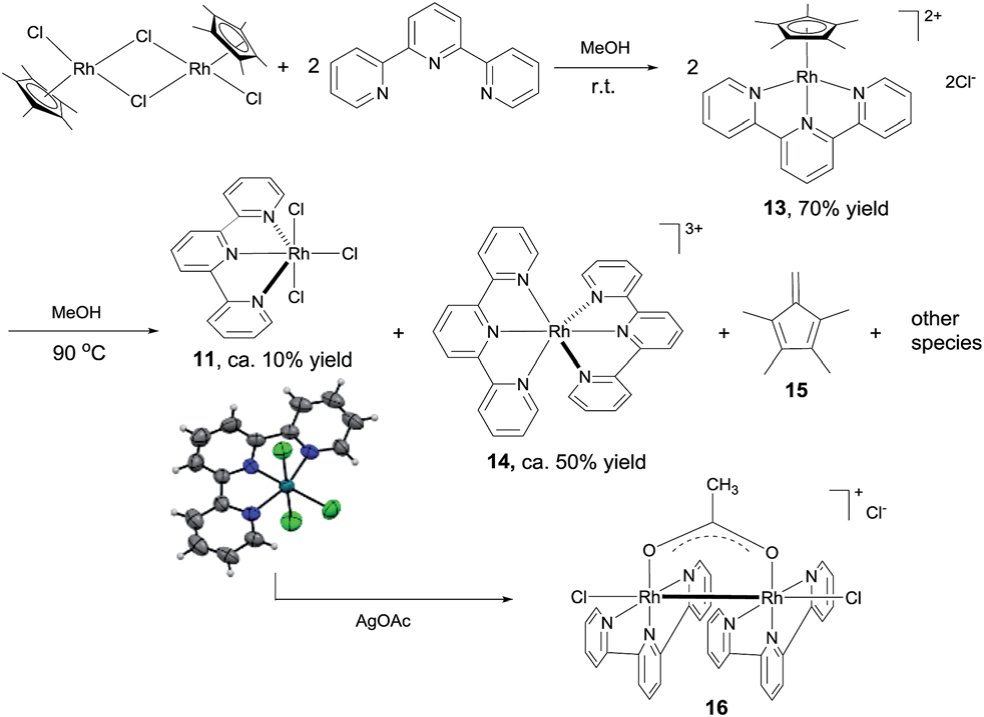
Identification of active catalytic species.

The results above suggest that complex 11 is a precatalyst for both types of cross coupling reactions. Prompted by this, we took a closer look at how it was formed and transferred into what active catalyst. Treating [Cp*RhCl_2_]_2_ with 2 equivalents of tpy *at room temperature* in MeOH for 1 h led to the complex 13 in 70% yield (Scheme 6). Using 13 as catalyst, 3,4-dimethoxybenzaldehyde was transformed to its methyl ester 7g in 37% yield in 3 h (Table S2†), indicating that 13 might be a precursor to 11. Indeed, stirring 13 or a mixture of [Cp*RhCl_2_]_2_ and 2 equivalents of tpy at 90 °C in MeOH for 6 h resulted in the formation of compounds 11, 14 and 15 and some unidentified species, with no 13 observed (Scheme 6). The structure of 11 was confirmed by X-ray diffraction.^16^ In the coupling of 3,4-dimethoxybenzaldehyde with MeOH, complex 11, 13 and 14 all displayed catalytic activity, with 14 being least active (ESI, Table S2†).

These results indicate that complex 11 is generated in the *in situ* catalytic reaction *via* the intermediate 13 and is the pre-catalyst for the cross coupling (Scheme 6). In fact, the analogous ruthenium and iridium complexes have been shown to catalyze alkylation of alcohols.^13*a*^ However, the fact that 11 is insoluble in MeOH in the absence of a base indicates that it may have undergone further transformations under the catalytic conditions. Thus, the reaction between 11 and NaOAc was studied. The crude ^1^H NMR of the mixture resulting from treating 11 with excess NaOAc (40 equivalents) in MeOH at ambient temperature showed that 11 was fully converted into a small amount of 14 and a major new compound 16 (Scheme 6). Gratifyingly, pure form of 16 could be readily obtained by reacting 11 with 2 equivalents of AgOAc. The structure of 16 has been fully established by comparison its ^1^H NMR, IR and UV-Vis data with the published literature^11^ as well as ^13^C NMR and HRMS (see the ESI†). 16 was also observed by treating 14 with NaOAc in refluxing MeOH. These observations point to 16 being the active catalyst for the coupling reactions. Indeed, 16 was highly active in the coupling of 3,4-dimethoxybenzaldehyde with MeOH in comparison with the *in situ* catalyst, 11, 13 or 14 (ESI, Table S2†).

Preliminary studies indicate that the binuclear structure of 16 is preserved in the coupling. Thus, when 16 (0.025 mmol) was treated with 2 equivalents of 4-nitrobenzaldehyde and NaOAc in 1.5 mL of MeOH at 90 °C for 6 h, the crude ^1^H NMR showed that the aldehyde was converted to the ester and more interestingly, the characteristic resonances of the ligands of 16 remained unchanged. In fact, replacing 16 with either 11 or [Cp*RhCl_2_]_2_ + tpy in this reaction all gave similar ^1^H NMR signals attributable to 16 (ESI, Fig. S2†), lending further support to 16 being the active catalyst. Dimeric Rh(ii) complexes are well documented in the literature^17^ and some of them have been used as catalysts in organic reactions.^12*b*,18^

**Scheme 7:**
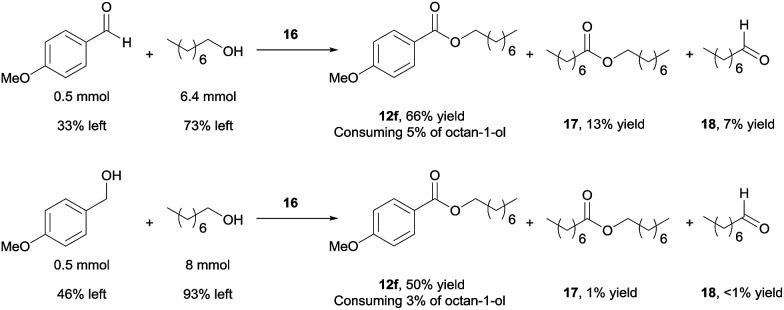
Reactions aimed to show the fate of access alcohols. The reaction conditions are the same for both reactions: 16 (2 mol%), NaOAc (1 equiv.), NaOH (5 mol%), 90 °C, 24 h. Yields were determined by ^1^H NMR with 1,3,5-trimethoxybenzene as internal standard.

Using 16 as catalyst, we further examined the chemoselectivity of the cross-coupling reactions. As shown in Scheme 7 (for more details, see ESI, Table S3†), reacting 0.5 mmol of 4-methoxybenzaldehyde with 6.4 mmol of octan-1-ol under the catalysis of 16 for 24 h, the desired cross-coupled ester (12f) was formed in 66% yield, with 33% of the aldehyde unchanged. Side products were observed for the octan-1-ol used in excess. The homo-coupled product octyl octanoate (17) was formed in 13% yield (based on octan-1-ol), along with 7% of octanal (18) derived from dehydrogenation of the alcohol. However, most of the octan-1-ol remained intact (73%). Under the same reaction conditions but increasing the aldehyde/alcohol ratio from 1 : 13 to 1 : 3, the selectivity was still good, albeit with a slower reaction. Thus, 12f was obtained in 41% yield, with 52% of the aldehyde unchanged and *ca.* 5% converted into a homo-coupled product (see ESI†). The yield of 17 and 18 decreased to 2% and 6%, respectively, in this case.

Better chemoselectivity was observed for the cross-coupling of alcohols (Scheme 7). The reaction between 0.5 mmol of 4-methoxybenzylalcohol with 8 mmol of octan-1-ol afforded 50% yield of 12f, with 46% of 4-methoxybenzylalcohol and 93% of octan-1-ol remained intact. Similarly, when the molar ratio of the two alcohols was changed from 1 : 16 to 1 : 3, 12f was obtained in 36% yield; only *ca.* 5% of homo-coupled ester product from 4-methoxybenzylalcohol and 5% of octyl octanoate were observed. Decarbonylation of the aldehyde was not observed in all cases.^19^ Taken together, the results demonstrate that the cross-coupling reactions are highly chemoselective when the aliphatic alcohol is used in excess and even when the quantity of alcohol is drastically reduced, the cross-coupled product still dominates, with the un-reacted alcohol remaining mostly intact. The mass balance of the benzylic substrates was excellent.

### 4. Proposed catalytic mechanism

**Scheme 8:**
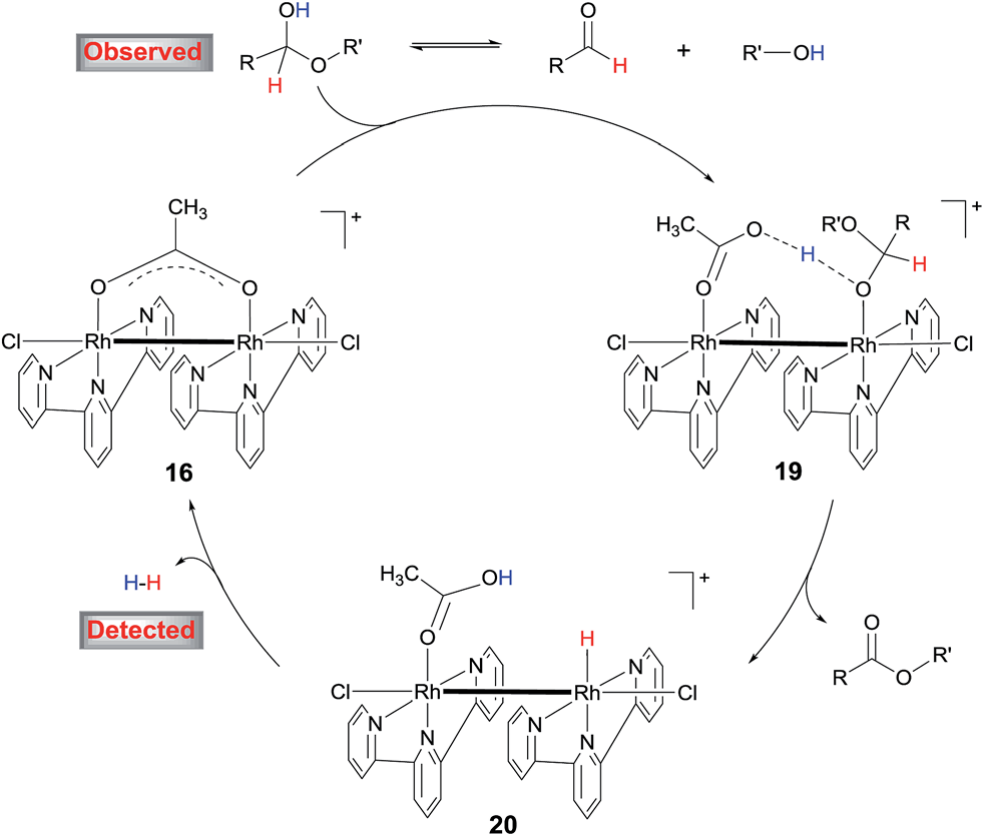
Proposed mechanism for the cross-coupling of aldehydes with alcohols.

On the basis of these results, a mechanism for the aldehyde–alcohol cross coupling is tentatively suggested (Scheme 8). The two substrates are in equilibrium with a hemiacetal intermediate, the characteristic resonance of which was observed in the ^1^H NMR of 4-nitrobenzaldehyde in CD_3_OD in the presence of NaOAc after heating for 5 min. Coordination of the hemiacetal to one of the Rh(ii) centres renders the hydroxyl proton more acidic such that it is readily deprotonated by the resulting, neighboring acetate in an intramolecular fashion, giving rise to 19. Elimination of the β hydrogen from 19 produces the ester and a hydride intermediate 20, which is intramolecularly protonated by the coordinated HOAc, releasing H_2_ while regenerating 16. The accelerating role of NaOH is not entirely clear at the moment; it may facilitate the formation of the hemiacetal or deprotonation of 19. In the case of alcohol cross-coupling, dehydrogenation of the aryl alcohol by the catalyst may occur first, affording an aldehyde, which then enters the same catalytic cycle, with HCO_3_^−^ replacing OAc^−^. An aldehyde intermediate was indeed observed by ^1^H NMR in the coupling of 4-methylbenzyl alcohol with MeOH.

## Conclusions

In conclusion, we have developed a novel catalytic system for dehydrogenative *cross-coupling* of aldehydes with alcohols as well as *cross-coupling* of primary alcohols to afford esters with H_2_ as the only by-product. The catalytic system shows broad substrate scope, providing an environmentally friendly alternative for ester preparation. A dimeric Rh(ii) complex was identified as the active catalyst, which appears to function *via* the cooperation of both Rh(ii) centres, with the base acting as a proton shuttle. Detailed mechanistic studies as well as further application of the dimeric rhodium complex in catalysis are underway in our laboratory.

## Supplementary Material

SC-007-C6SC00145A-s001
