# Correction: Chemoselective dehydrogenative esterification of aldehydes and alcohols with a dimeric rhodium(ii) catalyst

**DOI:** 10.1039/c7sc90054f

**Published:** 2017-07-28

**Authors:** Junjie Cheng, Meijuan Zhu, Chao Wang, Junjun Li, Xue Jiang, Yawen Wei, Weijun Tang, Dong Xue, Jianliang Xiao

**Affiliations:** a Key Laboratory of Applied Surface and Colloid Chemistry , Ministry of Education , School of Chemistry and Chemical Engineering , Shaanxi Normal University , Xi’an , 710062 , China . Email: c.wang@snnu.edu.cn; b Department of Chemistry , University of Liverpool , Liverpool , L69 7ZD , UK . Email: j.xiao@liverpool.ac.uk

## Abstract

Correction for ‘Chemoselective dehydrogenative esterification of aldehydes and alcohols with a dimeric rhodium(ii) catalyst’ by Junjie Cheng *et al.*, *Chem. Sci.*, 2016, **7**, 4428–4434.



## 

The authors regret that the incorrect temperature was stated on p. 4432, left column, line 5, of the original paper. The corrected sentence, in which “at ambient temperature” has been corrected to “at 90 °C”, is as follows:

“The crude ^1^H NMR of the mixture resulting from treating **11** with excess NaOAc (40 equivalents) in MeOH at 90 °C showed that **11** was fully converted into a small amount of **14** and a major new compound **16** (Scheme 6).”

The Royal Society of Chemistry apologises for these errors and any consequent inconvenience to authors and readers.

