# Supplementary material for: Chemoselective dehydrogenative esterification of aldehydes and alcohols with a dimeric rhodium(ii) catalyst
Source: Chem Sci. 2016 Mar 29;7(7):4428–34. doi: 10.1039/c6sc00145a (PMC6090528; doi:10.1039/c6sc00145a)
Supplement: SC-007-C6SC00145A-s001 [file SC-007-C6SC00145A-s001.pdf]

Electronic Supplementary Material (ESI) for Chemical Science.
This journal is © The Royal Society of Chemistry 2016

Chemoselective Dehydrogenative Esterification of Aldehydes and Alcohols with a Dimeric Rhodium(II) Catalyst

Junjie Cheng[a] *Meijuan Zhu,*[a] *Chao Wang,**[a] *Junjun Li,*[a] *Xue Jiang,*[a] *Yawen Wei,*[a] *Weijun Tang,*[a] *Dong Xue,*[a] *and Jianliang Xiao**[a,b]

[a] Key Laboratory of Applied Surface and Colloid Chemistry of Ministry of Education, and School of Chemistry and Chemical Engineering, Shaanxi Normal University, Xi'an, 710062, China, Email: c.wang@snnu.edu.cn
[b] Department of Chemistry, University of Liverpool, Liverpool, L69 7ZD, UK, Email: j.xiao@liverpool.ac.uk

Supporting Information

Contents

1. General information……………………………………..……………………...S2

2. Evidence for high chemoselectivity in cross-coupling of aldehyde with alcohol....S3

3. Optimization of conditions for cross-coupling of alcohols.……………………......S4

4. Comparison of activity of in situ catalyst and isolated complexes...................…S5

5. Evidence supporting **16** as the real catalyst………………………..………..S5-S6

6. Chemoselectivity studies for **16**……………….…………………….…..............S6-S8

7. Procedure for isolation of metal complexes…………………………………...S8-S8

8. General procedure for the esterification reaction……..…………………….. S9-S11

9. Detection of hydrogen gas……..…........…........………………..……...………..S11

10. Analytic data of metal complexes………………………..……………...S12-S13

11. Analytic data of intermediates and products………...…………....…...…...S14-S34

12. References...…….........…… S34-S35

13. Traces of ^{1}H NMR, ^{13}C NMR, HRMS, IR, UV, and GC spectra…......…S36-S100

14. Crystallographic data for complex **11**……………………………..……...S101-106

1. General information

Unless otherwise specified, the chemicals were obtained commercially and used without further purification. NMR spectra were recorded on a Bruker 400 MHz NMR spectrometer with TMS as the internal standard. GC and GC-MS analysis was carried out on an Agilent 7890A GC with a HP-5 MS column (quartz capillary column, 30 m x 0.25 mm x 0.25 μm) and an Agilent 5975C mass-selective detector (58 psi helium gas, 58 psi hydrogen gas, injector temperature 250 ℃, FID detector, temperature 300 ℃). The GC trace of hydrogen gas was recorded on a HT SP-6890 instrument with a 5 Å molecular sieves column (3 m x 4 mm) and a TCD detector. HRMS (ESI) data were recorded on a Bruker Apex IV FTMS spectrometer.

2. Evidence for high chemoselectivity in cross-coupling of aldehyde with alcohol

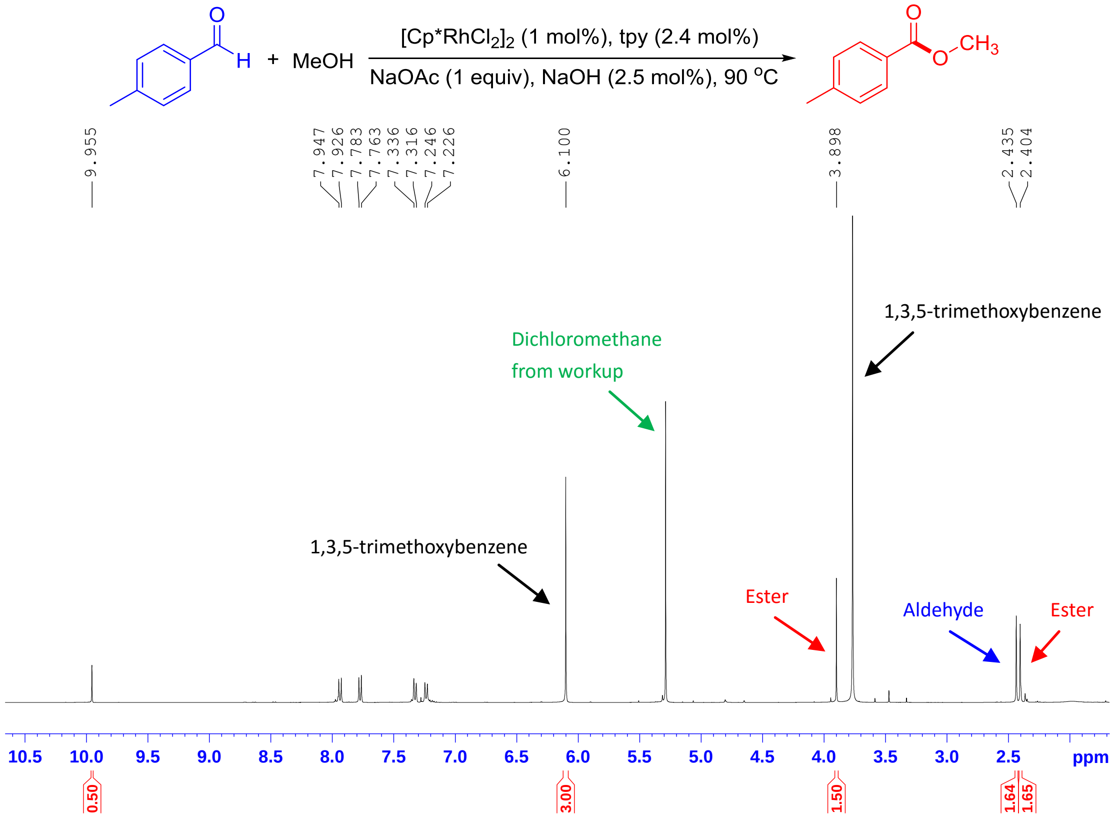

Figure S1. The crude ^{1}H NMR of a mixture resulting from reacting 0.5 mmol of 4-methylbenzaldehyde with 1.5 mmol MeOH (1,3,5-trimethoxybenzene as internal standard in $CDCl_3$). *The crude* ^{1}H *NMR showed that no 4-methylbenzylalcohol was observed in the reaction mixture*. Reaction conditions: 4-methylbenzaldehyde (0.5 mmol), $[Cp^*RhCl_2]_2$ (0.005 mmol), tpy (0.012 mmol), NaOH (0.012 mmol), NaOAc (0.5 mmol)，MeOH (1.5 mL), 90 ^{o}C, 12 h.

3. Optimisation of conditions for cross-coupling of alcohols

Table S1 The effect of base on **11** catalysed cross-coupling of alcohols

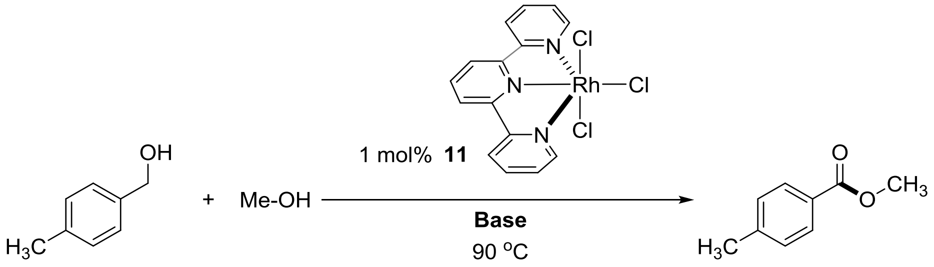

Entry	**Base**	Equivalent	Yield (%)
1	Pyridine	1	0
2	DBU	1	0
3	Triethylamine	1	40
4	Diisopropylamine	1	44
5	NaOAc	1	22
6	NaOH	1	20
7	*t*-BuOK	1	44
8	$NaHCO_3$	1	58
9	$NaHCO_3$	0.5	**70**

Reaction conditions: 4-methylbenzaldehyde (0.5 mmol), MeOH (2 mL), **11** (0.005 mmol), base, 90 ^{o}C. Yields were determined by ^{1}H NMR with 1,3,5-trimethoxybenzene as internal standard.

4. Comparison of activity of in situ catalyst and isolated complexes

Table S2 Results obtained in a model reaction

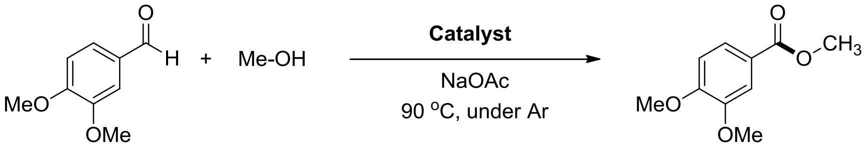

Entry	**Catalyst**	Time (h)	Yield (%)
1	$[Cp^*RhCl_2]_2$ + tpy in situ	3	23
2	**11**	3	33
3	**13**	3	37
4	**14**	3	9
5	**16**	3	**39**

Reaction conditions: aldehyde (0.5 mmol), NaOAc (0.5 mmol) (no NaOH added), MeOH (2 mL), under Ar atmosphere, 90 °C, substrate/Rh molar ratio = 50:1. Yields were determined by ^{1}H NMR with 1,3,5-trimethoxybenzene as internal standard.

5. Evidence supporting 16 as the real catalyst

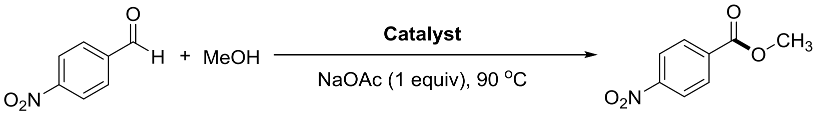

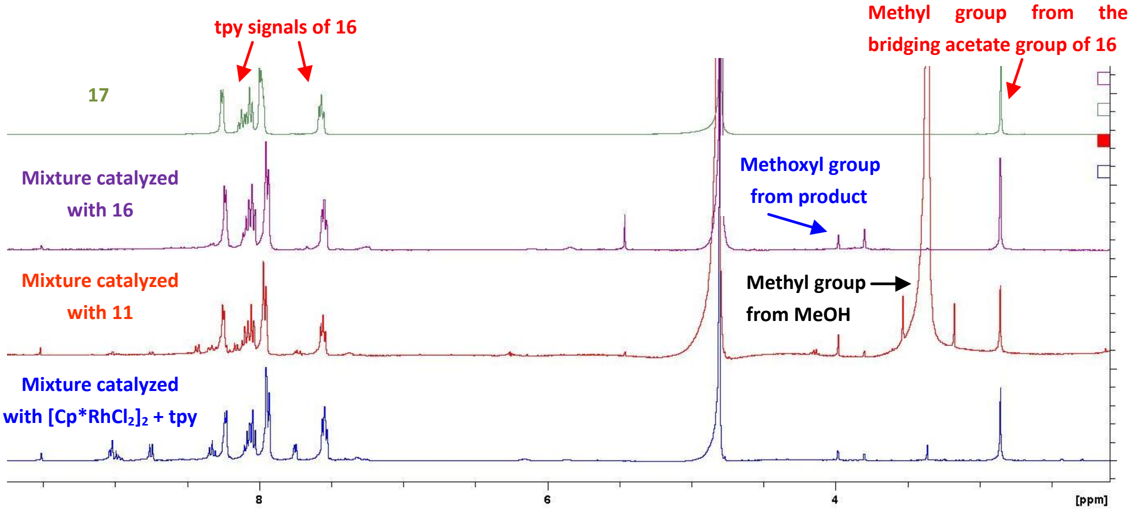

Figure S2. The ^{1}H NMR traces of **16** and those of reaction mixtures using 50% of **16**, **11**, and $[Cp^*RhCl_2]_2$ + tpy, respectively (D_2O as solvent). Reaction conditions: catalyst (0.025 mmol), *p*-nitrobenzaldehyde (0.05 mmol), NaOAc (0.05 mmol),

MeOH (1.5 mL), 90 °C, 6 h. After each reaction, MeOH was removed under reduced pressure and the mixture dissolved in D_2O for ^{1}H NMR measurement. The low intensity of the product peaks is due to its low solubility in D_2O.

6. Chemoselectivity studies for 16

Table S3 Chemoselectivity for **16** catalysed couplings

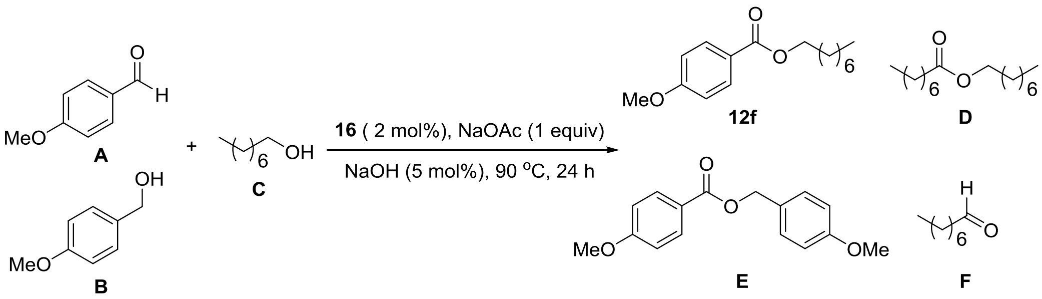

Substrates	Amount of **C**	**12f (%)**	**A** (%)	**B** (%)	**C** (%)	**D** (%)[a]	**E** (%)	**F** (%)[a]
**A+C**	6.4 mmol	66	33	trace	73	13	trace	7
**A+C**	1.5 mmol	41	52	0	62[b]	2	ca. 5	6
**B+C**	8 mmol	50	2	46	93	1	trace	<1
**B+C**	1.5 mmol	36	32	27	64[b]	5	ca. 5	10

Reaction conditions: **A** or **B** (0.5 mmol), octan-1-ol (1.5 mmol, 6.4 mmol or 8 mmol), **16** (2 mol%), NaOAc (0.5 mmol), NaOH (5 mol%), under Ar atmosphere with an empty balloon, 90 °C, 24 h. Yields were determined by ^{1}H NMR with 1,3,5-trimethoxybenzene as internal standard (0.2 mmol). [a] Yields based on octan-1-ol. [b] The mass balance for octan-1-ol was less than 100% when a relatively small amount of starting material was used, probably due to its evaporation.

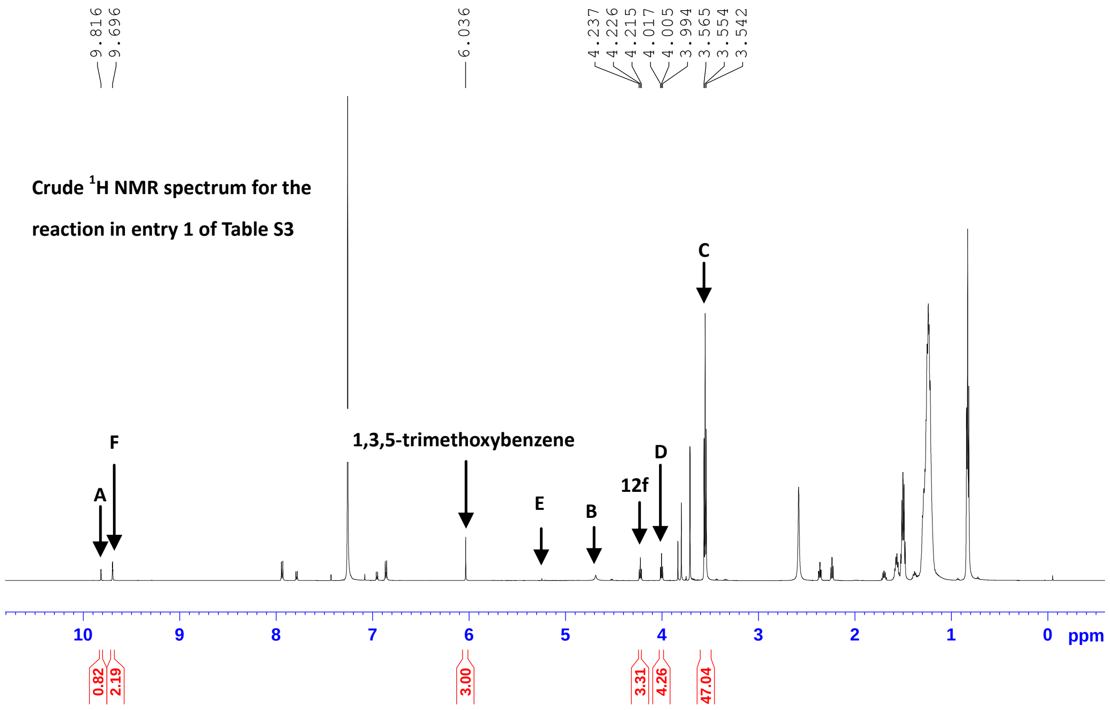
9.816
9.696
6.036
4.237
4.226
4.215
4.017
4.005
3.994
3.565
3.554
3.542
Crude ¹H NMR spectrum for the reaction in entry 1 of Table S3
C
F
A
1,3,5-trimethoxybenzene
D
12f
E
B
10
9
8
7
6
5
4
3
2
1
0
ppm
0.82
2.19
3.00
3.31
4.26
47.04

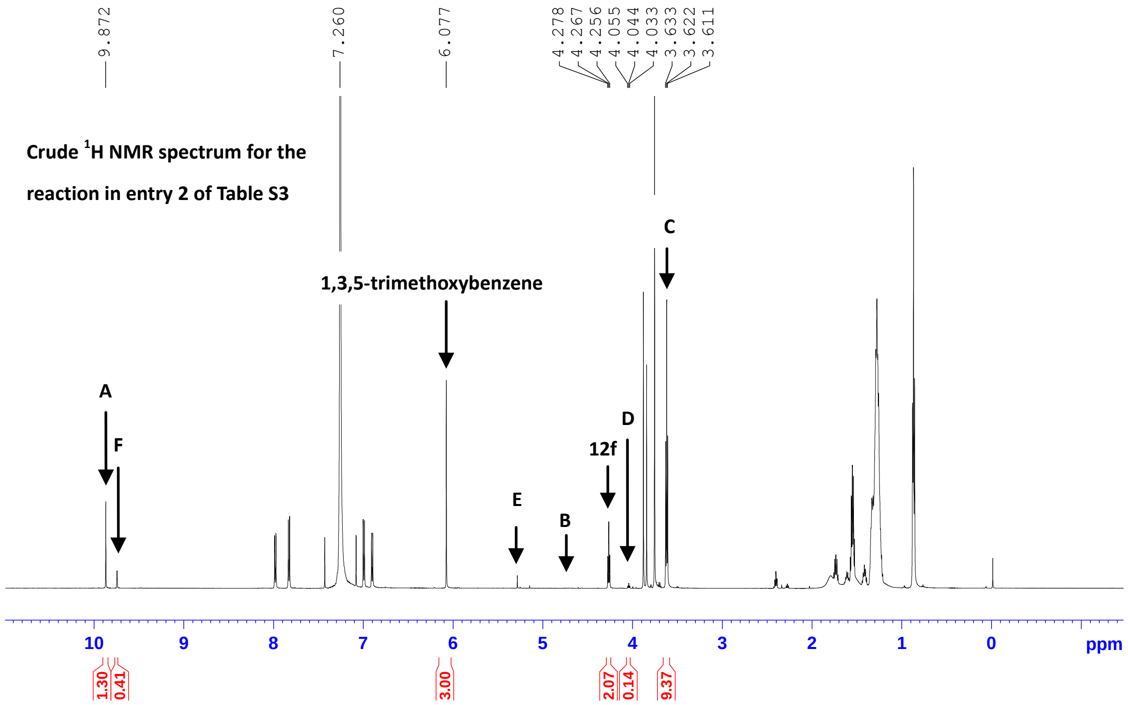
9.872
7.260
6.077
4.278
4.267
4.256
4.055
4.044
4.033
3.633
3.622
3.611
Crude ¹H NMR spectrum for the reaction in entry 2 of Table S3
C
1,3,5-trimethoxybenzene
A
F
D
12f
E
B
10
9
8
7
6
5
4
3
2
1
0
ppm
1.30
0.41
3.00
2.07
0.14
9.37

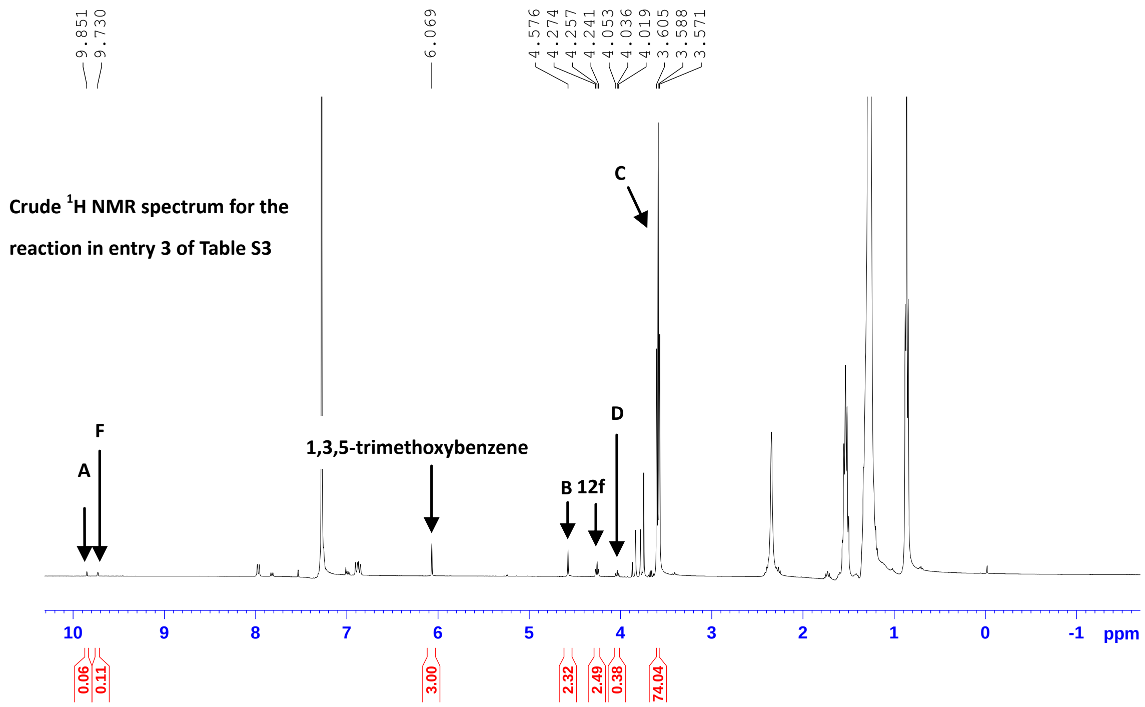
9.851
9.730
6.069
4.576
4.274
4.257
4.241
4.053
4.036
4.019
3.605
3.588
3.571
Crude ¹H NMR spectrum for the
reaction in entry 3 of Table S3
C
D
F
A
1,3,5-trimethoxybenzene
B 12f
10 9 8 7 6 5 4 3 2 1 0 -1 ppm
0.06
0.11
3.00
2.32
2.49
0.38
74.04

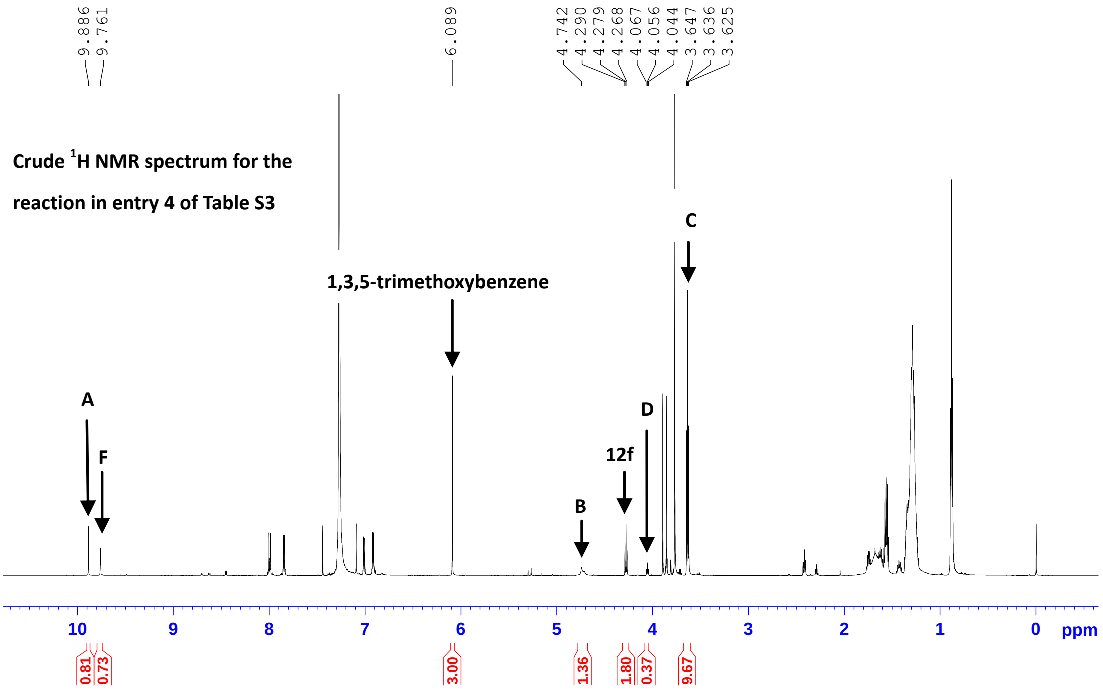
9.886
9.761
6.089
4.742
4.290
4.279
4.268
4.067
4.056
4.044
3.647
3.636
3.625
Crude ¹H NMR spectrum for the
reaction in entry 4 of Table S3
C
1,3,5-trimethoxybenzene
A
F
D
12f
B
10 9 8 7 6 5 4 3 2 1 0 ppm
0.81
0.73
3.00
1.36
1.80
0.37
9.67

7. Procedure for isolation of metal complexes

7.1 Formation of complex 13

$[Cp^*RhCl_2]_2$ (0.062 g, 0.1 mmol) was placed in a flask. MeOH (8 mL) and 2,2':6',2"-tripyridine (0.046 g, 0.2 mmol) were added, and the mixture was stirred for 60 min at room temperature. The solvent was evaporated, and the product purified by flash chromatography using methylene chloride and methanol as eluent. Yield: 0.071 g, 70%. Single crystals of **11** were formed in a methanol solution of **13** at room temperature.

7.2 Isolation of complexes 11 and 14

$[Cp^*RhCl_2]_2$ (0.062 mg, 0.1 mmol) was placed in a flask. MeOH (8 mL) and 2,2':6',2"-tripyridine (0.046 g, 0.2 mmol) were added, and the mixture was refluxed for 6 h. After evaporation of solvent, the residue was washed with 30 mL of methylene chloride. The resulting solid was then dissolved in methanol. The insoluble deep orange solid was identified to be **11** (ca. 10% yield), whilst evaporation of the solvent from the methanol solution gave an orange solid of **8**. Yield: 0.047 g, 50%.

7.3 Preparation of 11 with a known method[1]

A solution of $RhCl_3(H_2O)_3$ (0.253 g, 0.96 mmol) in EtOH (4 mL) was poured into a solution of 2,2':6',2"-tripyridine (0.224 g, 0.96 mmol) in 4 mL of EtOH. The dark orange mixture was heated under reflux for 2 h. After cooling to room temperature, the resulting yellow microcrystalline solid was filtered off, washed with EtOH and Et_2O sequentially, and then dried in vacuo. Yield: 0.425 g, 76%.

7.4 Isolation of complex 16[2]

CH_3COOAg (0.05 g, 0.3 mmol) and **11** (0.066 g, 0.15 mmol) were refluxed in methanol (6 mL) for 12 h. After cooling to rt, the reaction mixture was filtered. The solvent of the filtrate was removed under reduced pressure, and the residue washed with 30 mL of methylene chloride. The resulting solid was dissolved in 50 mL of methanol and the insoluble solid removed by filtration. The methanol solution was

concentrated to 15 mL, to which were added 5 mL of methylene chloride and 20 mL of petroleum ether sequentially. The resulting red precipitate was collected by filtration and dried in vacuo. Yield: 0.030 g, 48%.

7.5 Preparation of $[Rh(tpy)_2][PF_6]_3$[3]

$[RhCl_3(tpy)]$ (0.022 g, 0.05 mmol) and 2,2':6',2"-tripyridine (0.012 g, 0.05 mmol) and were refluxed in ethanol-water (1:1, 7 mL) for 5 h. The reaction mixture was filtered, and NH_4PF_6 (0.025 g, 0.15 mmol) was added to the filtrate. The pink precipitate thus produced was isolated by filtration and washed sequentially with 1.5 mL of water, ethanol and diethyl ether. The resulting solid was dissolved in a minimum volume of acetonitrile and then precipitated by addition of diethyl ether, yielding $[Rh(tpy)_2][PF_6]_3$ in 70% yield (0.035 g). This complex has the same cation as **14**.

8. General procedure for the esterification reaction

8.1 General procedure for coupling of aldehydes with alcohols

Aldehyde (0.5 mmol), $[Cp^*RhCl_2]_2$ (0.005 mmol), 2,2':6',2"-tripyridine (0.012 mmol), CH_3COONa (0.5 mmol), NaOH (0.0125 mmol), MeOH (2 mL) and a magnetic stir bar were placed in a Radleys Carousel tube. The mixture was bubbled with argon for 15 min and the tube was sealed and connected to an empty balloon. The mixture was then heated at 90 ℃ for 6-48 h. After cooling to room temperature, the mixture was extracted with methylene chloride (3 x 10 mL). The organic layers were washed with brine and dried over Na_2SO_4. The solvent was evaporated under reduced pressure and the product purified by flash chromatography using petroleum ether and ethyl acetate as eluent. The reactions of aldehydes with other alcohols were carried out with $[Cp^*RhCl_2]_2$ (0.010 mmol), 2,2':6',2"-tripyridine (0.024 mmol) and 1 mL of alcohol.

8.2 General procedure for cross-coupling of alcohols

A benzyl alcohol (0.5 mmol), **11** (0.005 mmol), $NaHCO_3$ (0.25 mmol), an aliphatic

alcohol (2 mL, but 1 mL for alcohols other than MeOH) and a magnetic stir bar were placed in a Radleys Carousel tube. The mixture was bubbled with argon for 15 min and the tube was sealed and connected to an empty balloon. The mixture was then heated at 90 ^{o}C for 12-48 h. After cooling to room temperature, the mixture was extracted with methylene chloride (3 x 10 mL). The organic layers were washed with brine and dried over Na_2SO_4. The solvent was evaporated under reduced pressure and the product purified by flash chromatography using petroleum ether and ethyl acetate as eluent.

9. Detection of hydrogen gas

In a Radleys Carousel tube, *p*-nitrobenzaldehyde (2 mmol), methanol (4 mL), $[Cp^{*}RhCl_2]_2$ (2 mol%), tpy (4.8 mol%), and NaOAc (2 mmol) were stirred under a nitrogen atmosphere for 3 h at 90 ℃. The head gas was collected by a gas-tight syringe and analyzed by GC. H_2 was detected (Figure S3).

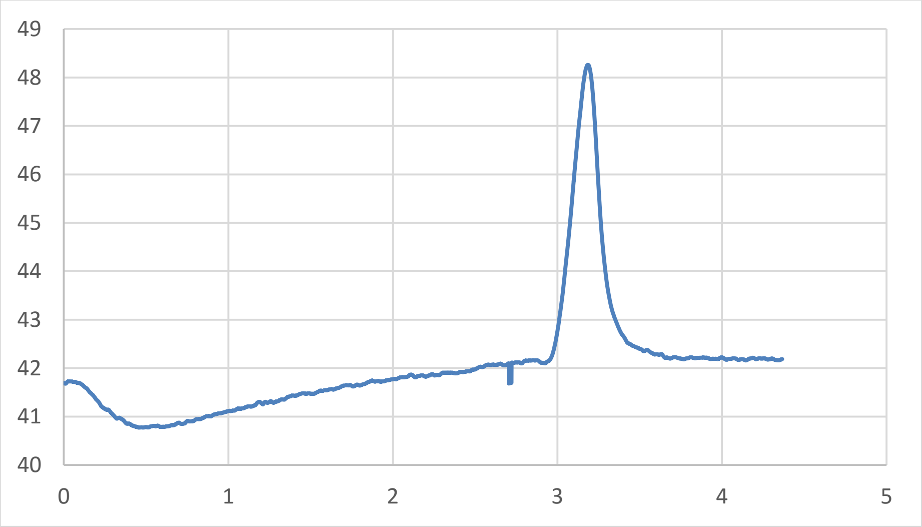

Figure S3. Detection of hydrogen gas with GC. GC parameters: injection temperature = 120 ^{o}C, column temperature = 70 ^{o}C, detector temperature = 120 ^{o}C. 5 Å molecular sieves column was used, and the carrier gas was N_2.

10. Analytic data of metal complexes

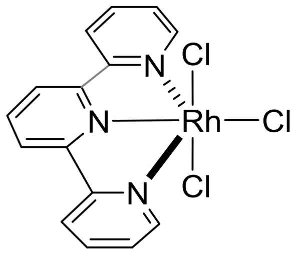

11

5:[1] deep orange solid; ^{1}H NMR (DMSO-d$_6$, 400 MHz) δ (ppm): 9.28 (d, *J* = 5.6 Hz, 2H), 8.82 (d, *J* = 8.0 Hz, 2H), 8.78 (d, *J* = 8.0 Hz, 2H), 8.55 (t, *J* = 8.0 Hz, 1H), 8.39 (t, *J* = 8.0 Hz, 2H), 7.94-7.98 (m, 2H). ^{13}C NMR (DMSO-d$_6$, 100 MHz) δ (ppm): 157.2, 155.2, 153.2, 140.5, 140.3, 128.4, 125.1, 124.4.

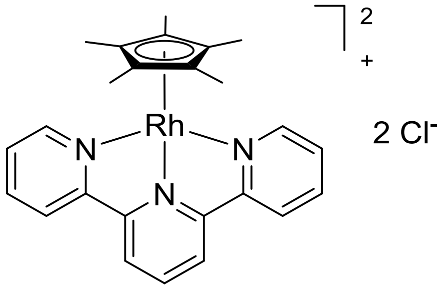

13

13: red solid; ^{1}H NMR (CD$_3$OD, 400 MHz) δ (ppm): 8.93 (d, *J* = 4.8 Hz, 2H), 8.87 (d, *J* = 8.0 Hz, 2H), 8.58 (d, *J* = 8.0 Hz, 2H), 8.44 (t, *J* = 8.0 Hz, 1H), 8.21 (td, *J* = 1.6, 8.0 Hz, 2H), 7.78-7.82 (m, 2H), 1.19 (s, 15H). ^{13}C NMR (CD$_3$OD, 100 MHz) δ (ppm): 161.0, 156.8, 152.7, 142.2, 140.5, 128.3, 128.1, 127.1, 99.0, 8.62; HRMS (ESI) for $C_{25}H_{26}ClN_3Rh[M+Cl]^+$: calc.: 506.0870; found: 506.0868.

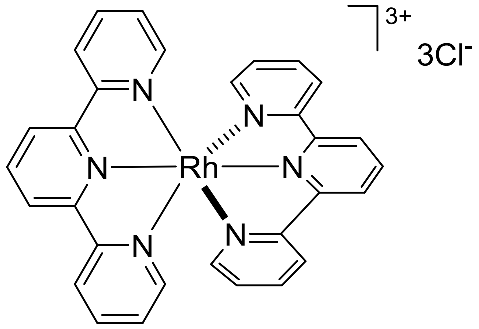

14

14: orange solid; ^{1}H NMR (CD$_3$OD, 400 MHz) δ (ppm): 9.17 (d, *J* = 8.0 Hz, 2H),

9.01 (t, J = 8.0 Hz, 1H), 8.92 (d, J = 8.0 Hz, 2H), 8.39 (td, J = 8.0, 1.2 Hz, 2H), 7.91 (d, J = 5.6 Hz, 2H), 7.60-7.64 (m, 2H). ^{13}C NMR (CD_3OD, 100 MHz) δ (ppm): 158.5, 155.4, 153.8, 145.5, 144.2, 131.5, 129.0, 128.7; HRMS (ESI) for $C_{30}H_{22}Cl_2N_6Rh$ $[M+2Cl]^+$: calc.: 639.0338; found: 639.0348.

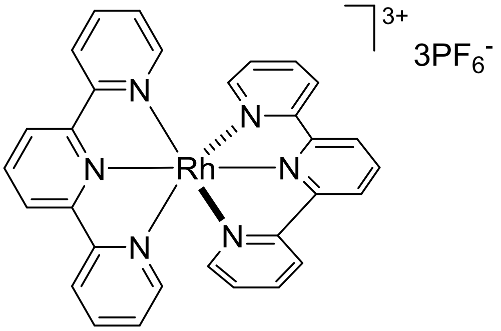

[[Rh(tpy)$_2$][PF$_6$]$_3$]:[3] pink solid; ^{1}H NMR (CD_3OD, 400 MHz) δ (ppm): 9.09 (d, J = 8.4 Hz, 2H), 8.94-8.98 (m, 1H), 8.83 (d, J = 8.4 Hz, 2H), 8.32-8.35 (m, 2H), 7.81 (d, J = 5.2 Hz, 2H), 7.55-7.56 (m, 2H).

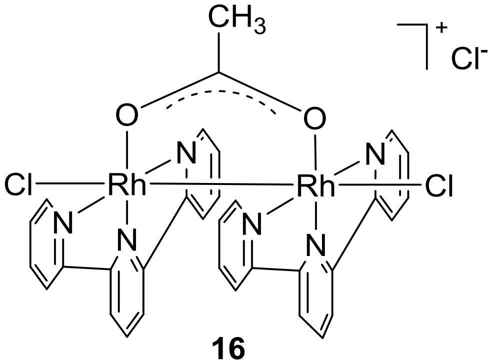

16

16:[2] red solid; ^{1}H NMR (D_2O, 400 MHz) δ (ppm): 8.14 (d, J = 5.6 Hz, 4H), 7.85-8.02 (m, 14H), 7.44 (t, J = 6.6 Hz, 4H), 2.72 (s, 3H). ^{13}C NMR (D_2O, 100 MHz) δ (ppm): 192.3, 155.2, 154.7, 151.5, 140.2, 139.2, 129.1, 124.6, 123.8, 24.5; HRMS (ESI) for $C_{32}H_{25}Cl_2N_6O_2Rh_2$ $[M]^+$: calc.: 800.9526; found: 800.9530; IR (in KBr, cm^{-1}): 1447 (V^s COO), 1546 (V^{as}, COO); UV-Vis (H_2O, nm): 230, 270, 315, 345.

11. Analytic data of intermediates and products

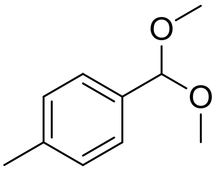

1-(Dimethoxymethyl)-4-methylbenzene: ^{1}H NMR ($CDCl_3$, 400 MHz) δ (ppm): 7.34 (d, *J* = 8.0 Hz, 2H), 7.18 (d, *J* = 8.0 Hz, 2H), 5.37 (s, 1H), 3.33 (s, 6H), 2.36 (s, 3H).

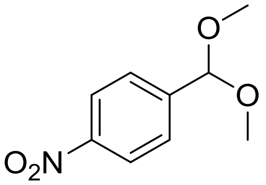

1-(Dimethoxymethyl)-4-nitrobenzene: ^{1}H NMR ($CDCl_3$, 400 MHz) δ (ppm): 8.21 (d, *J* = 8.4 Hz, 2H), 7.63 (d, *J* = 8.8 Hz, 2H), 5.46 (s, 1H), 3.33 (s, 6H).

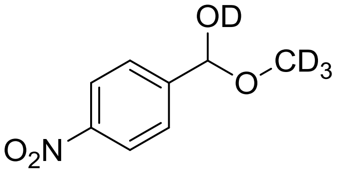

^{1}H NMR ($CDCl_3$, 400 MHz) δ (ppm): 8.19 (d, *J* = 8.8 Hz, 2H), 7.69 (d, *J* = 8.8 Hz, 2H), 5.60 (s, 1H).

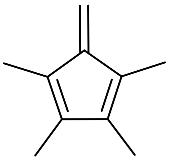

15

1,2,3,4-Tetramethyl-5-methylenecyclopenta-1,3-diene: ^{1}H NMR (CD_3OD, 400 MHz) δ (ppm): 5.41 (s, 2H), 1.86 (s, 6H), 1.81 (s, 6H). MS (EI) for $C_{10}H_{14}$ $[M]^+$: 134.

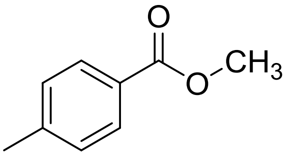

7a

Methyl 4-methylbenzoate:[4] Starting from aldehyde: substrate (0.5 mmol), [Cp*RhCl$_2$]$_2$ (0.005 mmol), tpy (0.012 mmol), NaOH (0.012 mmol) and NaOAc (0.5 mmol); 94% yield in 12 h; Starting from alcohol: RCH$_2$OH (0.5 mmol), MeOH (2 mL), **11** (0.005 mmol), NaHCO$_3$ (0.25 mmol); 96% yield in 12 h; both yields were determined by ^{1}H NMR analysis with 1,3,5-trimethoxybenzene as internal standard; colorless oil; ^{1}H NMR (CDCl$_3$, 400 MHz) δ (ppm): 7.92 (d, *J* = 8.4 Hz, 2H), 7.23 (d, *J* = 8.4 Hz, 2H), 3.90 (s, 3H), 2.41 (s, 3H); ^{13}C NMR (CDCl$_3$, 100 MHz) δ (ppm): 167.2, 143.5, 129.6, 129.1, 127.5, 51.9, 21.6; MS (EI) for $C_9H_{10}O_2$ $[M]^+$: 150.

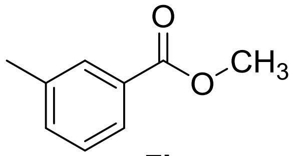

7b

Methl 3-methylbenzoate:[5] Starting from aldehyde: substrate (0.5 mmol), [Cp*RhCl$_2$]$_2$ (0.005 mmol), tpy (0.012 mmol), NaOH (0.012 mmol) and NaOAc (0.5 mmol); 92% yield in 12 h; Starting from alcohol: RCH$_2$OH (0.5 mmol), MeOH (2 mL), **11** (0.005 mmol), NaHCO$_3$ (0.25 mmol); 89% yield in 18 h; both yields determined by ^{1}H NMR analysis with 1,3,5-trimethoxybenzene as internal standard; colorless oil; ^{1}H NMR (CDCl$_3$, 400 MHz) δ (ppm): 7.83-7.86 (m, 2H), 7.26-7.36 (m, 2H), 3.90 (s, 3H), 2.39 (s, 3H), ^{13}C NMR (CDCl$_3$, 100 MHz) δ (ppm): 166.2, 137.1, 132.6, 129.1, 127.2, 125.6, 50.9, 20.2; MS (EI) for $C_9H_{10}O_2$ $[M]^+$: 150.

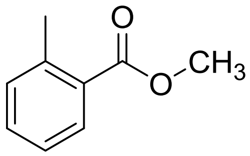

7c

Methyl 4-methylbenzoate:[5] Starting from aldehyde: substrate (0.5 mmol), [Cp*RhCl$_2$]$_2$ (0.005 mmol), tpy (0.012 mmol), NaOH (0.012 mmol) and NaOAc (0.5 mmol); 75% yield in 24 h; Starting from alcohol: RCH$_2$OH (0.5 mmol), MeOH (2 mL), **11** (0.005 mmol), NaHCO$_3$ (0.25 mmol); 59% yield in 24 h; both yields determined by ^{1}H NMR analysis with 1,3,5-trimethoxybenzene as internal standard; colorless oil; ^{1}H NMR (CDCl$_3$, 400 MHz) δ (ppm): 7.91-7.93 (m, 1H), 7.40 (td, *J* = 7.6, 1.2 Hz, 1H), 7.23-7.27 (m, 2H), 3.89 (s, 3H), 2.60 (s, 3H);^{13}C NMR (CDCl$_3$, 100

MHz) δ (ppm): 168.0, 140.1, 131.9, 131.6, 130.5, 129.5, 125.6, 51.7, 21.7; MS (EI) for $C_9H_{10}O_2$ $[M]^+$: 150.

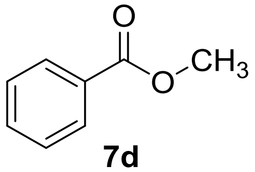

7d

Methyl benzoate:[4] Starting from aldehyde: substrate (0.5 mmol), $[Cp^*RhCl_2]_2$ (0.005 mmol), tpy (0.012 mmol), NaOH (0.012 mmol) and NaOAc (0.5 mmol); 90% yield in 12 h; Starting from alcohol: RCH_2OH (0.5 mmol), MeOH (2 mL), **11** (0.005 mmol), $NaHCO_3$ (0.25 mmol); 85% yield in 12 h; both yields determined by ^1H NMR analysis with 1,3,5-trimethoxybenzene as internal standard; ^1H NMR ($CDCl_3$, 400 MHz) δ (ppm): 8.04 (d, J = 6.8 Hz, 2H), 7.56 (t, J = 7.6 Hz, 1H), 7.44 (t, J = 7.6 Hz, 2H), 3.92 (s, 3H). ^{13}C NMR ($CDCl_3$, 100 MHz) δ (ppm): 167.1, 132.9, 130.2, 129.6, 128.3, 52.1; MS (EI) for $C_8H_8O_2$ $[M]^+$: 136.

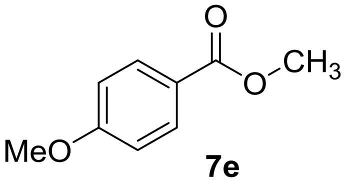

7e

Methyl 4-methoxybenzoate:[4] Starting from aldehyde: substrate (0.5 mmol), $[Cp^*RhCl_2]_2$ (0.005 mmol), tpy (0.012 mmol), NaOH (0.012 mmol) and NaOAc (0.5 mmol); 78 mg; 94% yield in 12 h; Starting from alcohol: RCH_2OH (0.5 mmol), MeOH (2 mL), **11** (0.005 mmol), $NaHCO_3$ (0.25 mmol); 94% yield in 18 h; white solid; m.p. = 49-50 ^oC; ^1H NMR ($CDCl_3$, 400 MHz) δ (ppm): 7.99 (d, J = 8.8 Hz, 2H), 6.91 (d, J = 8.8 Hz, 2H), 3.88 (s, 3H), 3.85 (s, 3H); ^{13}C NMR ($CDCl_3$, 100 MHz) δ (ppm): 166.9, 163.3, 131.6, 122.6, 113.6, 55.4, 51.8; MS (EI) for $C_9H_{10}O_3$ $[M]^+$: 166.

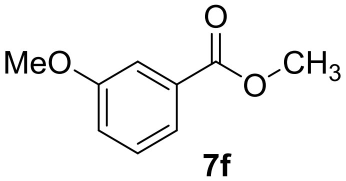

7f

Methyl 3-methoxybenzoate:[8] Starting from aldehyde: substrate (0.5 mmol), $[Cp^*RhCl_2]_2$ (0.005 mmol), tpy (0.012 mmol), NaOH (0.012 mmol) and NaOAc (0.5 mmol); 76 mg; 92% yield in 12 h; Starting from alcohol: RCH_2OH (0.5 mmol),

MeOH (2 mL), **11** (0.005 mmol), $NaHCO_3$ (0.25 mmol); 73% yield in 18 h; white solid; m.p. = 45-46 ^oC; ^1H NMR ($CDCl_3$, 400 MHz) δ (ppm): 7.63 (dt, *J* = 7.6, 1.2 Hz, 1H), 7.56 (q, *J* = 1.2 Hz, 1H), 7.34 (t, *J* = 7.6 Hz, 1H), 7.08-7.11(m, 1H), 3.91 (s, 3H), 3.84 (s, 3H); ^{13}C NMR ($CDCl_3$, 100 MHz) δ (ppm): 167.0, 159.6, 131.5, 129.4, 122.0, 119.5, 114.0, 55.4, 52.2; MS (EI) for $C_9H_{10}O_3$ $[M]^+$: 166.

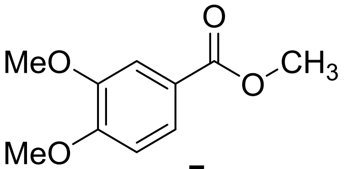

7g

Methyl 3,4-dimethoxybenzoate:[4] Starting from aldehyde: substrate (0.5 mmol), $[Cp^*RhCl_2]_2$ (0.005 mmol), tpy (0.012 mmol), NaOH (0.012 mmol) and NaOAc (0.5 mmol); 93 mg; 95% yield in 6 h; Starting from alcohol: RCH_2OH (0.5 mmol), MeOH (2 mL), **11** (0.005 mmol), $NaHCO_3$ (0.25 mmol); 96% yield in 18 h; white solid; m.p. = 55-56 ^oC; ^1H NMR ($CDCl_3$, 400 MHz) δ (ppm): 7.68 (dd, *J* = 8.4, 2.0 Hz, 1H), 7.54 (d, *J* = 2.0 Hz, 1H), 6.88 (d, *J* = 8.8 Hz, 1H), 3.93 (s, 3H), 3.927 (s,3H), 3.89 (s, 3H); ^{13}C NMR ($CDCl_3$, 100 MHz) δ (ppm): 166.9, 153.0, 148.6, 123.6, 122.7, 112.0, 110.3, 56.0, 52.0; MS (EI) for $C_{10}H_{12}O_4$ $[M]^+$: 196.

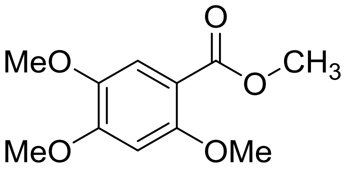

7h

Methyl 2,4,5-trimethoxybenzoate:[10] Starting from aldehyde: substrate (0.5 mmol), $[Cp^*RhCl_2]_2$ (0.005 mmol), tpy (0.012 mmol), NaOH (0.012 mmol) and NaOAc (0.5 mmol); 105 mg; 93% yield in 6 h; Starting from alcohol: RCH_2OH (0.5 mmol), MeOH (2 mL), **11** (0.005 mmol), $NaHCO_3$ (0.25 mmol); 88% yield in 18 h; white solid; m.p. = 89-90 ^oC; ^1H NMR ($CDCl_3$, 400 MHz) δ (ppm): 7.40 (s, 1H), 6.52 (s, 1H), 3.93 (s, 3H) 3.90 (s, 3H), 3.86 (s, 6H); ^{13}C NMR ($CDCl_3$, 100 MHz) δ (ppm): 166.1, 155.7, 153.6, 142.6, 114.5, 110.7, 97.8, 57.1, 56.4, 56.0, 51.8; MS (EI) for $C_{11}H_{14}O_5$ $[M]^+$: 226.

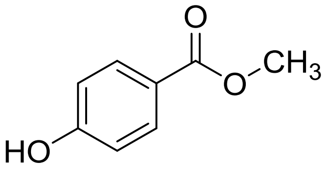

7i

Methyl 4-hydroxybenzoate:[4] Aldehyde substrate (0.5 mmol), $[Cp^*RhCl_2]_2$ (0.01 mmol), tpy (0.024 mmol), NaOH (0.024 mmol) and NaOAc (0.5 mmol); 65 mg; 86% yield in 24 h; white solid; m.p = 115-116 ^oC; ^1H NMR ($CDCl_3$, 400 MHz) δ (ppm): 7.95 (d, J = 8.4 Hz, 2H), 6.88 (d, J = 8.4 Hz, 2H), 3.90 (s, 3H); ^{13}C NMR ($CDCl_3$, 100 MHz) δ (ppm): 167.4, 160.2, 132.0, 122.4, 115.3, 52.1; MS (EI) for $C_8H_8O_3$ $[M]^+$: 152.

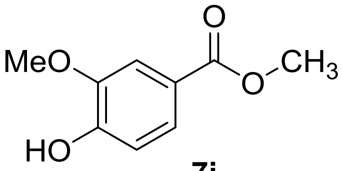

7j

Methyl 4-hydroxy-3-methoxybenzoate:[4] Aldehyde substrate (0.5 mmol), $[Cp^*RhCl_2]_2$ (0.005 mmol), tpy (0.012 mmol), NaOH (0.012 mmol) and NaOAc (0.5 mmol); 76 mg; 83% yield in 24 h; white solid; m.p. = 64-65 ^oC; ^1H NMR ($CDCl_3$, 400 MHz) δ (ppm): 7.64 (dd, J = 8.0, 1.6 Hz, 1H), 7.55 (d, J = 1.6 Hz, 1H), 6.94 (d, J = 8.0 Hz, 1H), 6.02, (s, 1H), 3.95 (s, 3H) 3.89 (s, 3H); ^{13}C NMR ($CDCl_3$, 100 MHz) δ (ppm): 166.9, 150.0, 146.2, 124.2, 122.3, 114.1, 111.7, 56.1, 52.0; MS (EI) for $C_9H_{10}O_{34}$ $[M]^+$: 182.

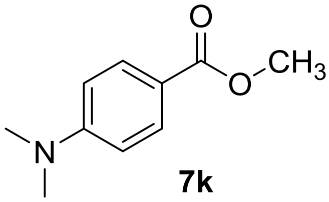

7k

Methyl 4-(dimethylamino)benzoate:[9] Starting from aldehyde: substrate (0.5 mmol), $[Cp^*RhCl_2]_2$ (0.005 mmol), tpy (0.012 mmol), NaOH (0.012 mmol) and NaOAc (0.5 mmol); 85 mg; 95% yield in 6 h; Starting from alcohol: RCH_2OH (0.5 mmol), MeOH (2 mL), **5** (0.005 mmol), $NaHCO_3$ (0.25 mmol); 94% yield in 24 h; white solid; m.p. = 101-102 ^oC; ^1H NMR ($CDCl_3$, 400 MHz) δ (ppm): 7.91 (d, J = 8.8 Hz, 2H), 6.65 (d,

J = 8.8 Hz, 2H), 3.85, (s, 3H), 3.04 (s, 6H); ^{13}C NMR ($CDCl_3$, 100 MHz) δ (ppm): 167.5, 153.3, 131.3, 117.0, 110.7, 51.5, 40.0; MS (EI) for $C_{10}H_{13}NO_2$ $[M]^+$: 179.

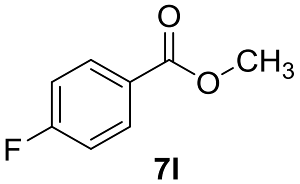

7l

Methyl 4-fluorobenzoate:[5] Aldehyde substrate (0.5 mmol), $[Cp^*RhCl_2]_2$ (0.005 mmol), tpy (0.012 mmol), NaOH (0.012 mmol) and NaOAc (0.5 mmol); 90% yield in 24 h; Yield was determined by ^{1}H NMR analysis with 1,3,5-trimethoxybenzene as internal standard; colorless oil; ^{1}H NMR ($CDCl_3$, 400 MHz) δ (ppm): 8.03-8.08 (m, 2H), 7.11 (t, J = 8.8 Hz, 2H), 3.91 (s, 3H); ^{13}C NMR ($CDCl_3$, 100 MHz) δ (ppm): 166.1, 165.8 (d, $^1J_{C\text{-}F}$ = 252.2 Hz), 132.1 (d, $^3J_{C\text{-}F}$ = 9.4 Hz), 126.4 (d, $^4J_{C\text{-}F}$ = 3.0 Hz), 115.5 (d, $^2J_{C\text{-}F}$ = 21.9 Hz), 52.2; MS (EI) for $C_8H_7FO_2$ $[M]^+$: 154.

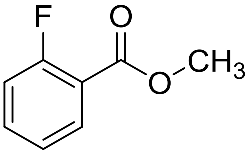

7m

Methyl 2-fluorobenzoate:[9] Aldehyde substrate (0.5 mmol), $[Cp^*RhCl_2]_2$ (0.005 mmol), tpy (0.012 mmol), NaOH (0.012 mmol) and NaOAc (0.5 mmol); 90% yield in 24 h; determined by ^{1}H NMR analysis with 1,3,5-trimethoxybenzene as internal standard; colorless oil; ^{1}H NMR ($CDCl_3$, 400 MHz) δ (ppm):7.94 (td, J = 2.0, 7.6 Hz, 1H), 7.49-7.54 (m, 1H), 7.18-7.22 (m, 1H), 7.10-7.16 (m, 1H); ^{13}C NMR ($CDCl_3$, 100 MHz) δ (ppm): 164.9 (d, $^4J_{C\text{-}F}$ = 3.6 Hz), 161.9 (d, $^1J_{C\text{-}F}$ = 258.1 Hz), 134.4 (d, $^3J_{C\text{-}F}$ = 9.0 Hz), 132.1, 123.9 (d, $^4J_{C\text{-}F}$ = 4.0 Hz), 118.6 (d, $^3J_{C\text{-}F}$ = 9.9 Hz), 116.9 (d, $^2J_{C\text{-}F}$ = 22.2 Hz), 52.3; MS (EI) for $C_8H_7FO_2$ $[M]^+$: 154.

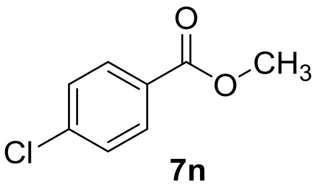

7n

Methyl 4-chlorobenzoate:[4] Starting from aldehyde: substrate (0.5 mmol), $[Cp^*RhCl_2]_2$ (0.005 mmol), tpy (0.012 mmol), NaOH (0.012 mmol) and NaOAc (0.5 mmol); 94% yield in 24 h; Starting from alcohol: RCH_2OH (0.5 mmol), MeOH (2

mL), **11** (0.005 mmol), $NaHCO_3$ (0.25 mmol); 97% yield in 18 h; both yields were determined by ^1H NMR analysis with 1,3,5-trimethoxybenzene as internal standard; colorless oil; ^1H NMR ($CDCl_3$, 400 MHz) δ (ppm): 7.96 (d, J = 8.4 Hz, 2H), 7.40 (d, J = 8.4 Hz, 2H), 3.90 (s, 3H); ^{13}C NMR ($CDCl_3$, 100 MHz) δ (ppm): 165.2, 138.3, 129.9, 127.7, 127.6, 51.2; MS (EI) for $C_8H_7ClO_2$ $[M]^+$: 170.

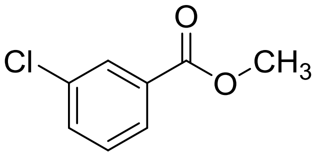

7o

Methyl 3-chlorobenzoate:[8] Starting from aldehyde: substrate (0.5 mmol), $[Cp*RhCl_2]_2$ (0.005 mmol), tpy (0.012 mmol), NaOH (0.012 mmol) and NaOAc (0.5 mmol); 92% yield in 24 h; Starting from alcohol: RCH_2OH (0.5 mmol), MeOH (2 mL), **11** (0.005 mmol), $NaHCO_3$ (0.25 mmol); 85% yield in 24 h; both yields were determined by ^1H NMR analysis with 1,3,5-trimethoxybenzene as internal standard; colorless oil; ^1H NMR ($CDCl_3$, 400 MHz) δ (ppm): 7.99-8.00 (m, 1H), 7.89-7.92 (m, 1H), 7.50-7.52 (m, 1H), 7.34-7.38 (m, 1H), 3.91 (s, 3H); ^{13}C NMR ($CDCl_3$, 100 MHz) δ (ppm): 165.8, 134.5, 132.9, 131.9, 129.7, 127.7, 52.4; MS (EI) for $C_8H_7ClO_2$ $[M]^+$: 170.

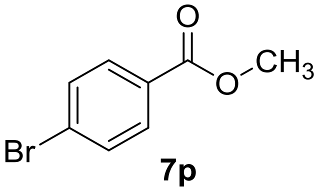

7p

Methyl 4-bromobenzoate:[5] Starting from aldehyde: substrate (0.5 mmol), $[Cp*RhCl_2]_2$ (0.01 mmol), tpy (0.024 mmol), NaOH (0.024 mmol) and NaOAc (0.5 mmol); 95 mg; 89% yield in 24 h; Starting from alcohol: RCH_2OH (0.5 mmol), MeOH (2 mL), **11** (0.01 mmol), $NaHCO_3$ (0.5 mmol); 85% yield in 24 h; white solid; m.p. = 77-78 ^oC; ^1H NMR ($CDCl_3$, 400 MHz) δ (ppm): 7.90 (d, J = 8.8 Hz, 2H), 7.58 (d, J = 8.8 Hz, 2H), 3.91 (s, 3H); ^{13}C NMR ($CDCl_3$, 100 MHz) δ (ppm): 166.4, 131.7, 131.1, 129.1, 128.0, 52.3; MS (EI) for $C_8H_7BrO_2$ $[M]^+$: 213.

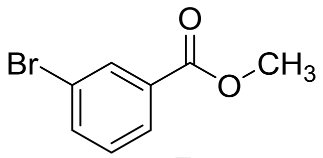

7q

Methyl 3-bromobenzoate:[4] Aldehyde substrate (0.5 mmol), $[Cp^*RhCl_2]_2$ (0.01 mmol), tpy (0.024 mmol), NaOH (0.024 mmol) and NaOAc (0.5 mmol); 93 mg; 87% yield in 24 h; colorless oil; ^{1}H NMR ($CDCl_3$, 400 MHz) δ (ppm): 8.17 (t, J = 1.6 Hz, 1H), 7.95-7.97 (m, 1H), 7.66-7.69 (m, 1H), 7.31 (t, J = 8.0 Hz, 1H), 3.92 (s, 3H); ^{13}C NMR ($CDCl_3$, 100 MHz) δ (ppm): 165.7, 135.8, 132.6, 132.0, 129.9, 128.1, 122.4, 52.4; MS (EI) for $C_8H_7BrO_2$ $[M]^+$: 213.

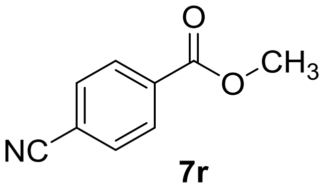

7r

Methyl 4-cyanobenzoate:[6] Aldehyde substrate (0.5 mmol), $[Cp^*RhCl_2]_2$ (0.005 mmol), tpy (0.012 mmol), NaOH (0.012 mmol) and NaOAc (0.5 mmol); 72 mg; 90% yield in 24 h; white solid; m.p. = 61-62 ^{o}C; ^{1}H NMR ($CDCl_3$, 400 MHz) δ (ppm): 8.14 (d, J = 8.4 Hz, 2H), 7.74 (d, J = 8.4 Hz, 2H), 3.96 (s, 3H); ^{13}C NMR ($CDCl_3$, 100 MHz) δ (ppm): 165.4, 134.0, 132.2, 130.1, 117.9, 116.4, 52.7; MS (EI) for $C_9H_7NO_2$ $[M]^+$: 161.

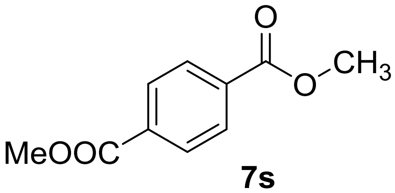

7s

Dimethyl terephthalate:[7] Aldehyde substrate (0.5 mmol), $[Cp^*RhCl_2]_2$ (0.005 mmol), tpy (0.012 mmol), NaOH (0.012 mmol) and NaOAc (0.5 mmol); 89 mg; 92% yield in 12 h; 85% yield in 24 h starting from terephthalaldehyde; white solid; m.p. = 134-135 ^{o}C; ^{1}H NMR ($CDCl_3$, 400 MHz) δ (ppm): 8.1 (s, 4H), 3.95 (s, 6H); ^{13}C NMR ($CDCl_3$, 100 MHz) δ (ppm): 166.3, 133.9, 129.6, 52.4; MS (EI) for $C_{10}H_{10}O_4$ $[M]^+$: 194.

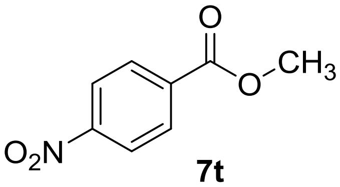

7t

Methyl 4-nitrobenzoate:[4] Aldehyde substrate (0.5 mmol), [Cp*RhCl$_2$]$_2$ (0.005 mmol), tpy (0.012 mmol), NaOAc (0.5 mmol); 83 mg; 92% yield in 6 h; white solid; m.p. = 87-88 ^{o}C; ^{1}H NMR (CDCl$_3$, 400 MHz) δ (ppm): 8.29 (d, *J* = 9.2 Hz, 2H), 8.21 (d, *J* = 8.8 Hz, 2H), 3.98 (s, 3H); ^{13}C NMR (CDCl$_3$, 100 MHz) δ (ppm): 165.2, 150.6, 135.5, 130.7, 123.5, 52.8; HRMS (ESI) for $C_8H_7NO_4$ [M+Na]$^+$: calc.: 204.0273; found: 204.0269.

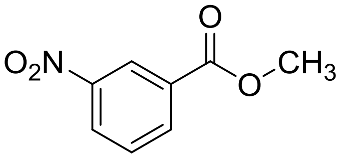

7u

Methyl 3-nitrobenzoate:[7] Aldehyde substrate (0.5 mmol), [Cp*RhCl$_2$]$_2$ (0.005 mmol), tpy (0.012 mmol), NaOAc (0.5 mmol); 83 mg; 91% yield in 6 h; white solid; m.p. = 72-73 ^{o}C; ^{1}H NMR (CDCl$_3$, 400 MHz) δ (ppm): 8.86-8.87 (m, 1H), 8.40-8.43 (m, 1H), 8.35-8.38 (m, 1H), 7.66 (t, *J* = 8.0 Hz, 1H), 3.99 (s, 3H); ^{13}C NMR (CDCl$_3$, 100 MHz) δ (ppm): 165.0, 148.3, 135.3, 131.9, 129.6, 127.4, 124.6, 52.8; MS (EI) for $C_8H_7NO_4$ [M]$^+$: 181.

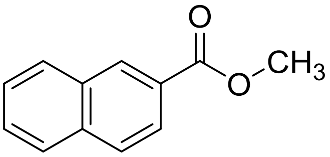

7v

Methyl 2-naphthpate:[6] Starting from aldehyde: substrate (0.5 mmol), [Cp*RhCl$_2$]$_2$ (0.005 mmol), tpy (0.012 mmol), NaOH (0.012 mmol) and NaOAc (0.5 mmol); 74 mg; 80% yield in 12 h; Starting from alcohol: RCH$_2$OH (0.5 mmol), MeOH (2 mL), **11** (0.005 mmol), NaHCO$_3$ (0.25 mmol); 89% yield in 48 h; white solid; m.p. = 72-73 ^{o}C; ^{1}H NMR (CDCl$_3$, 400 MHz) δ (ppm): 8.62 (s, 1H), 8.07 (dd, *J* = 8.8, 1.6 Hz, 1H), 7.96 (d, *J* = 8.0 Hz, 1H), 7.89 (d, *J* = 8.4 Hz, 2H), 7.53-7.62 (m, 2H), 3.99 (s, 3H); ^{13}C NMR (CDCl$_3$, 100 MHz) δ (ppm): 167.3, 135.5, 132.5, 131.1, 129.4, 128.23, 128.15, 127.8, 127.4, 126.6, 125.2, 52.2; MS (EI) for $C_{12}H_{10}O_2$ [M]$^+$: 186.

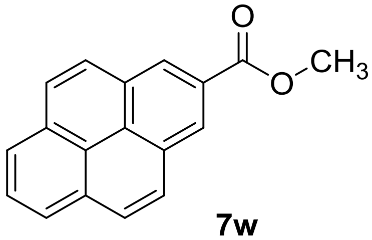

7w

Methyl 3a[1], 5a[1]-dihydropyrene-1-carboxylate: Aldehyde substrate (0.5 mmol), $[Cp^*RhCl_2]_2$ (0.005 mmol), tpy (0.012 mmol), NaOH (0.012 mmol) and NaOAc (0.5 mmol); 126 mg; 96% yield in 12 h; white solid; m.p. = 79-80 ^oC; ^1H NMR ($CDCl_3$, 400 MHz) δ (ppm): 9.27 (d, J = 9.6 Hz, 1H), 8.63 (d, J = 8.0 Hz, 1H), 8.23-8.28 (m, 3H), 8.16-8.19 (m, 2H), 8.04-8.09 (m, 2H), 4.11 (s, 3H); ^{13}C NMR ($CDCl_3$, 100 MHz) δ (ppm): 168.5, 134.3, 131.1, 131.0, 130.4, 129.6, 129.5, 128.4, 127.2, 126.32, 126.30, 126.2, 124.92, 124.86, 124.2, 124.1, 123.5, 52.3; HRMS (ESI) for $C_{18}H_{14}O_2$ $[M+Na]^+$: calc.: 283.0735; found: 283.0755.

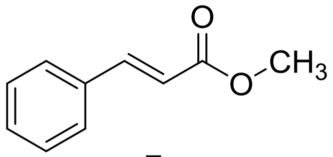

7x

Methyl cinnamate:[4] Aldehyde substrate (0.5 mmol), $[Cp^*RhCl_2]_2$ (0.005 mmol), tpy (0.012 mmol), NaOH (0.012 mmol) and NaOAc (0.5 mmol); 71 mg; 88% yield in 24 h; colorless oil; ^1H NMR ($CDCl_3$, 400 MHz) δ (ppm): 7.70 (d, J = 16 Hz, 1H), 7.51-7.54 (m, 2H), 7.37-7.39 (m, 3H), 6.45 (d, J = 16 Hz, 1H), 3.81 (s, 3H); ^{13}C NMR ($CDCl_3$, 100 MHz) δ (ppm): 167.4, 144.9, 134.4, 130.3, 128.9, 128.1, 117.8, 51.7; MS (EI) for $C_{10}H_{10}O_2$ $[M]^+$: 162.

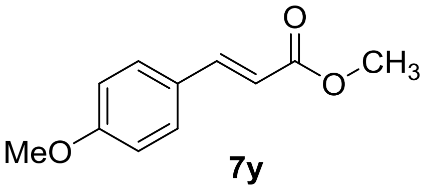

7y

(E)-methyl 3-(4-methoxyphenyl)acrylate:[19] Aldehyde substrate (0.5 mmol), $[Cp^*RhCl_2]_2$ (0.01 mmol), tpy (0.024 mmol), NaOH (0.024 mmol) and NaOAc (0.5 mmol); 77 mg; 80% yield in 24 h; white solid; m.p. = 81-82 ^oC; ^1H NMR ($CDCl_3$, 400 MHz) δ (ppm): 7.65 (d, J = 16.0 Hz, 1H), 7.48 (d, J = 8.8 Hz, 2H), 6.90 (d, J =

8.8 Hz, 2H), 6.31 (d, J = 16.0 Hz, 1H), 3.84 (s, 3H), 3.80 (s, 3H); ^{13}C NMR ($CDCl_3$, 100 MHz) δ (ppm): 167.8, 161.4, 144.5, 129.7, 127.1, 115.3, 114.3, 55.4, 51.6; MS (EI) for $C_{11}H_{12}O_3$ $[M]^+$: 192.

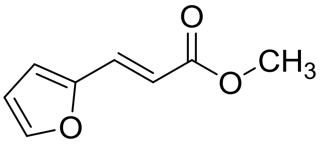

7z

(E)-methyl 3-(funan-2-yl)acrylate:[18] Aldehyde substrate (0.5 mmol), $[Cp^*RhCl_2]_2$ (0.01 mmol), tpy (0.024 mmol), NaOH (0.024 mmol) and NaOAc (0.5 mmol); 85% yield in 24 h; yield was determined by NMR analysis; colorless oil; ^{1}H NMR ($CDCl_3$, 400 MHz) δ (ppm): 7.46 (d, J = 1.2 Hz, 1H), 7.42 (d, J = 15.6 Hz, 1H), 6.59 (d, J = 3.6 Hz, 1H), 6.45 (dd, J = 3.6, 1.6 Hz, 1H), 6.31 (d, J = 15.6 Hz, 1H), 3.77 (s, 3H); ^{13}C NMR ($CDCl_3$, 100 MHz) δ (ppm): 167.4, 150.8, 144.7, 131.1, 115.4, 114.8, 112.2, 51.6; MS (EI) for $C_8H_8O_3$ $[M]^+$: 152.

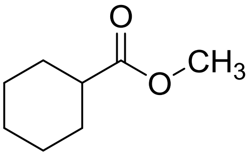

9a

Methyl cyclohexanecarboxylate:[22] 75% yield in 24 h determined by GC analysis; colorless oil; MS (EI) for $C_8H_{14}O_2$ $[M]^+$: Found: 142.

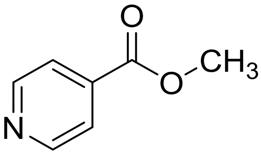

9b

Methyl isonicotinate:[13] Aldehyde substrate (0.5 mmol), $[Cp^*RhCl_2]_2$ (0.005 mmol), tpy (0.012 mmol), NaOH (0.012 mmol) and NaOAc (0.5 mmol); 87% yield in 24 h; determined by ^{1}H NMR analysis with 1,3,5-trimethoxybenzene as internal standard; colorless oil; ^{1}H NMR ($CDCl_3$, 400 MHz) δ (ppm): 8.69 (d, J = 4.4 Hz, 2H), 7.75 (d, J = 4.4 Hz, 2H), 3.87 (s, 3H); ^{13}C NMR ($CDCl_3$, 100 MHz) δ (ppm): 165.3, 150.4, 137.1, 122.6, 52.5; MS (EI) for $C_7H_7NO_2$ $[M]^+$: 137.

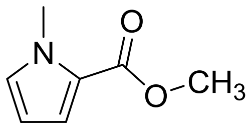

9c

Methyl 1-methyl-1 N-pyrrole-2-carboxylate:[14] Aldehyde substrate (0.5 mmol), $[Cp^*RhCl_2]_2$ (0.01 mmol), tpy (0.024 mmol), NaOH (0.024 mmol) and NaOAc (0.5 mmol); 75% yield in 48 h; yield was determined by ^{1}H NMR analysis with 1,3,5-trimethoxybenzene as internal standard; colorless oil; ^{1}H NMR ($CDCl_3$, 400 MHz) δ (ppm): 6.94 (dd, J = 4.0, 2.4 Hz, 1H), 6.78 (t, J = 2.0 Hz, 1H), 6.11 (dd, J = 4.0, 2.4 Hz, 1H), 3.92 (s, 3H), 3.81 (s, 3H); ^{13}C NMR ($CDCl_3$, 100 MHz) δ (ppm): 161.7, 129.4, 122.3, 117.7, 107.8, 50.9, 36.7; MS (EI) for $C_7H_9NO_2$ $[M]^+$: 139.

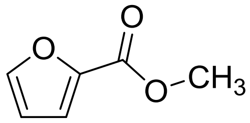

9d

Methyl funan-2-carboxylate:[4] Starting from aldehyde: substrate (0.5 mmol), $[Cp^*RhCl_2]_2$ (0.005 mmol), tpy (0.012 mmol), NaOH (0.012 mmol) and NaOAc (0.5 mmol); 80% yield in 24 h; Starting from alcohol: RCH_2OH (0.5 mmol), MeOH (2 mL), **11** (0.005 mmol), $NaHCO_3$ (0.25 mmol); 62% yield in 24 h; both yields were determined by ^{1}H NMR analysis with 1,3,5-trimethoxybenzene as internal standard; colorless oil; ^{1}H NMR ($CDCl_3$, 400 MHz) δ (ppm): 7.57 (s, 1H), 7.18 (d, J = 3.2 Hz, 1H), 6.51 (dd, J = 3.2, 1.6 Hz, 1H), 3.90 (s, 3H); ^{13}C NMR ($CDCl_3$, 100 MHz) δ (ppm): 159.1, 146.3, 144.6, 117.9, 111.8, 51.9; MS (EI) for $C_6H_6O_3$ $[M]^+$: 126.

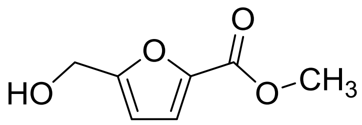

9e

Methyl 5-(hydroxymethyl)furan-2-carboxylate:[17] Starting from aldehyde: substrate (0.5 mmol), $[Cp^*RhCl_2]_2$ (0.005 mmol), tpy (0.012 mmol), NaOH (0.012 mmol) and NaOAc (0.5 mmol); 64 mg; 82% yield in 24 h; Starting from alcohol: RCH_2OH (0.5 mmol), MeOH (2 mL), **11** (0.01 mmol), $NaHCO_3$ (0.25 mmol); 63% yield in 48 h; colorless oil; ^{1}H NMR ($CDCl_3$, 400 MHz) δ (ppm): 7.08 (d, J = 3.6 Hz,

1H), 6.37 (d, J = 3.2 Hz, 1H), 4.62 (s, 2H), 3.84 (s, 3H), 3.32 (s, 1H); ^{13}C NMR ($CDCl_3$, 100 MHz) δ (ppm): 159.2, 158.6, 143.7, 118.9, 109.3, 57.3, 51.8; MS (EI) for $C_7H_8O_4$ $[M]^+$: 156.

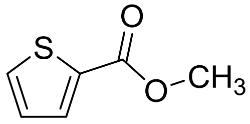

9f

Methyl thiophene-2-carboxylate:[5] Starting from aldehyde: substrate (0.5 mmol), $[Cp^*RhCl_2]_2$ (0.005 mmol), tpy (0.012 mmol), NaOH (0.012 mmol) and NaOAc (0.5 mmol); 82% yield in 24 h; Starting from alcohol: RCH_2OH (0.5 mmol), MeOH (2 mL), **11** (0.005 mmol), $NaHCO_3$ (0.25 mmol); 76% yield in 24 h; both yields were determined by ^1H NMR analysis with 1,3,5-trimethoxybenzene as internal standard; colorless oil; ^1H NMR ($CDCl_3$, 400 MHz) δ (ppm): 7.79 (dd, J = 4.0, 0.8 Hz, 1H), 7.54 (dd, J = 4.8, 0.8 Hz, 1H), 7.08-7.10 (m, 1H), 3.88 (s, 3H); ^{13}C NMR ($CDCl_3$, 100 MHz) δ (ppm): 162.5, 133.5, 133.4, 132.3, 127.7, 52.0; MS (EI) for $C_6H_6O_2S$ $[M]^+$: 142.

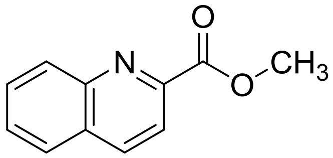

9g

Methyl quinoline-2-carboxylate:[14] Aldehyde substrate (0.5 mmol), $[Cp^*RhCl_2]_2$ (0.005 mmol), tpy (0.012 mmol), NaOH (0.012 mmol) and NaOAc (0.5 mmol); 75 mg; 80% yield in 24 h; white solid; m.p. = 77-78 °C; ^1H NMR ($CDCl_3$, 400 MHz) δ (ppm): 8.32 (dd, J = 8.4, 1.6 Hz, 2H), 8.21 (d, J = 8.4 Hz, 1H), 7.90 (dd, J = 8.0, 1.2 Hz, 1H), 7.78-7.82 (m, 1H), 7.64-7.68 (m, 1H), 4.09 (s, 3H); ^{13}C NMR ($CDCl_3$, 100 MHz) δ (ppm): 166.0, 147.9, 147.6, 137.3, 130.7, 130.3, 129.4, 128.7, 127.6, 121.0, 53.2; MS (EI) for $C_{11}H_9NO_2$ $[M]^+$: 187.

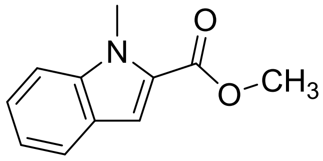

9h

Methyl 1-methyl-1N-indole-2-carboxylate:[15] Aldehyde substrate (0.5 mmol),

[Cp*RhCl$_2$]$_2$ (0.005 mmol), tpy (0.012 mmol), NaOH (0.012 mmol) and NaOAc (0.5 mmol); 91 mg; 96% yield in 24 h; white solid; m.p. = 90-91 ^{o}C; ^{1}H NMR (CDCl$_3$, 400 MHz) δ (ppm): 7.68 (d, *J* = 8.0 Hz, 1H), 7.34-7.41 (m, 2H), 7.30 (s, 1H), 7.14-7.18 (m, 1H), 4.09 (s, 3H), 3.92 (s, 3H); ^{13}C NMR (CDCl$_3$, 100 MHz) δ (ppm): 162.7, 139.7, 127.7, 125.9, 125.0, 122.6, 120.6, 110.2, 51.6, 31.6; MS (EI) for $C_{11}H_{11}NO_2$ [M]$^+$: 189.

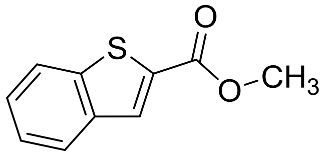

9i

Methyl benzo[*b*]thiophene-2-carboxylate:[16] Aldehyde substrate (0.5 mmol), [Cp*RhCl$_2$]$_2$ (0.005 mmol), tpy (0.012 mmol), NaOH (0.012 mmol) and NaOAc (0.5 mmol); 91 mg; 95% yield in 24 h; white solid; m.p. = 65-66 ^{o}C; ^{1}H NMR (CDCl$_3$, 400 MHz) δ (ppm): 8.07 (s, 1H), 7.86-7.89 (m, 2H), 7.44-7.48 (m, 1H), 7.39-7.43 (m, 1H), 3.95 (s, 3H); ^{13}C NMR (CDCl$_3$, 100 MHz) δ (ppm): 163.3, 142.2, 138.7, 133.3, 130.6, 127.0, 125.6, 124.9, 122.8, 52.5; MS (EI) for $C_{10}H_8O_2S$ [M]$^+$: 192.

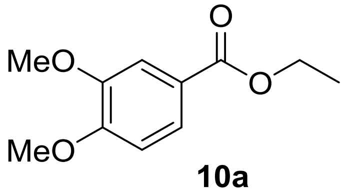

10a

Ethyl 3,4-dimethoxybenzoate:[20] : Aldehyde (0.5 mmol), alcohol (1 mL), [Cp*RhCl$_2$]$_2$ (0.01 mmol), tpy (0.024 mmol), NaOAc (0.5 mmol), NaOH (0.025 mmol); 79 mg; 75% yield in 48 h; white solid; m.p. = 35-36 ^{o}C; ^{1}H NMR (CDCl$_3$, 400 MHz) δ (ppm): 7.69 (dd, *J* = 8.4, 1.6 Hz, 1H), 7.55 (d, *J* = 1.6 Hz, 1H), 6.88 (d, *J* = 8.4 Hz, 1H), 4.36 (q, *J* = 7.2 Hz, 2H), 3.93 (s, 6H), 1.39 (t, *J* = 7.2 Hz, 3H); ^{13}C NMR (CDCl$_3$, 100 MHz) δ (ppm): 166.4, 152.9, 148.6, 123.5, 123.1, 112.0, 110.2, 60.8, 56.0, 14.4; MS (EI) for $C_{11}H_{14}O_4$ [M]$^+$: 210.

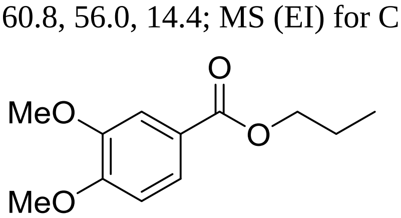

10b

Prpoyl 3,4-dimethoxybenzoate:[20] Aldehyde (0.5 mmol), alcohol (1 mL), [Cp*RhCl$_2$]$_2$ (0.01 mmol), tpy (0.024 mmol), NaOAc (0.5 mmol), NaOH (0.025 mmol); 87 mg; 78% yield in 48 h; white solid; m.p. = 56-57 ^{o}C; ^{1}H NMR (CDCl$_3$, 400 MHz) δ (ppm): 7.68 (dd, *J* = 8.4, 2.0 Hz, 1H), 7.54 (d, *J* = 2.0 Hz, 1H), 6.87 (d, *J* = 8.4 Hz, 1H), 4.24 (t, *J* = 6.8 Hz, 2H), 3.92 (s, 6H), 1.73-1.82 (m, 2H), 1.01 (t, *J* = 7.6 Hz, 3H); ^{13}C NMR (CDCl$_3$, 100 MHz) δ (ppm): 166.3, 152.8, 148.5, 123.4, 123.0, 111.9, 110.1, 66.3, 55.9, 22.1, 10.4; MS (EI) for $C_{12}H_{16}O_4$ [M]$^+$: 224.

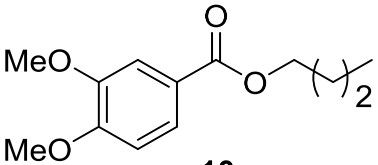

10c

Butyl 3,4-dimethoxybenzoate:[20] Aldehyde (0.5 mmol), alcohol (1 mL), [Cp*RhCl$_2$]$_2$ (0.01 mmol), tpy (0.024 mmol), NaOAc (0.5 mmol), NaOH (0.025 mmol); 83 mg; 70% yield in 48 h; white solid; m.p. = 153-154 ^{o}C; ^{1}H NMR (CDCl$_3$, 400 MHz) δ (ppm): 7.68 (dd, *J* = 8.4, 2.0 Hz, 1H), 7.55 (d, *J* = 2.0 Hz, 1H), 6.84 (d, *J* = 8.4 Hz, 1H), 4.30 (t, *J* = 6.8 Hz, 2H), 3.94 (s, 3H), 3.93 (s, 3H), 1.71-1.78 (m, 2H), 1.43-1.52 (m, 2H), 0.98 (t, *J* = 7.6 Hz, 3H); ^{13}C NMR (CDCl$_3$, 100 MHz) δ (ppm): 166.5, 152.9, 148.6, 123.5, 123.1, 112.0, 110.2, 64.7, 56.0, 30.9, 19.3, 13.8; MS (EI) for $C_{13}H_{18}O_4$ [M]$^+$: 238.

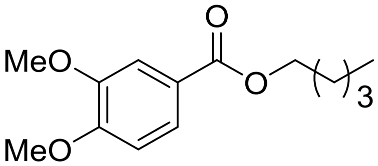

10d

Penyl 3,4-dimethoxybenzoate:[20] Aldehyde (0.5 mmol), alcohol (1 mL), [Cp*RhCl$_2$]$_2$ (0.01 mmol), tpy (0.024 mmol), NaOAc (0.5 mmol), NaOH (0.025 mmol); 102 mg; 82% yield in 24 h; white solid; m.p. = 39-40 ^{o}C; ^{1}H NMR (CDCl$_3$, 400 MHz) δ (ppm): 7.68 (dd, *J* = 8.4, 2.0 Hz, 1H), 7.54 (d, *J* = 2.0 Hz, 1H), 6.88 (d, *J* = 8.8 Hz, 1H), 4.29 (t, *J* = 6.4 Hz, 2H), 3.93 (s, 6 H), 1.73-1.80 (m, 2H), 1.36-1.44 (m, 4H), 0.93 (t, *J* = 7.2 Hz, 3H); ^{13}C NMR (CDCl$_3$, 100 MHz) δ (ppm): 166.4, 152.9, 148.6, 123.4, 123.1, 112.0, 110.2, 64.9, 55.9, 28.4, 28.2, 22.3, 13.9; MS (EI) for $C_{14}H_{20}O_4$ [M]$^+$: 252.

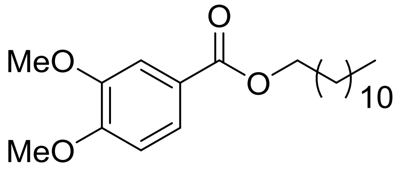

10e

Dodecyl 3,4-dimethoxybenzoate:[21] Aldehyde (0.5 mmol), alcohol (1 mL), $[Cp^*RhCl_2]_2$ (0.01 mmol), tpy (0.024 mmol), NaOAc (0.5 mmol), NaOH (0.025 mmol); 150 mg; 86% yield in 24 h; white solid; m.p. = 45-46 ^oC; ^1H NMR ($CDCl_3$, 400 MHz) δ (ppm): 7.68 (dd, *J* = 8.4, 2.0 Hz, 1H), 7.55 (d, *J* = 2.0 Hz, 1H), 6.88 (d, *J* = 8.4 Hz, 1H), 4.29 (t, *J* = 6.4 Hz, 2H), 3.93 (s, 6H), 1.72-1.79 (m, 2H), 1.39-1.45 (m, 2H), 1.20-1.39 (m, 16H), 0.88 (t, *J* = 6.4 Hz, 3H); ^{13}C NMR ($CDCl_3$, 100 MHz) δ (ppm): 166.5, 152.9, 148.6, 123.5, 123.1, 112.0, 110.2, 65.0, 56.0, 31.9, 29.65, 29.63, 29.58, 29.54, 29.34, 29.30, 28.8, 26.1, 22.7, 14.1; HRMS (ESI) for $C_{21}H_{34}O_4$ $[M+Na]^+$: calc.: 373.2355; found: 373.2334.

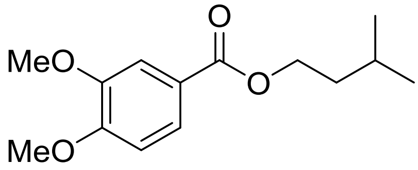

10f

Isopentyl 3,4-dimethoxybenzoate:[20] Aldehyde (0.5 mmol), alcohol (1 mL), $[Cp^*RhCl_2]_2$ (0.01 mmol), tpy (0.024 mmol), NaOAc (0.5 mmol), NaOH (0.025 mmol); 98 mg; 78% yield in 24 h; white solid; m.p. = 105-106 ^oC; ^1H NMR ($CDCl_3$, 400 MHz) δ (ppm): 7.67 (dd, *J* = 8.4, 2.0 Hz, 1H), 7.54 (d, *J* = 2.0 Hz, 1H), 6.88 (d, *J* = 8.4 Hz, 1H), 4.33 (t, *J* = 6.8 Hz, 2H), 3.933 (s, 3H), 3.930 (s, 3H), 1.74-1.84 (m, 1H), 1.66 (q, *J* = 6.8 Hz, 3H), 0.97 (d, *J* = 6.4 Hz, 6H), 0.97 (s, 3H); ^{13}C NMR ($CDCl_3$, 100 MHz) δ (ppm): 166.4, 152.9, 148.6, 123.1, 112.0, 110.2, 63.5, 56.0, 37.5, 25.3, 22.5: MS (EI) for $C_{14}H_{20}O_4$ $[M]^+$: 252.

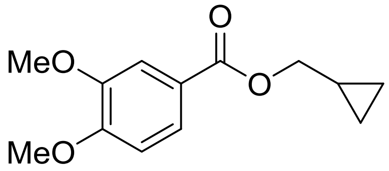

10g

Cyclopropylmethyl 3,4-dimethoxybenzoate: Aldehyde (0.5 mmol), alcohol (1 mL),

$[Cp^*RhCl_2]_2$ (0.01 mmol), tpy (0.024 mmol), NaOAc (0.5 mmol), NaOH (0.025 mmol); 39 mg; 33% yield in 48 h; white solid; m.p. = 44-45 °C; ^{1}H NMR ($CDCl_3$, 400 MHz) δ (ppm): 7.71 (dd, *J* = 8.4, 2.0 Hz, 1H), 7.56 (d, *J* = 1.6 Hz, 1H), 6.89 (d, *J* = 8.4 Hz, 1H), 4.13 (d, *J* = 7.2 Hz, 2H), 3.94 (s, 6H), 1.23-1.28 (m, 1H), 0.58-0.63 (m, 2H), 0.34-0.38 (m, 2H); ^{13}C NMR ($CDCl_3$, 100 MHz) δ (ppm): 166.5, 152.9, 148.6, 123.5, 123.1, 112.0, 110.2, 69.5, 56.0, 10.0, 3.3; HRMS (ESI) for $C_{13}H_{16}O_4$ $[M+Na]^+$: calc.: 259.0946; found: 259.0937.

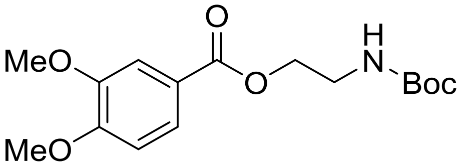

10h

2-((Tert-butoxycarbonyl)amino)ethyl 3,4-dimethbenzoate: Aldehyde (0.5 mmol), alcohol (1 mL), $[Cp^*RhCl_2]_2$ (0.01 mmol), tpy (0.024 mmol), NaOAc (0.5 mmol), NaOH (0.025 mmol); 81 mg; 50% yield in 48 h; white solid; m.p. = 90-91 °C; ^{1}H NMR ($CDCl_3$, 400 MHz) δ (ppm): 7.68 (dd, *J* = 8.4, 2.0 Hz, 1H), 7.54 (d, *J* = 2.0 Hz, 1H), 6.88 (d, *J* = 8.4 Hz, 1H), 4.84 (brs, 1H), 4.36 (t, *J* = 5.2 Hz, 2H), 3.939 (s, 3H), 3.935 (s, 3H), 3.52-3.53 (m, 2H), 1.44 (s, 9H); ^{13}C NMR ($CDCl_3$, 100 MHz) δ (ppm): 166.3, 155.8, 153.2, 148.7, 123.7, 122.4, 112.1, 110.2, 79.6, 64.1, 56.0, 39.9, 28.3; HRMS (ESI) for $C_{16}H_{22}NO_6$ $[M+Na]^+$: calc.: 348.1423; found: 348.1415.

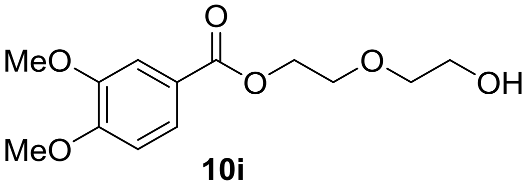

10i

4-Hydroxybutyl 3,4-dimethoxybenzoate: Aldehyde (0.5 mmol), alcohol (1 mL), $[Cp^*RhCl_2]_2$ (0.01 mmol), tpy (0.024 mmol), NaOAc (0.5 mmol), NaOH (0.025 mmol); 94 mg; 74% yield in 48 h; white solid; m.p. = 41-42 °C; ^{1}H NMR ($CDCl_3$, 400 MHz) δ (ppm): 7.69 (dd, *J* = 8.4, 1.6 Hz, 1H), 7.54 (d, *J* = 1.6 Hz, 1H), 6.88 (d, *J* = 8.4 Hz, 1H), 4.47 (t, *J* = 4.4 Hz, 2H), 3.93 (s, 3H), 3.927 (s, 3H), 3.83 (t, *J* = 4.4 Hz, 2H), 3.74 (s, 2H), 3.65 (t, *J* = 4.4 Hz, 2H), 2.19 (brs, 1H); ^{13}C NMR ($CDCl_3$, 100 MHz) δ (ppm): 166.4, 153.1, 148.6, 123.7, 122.5, 112.0, 110.2, 72.4, 69.3, 63.9, 61.8,

56.0; MS (EI) for $C_{13}H_{18}O_5$ $[M]^+$: 254.

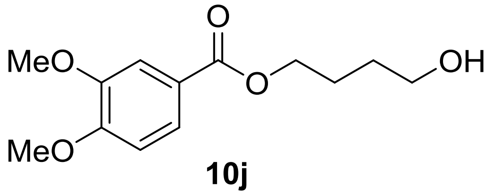

10j

2-(2-Hydroxyethoxy)ethyl 3,4-dimethoxybenzoate: Aldehyde (0.5 mmol), alcohol (1 mL), $[Cp^*RhCl_2]_2$ (0.01 mmol), tpy (0.024 mmol), NaOAc (0.5 mmol), NaOH (0.025 mmol); 101 mg; 75% yield in 48 h; white solid; m.p. = 43-44 °C; ^{1}H NMR ($CDCl_3$, 400 MHz) δ (ppm): 7.68 (dd, *J* = 8.4, 2.0 Hz, 1H), 7.54 (d, *J* = 2.0 Hz, 1H), 6.88 (d, *J* = 8.4 Hz, 1H), 4.34 (t, *J* = 6.4 Hz, 2H), 3.935 (s, 3H), 3.931 (s, 3H), 3.73 (t, *J* = 6.4 Hz, 2H), 1.83-1.90 (m, 2H), 1.69-1.76 (m, 2H), 1.44 (brs, 1H); ^{13}C NMR ($CDCl_3$, 100 MHz) δ (ppm): 166.4, 152.9, 148.6, 123.5, 122.8, 111.9, 110.2, 64.6, 62.4, 56.0, 29.2, 25.3; MS (EI) for $C_{13}H_{18}O_6$ $[M]^+$: 270.

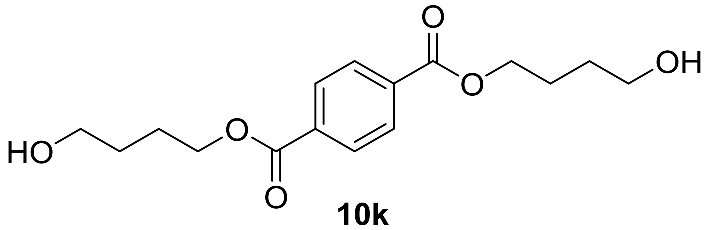

10k

4-Hydroxybutyl 4-(5-hydroxypentanoyl)benzoate: Aldehyde (0.5 mmol), alcohol (1 mL), $[Cp^*RhCl_2]_2$ (0.01 mmol), tpy (0.024 mmol), NaOAc (0.5 mmol), NaOH (0.025 mmol); 106 mg; 72% yield in 48 h; white solid; m.p. = 67-68 °C; ^{1}H NMR ($CDCl_3$, 400 MHz) δ (ppm): 8.066 (s, 2H), 8.064 (s, 2H), 4.37 (t, *J* = 6.4 Hz, 4H), 3.71 (t, *J* = 6.4 Hz, 4H), 1.97 (brs, 2H), 1.83-1.90 (m, 4H), 1.67-1.75 (m, 4H); ^{13}C NMR ($CDCl_3$, 100 MHz) δ (ppm): 165.9, 134.1, 129.5, 65.3, 62.3, 29.1, 25.2; HRMS (ESI) for $C_{16}H_{22}O_5$ $[M+Na]^+$: calc.: 333.1314; found: 333.1316.

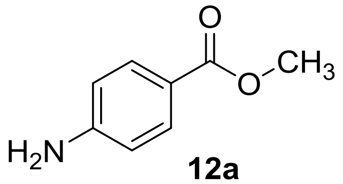

12a

Methyl 4-aminobenzoate:[12] RCH_2OH (0.5 mmol), MeOH (2 mL), **11** (0.005 mmol), $NaHCO_3$ (0.25 mmol); 56 mg; 74% yield in 24 h; white solid; m.p. = 103-104 °C; ^{1}H NMR ($CDCl_3$, 400 MHz) δ (ppm): 7.84 (d, *J* = 8.4 Hz, 2H), 6.63 (d, *J* = 8.8 Hz, 2H),

4.06 (brs, 2H), 3.85 (s, 3H); ^{13}C NMR ($CDCl_3$, 100 MHz) δ (ppm): 167.1, 150.7, 131.6, 119.7, 113.8, 51.6; MS (EI) for $C_8H_9NO_2$ $[M]^+$: 151.

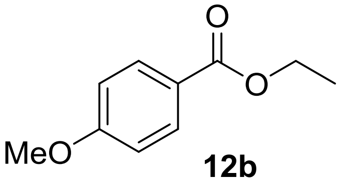

12b

Ethyl 4-methoxybenzoate:[23] RCH_2OH (0.5 mmol), R'OH (1 mL), **11** (0.005 mmol), $NaHCO_3$ (0.25 mmol), NaOH (0.05 mmol); 65 mg; 72% yield in 48 h; colorless oil; ^{1}H NMR ($CDCl_3$, 400 MHz) δ (ppm): 7.98 (d, J = 8.8 Hz, 2H), 6.89 (d, J = 8.8 Hz, 2H), 4.34 (q, J = 7.2 Hz, 2H), 3.83 (s, 3H), 1.36 (t, J = 7.2 Hz, 3H); ^{13}C NMR ($CDCl_3$, 100 MHz) δ (ppm): 166.4, 163.2, 131.5, 122.9, 113.5, 60.6, 55.4, 14.4; MS (EI) for $C_{10}H_{12}O_3$ $[M]^+$: 180.

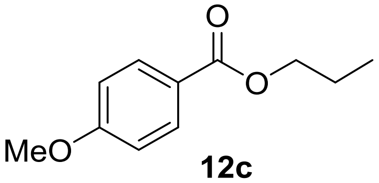

12c

Propyl 4-methoxybenzoate:[24] RCH_2OH (0.5 mmol), R'OH (1 mL), **11** (0.005 mmol), $NaHCO_3$ (0.25 mmol), NaOH (0.05 mmol); 80 mg; 83% yield in 48 h; colorless oil; ^{1}H NMR ($CDCl_3$, 400 MHz) δ (ppm): 8.01 (d, J = 8.8 Hz, 2H), 6.92 (d, J = 8.8 Hz, 2H), 4.25 (t, J = 6.8 Hz, 2H), 3.85 (s, 3H), 1.73-1.82 (m, 2H) , 1.02 (t, J = 7.2 Hz, 3H); ^{13}C NMR ($CDCl_3$, 100 MHz) δ (ppm): 166.5, 163.3, 131.5, 123.0, 113.6, 66.2, 55.4, 22.2, 10.5; MS (EI) for $C_{11}H_{14}O_3$ $[M]^+$: 194.

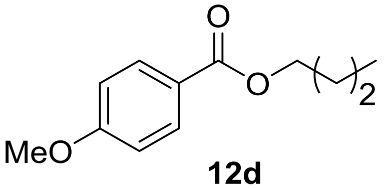

12d

Butyl 4-methoxybenzoate:[26] RCH_2OH (0.5 mmol), R'OH (1 mL), **11** (0.005 mmol), $NaHCO_3$ (0.25 mmol), NaOH (0.05 mmol); 81 mg; 78% yield in 48 h; colorless oil; ^{1}H NMR ($CDCl_3$, 400 MHz) δ (ppm): 8.00 (d, J = 9.2 Hz, 2H), 6.92 (d, J = 9.2 Hz, 2H), 4.29 (t, J = 6.4 Hz, 2H), 3.86 (s, 3H), 1.70-1.77 (m, 2H), 1.43-1.52 (m, 2H), 0.97 (t, J = 7.6 Hz, 3H); ^{13}C NMR ($CDCl_3$, 100 MHz) δ (ppm): 166.5, 163.2, 131.5, 123.0, 113.6, 64.5, 55.4, 30.9, 19.3, 13.8; MS (EI) for $C_{12}H_{16}O_3$ $[M]^+$: 208.

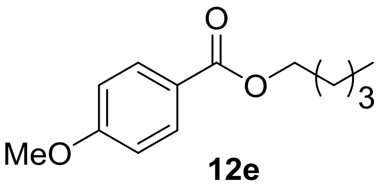

12e

Pentyl 4-methoxybenzoate:[25] RCH_2OH (0.5 mmol), R'OH (1 mL), **11** (0.005 mmol), $NaHCO_3$ (0.25 mmol), NaOH (0.05 mmol); 82 mg; 74% yield in 48 h; colorless oil; ^{1}H NMR ($CDCl_3$, 400 MHz) δ (ppm): 8.01 (d, J = 8.8 Hz, 2H), 6.92 (d, J = 9.2 Hz, 2H), 4.28 (t, J = 6.4 Hz, 2H), 3.86 (s, 3H), 1.72-1.79 (m, 2H), 1.37-1.43 (m, 4H), 0.93 (t, J = 7.2 Hz, 3H); ^{13}C NMR ($CDCl_3$, 100 MHz) δ (ppm): 166.5, 163.2, 131.5, 123.0, 113.6, 64.8, 55.4, 28.5, 28.2, 22.4, 14.0, MS (EI) for $C_{13}H_{18}O_3$ $[M]^+$: 222.

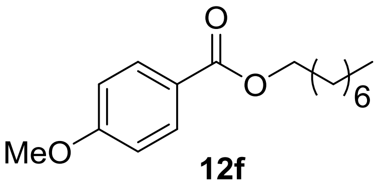

12f

Octyl 4-methoxybenzoate:[28] RCH_2OH (0.5 mmol), R'OH (1 mL), **11** (0.01 mmol), $NaHCO_3$ (0.25 mmol), NaOH (0.05 mmol); 88 mg; 67% yield in 48 h; colorless oil; ^{1}H NMR ($CDCl_3$, 400 MHz) δ (ppm): 7.99 (d, J = 9.2 Hz, 2H), 6.91 (d, J = 8.8 Hz, 2H), 4.27 (t, J = 6.4 Hz, 2H), 3.86 (s, 3H), 1.71-1.78 (m, 2H), 1.28-1.43 (m, 10H), 0.88 (t, J = 6.8 Hz, 3H); ^{13}C NMR ($CDCl_3$, 100 MHz) δ (ppm): 166.5, 163.2, 131.5, 123.0, 113.6, 64.9, 55.4, 31.8, 29.3, 29.2, 28.8, 26.1, 22.7, 14.1; HRMS (ESI) for $C_{16}H_{24}O_3$ $[M+Na]^+$: calc.: 287.1623; found: 287.1615.

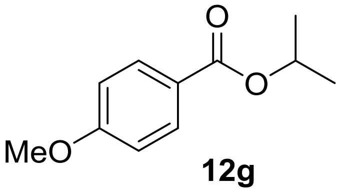

12g

Isopropyl 4-methoxybenzoate:[25] RCH_2OH (0.5 mmol), R'OH (1 mL), **11** (0.005 mmol), $NaHCO_3$ (0.25 mmol), NaOH (0.05 mmol); 52 mg; 54% yield in 48 h; colorless oil; ^{1}H NMR ($CDCl_3$, 400 MHz) δ (ppm): 8.00 (d, J = 9.2 Hz, 2H), 6.90 (d, J = 8.8 Hz, 2H), 5.18-5.27 (m, 1H), 3.86 (s, 3H), 1.36 (d, J = 6.4 Hz, 6H); ^{13}C NMR ($CDCl_3$, 100 MHz) δ (ppm): 166.5, 163.8, 132.1, 124.0, 114.1, 68.5, 56.0, 22.6; MS (EI) for $C_{11}H_{14}O_3$ $[M]^+$: 194.

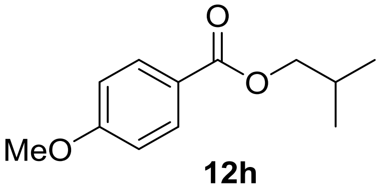

12h

Isobutyl 4-methoxybenzoate:[27] RCH_2OH (0.5 mmol), R'OH (1 mL), **11** (0.005 mmol), $NaHCO_3$ (0.25 mmol), NaOH (0.05 mmol); 72 mg; 69% yield in 48 h; colorless oil; ^1H NMR ($CDCl_3$, 400 MHz) δ (ppm): 8.00 (d, *J* = 8.8 Hz, 2H), 6.91 (d, *J* = 9.2 Hz, 2H), 4.06 (d, *J* = 6.4 Hz, 2H), 3.84 (s, 3H), 2.01-2.11 (m, 1H), 1.00 (d, *J* = 6.8 Hz, 6H); ^{13}C NMR ($CDCl_3$, 100 MHz) δ (ppm): 166.4, 163.3, 131.5, 123.0, 113.6, 70.7, 55.4, 27.9, 19.2, MS (EI) for $C_{12}H_{16}O_3$ $[M]^+$: 208.

12. References

[1] F. P. Pruchnik, P. Jakimowicz, Z. Ciunik, J. Zakrzewska-Czerwinska, A. Opolski, J.Wietrzyk, E. Wojdat, *Inorg. Chim. Acta*. **2002**, *334*, 59-66.

[2] F. P. Pruchnik, F. Robert, Y. Jeannin, S. Jeannin, *Inorg. Chem.* **1996**, *35*, 4261-4263.

[3] J. Paul, S. Spey, H. Adams, J. A. Thomas, *Inorg. Chim. Acta*. **2004,** *357*, 2827-2832.

[4] R. Gopinath, B. Barkakaty, B. Talukdar, B. K. Patel, *J. Org. Chem.* **2003**, *68*, 2944-2947.

[5] X. F. Wu, *Tetrahedron Lett*. **2012**, *53*, 3397-3399.

[6] R. Lerebours, C. Wolf, *J. Am. Chem. Soc*. **2006**, *128*, 13052-13053.

[7] B. R. Travis, M. Sivakumar, G. O. Hollist, B. Borhan, *Org. Lett*. **2003**, *5*, 1031-1034.

[8] E. G. Delany, C. L. Fagan, S. Gundala, A. Mari, *Chem. Commun*. **2013**, *49*, 6510-6512.

[9] S. K. Aavula, A. Chikkulapalli, N. Hanumanthappa, I. Jyothi, C. H. V. Kumar, S. G. Manjunatha, *Tetrahedron Lett.* **2013**, *54*, 5690-5694.

[10] Y. Zhu, H. Yan, L. H. Lu, D. F. Liu, G. W. Rong, J. C. Mao, *J. Org. Chem*. **2013**, *78*, 9898-9905.

[11] M. K. Agrawal, S. Adimurthy, P. K. Ghosh, *Synth. Commun.* **2012**, *42*, 2931-2936.

[12] I. Sorribes, G. Wienhofer, C. Vicent, K. Junge, R. Llusar, M. Beller, *Angew. Chem. Int. Ed*. **2012**, *51*, 7794-7798.

[13] K. K. Rajbongshi, M. J. Sarma, P. Phukan, *Tetrahedron Lett.* **2014**, *55*, 5358-5360.

[14] S. T. Heller, R. Sarpong, *Org. Lett*. **2010**, *12*, 4572-4575.

[15] K. Ukai, M. Aoki, J. Takaya, N. Iwasawa, *J. Am. Chem. Soc*. **2006**, *128*, 8706-8707.

[16] D. P. Hari, T. Hering, B. Konig, *Org. Lett*. **2012**, *14*, 5334-5337.

[17] C. Schmuck, U. Machon, *Eur. J. Org. Chem*. **2006**, *19*, 4385-4392.

[18] V. R. Chintareddy, A. Ellern, J. G. Verkade, *J. Org. Chem*. **2010**, *75*, 7166-7174.

[19] A. Derible, Y. C. Yang, P. H. Toy, J. M. Becht, C. L. Drian, *Tetrahedron Lett.* **2014**, *55*, 4331-4333.

[20] B. Narasimhan, S. Ohlan, R. Ohlan, V. Judge, R. Narang, *Eur. J. Med. Chem.* **2009**, *44*, 689-700.

[21] H. Nozary, C. P. P. Tissot, G. Bernardinell, *J. Am. Chem. Soc.* **1998**, *120*, 12274-12288.

[22] K. H. Park, K. Jang, H. J. Kim, S. U. Son, *Angew. Chem. Int. Ed*, **2007**, *46*, 1152-1155.
[23] J. Salvadori, E. Balducci, S. Zaza, E. Petricci, M. Taddei, *J. Org. Chem*, **2010**, *75*, 1841-1847.
[24] A. N. Rao, K. Ganesan, C. K. Shinde, *Synth. Commun.* **2012**, *42*, 2299-2308.
[25] X. S. Jia, H. L. Wang, Q. Huang, L. L. Kong, W. H. Zhang, *J. Chem. Res.* **2006,** 135-138.
[26] Q. Liu, G. Li, J. He, J. Liu, P. Li, A. W. Lei, *Angew. Chem. Int. Ed.* **2010**, *49,* 3371 -3374.
[27] J. I. Lee, *Bull. Kor. Chem. Soc.* **2011**, *32,* 1765-1768.
[28] M. Tamura, T. Tonomura, K. Shimizu, A. Satsumaa, *Green Chem.* **2012**, *14,* 984-991.

13. Traces of ^{1}H NMR, ^{13}C NMR, HRMS, IR and GC spectra

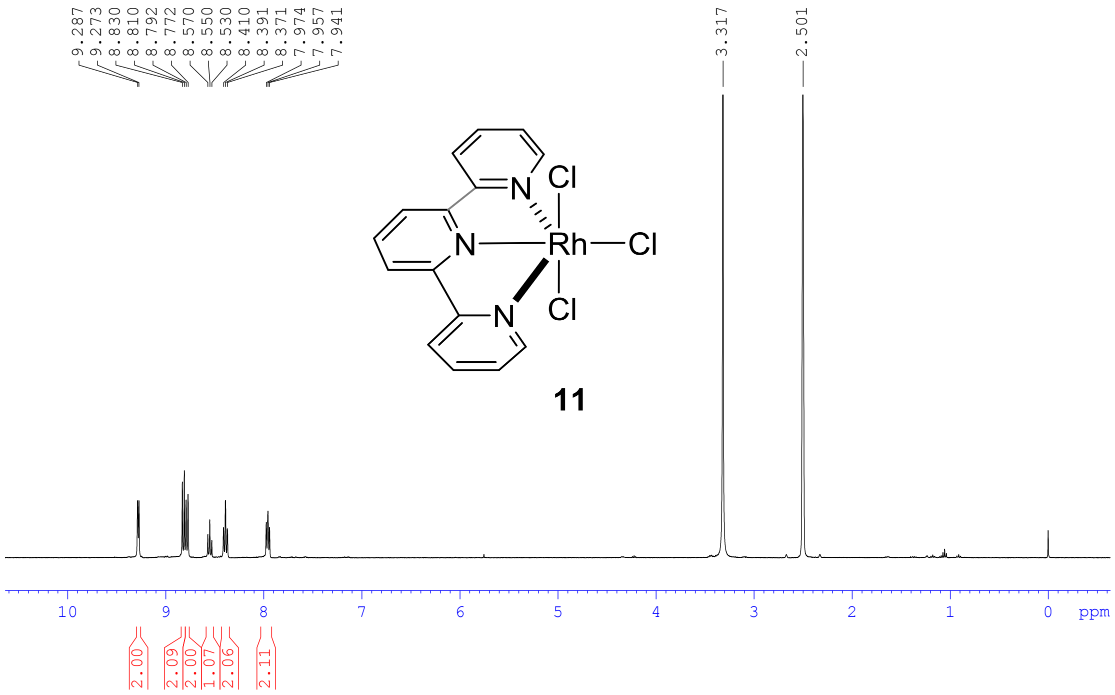

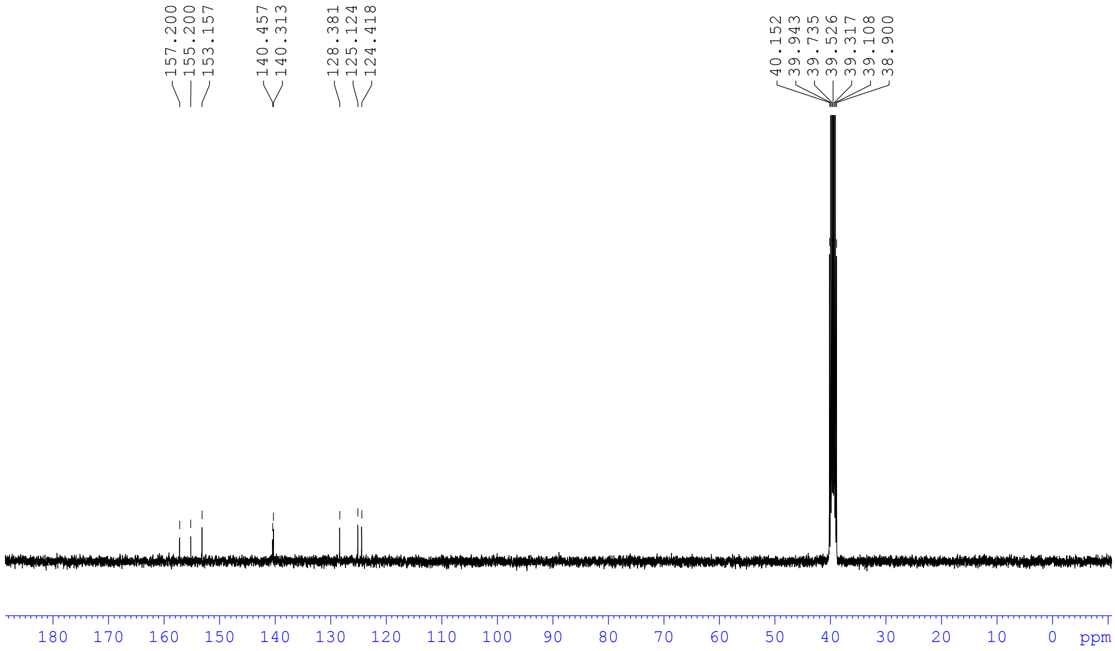

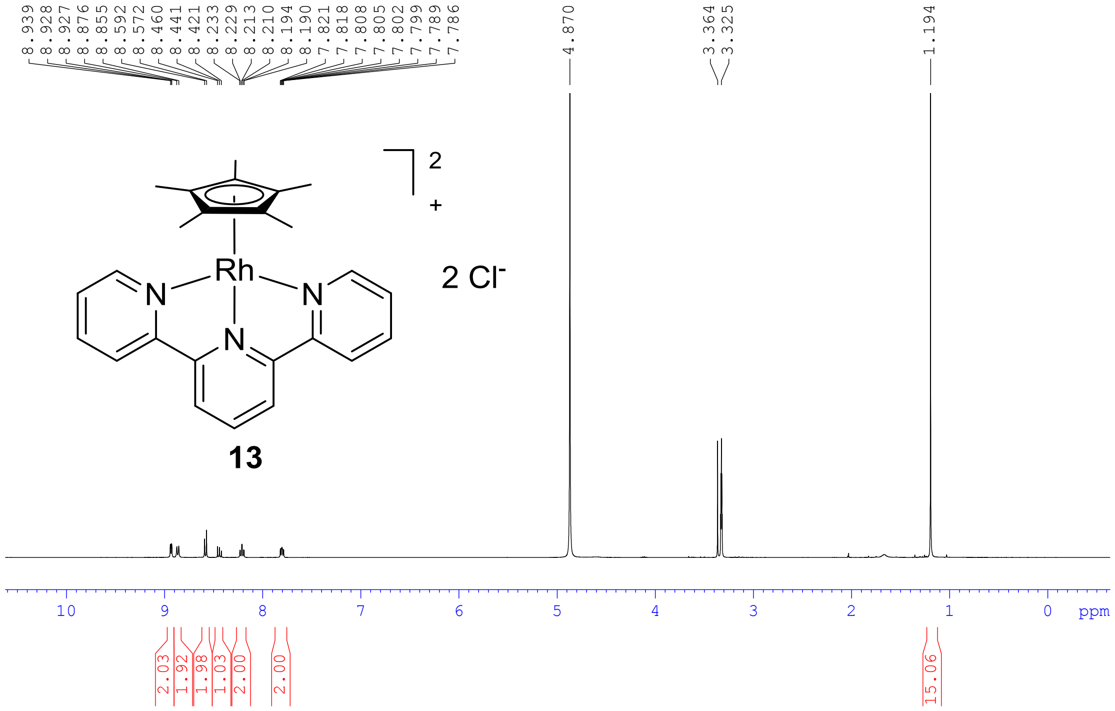
8.939 8.928 8.927 8.876 8.855 8.592 8.572 8.460 8.441 8.421 8.233 8.229 8.213 8.210 8.194 8.190 7.821 7.818 7.808 7.805 7.802 7.799 7.789 7.786
4.870
3.364 3.325
1.194
2+
2 Cl-
Rh
N
13
10 9 8 7 6 5 4 3 2 1 0 ppm
2.03 1.92 1.98 1.03 2.00 2.00
15.06

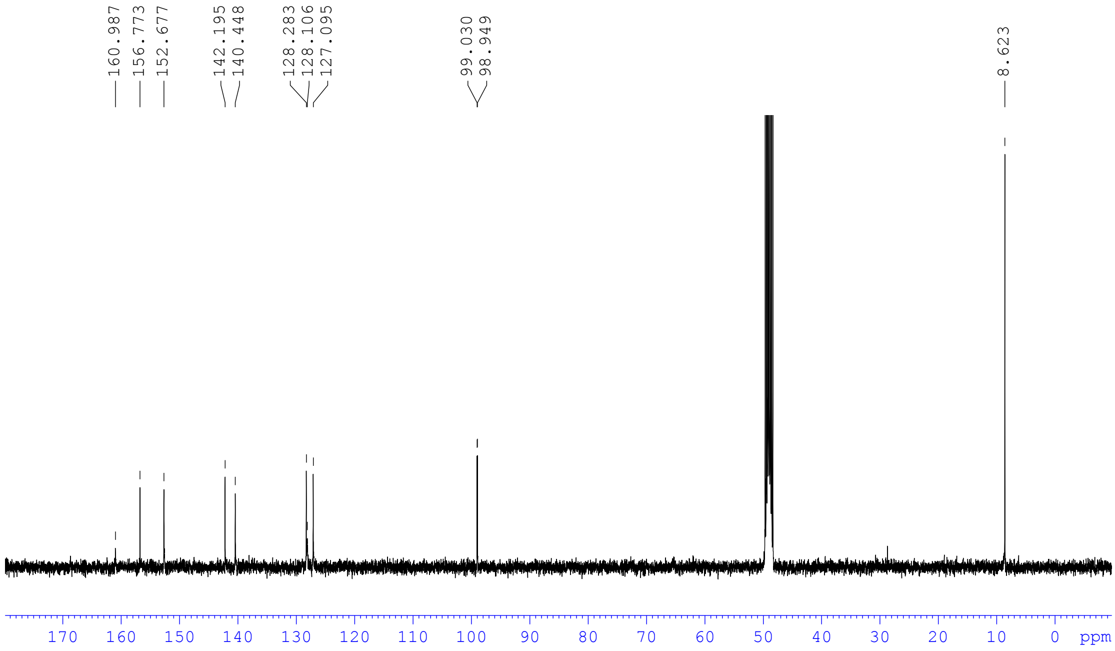
160.987 156.773 152.677
142.195 140.448
128.283 128.106 127.095
99.030 98.949
8.623
170 160 150 140 130 120 110 100 90 80 70 60 50 40 30 20 10 0 ppm

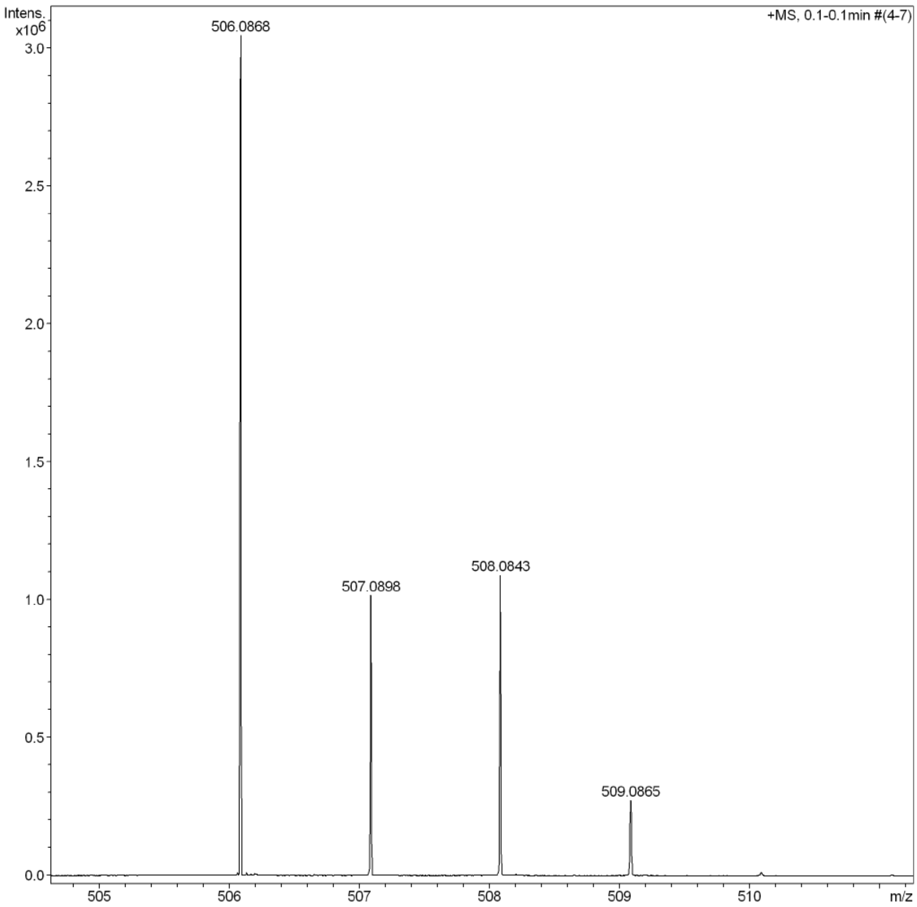
Intens.
x10^6
3.0
2.5
2.0
1.5
1.0
0.5
0.0
+MS, 0.1-0.1min #(4-7)
506.0868
507.0898
508.0843
509.0865
505
506
507
508
509
510
m/z

HRMS of **13**

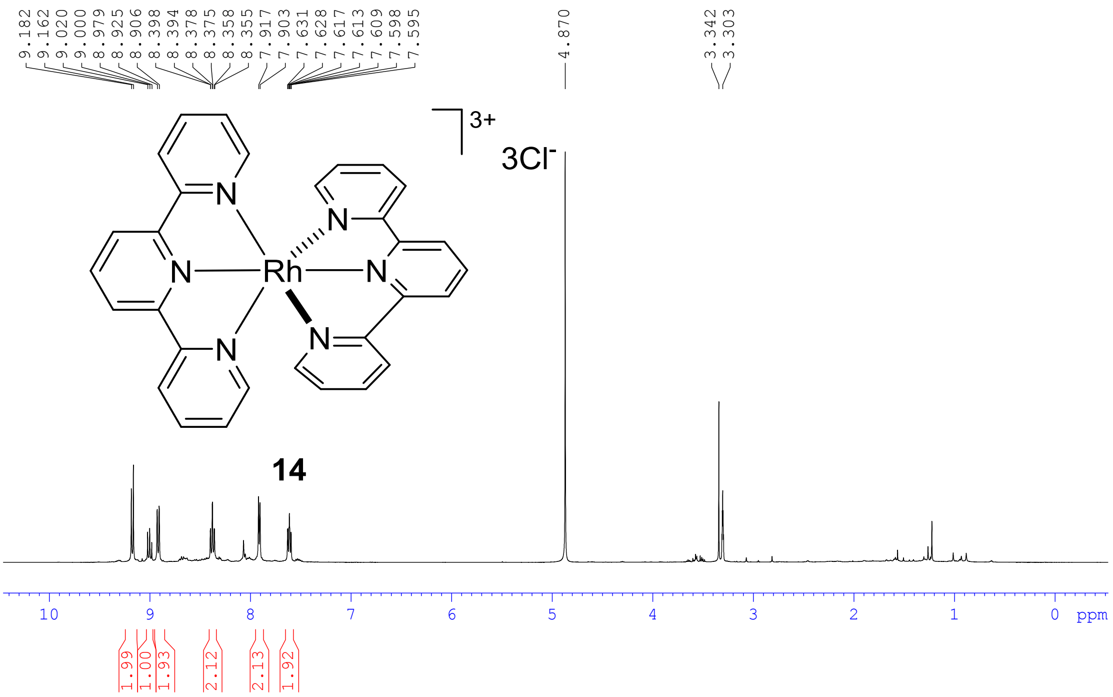
9.182
9.162
9.020
9.000
8.979
8.925
8.906
8.398
8.394
8.378
8.375
8.358
8.355
7.917
7.903
7.631
7.628
7.617
7.613
7.609
7.598
7.595
4.870
3.342
3.303
3+
3Cl-
N
Rh
14
10
9
8
7
6
5
4
3
2
1
0
ppm
1.99
1.00
1.93
2.12
2.13
1.92

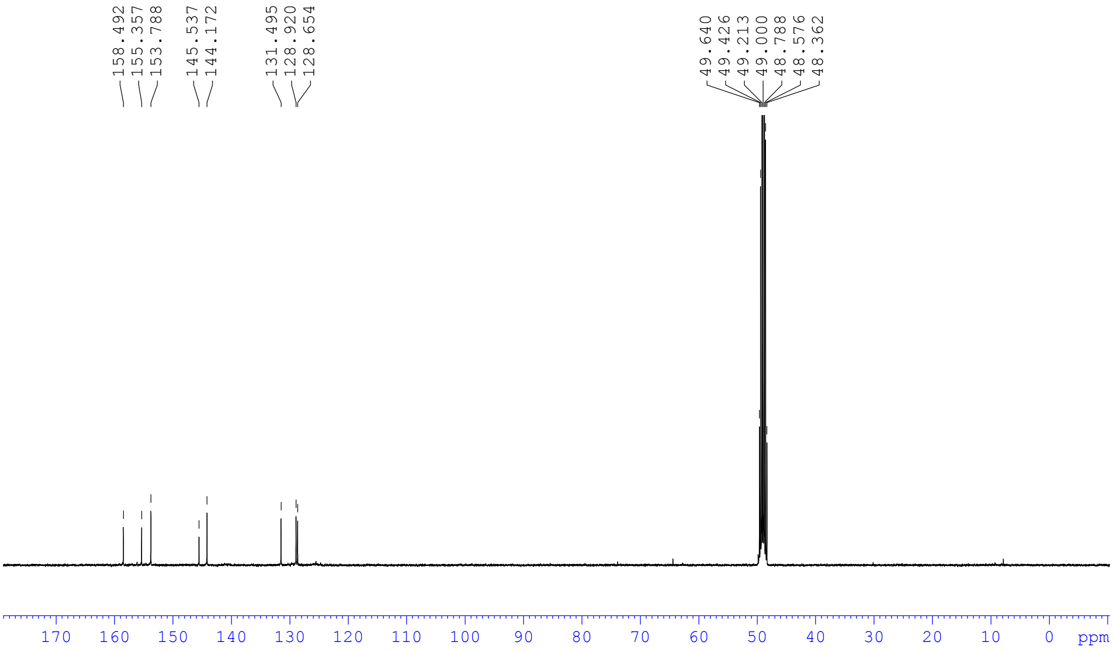
158.492
155.357
153.788
145.537
144.172
131.495
128.920
128.654
49.640
49.426
49.213
49.000
48.788
48.576
48.362
170
160
150
140
130
120
110
100
90
80
70
60
50
40
30
20
10
0
ppm

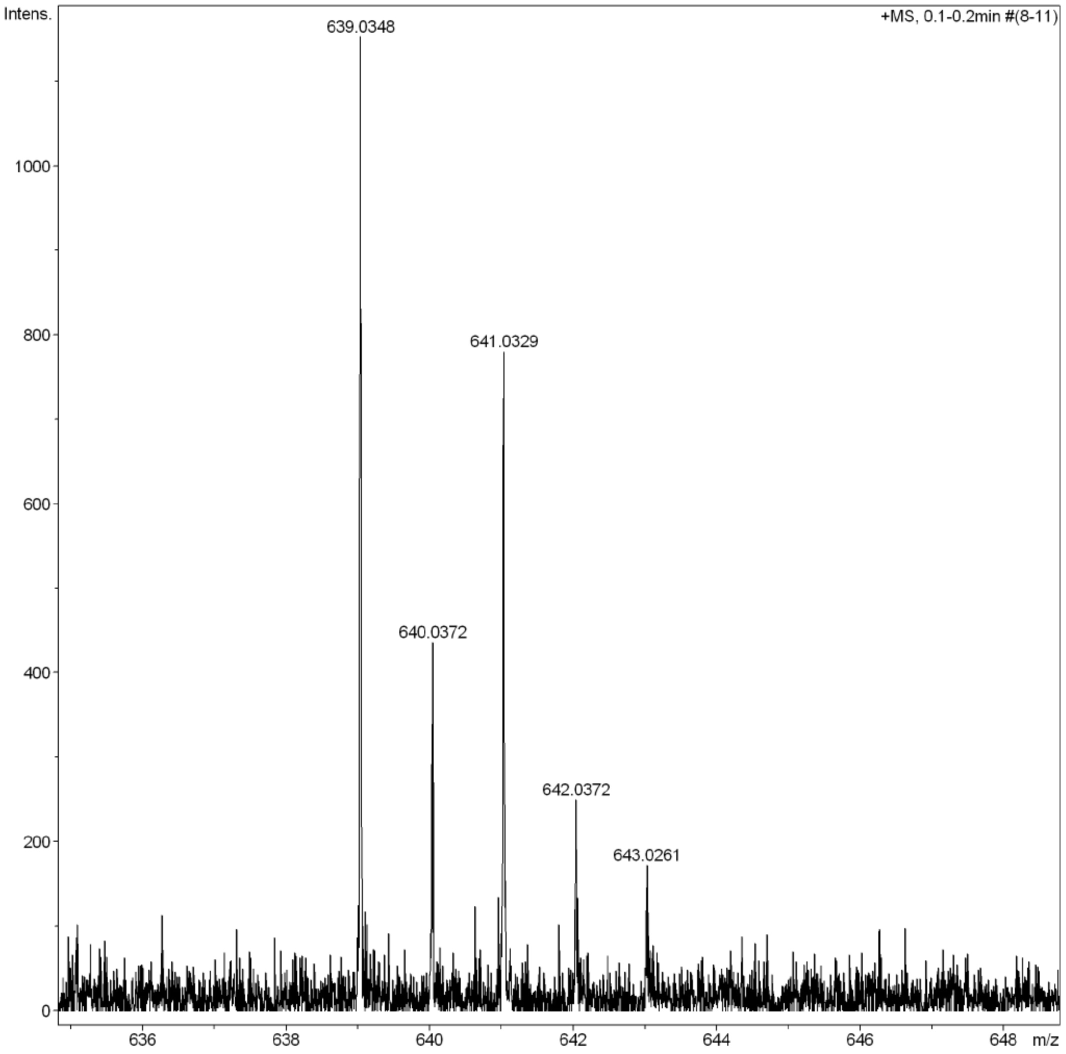
Intens.
+MS, 0.1-0.2min #(8-11)
639.0348
641.0329
640.0372
642.0372
643.0261
1000
800
600
400
200
0
636
638
640
642
644
646
648
m/z

HRMS of **14**

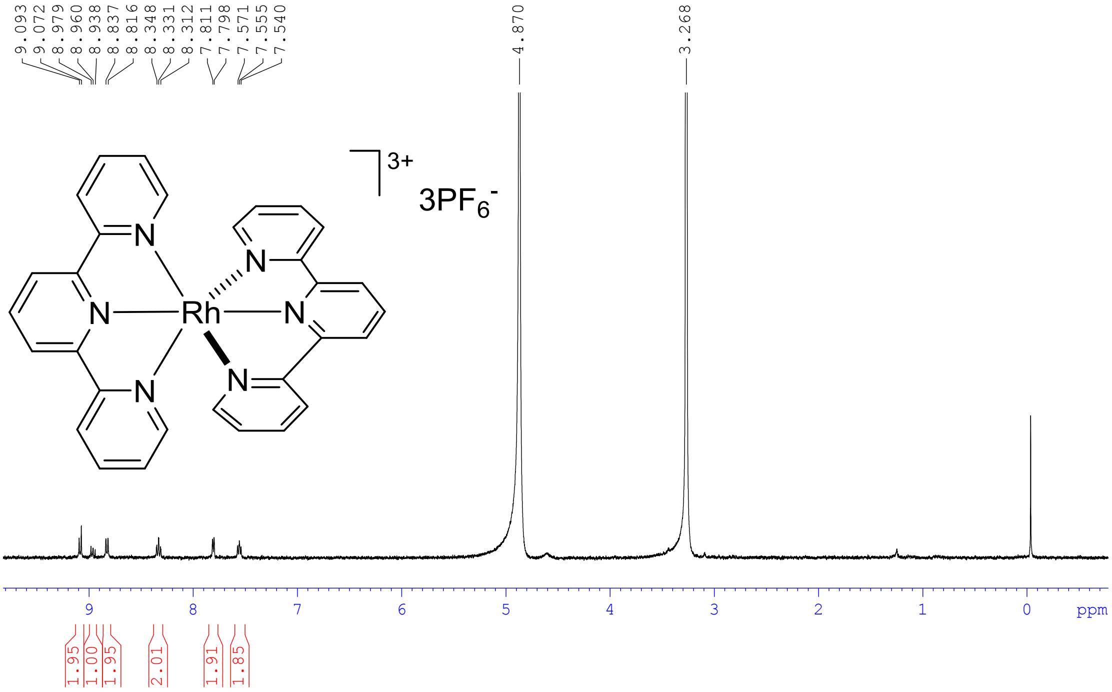

9.093
9.072
8.979
8.960
8.938
8.837
8.816
8.348
8.331
8.312
7.811
7.798
7.571
7.555
7.540
4.870
3.268
3+
$3PF_6^-$
Rh
N
9
8
7
6
5
4
3
2
1
0
ppm
1.95
1.00
1.95
2.01
1.91
1.85

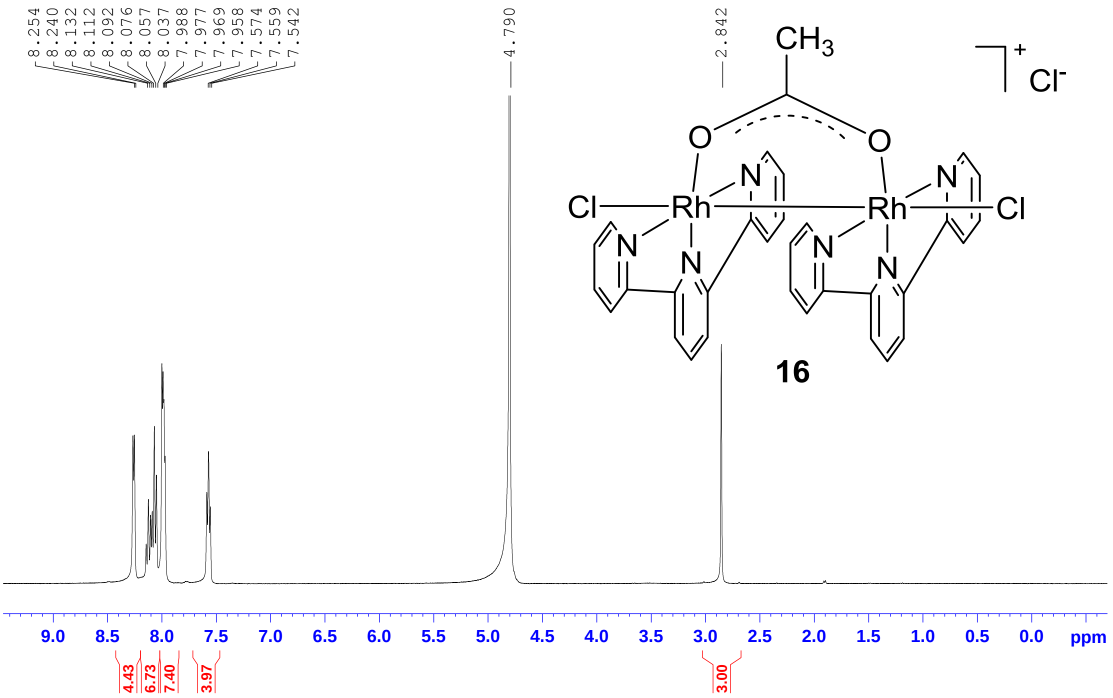
8.254
8.240
8.132
8.112
8.092
8.076
8.057
8.037
7.988
7.977
7.969
7.958
7.574
7.559
7.542
4.790
2.842
CH3
Cl-
O
O
Cl
Rh
Rh
Cl
N
N
N
16
9.0 8.5 8.0 7.5 7.0 6.5 6.0 5.5 5.0 4.5 4.0 3.5 3.0 2.5 2.0 1.5 1.0 0.5 0.0 ppm
4.43
6.73
7.40
3.97
3.00

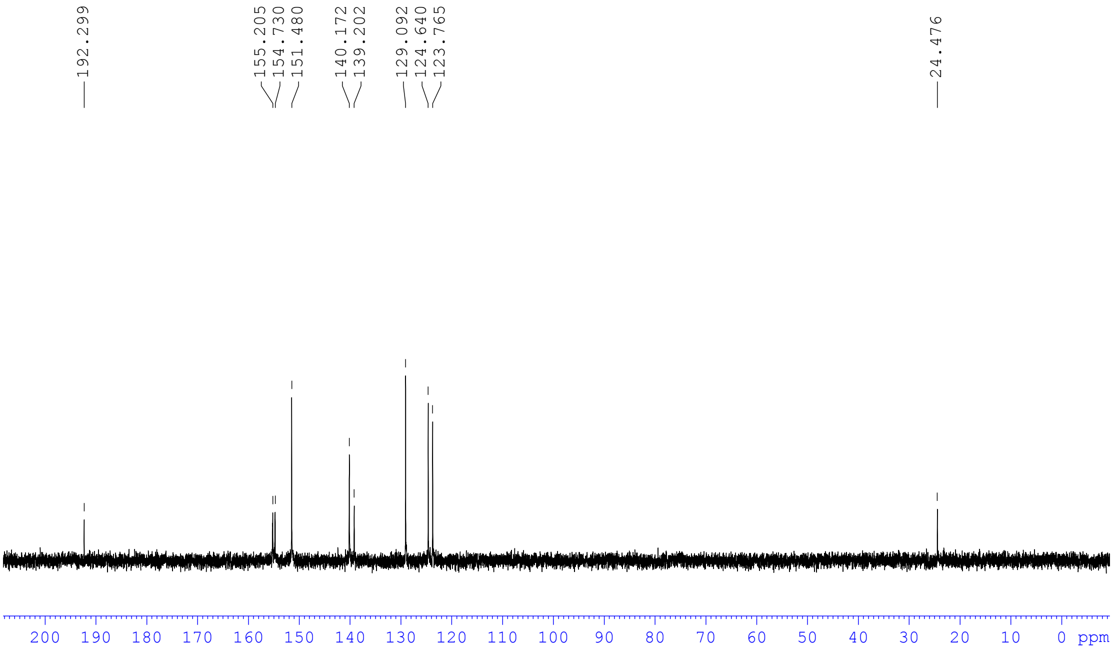
192.299
155.205
154.730
151.480
140.172
139.202
129.092
124.640
123.765
24.476
200 190 180 170 160 150 140 130 120 110 100 90 80 70 60 50 40 30 20 10 0 ppm

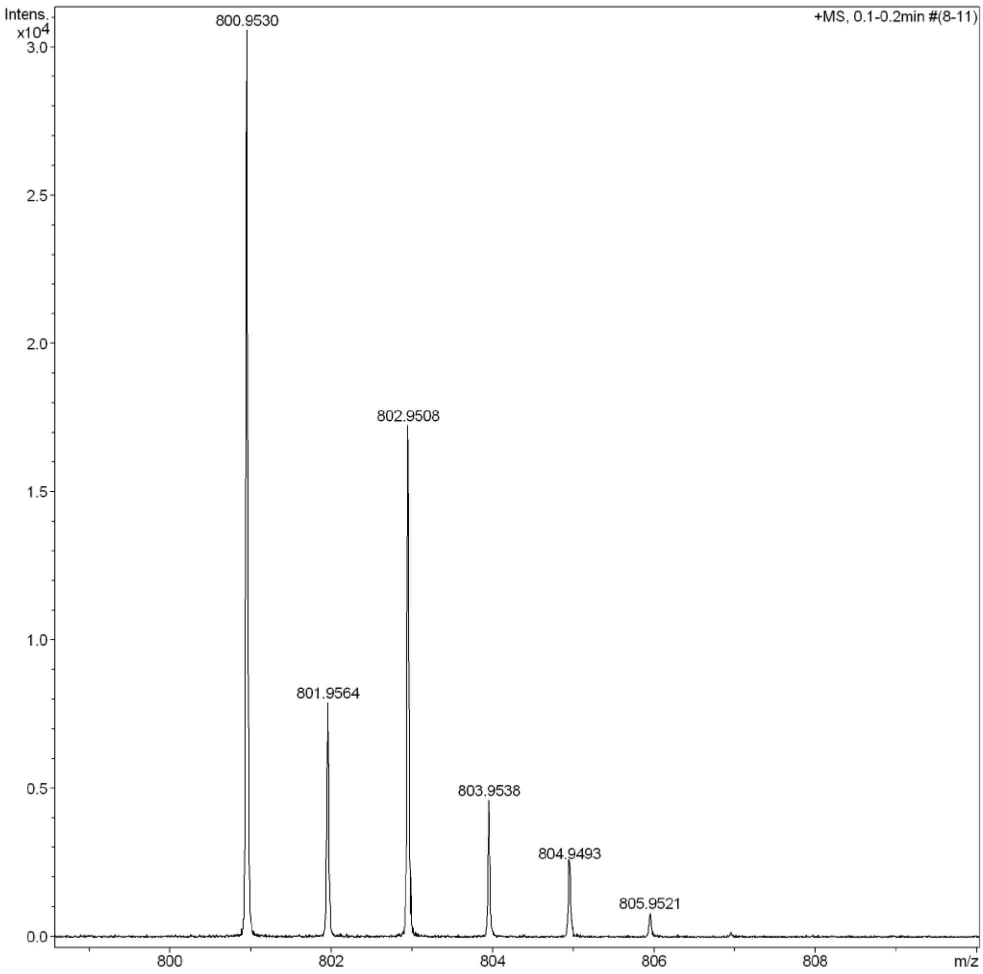

HRMS of **16**

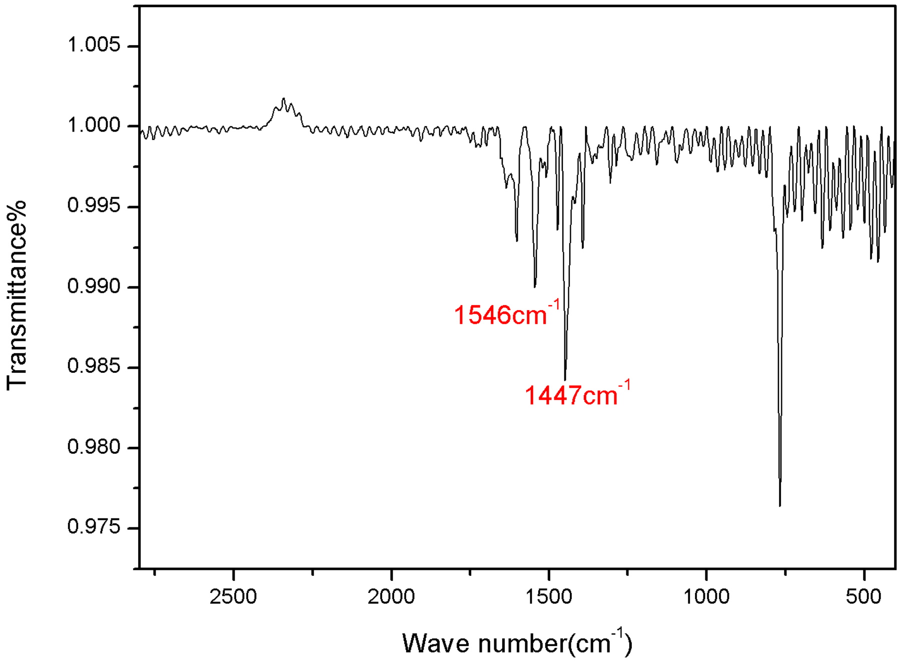

IR of **16**

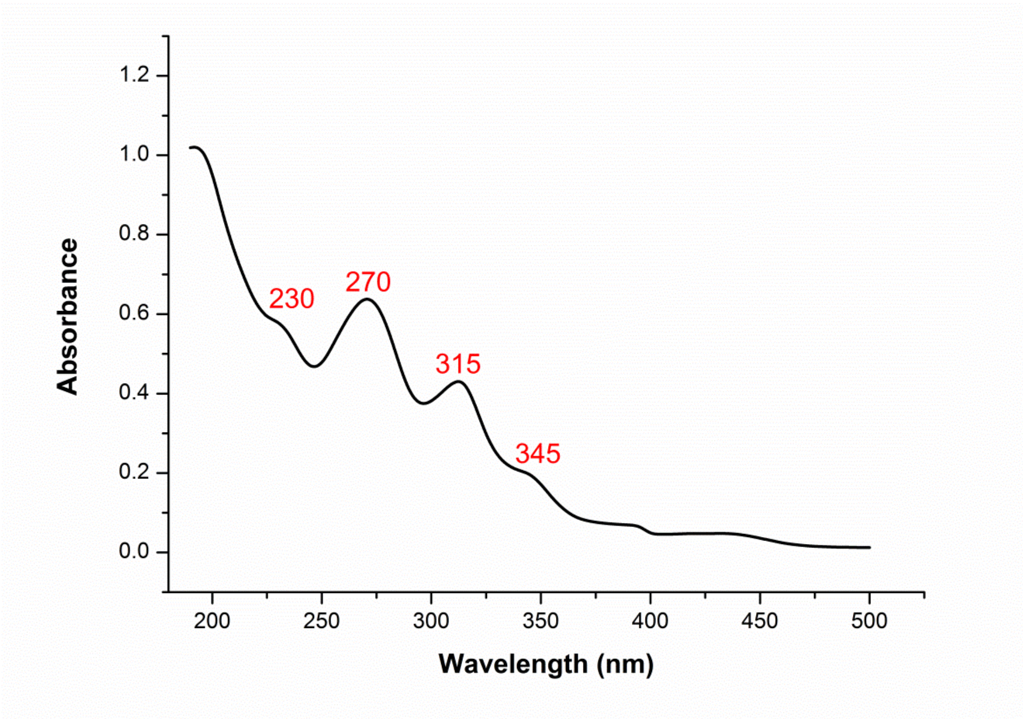

Absorbance
Wavelength (nm)
230
270
315
345
1.2
1.0
0.8
0.6
0.4
0.2
0.0
200
250
300
350
400
450
500

UV of **16**

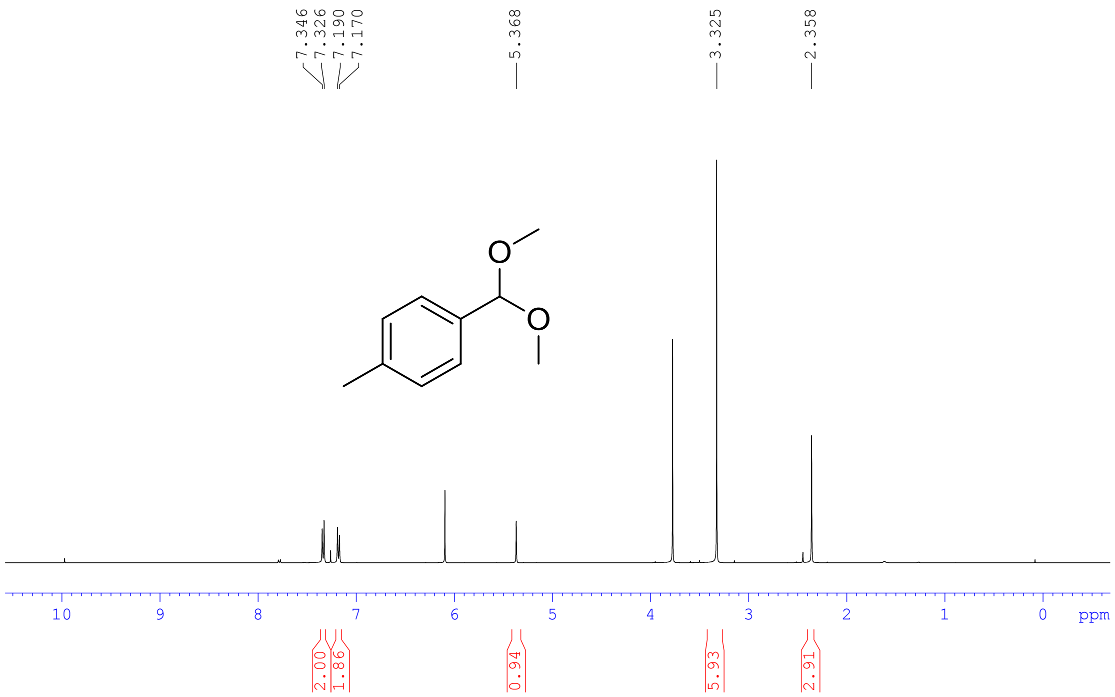

7.346
7.326
7.190
7.170
5.368
3.325
2.358
O
O
ppm
2.00
1.86
0.94
5.93
2.91

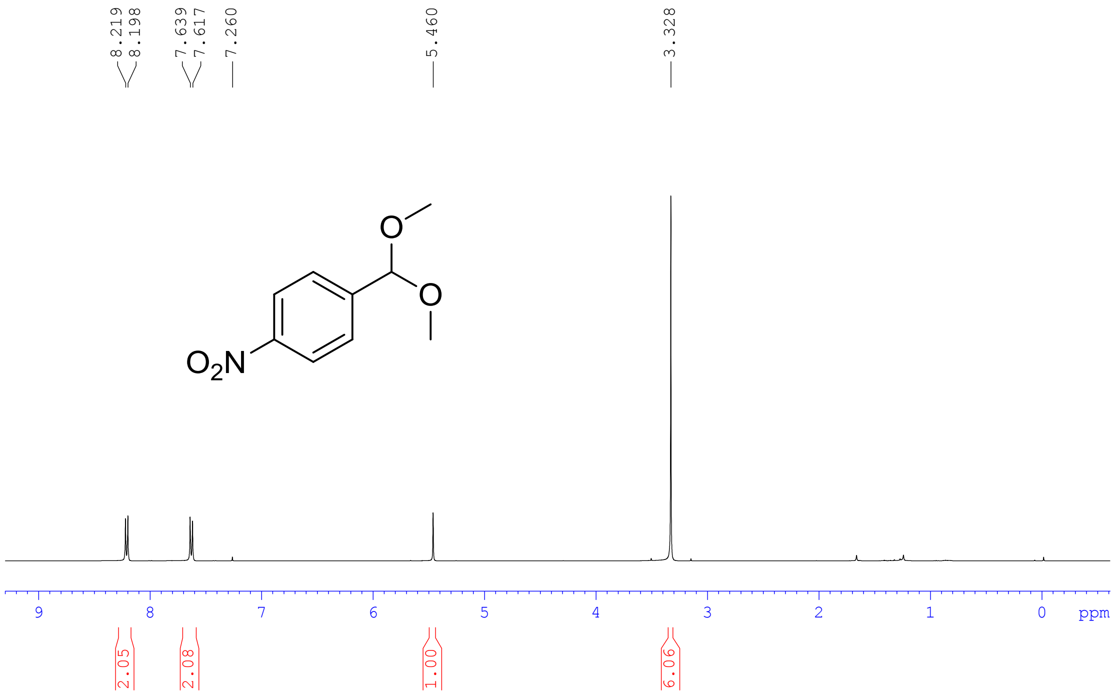

8.219
8.198
7.639
7.617
7.260
5.460
3.328
O
O
O_2N
ppm
2.05
2.08
1.00
6.06

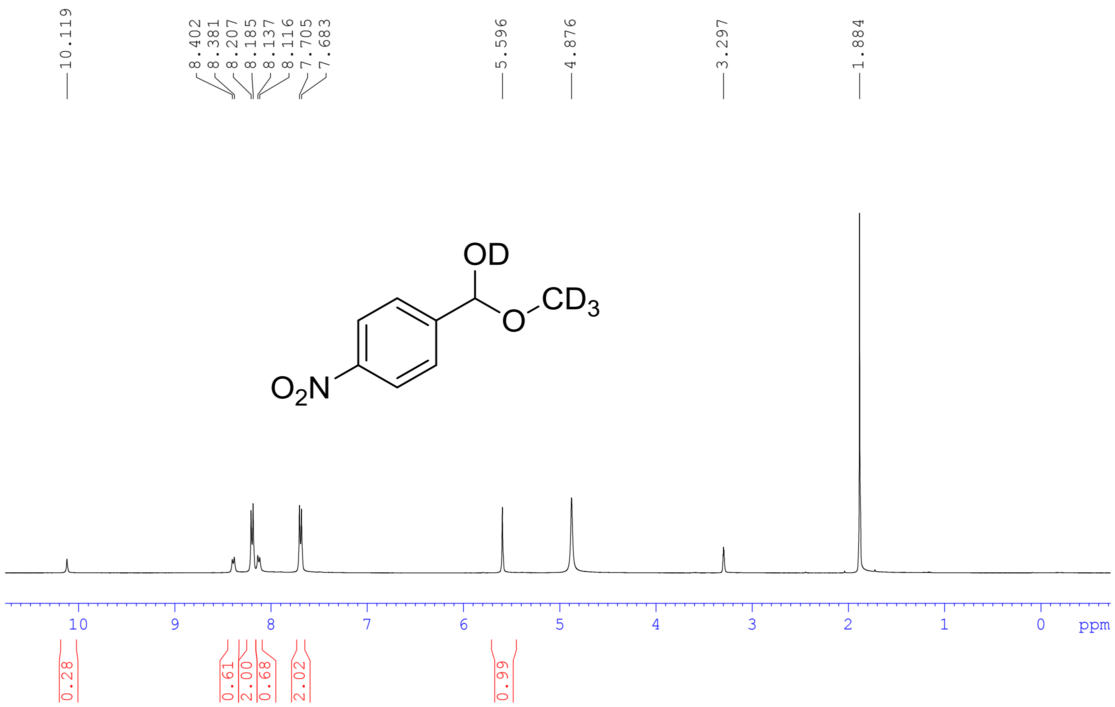
10.119
8.402
8.381
8.207
8.185
8.137
8.116
7.705
7.683
5.596
4.876
3.297
1.884
OD
O
CD3
O2N
10
9
8
7
6
5
4
3
2
1
0
ppm
0.28
0.61
2.00
0.68
2.02
0.99

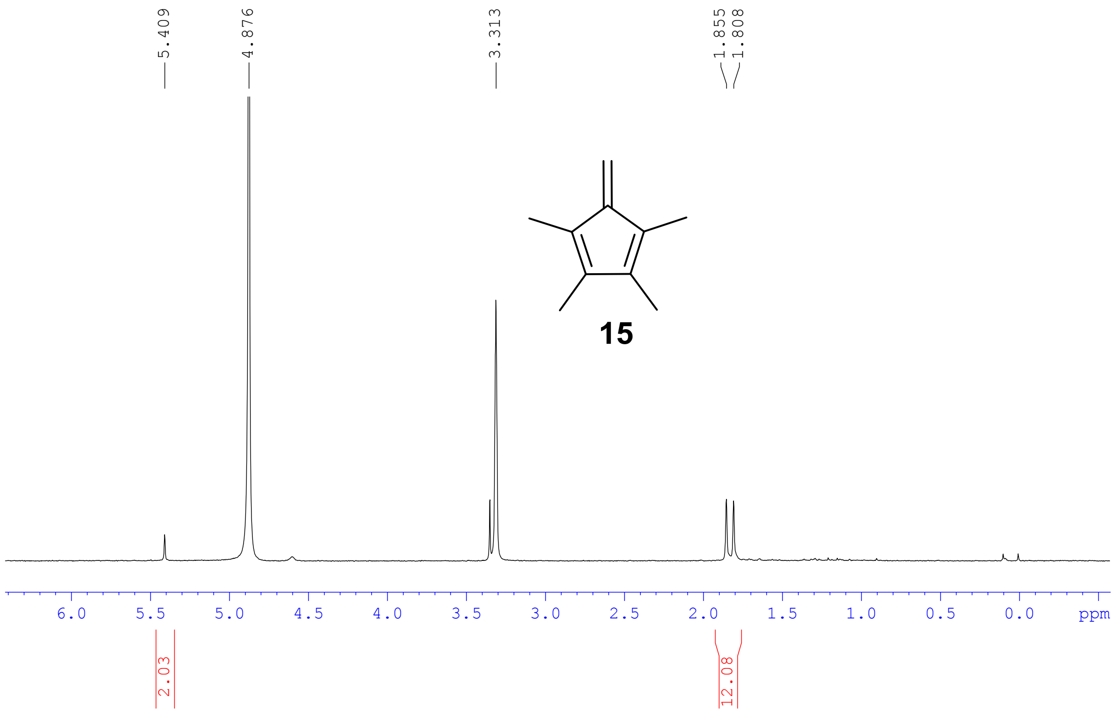
5.409
4.876
3.313
1.855
1.808
15
6.0
5.5
5.0
4.5
4.0
3.5
3.0
2.5
2.0
1.5
1.0
0.5
0.0
ppm
2.03
12.08

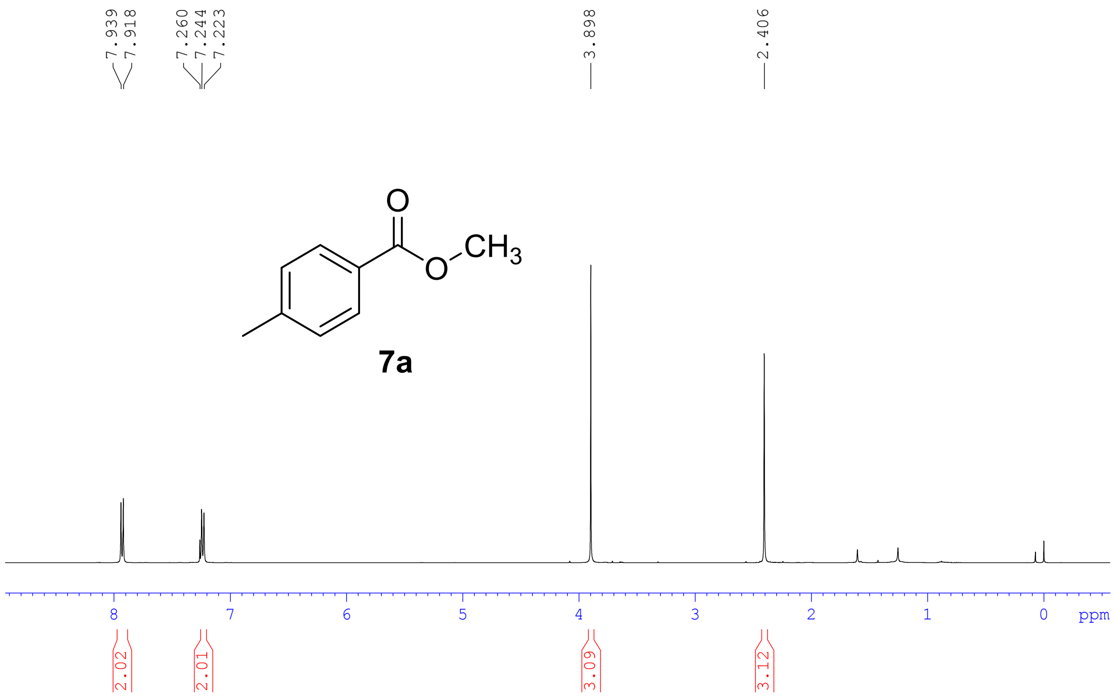
7.939
7.918
7.260
7.244
7.223
3.898
2.406
O
O
CH3
7a
8
7
6
5
4
3
2
1
0
ppm
2.02
2.01
3.09
3.12

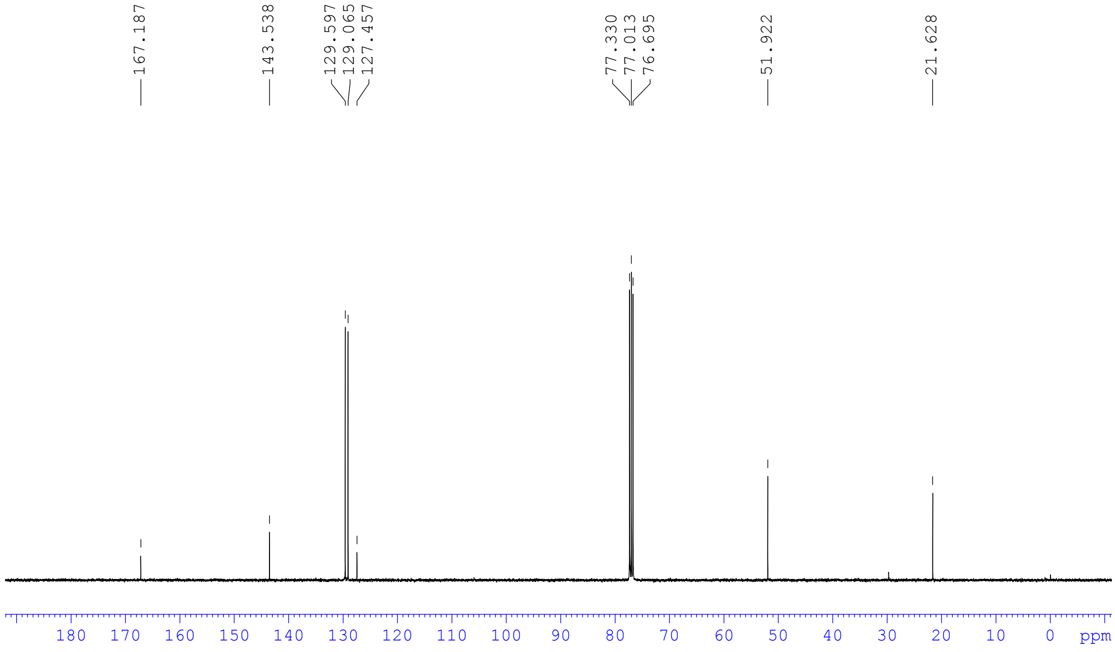
167.187
143.538
129.597
129.065
127.457
77.330
77.013
76.695
51.922
21.628
180
170
160
150
140
130
120
110
100
90
80
70
60
50
40
30
20
10
0
ppm

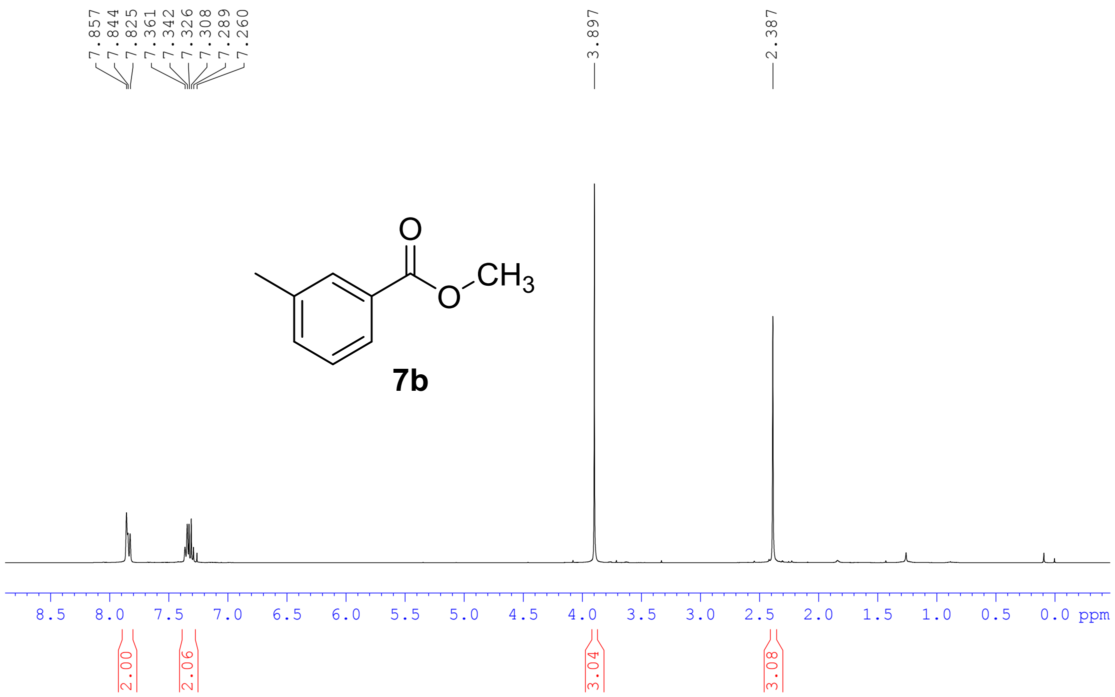
7.857
7.844
7.825
7.361
7.342
7.326
7.308
7.289
7.260
3.897
2.387
O
O
CH3
7b
8.5 8.0 7.5 7.0 6.5 6.0 5.5 5.0 4.5 4.0 3.5 3.0 2.5 2.0 1.5 1.0 0.5 0.0 ppm
2.00
2.06
3.04
3.08

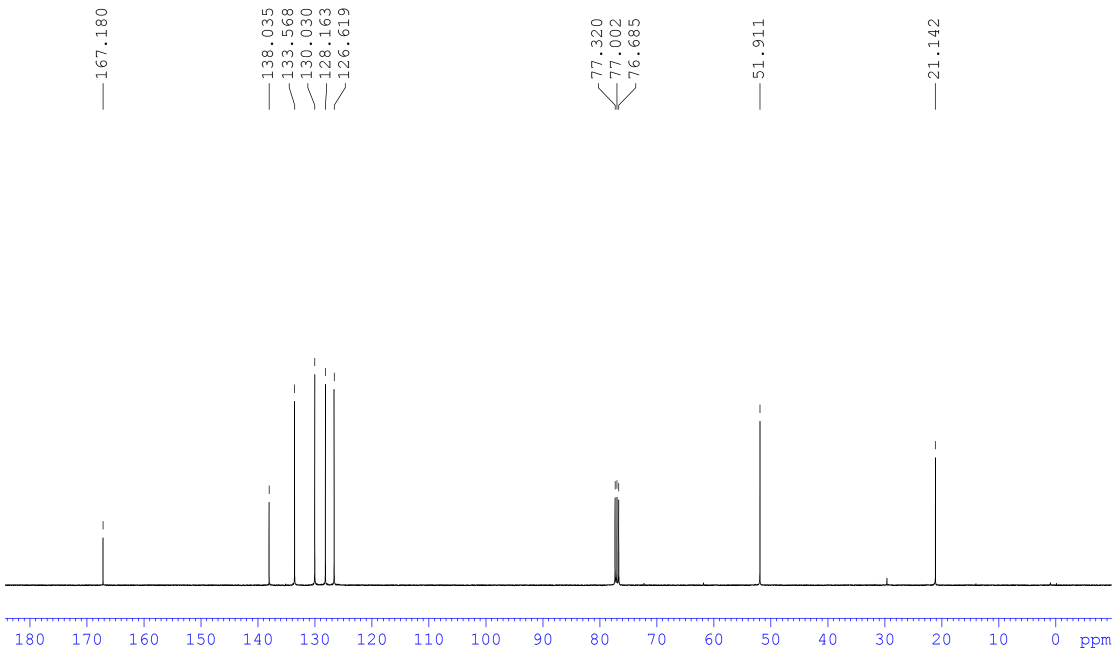
167.180
138.035
133.568
130.030
128.163
126.619
77.320
77.002
76.685
51.911
21.142
180 170 160 150 140 130 120 110 100 90 80 70 60 50 40 30 20 10 0 ppm

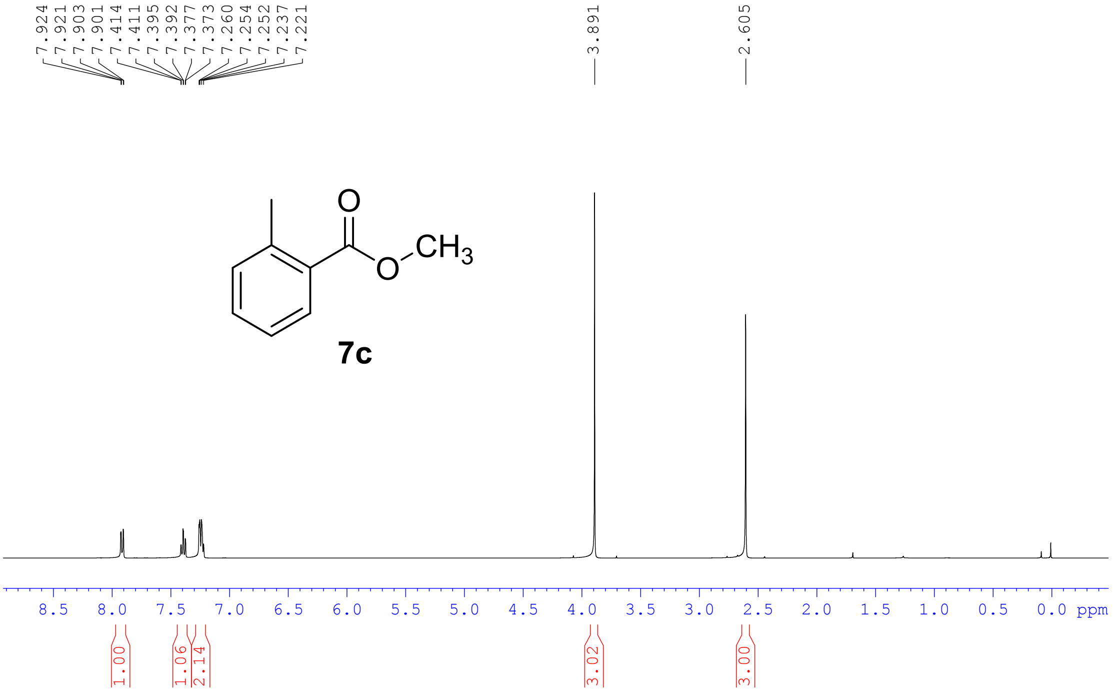
O
O
CH3
7c
7.924
7.921
7.903
7.901
7.414
7.411
7.395
7.392
7.377
7.373
7.260
7.254
7.252
7.237
7.221
3.891
2.605
8.5 8.0 7.5 7.0 6.5 6.0 5.5 5.0 4.5 4.0 3.5 3.0 2.5 2.0 1.5 1.0 0.5 0.0 ppm
1.00
1.06
2.14
3.02
3.00

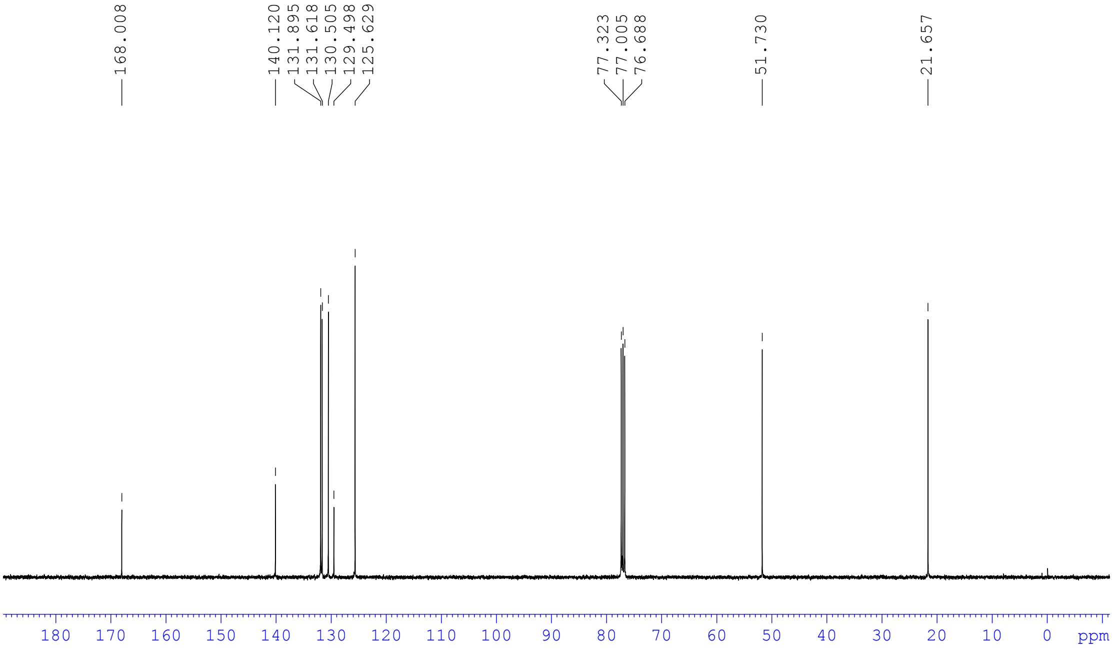
168.008
140.120
131.895
131.618
130.505
129.498
125.629
77.323
77.005
76.688
51.730
21.657
180 170 160 150 140 130 120 110 100 90 80 70 60 50 40 30 20 10 0 ppm

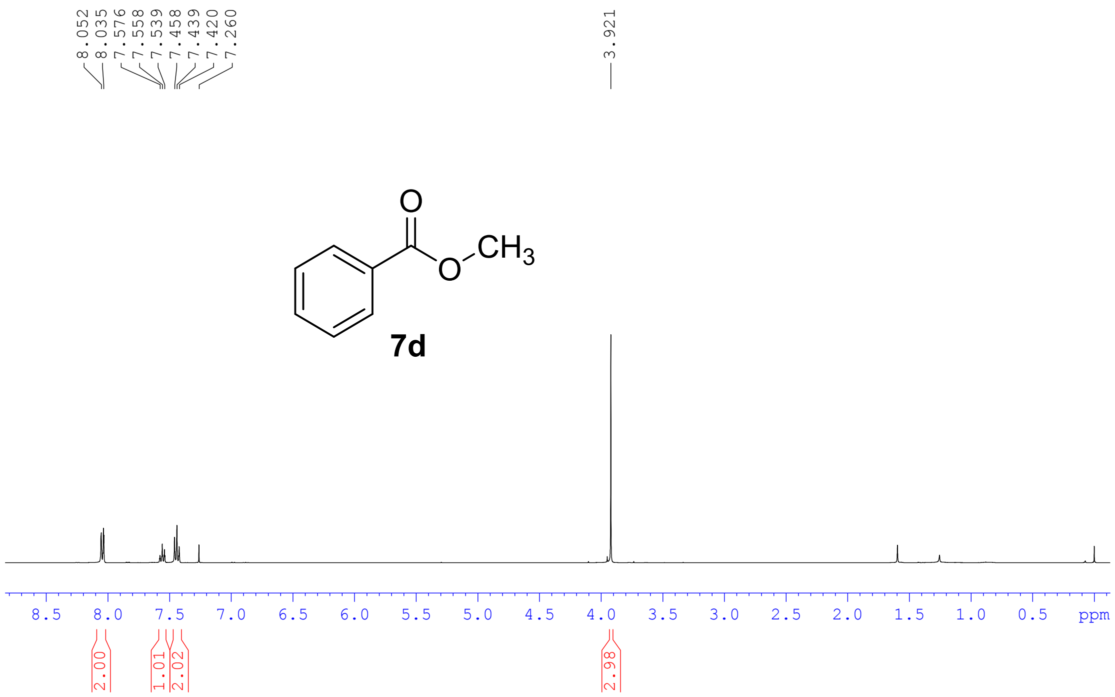
8.052
8.035
7.576
7.558
7.539
7.458
7.439
7.420
7.260
3.921
O
O
CH3
7d
8.5 8.0 7.5 7.0 6.5 6.0 5.5 5.0 4.5 4.0 3.5 3.0 2.5 2.0 1.5 1.0 0.5 ppm
2.00
1.01
2.02
2.98

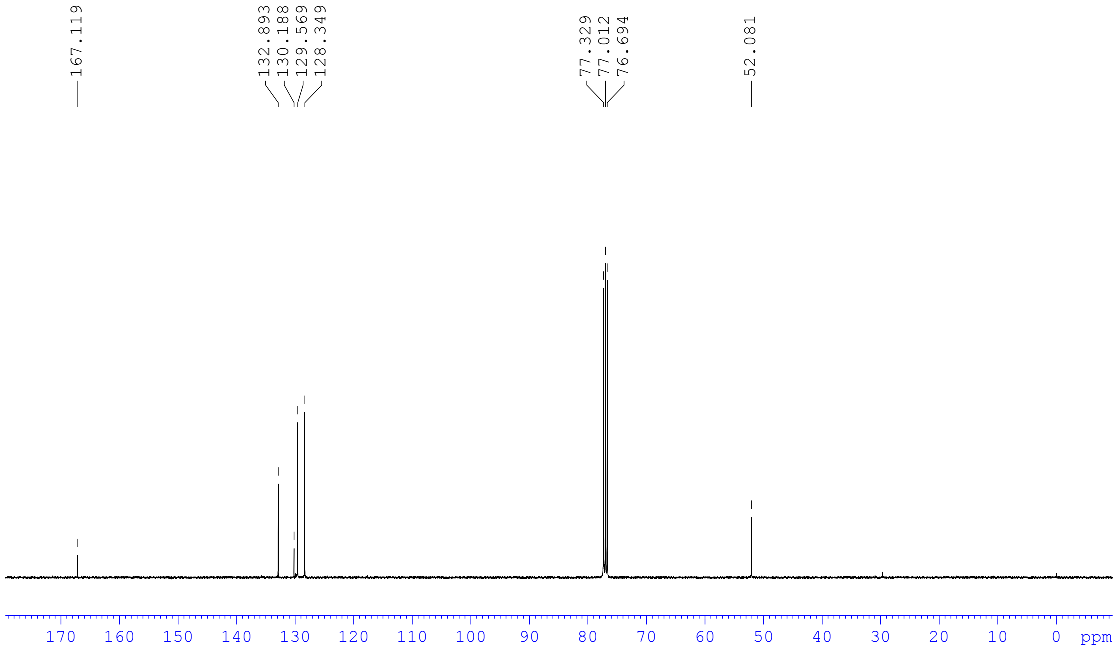
167.119
132.893
130.188
129.569
128.349
77.329
77.012
76.694
52.081
170 160 150 140 130 120 110 100 90 80 70 60 50 40 30 20 10 0 ppm

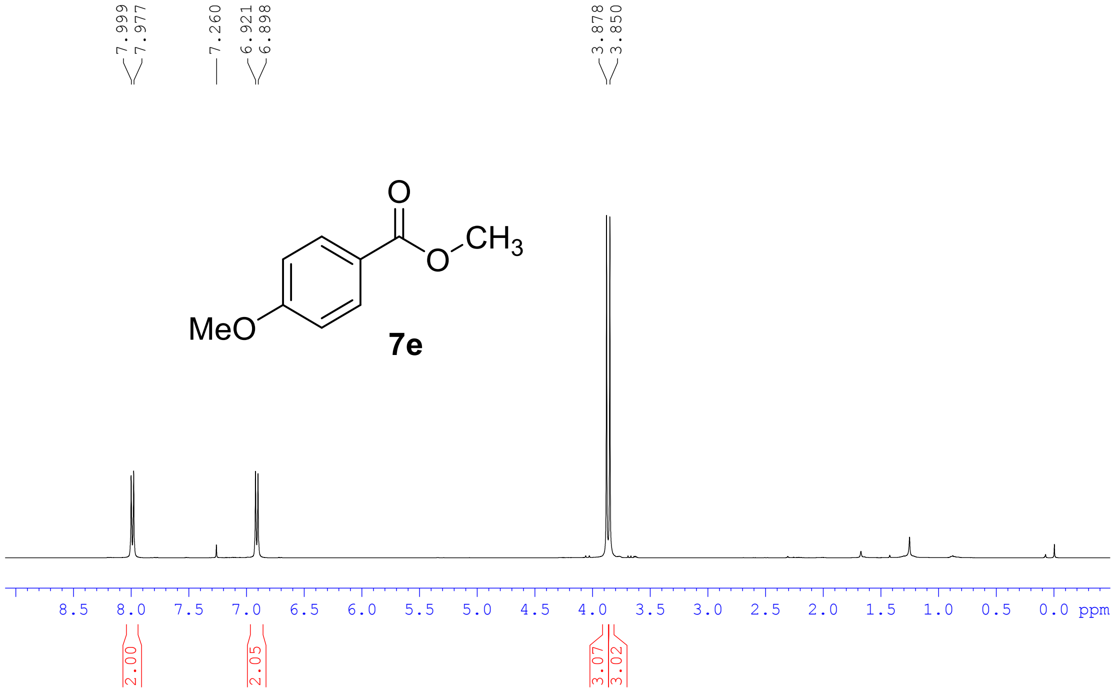
7.999
7.977
7.260
6.921
6.898
3.878
3.850
O
CH3
MeO
7e
8.5 8.0 7.5 7.0 6.5 6.0 5.5 5.0 4.5 4.0 3.5 3.0 2.5 2.0 1.5 1.0 0.5 0.0 ppm
2.00
2.05
3.07
3.02

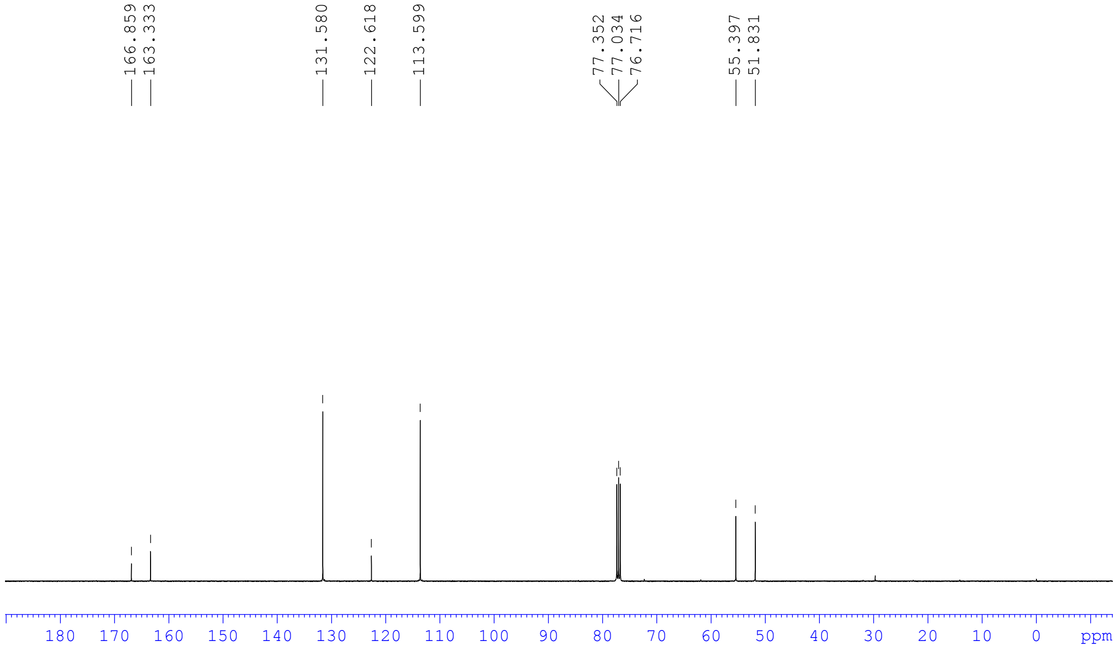
166.859
163.333
131.580
122.618
113.599
77.352
77.034
76.716
55.397
51.831
180 170 160 150 140 130 120 110 100 90 80 70 60 50 40 30 20 10 0 ppm

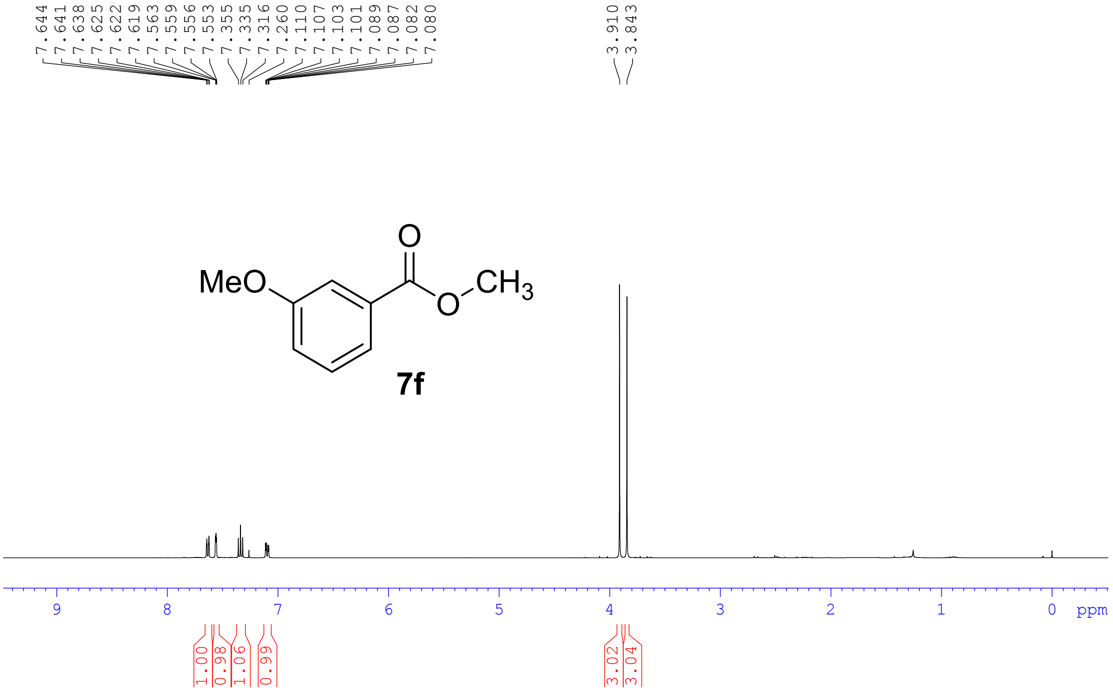
7.644
7.641
7.638
7.625
7.622
7.619
7.563
7.559
7.556
7.553
7.355
7.335
7.316
7.260
7.110
7.107
7.103
7.101
7.089
7.087
7.082
7.080
3.910
3.843
MeO
O
O
CH3
7f
1.00
0.98
1.06
0.99
3.02
3.04
ppm

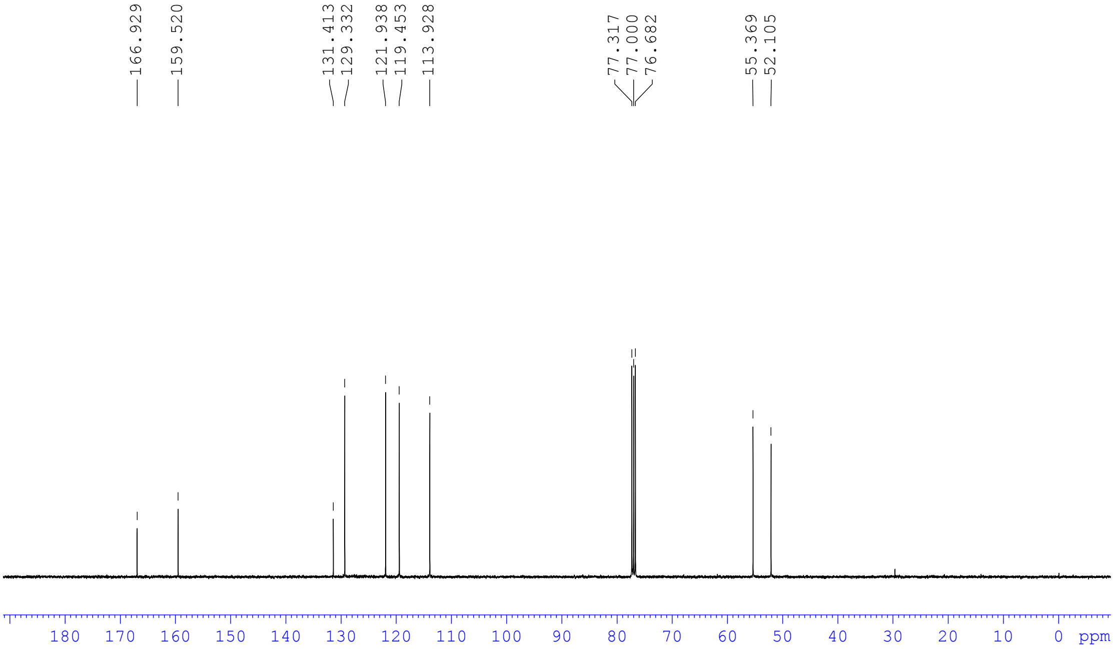
166.929
159.520
131.413
129.332
121.938
119.453
113.928
77.317
77.000
76.682
55.369
52.105
ppm

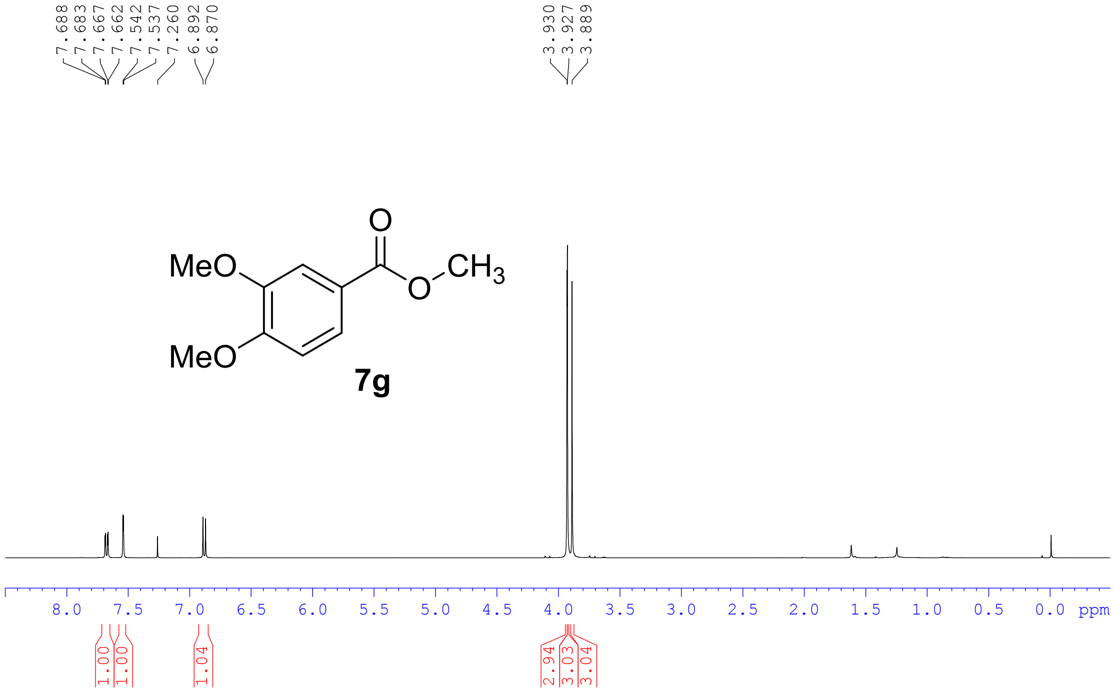
7.688
7.683
7.667
7.662
7.542
7.537
7.260
6.892
6.870
3.930
3.927
3.889
MeO
MeO
O
O
CH3
7g
8.0 7.5 7.0 6.5 6.0 5.5 5.0 4.5 4.0 3.5 3.0 2.5 2.0 1.5 1.0 0.5 0.0 ppm
1.00
1.00
1.04
2.94
3.03
3.04

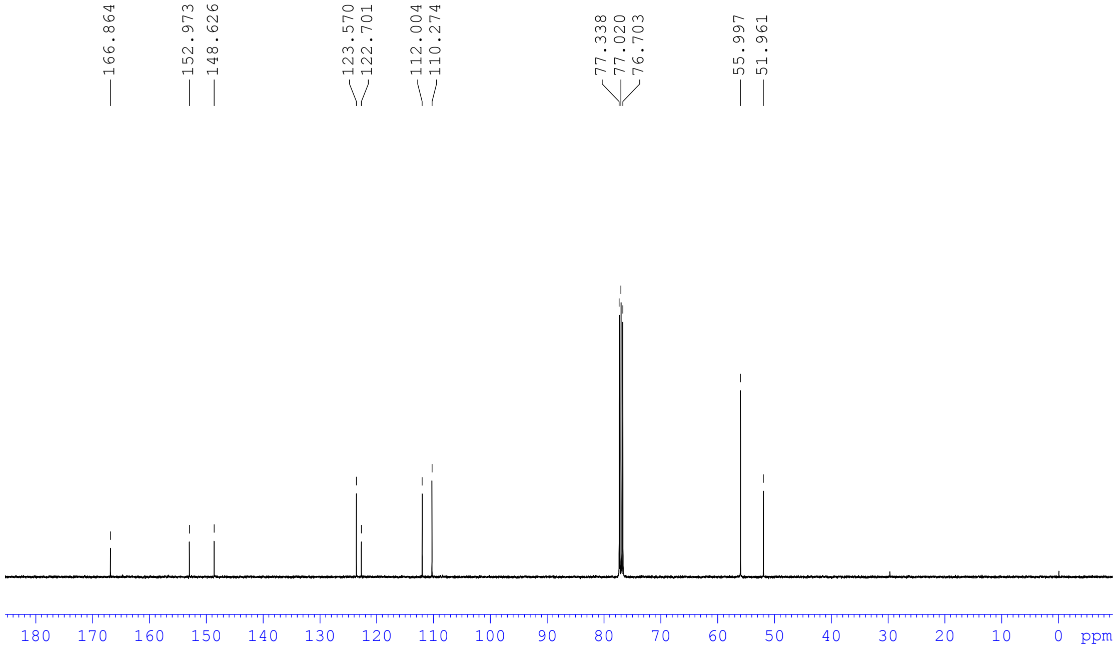
166.864
152.973
148.626
123.570
122.701
112.004
110.274
77.338
77.020
76.703
55.997
51.961
180 170 160 150 140 130 120 110 100 90 80 70 60 50 40 30 20 10 0 ppm

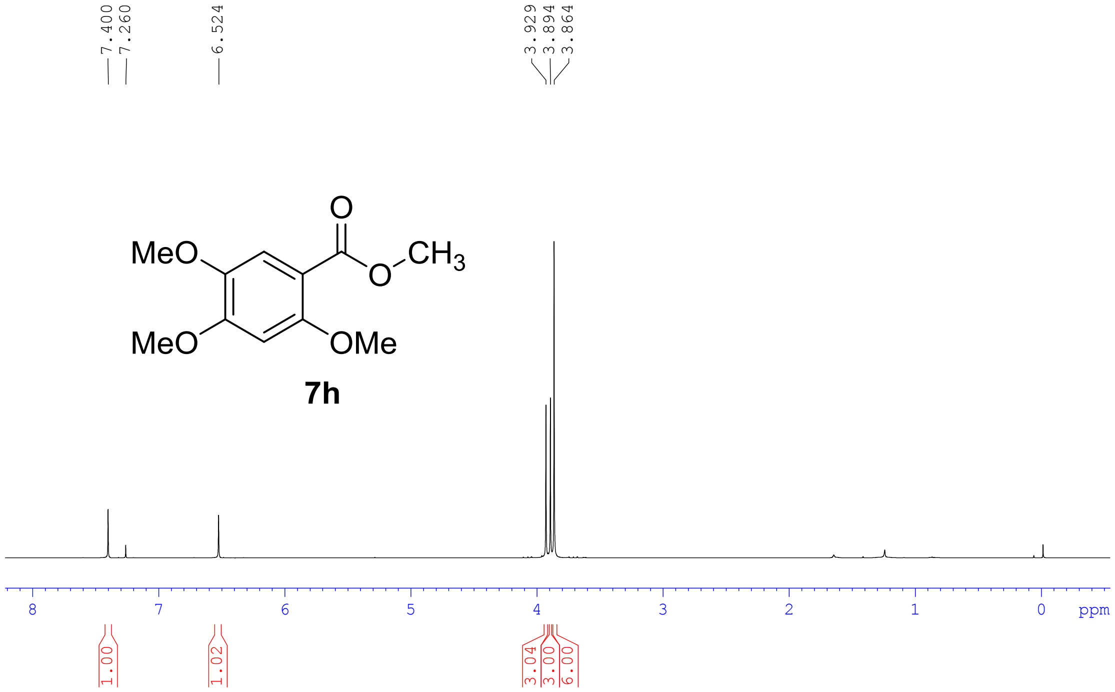
7.400
7.260
6.524
3.929
3.894
3.864
MeO
MeO
O
CH3
OMe
7h
8
7
6
5
4
3
2
1
0
ppm
1.00
1.02
3.04
3.00
6.00

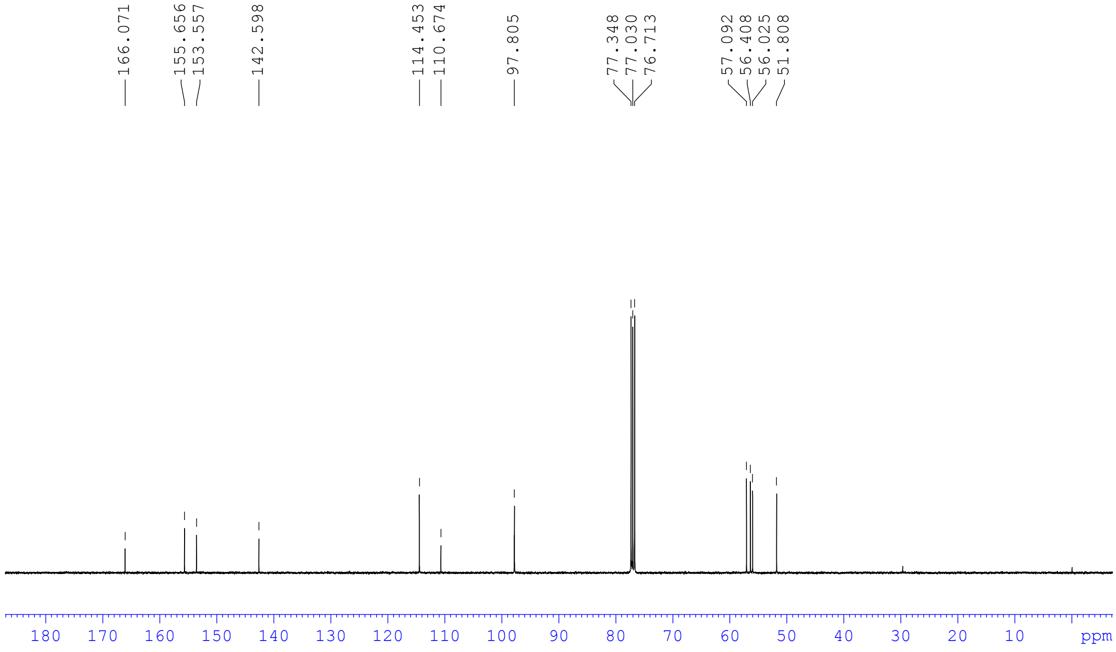
166.071
155.656
153.557
142.598
114.453
110.674
97.805
77.348
77.030
76.713
57.092
56.408
56.025
51.808
180
170
160
150
140
130
120
110
100
90
80
70
60
50
40
30
20
10
ppm

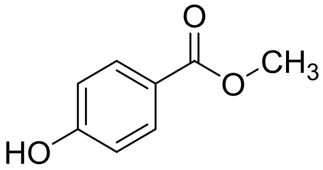
O
CH3
O
HO

7i

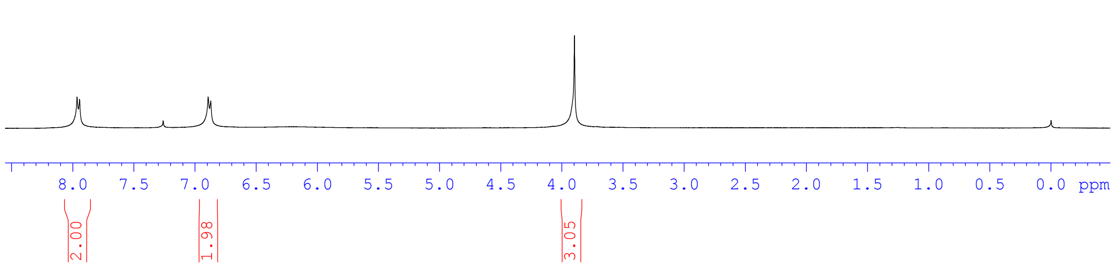
7.963
7.942
7.260
6.892
6.871
3.897
8.0 7.5 7.0 6.5 6.0 5.5 5.0 4.5 4.0 3.5 3.0 2.5 2.0 1.5 1.0 0.5 0.0 ppm
2.00
1.98
3.05

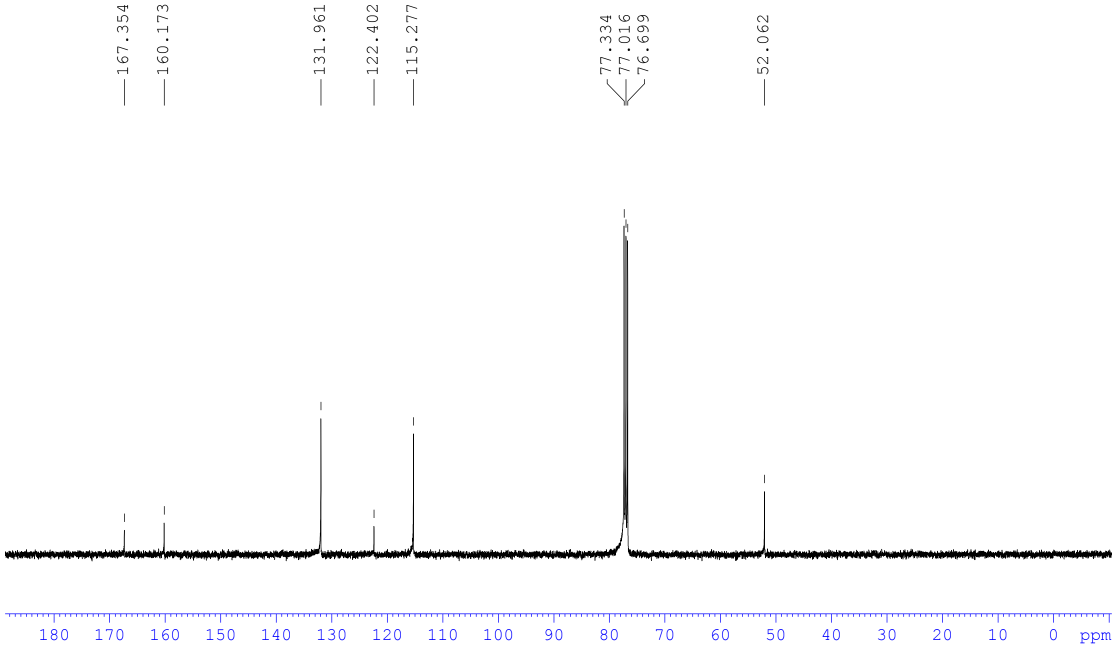
167.354
160.173
131.961
122.402
115.277
77.334
77.016
76.699
52.062
180 170 160 150 140 130 120 110 100 90 80 70 60 50 40 30 20 10 0 ppm

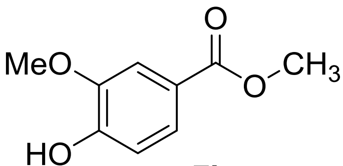
MeO
HO
O
O
CH3

7j

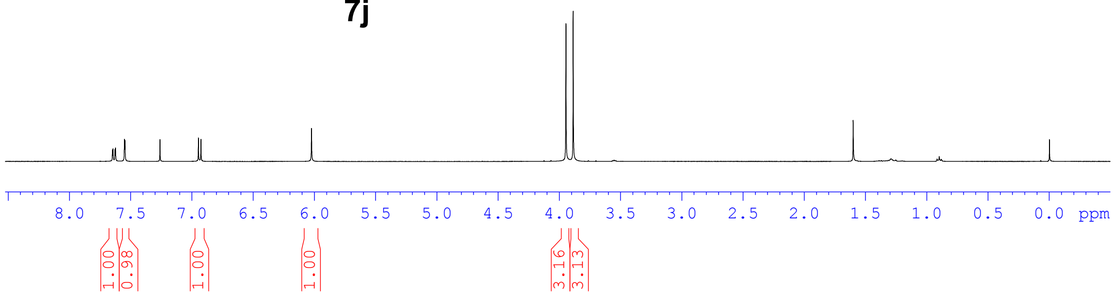
7.648
7.644
7.628
7.623
7.550
7.546
7.260
6.946
6.926
6.023
3.946
3.886
8.0 7.5 7.0 6.5 6.0 5.5 5.0 4.5 4.0 3.5 3.0 2.5 2.0 1.5 1.0 0.5 0.0 ppm
1.00
0.98
1.00
1.00
3.16
3.13

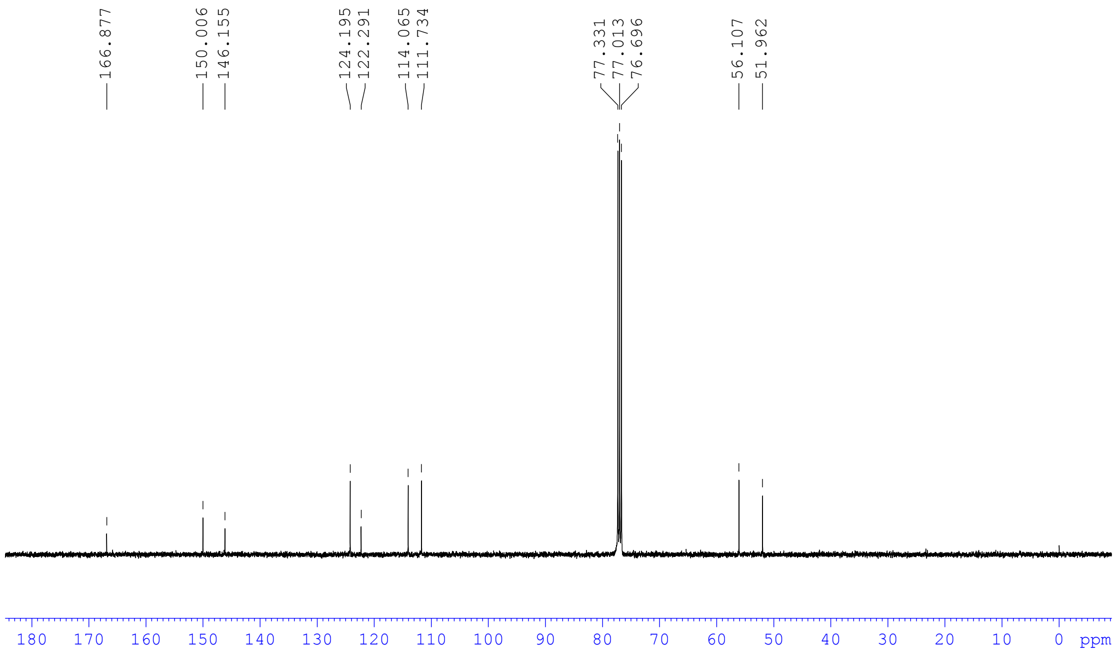
166.877
150.006
146.155
124.195
122.291
114.065
111.734
77.331
77.013
76.696
56.107
51.962
180 170 160 150 140 130 120 110 100 90 80 70 60 50 40 30 20 10 0 ppm

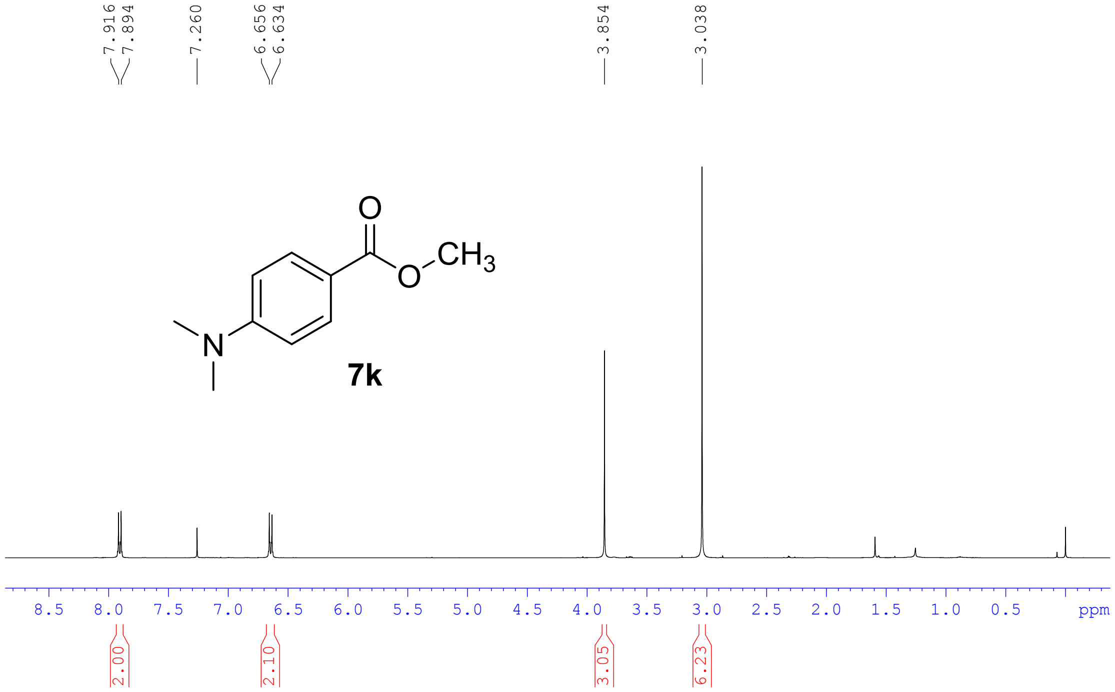
7.916
7.894
7.260
6.656
6.634
3.854
3.038
7k
8.5 8.0 7.5 7.0 6.5 6.0 5.5 5.0 4.5 4.0 3.5 3.0 2.5 2.0 1.5 1.0 0.5 ppm
2.00
2.10
3.05
6.23

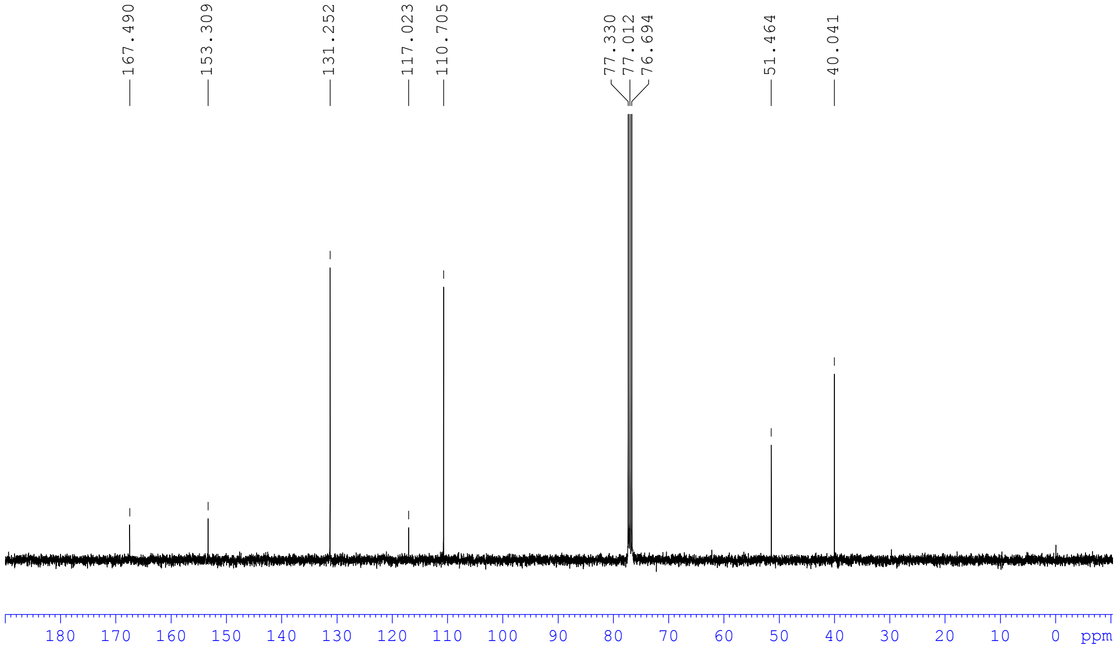
167.490
153.309
131.252
117.023
110.705
77.330
77.012
76.694
51.464
40.041
180 170 160 150 140 130 120 110 100 90 80 70 60 50 40 30 20 10 0 ppm

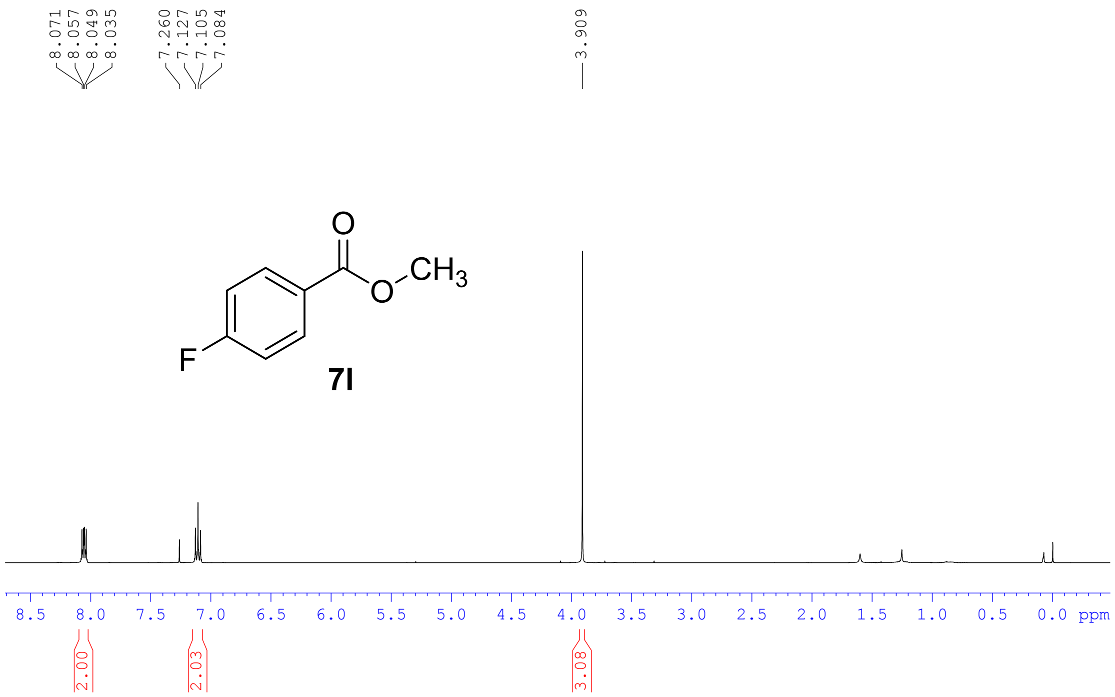
8.071
8.057
8.049
8.035
7.260
7.127
7.105
7.084
3.909
O
CH3
F
7l
8.5 8.0 7.5 7.0 6.5 6.0 5.5 5.0 4.5 4.0 3.5 3.0 2.5 2.0 1.5 1.0 0.5 0.0 ppm
2.00
2.03
3.08

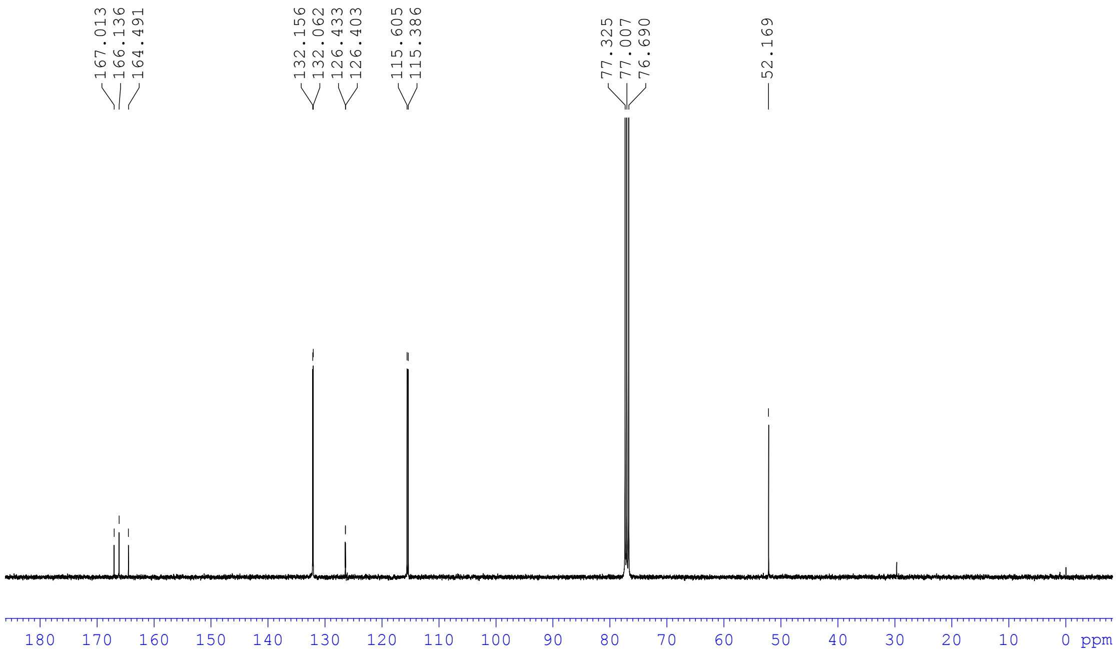
167.013
166.136
164.491
132.156
132.062
126.433
126.403
115.605
115.386
77.325
77.007
76.690
52.169
180 170 160 150 140 130 120 110 100 90 80 70 60 50 40 30 20 10 0 ppm

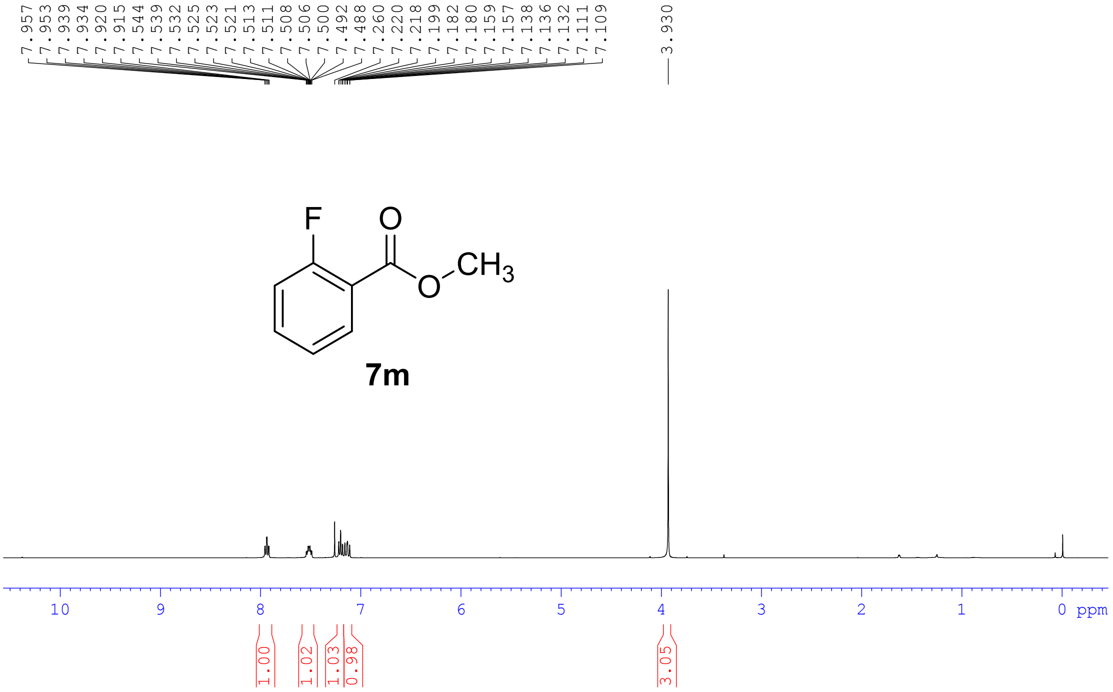
7.957
7.953
7.939
7.934
7.920
7.915
7.544
7.539
7.532
7.525
7.523
7.521
7.513
7.511
7.508
7.506
7.500
7.492
7.488
7.260
7.220
7.218
7.199
7.182
7.180
7.159
7.157
7.138
7.136
7.132
7.111
7.109
3.930
F
O
O
CH3
7m
10
9
8
7
6
5
4
3
2
1
0 ppm
1.00
1.02
1.03
0.98
3.05

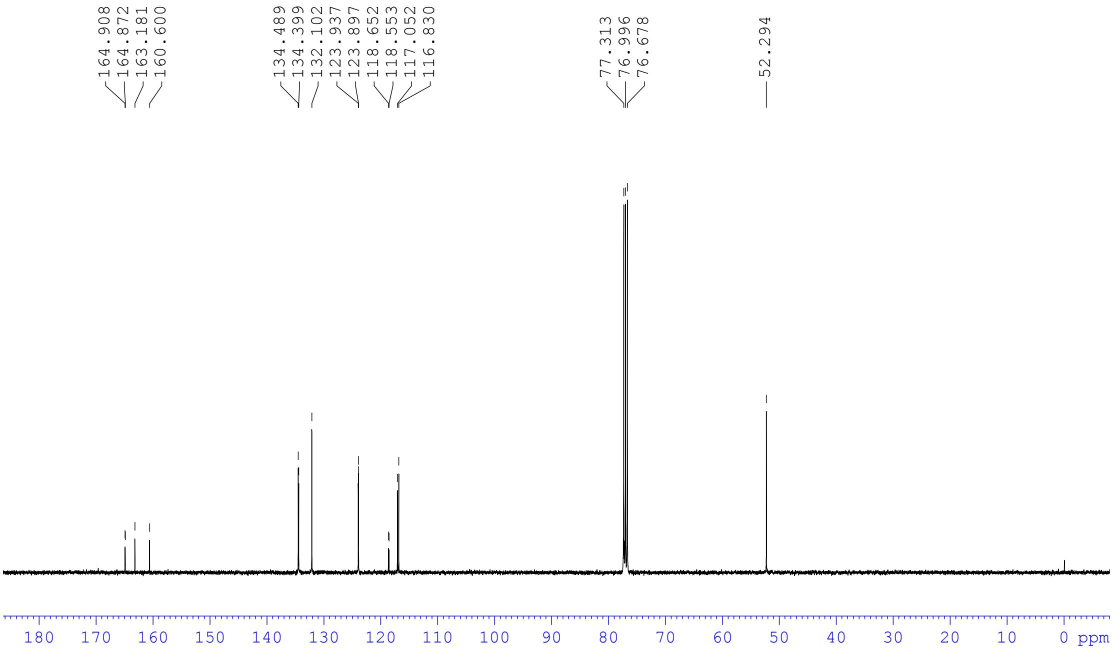
164.908
164.872
163.181
160.600
134.489
134.399
132.102
123.937
123.897
118.652
118.553
117.052
116.830
77.313
76.996
76.678
52.294
180
170
160
150
140
130
120
110
100
90
80
70
60
50
40
30
20
10
0 ppm

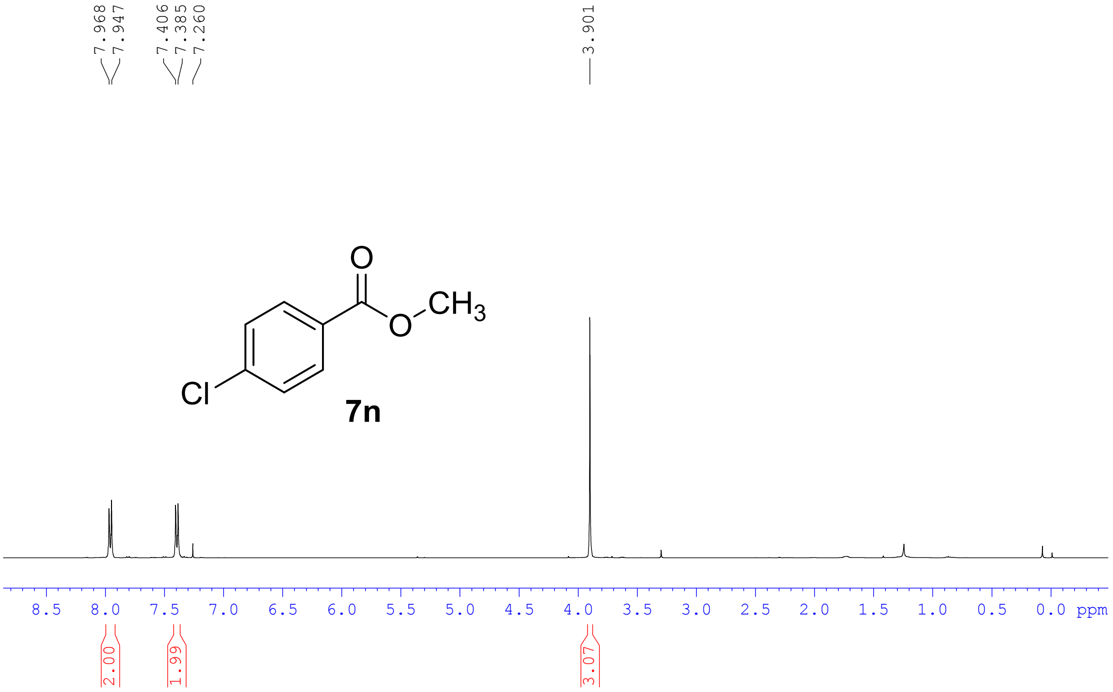
7.968
7.947
7.406
7.385
7.260
3.901
O
O
CH3
Cl
7n
8.5 8.0 7.5 7.0 6.5 6.0 5.5 5.0 4.5 4.0 3.5 3.0 2.5 2.0 1.5 1.0 0.5 0.0 ppm
2.00
1.99
3.07

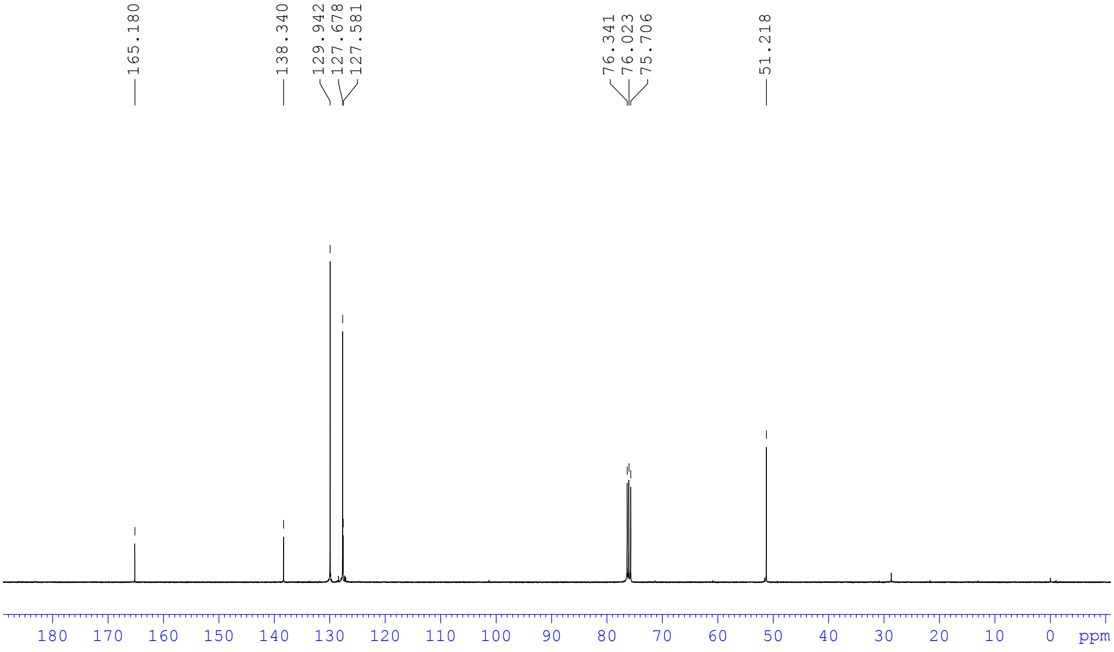
165.180
138.340
129.942
127.678
127.581
76.341
76.023
75.706
51.218
180 170 160 150 140 130 120 110 100 90 80 70 60 50 40 30 20 10 0 ppm

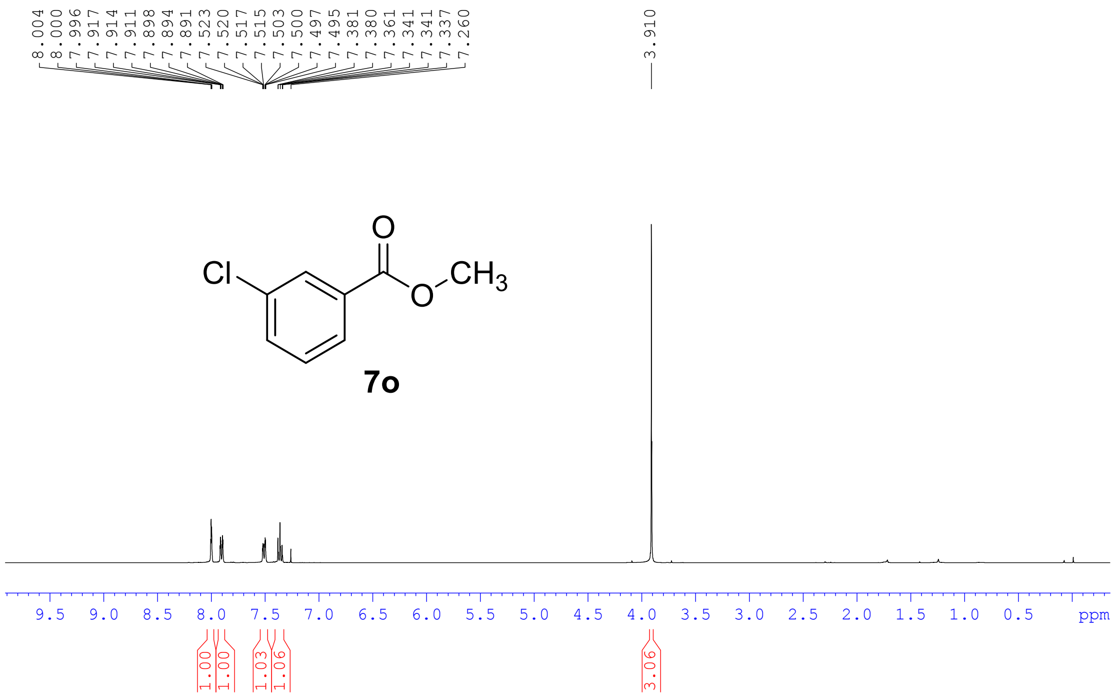

8.004
8.000
7.996
7.917
7.914
7.911
7.898
7.894
7.891
7.523
7.520
7.517
7.515
7.503
7.500
7.497
7.495
7.381
7.380
7.361
7.341
7.337
7.260
3.910
Cl
O
O
CH3
7o
9.5 9.0 8.5 8.0 7.5 7.0 6.5 6.0 5.5 5.0 4.5 4.0 3.5 3.0 2.5 2.0 1.5 1.0 0.5 ppm
1.00
1.00
1.03
1.06
3.06

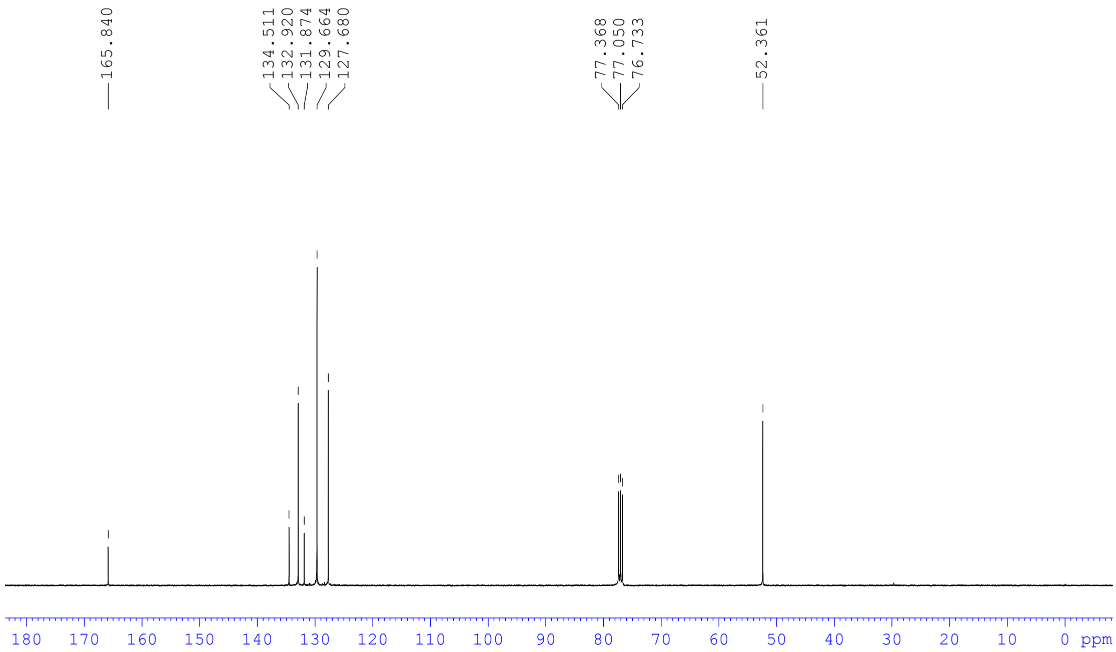

165.840
134.511
132.920
131.874
129.664
127.680
77.368
77.050
76.733
52.361
180 170 160 150 140 130 120 110 100 90 80 70 60 50 40 30 20 10 0 ppm

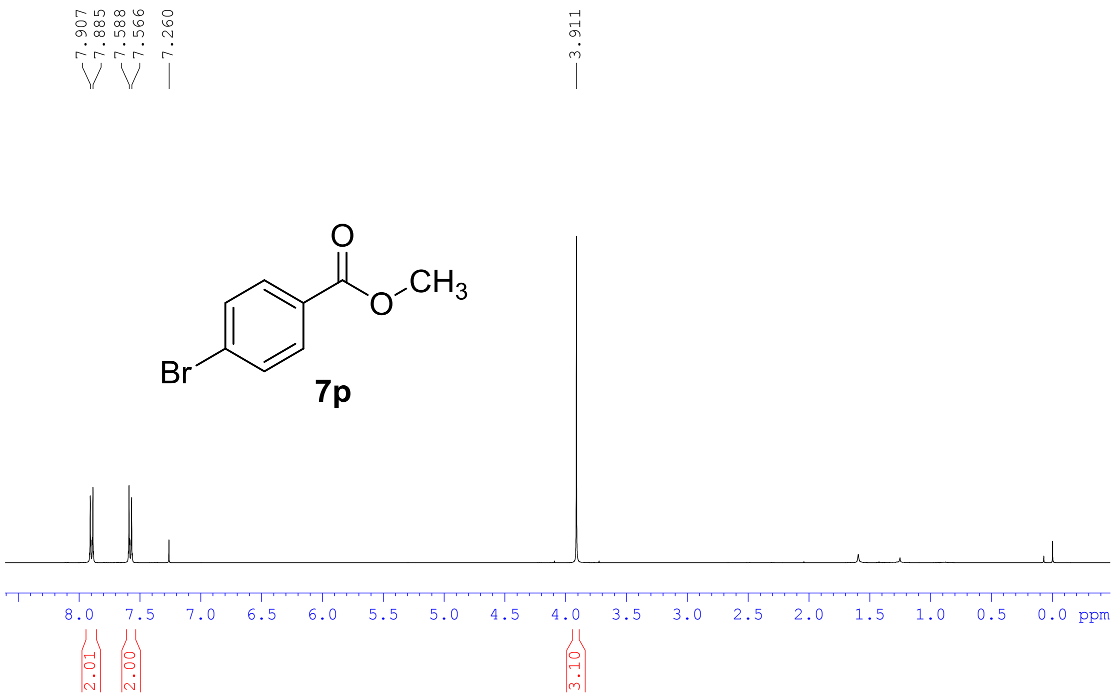
7.907
7.885
7.588
7.566
7.260
3.911
O
O
CH3
Br
7p
8.0 7.5 7.0 6.5 6.0 5.5 5.0 4.5 4.0 3.5 3.0 2.5 2.0 1.5 1.0 0.5 0.0 ppm
2.01
2.00
3.10

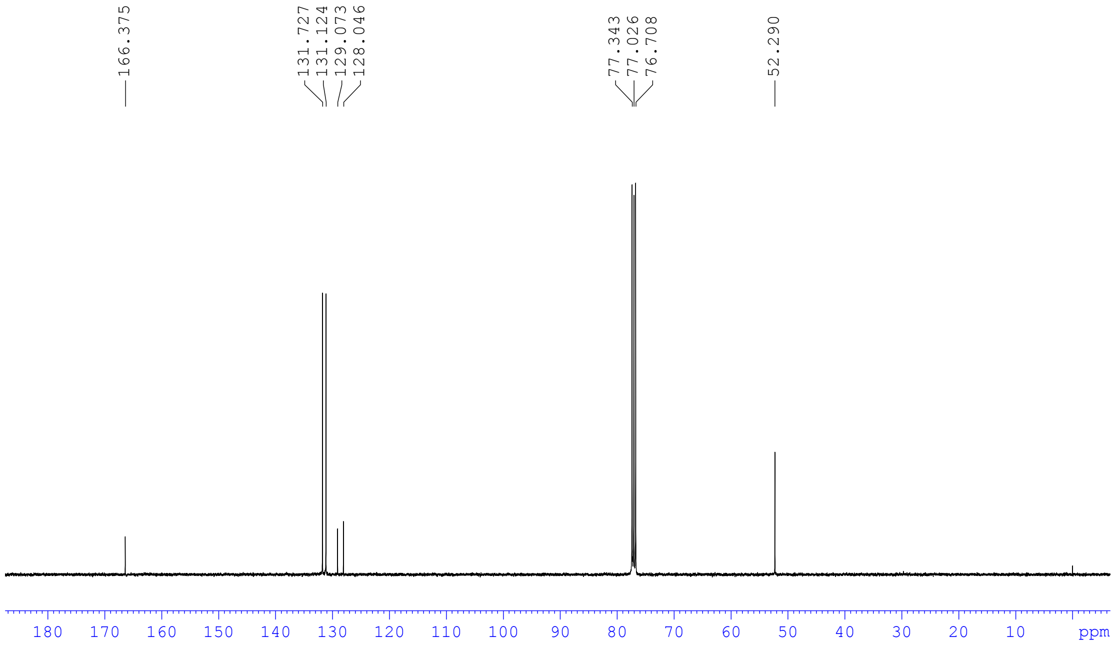
166.375
131.727
131.124
129.073
128.046
77.343
77.026
76.708
52.290
180 170 160 150 140 130 120 110 100 90 80 70 60 50 40 30 20 10 ppm

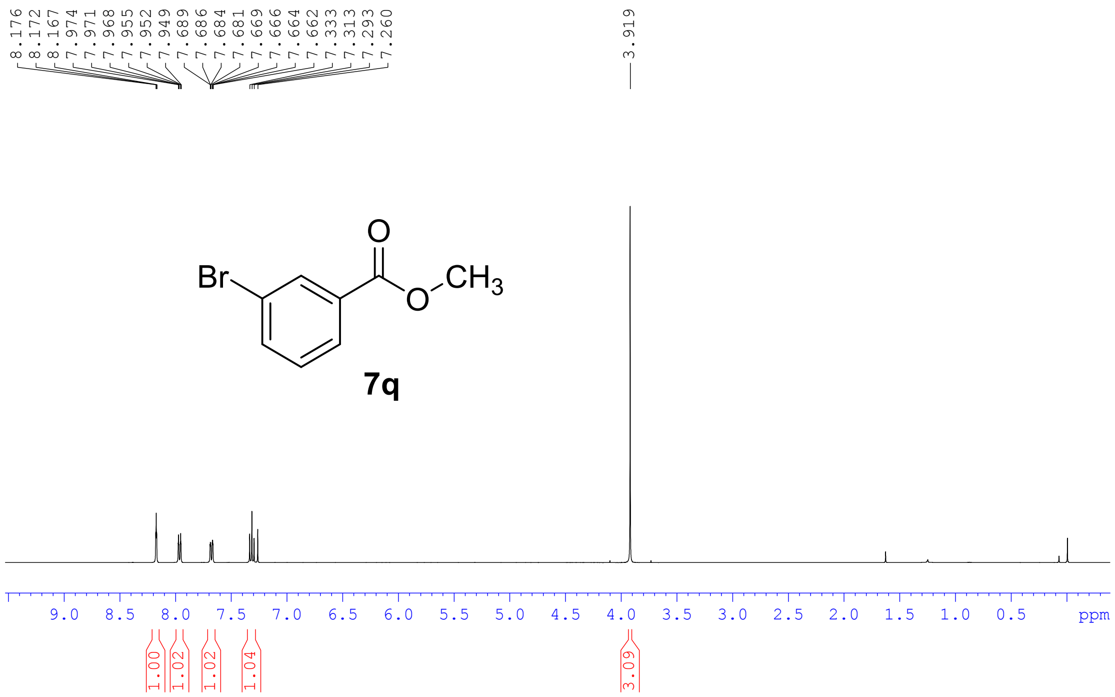
8.176
8.172
8.167
7.974
7.971
7.968
7.955
7.952
7.949
7.689
7.686
7.684
7.681
7.669
7.666
7.664
7.662
7.333
7.313
7.293
7.260
3.919
Br
O
O
CH3
7q
9.0 8.5 8.0 7.5 7.0 6.5 6.0 5.5 5.0 4.5 4.0 3.5 3.0 2.5 2.0 1.5 1.0 0.5 ppm
1.00
1.02
1.02
1.04
3.09

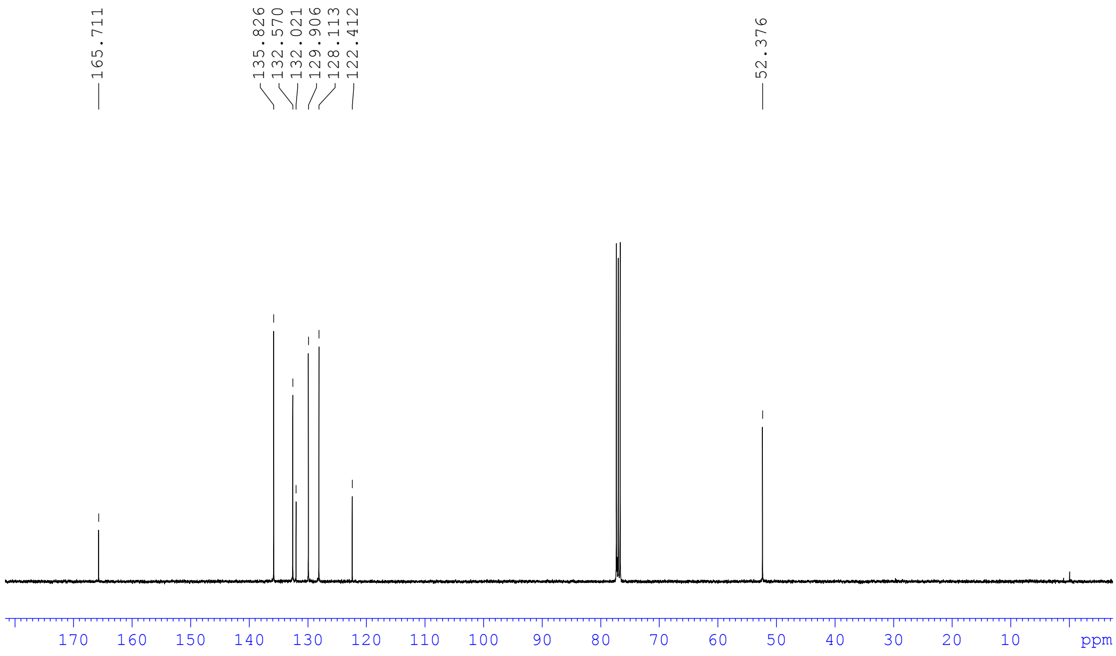
165.711
135.826
132.570
132.021
129.906
128.113
122.412
52.376
170 160 150 140 130 120 110 100 90 80 70 60 50 40 30 20 10 ppm

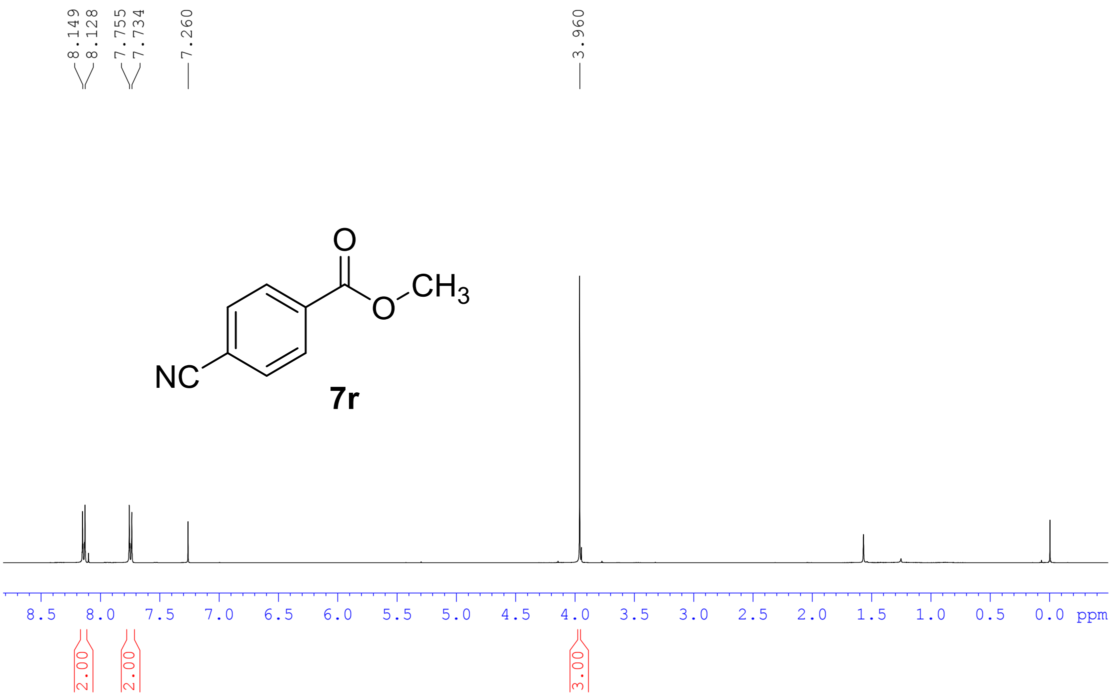
8.149
8.128
7.755
7.734
7.260
3.960
7r
8.5 8.0 7.5 7.0 6.5 6.0 5.5 5.0 4.5 4.0 3.5 3.0 2.5 2.0 1.5 1.0 0.5 0.0 ppm
2.00
2.00
3.00

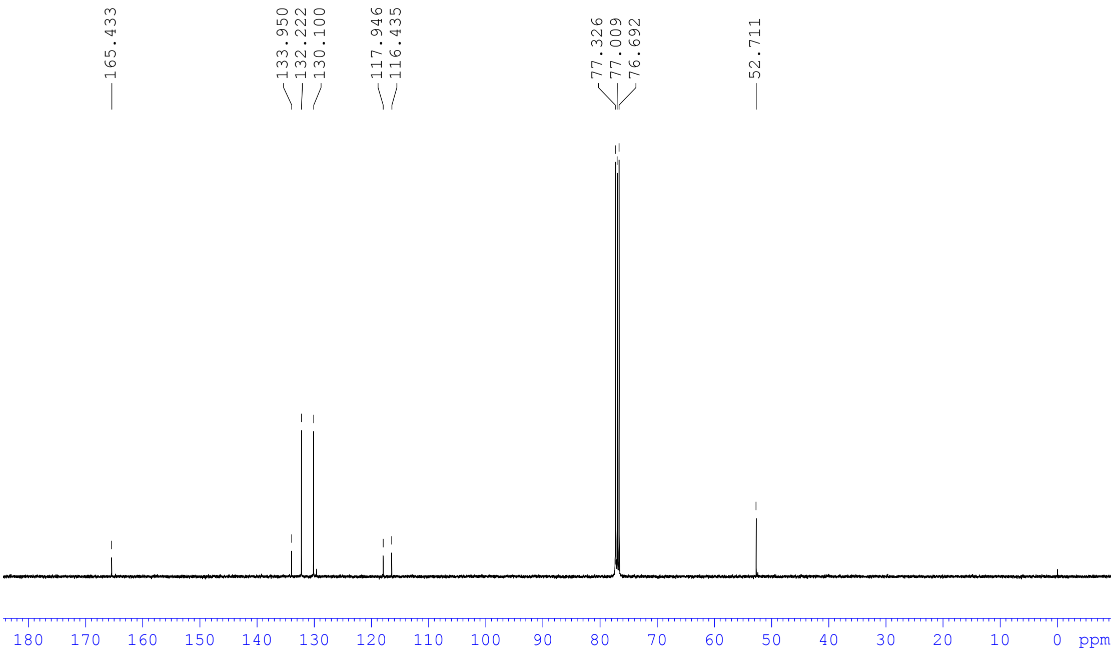
165.433
133.950
132.222
130.100
117.946
116.435
77.326
77.009
76.692
52.711
180 170 160 150 140 130 120 110 100 90 80 70 60 50 40 30 20 10 0 ppm

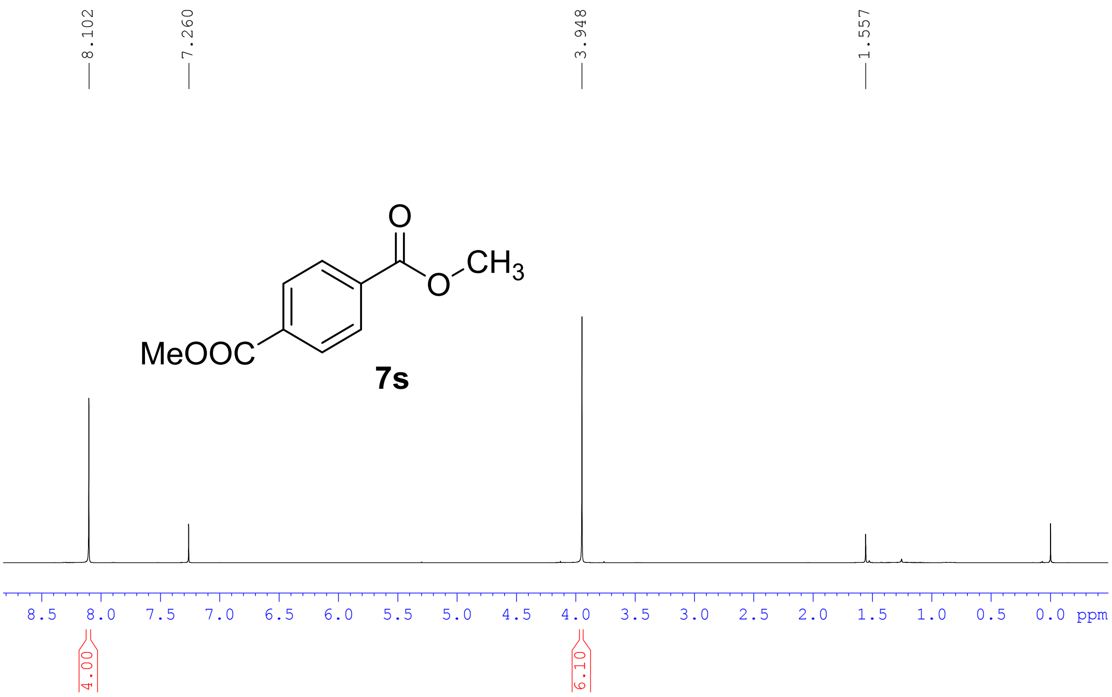
8.102
7.260
3.948
1.557
MeOOC
O
O
CH3
7s
8.5 8.0 7.5 7.0 6.5 6.0 5.5 5.0 4.5 4.0 3.5 3.0 2.5 2.0 1.5 1.0 0.5 0.0 ppm
4.00
6.10

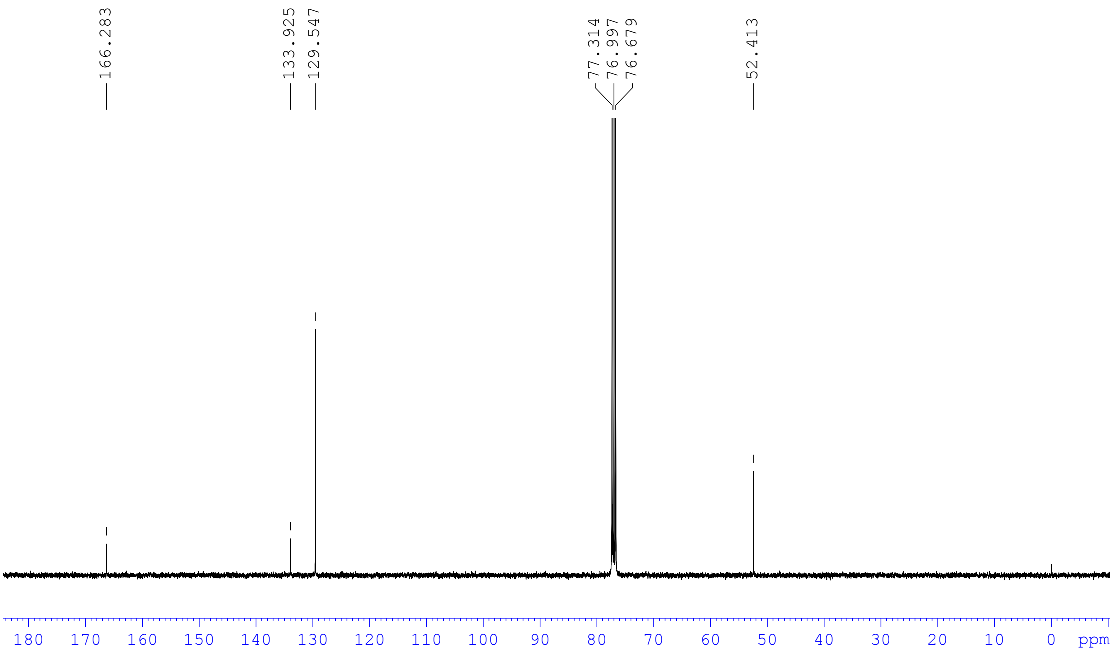
166.283
133.925
129.547
77.314
76.997
76.679
52.413
180 170 160 150 140 130 120 110 100 90 80 70 60 50 40 30 20 10 0 ppm

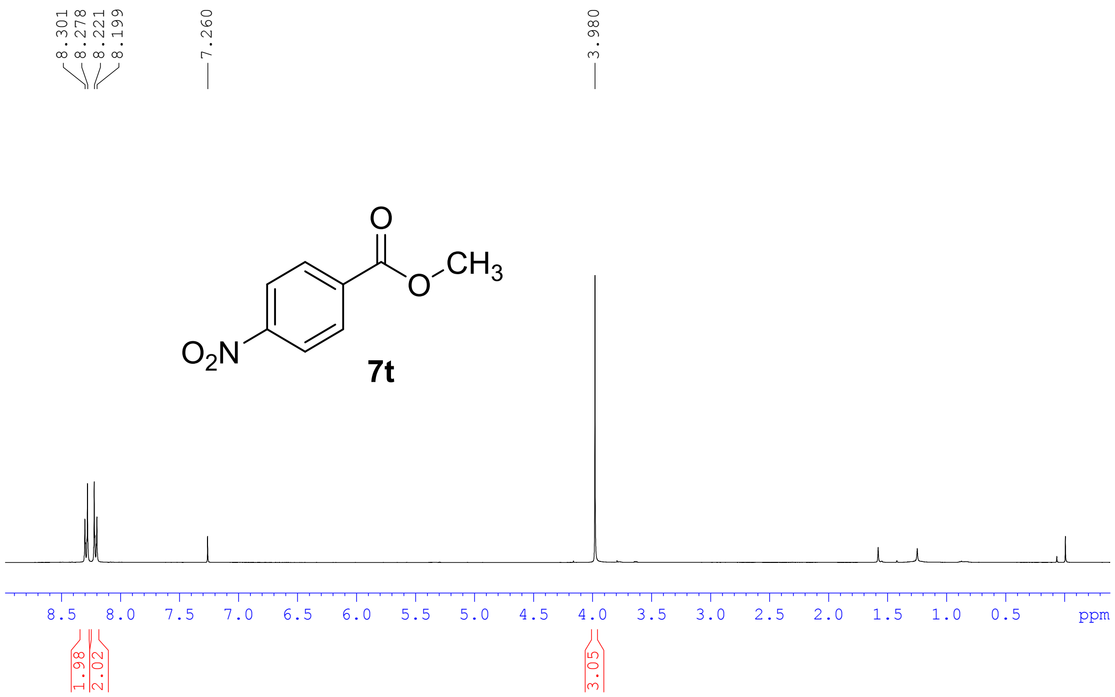

8.301
8.278
8.221
8.199
7.260
3.980
O
CH3
O2N
O
7t
1.98
2.02
3.05
ppm

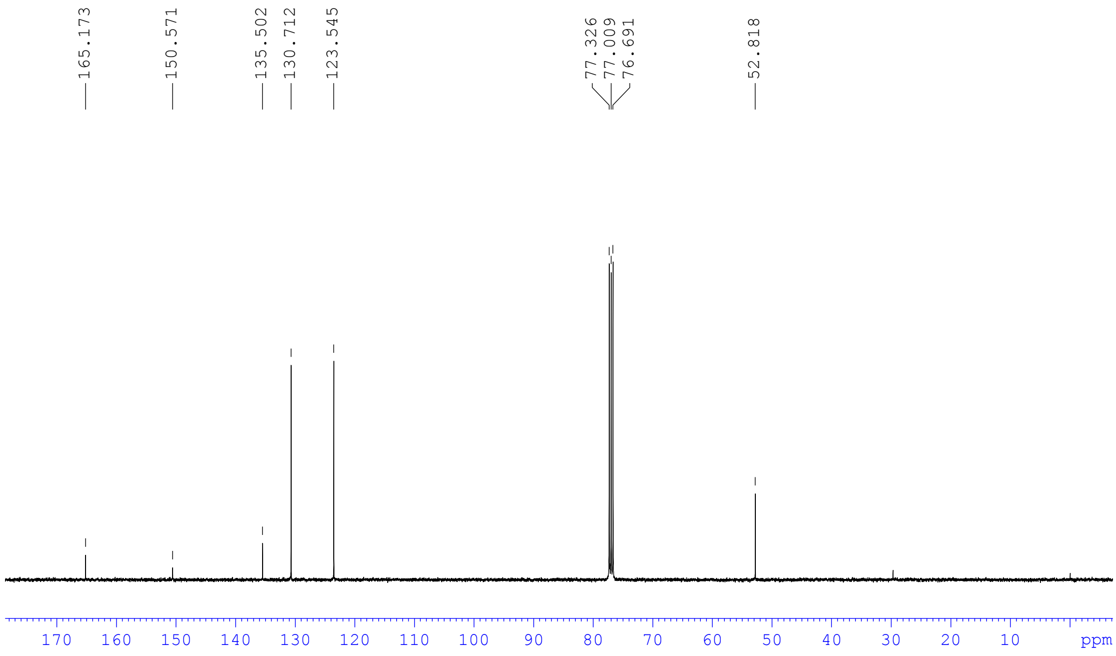

165.173
150.571
135.502
130.712
123.545
77.326
77.009
76.691
52.818
ppm

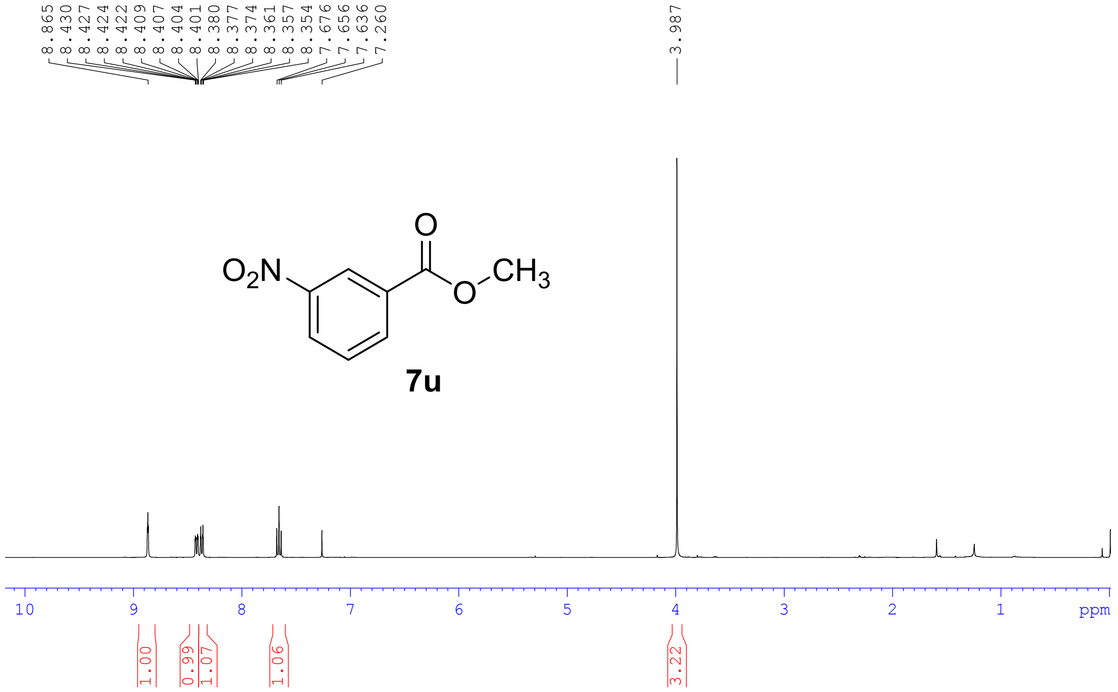
8.865
8.430
8.427
8.424
8.422
8.409
8.407
8.404
8.401
8.380
8.377
8.374
8.361
8.357
8.354
7.676
7.656
7.636
7.260
3.987
O2N
O
O
CH3
7u
1.00
0.99
1.07
1.06
3.22
ppm

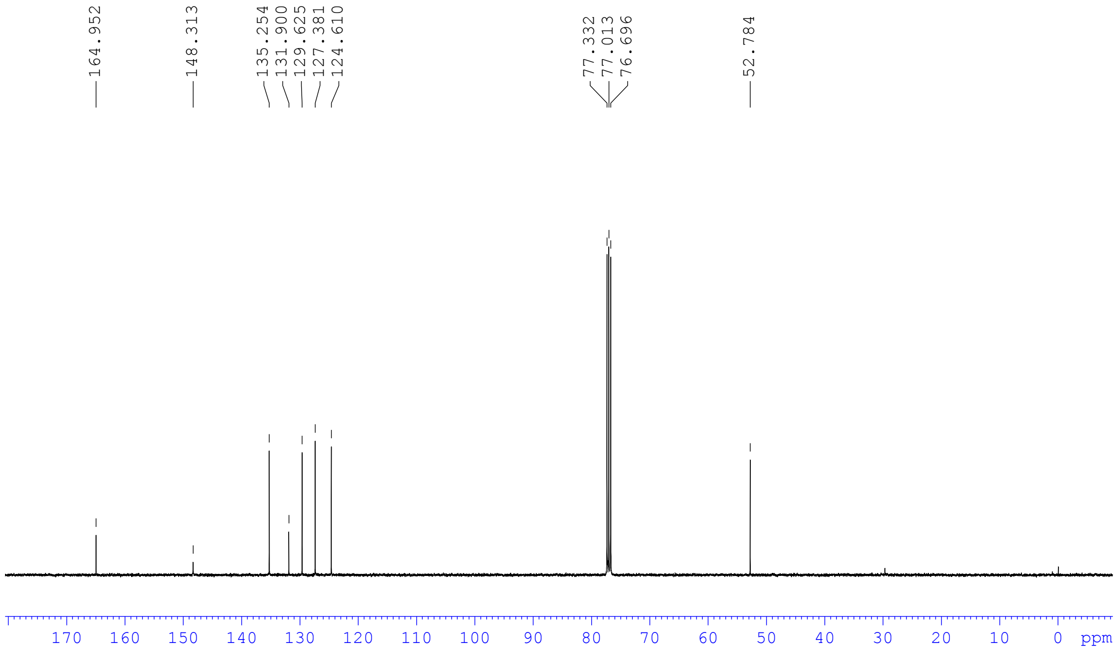
164.952
148.313
135.254
131.900
129.625
127.381
124.610
77.332
77.013
76.696
52.784
ppm

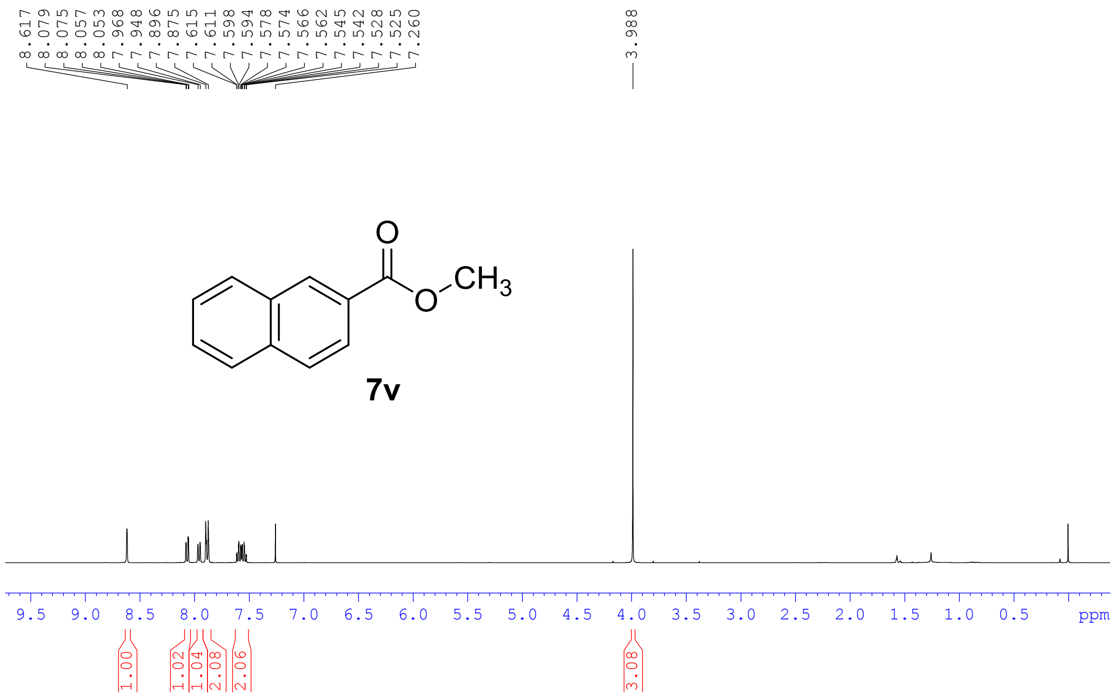
8.617
8.079
8.075
8.057
8.053
7.968
7.948
7.896
7.875
7.615
7.611
7.598
7.594
7.578
7.574
7.566
7.562
7.545
7.542
7.528
7.525
7.260
3.988
O
O
CH3
7v
9.5 9.0 8.5 8.0 7.5 7.0 6.5 6.0 5.5 5.0 4.5 4.0 3.5 3.0 2.5 2.0 1.5 1.0 0.5 ppm
1.00
1.02
1.04
2.08
2.06
3.08

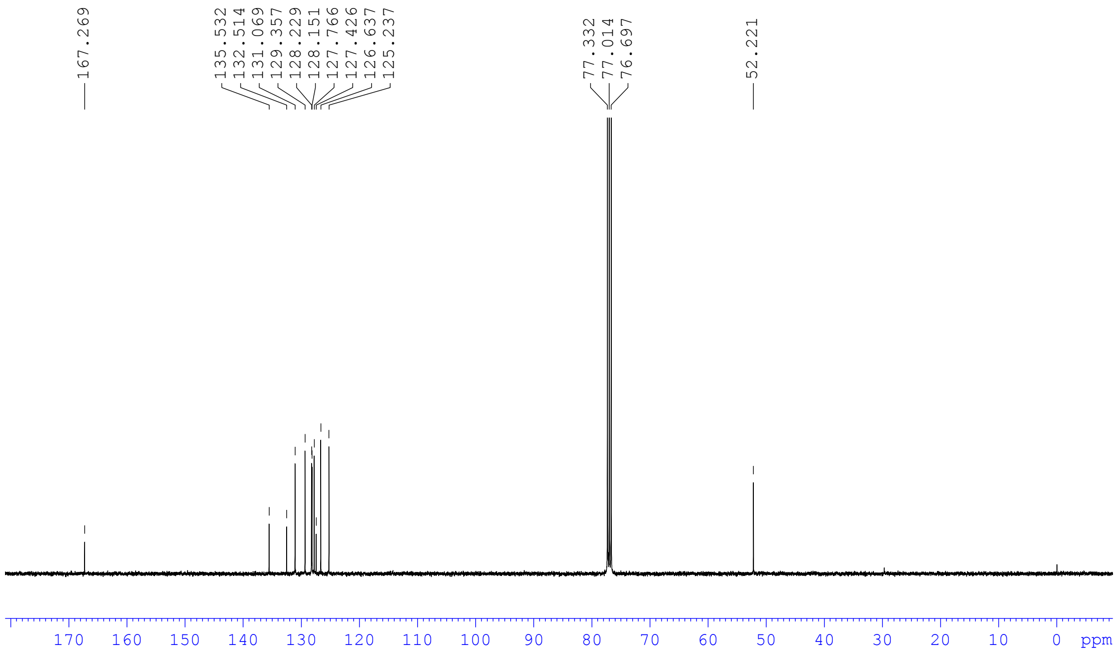
167.269
135.532
132.514
131.069
129.357
128.229
128.151
127.766
127.426
126.637
125.237
77.332
77.014
76.697
52.221
170 160 150 140 130 120 110 100 90 80 70 60 50 40 30 20 10 0 ppm

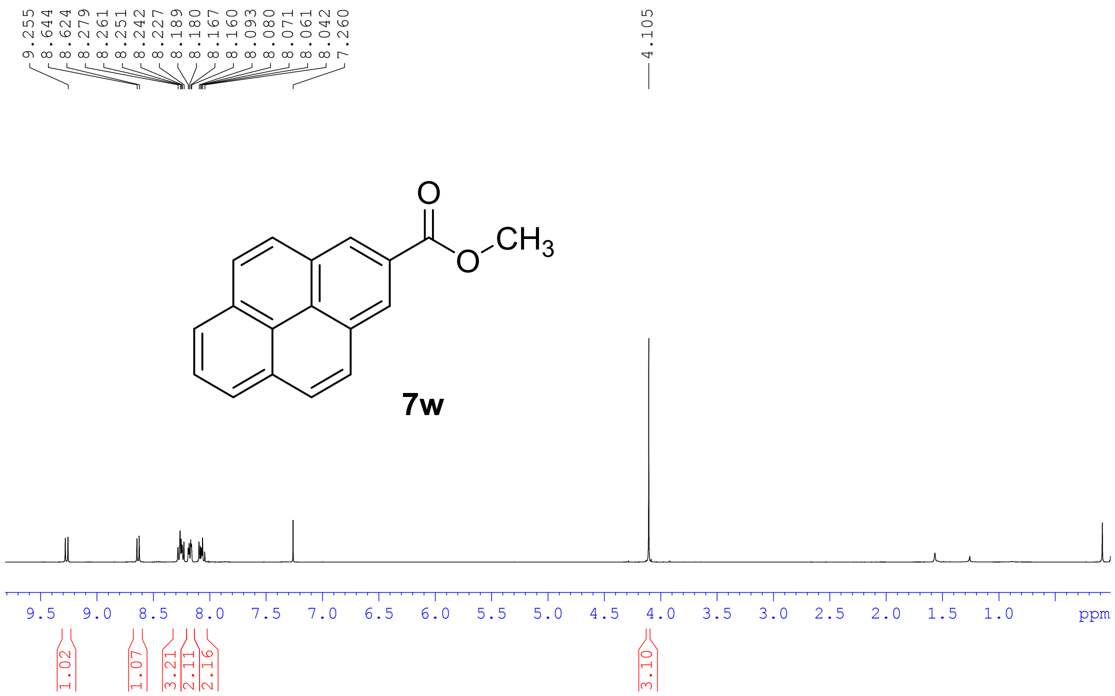
9.255
8.644
8.624
8.279
8.261
8.251
8.242
8.227
8.189
8.180
8.167
8.160
8.093
8.080
8.071
8.061
8.042
7.260
4.105
O
O
CH3
7w
9.5 9.0 8.5 8.0 7.5 7.0 6.5 6.0 5.5 5.0 4.5 4.0 3.5 3.0 2.5 2.0 1.5 1.0 ppm
1.02
1.07
3.21
2.11
2.16
3.10

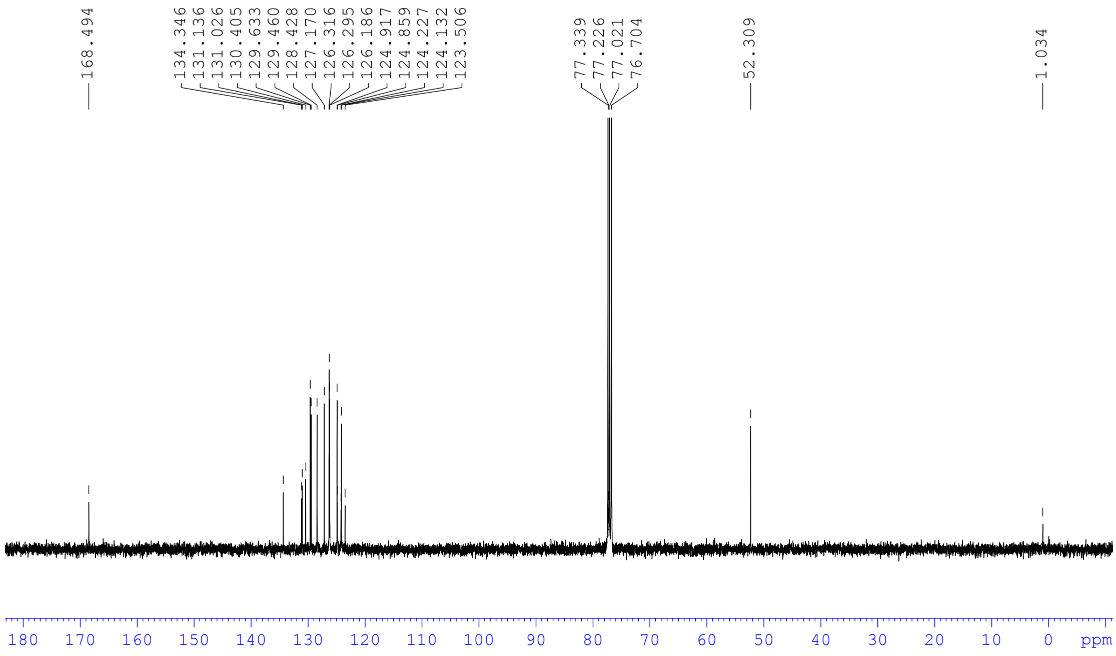
168.494
134.346
131.136
131.026
130.405
129.633
129.460
128.428
127.170
126.316
126.295
126.186
124.917
124.859
124.227
124.132
123.506
77.339
77.226
77.021
76.704
52.309
1.034
180 170 160 150 140 130 120 110 100 90 80 70 60 50 40 30 20 10 0 ppm

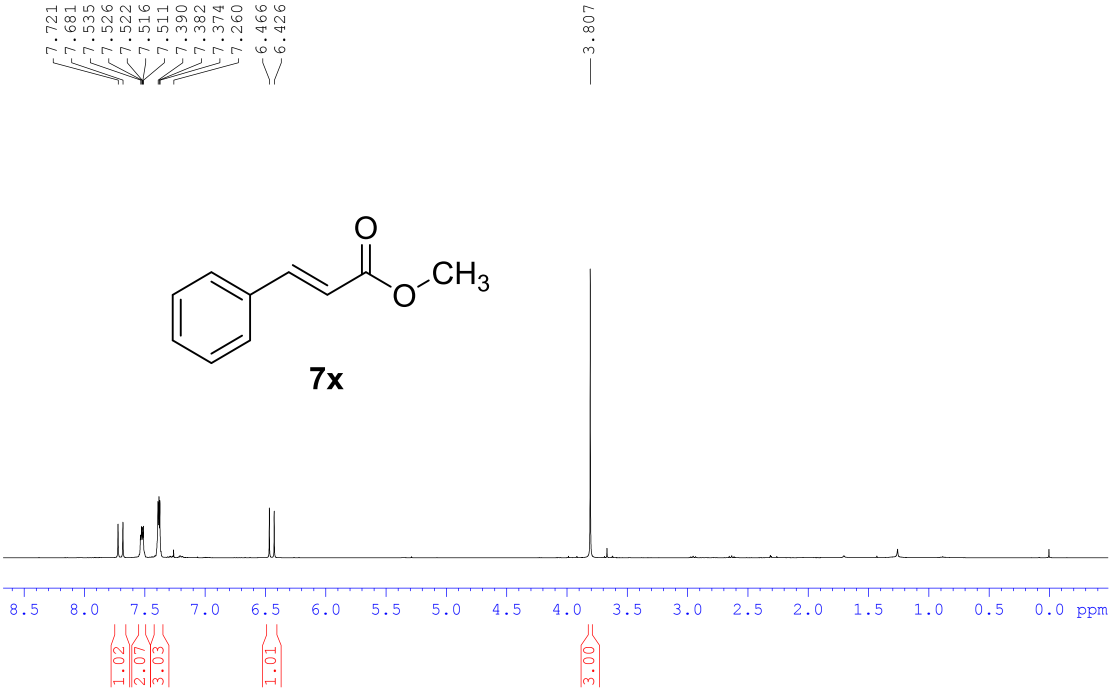
7.721
7.681
7.535
7.526
7.522
7.516
7.511
7.390
7.382
7.374
7.260
6.466
6.426
3.807
O
O
CH3
7x
8.5 8.0 7.5 7.0 6.5 6.0 5.5 5.0 4.5 4.0 3.5 3.0 2.5 2.0 1.5 1.0 0.5 0.0 ppm
1.02
2.07
3.03
1.01
3.00

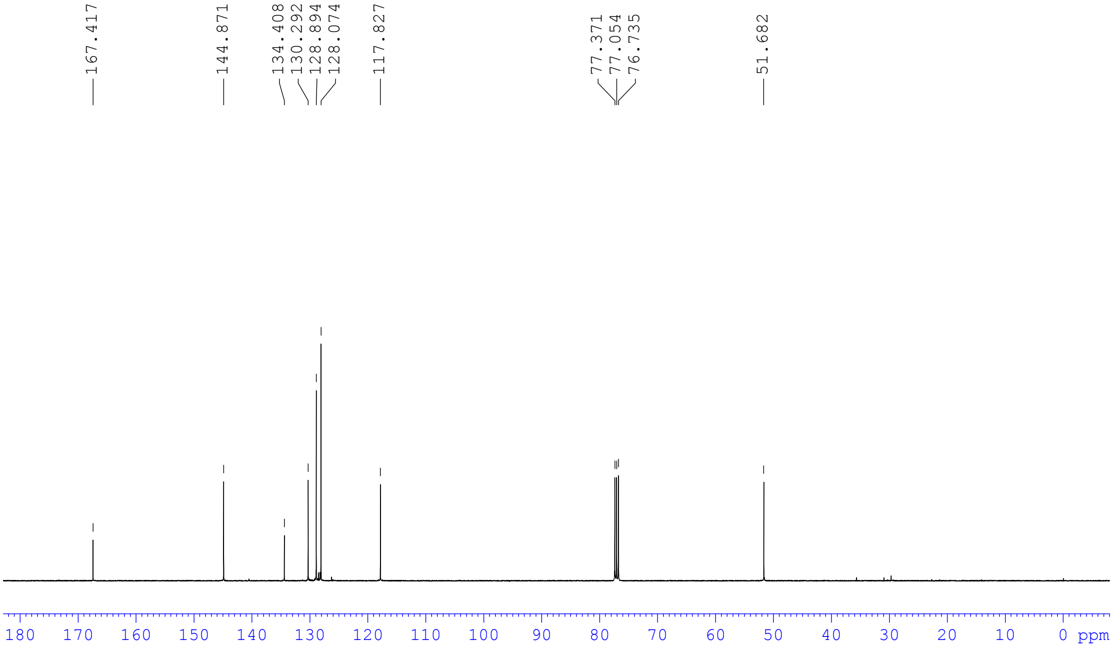
167.417
144.871
134.408
130.292
128.894
128.074
117.827
77.371
77.054
76.735
51.682
180 170 160 150 140 130 120 110 100 90 80 70 60 50 40 30 20 10 0 ppm

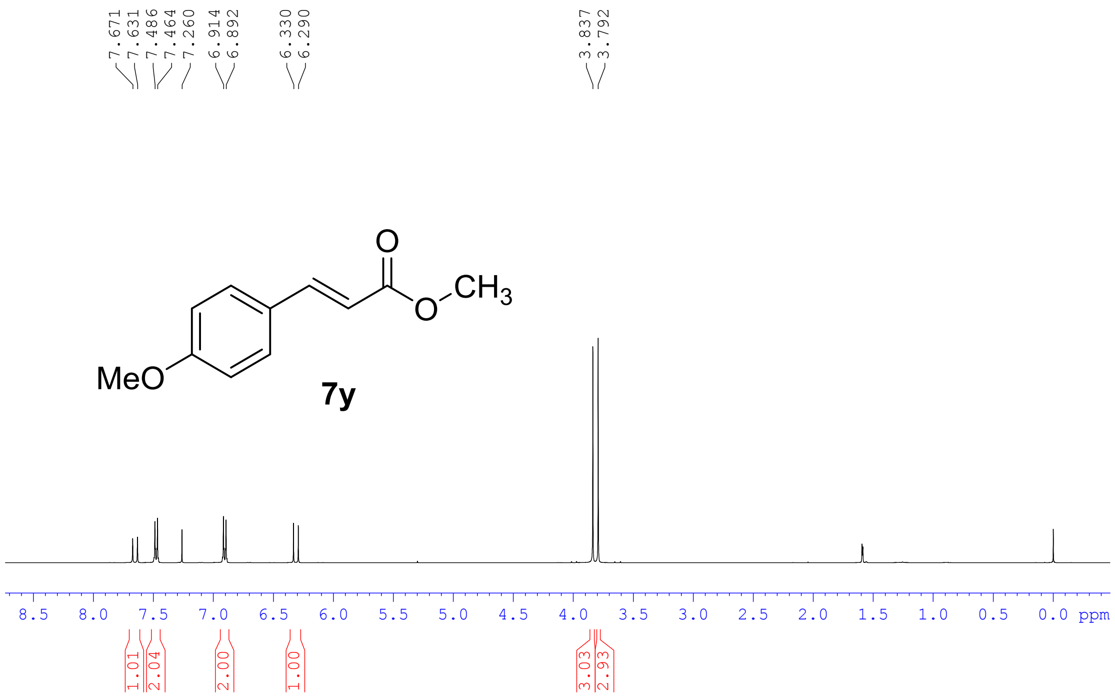
7.671
7.631
7.486
7.464
7.260
6.914
6.892
6.330
6.290
3.837
3.792
MeO
O
O
CH3
7y
8.5 8.0 7.5 7.0 6.5 6.0 5.5 5.0 4.5 4.0 3.5 3.0 2.5 2.0 1.5 1.0 0.5 0.0 ppm
1.01
2.04
2.00
1.00
3.03
2.93

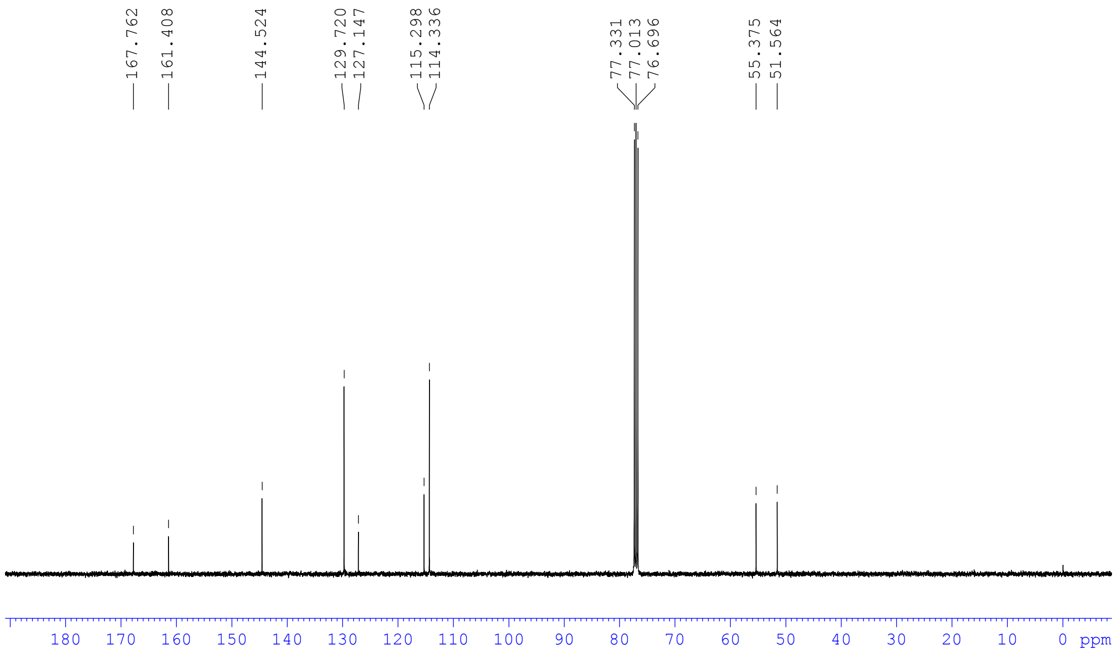
167.762
161.408
144.524
129.720
127.147
115.298
114.336
77.331
77.013
76.696
55.375
51.564
180 170 160 150 140 130 120 110 100 90 80 70 60 50 40 30 20 10 0 ppm

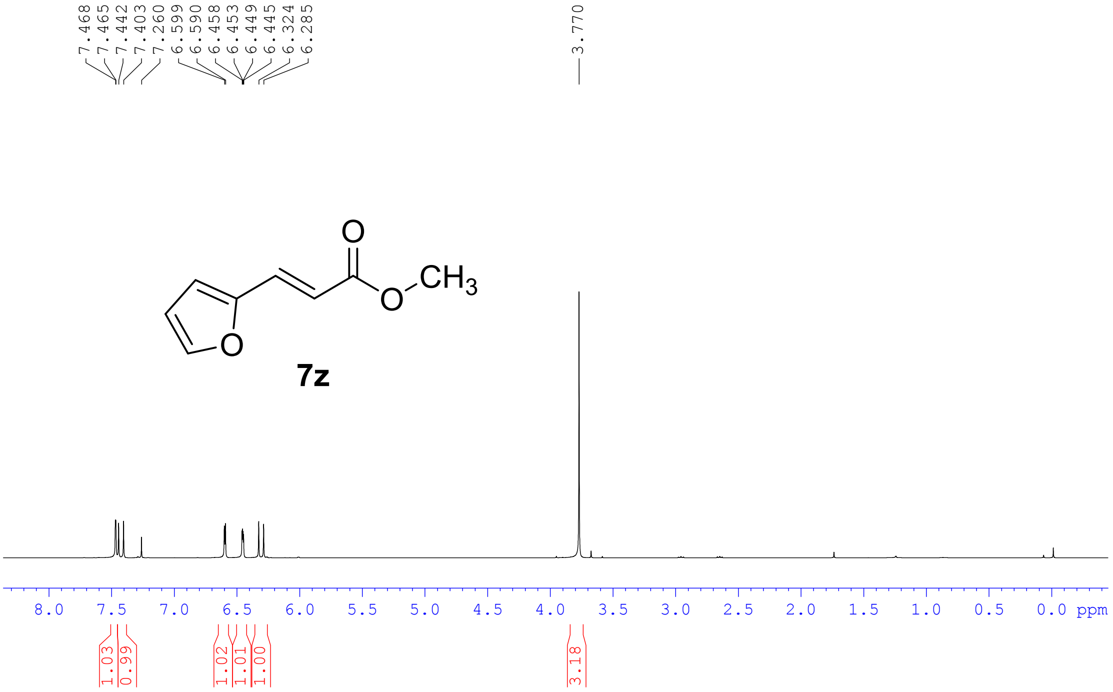
7.468
7.465
7.442
7.403
7.260
6.599
6.590
6.458
6.453
6.449
6.445
6.324
6.285
3.770
O
O
CH3
O
7z
ppm
1.03
0.99
1.02
1.01
1.00
3.18

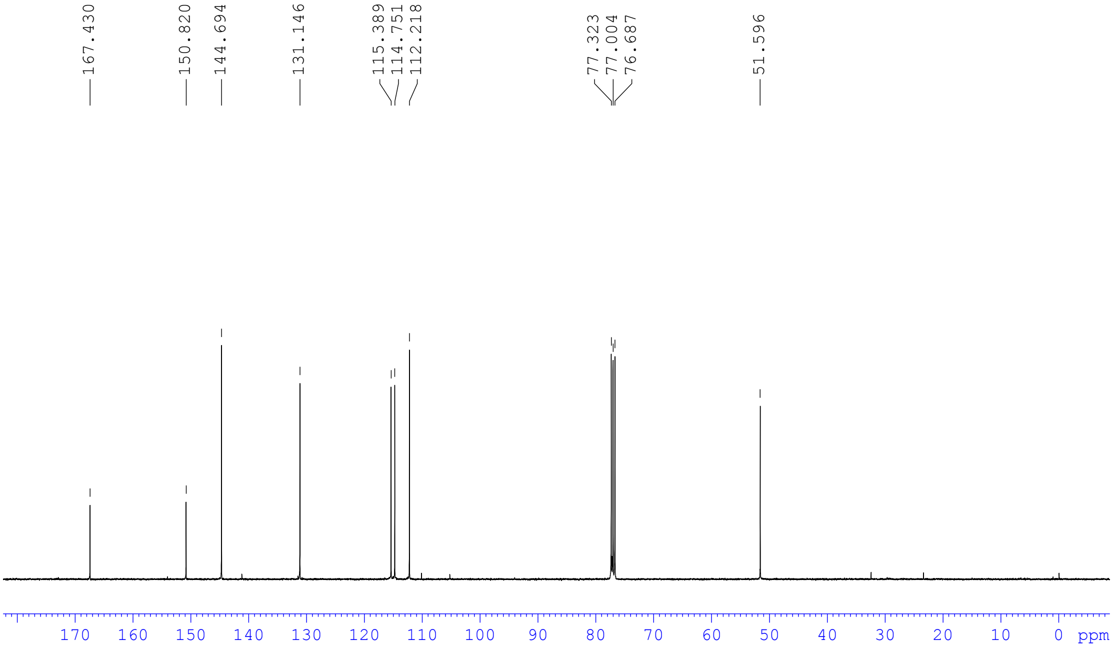
167.430
150.820
144.694
131.146
115.389
114.751
112.218
77.323
77.004
76.687
51.596
ppm

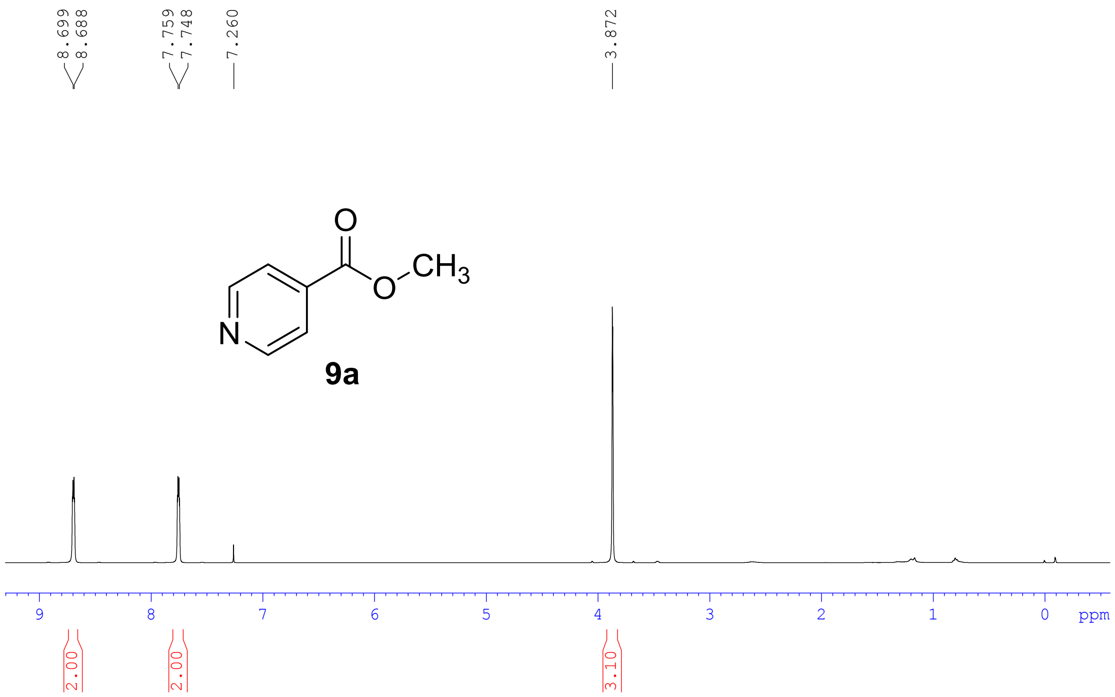
8.699
8.688
7.759
7.748
7.260
3.872
9a
2.00
2.00
3.10
9 8 7 6 5 4 3 2 1 0 ppm

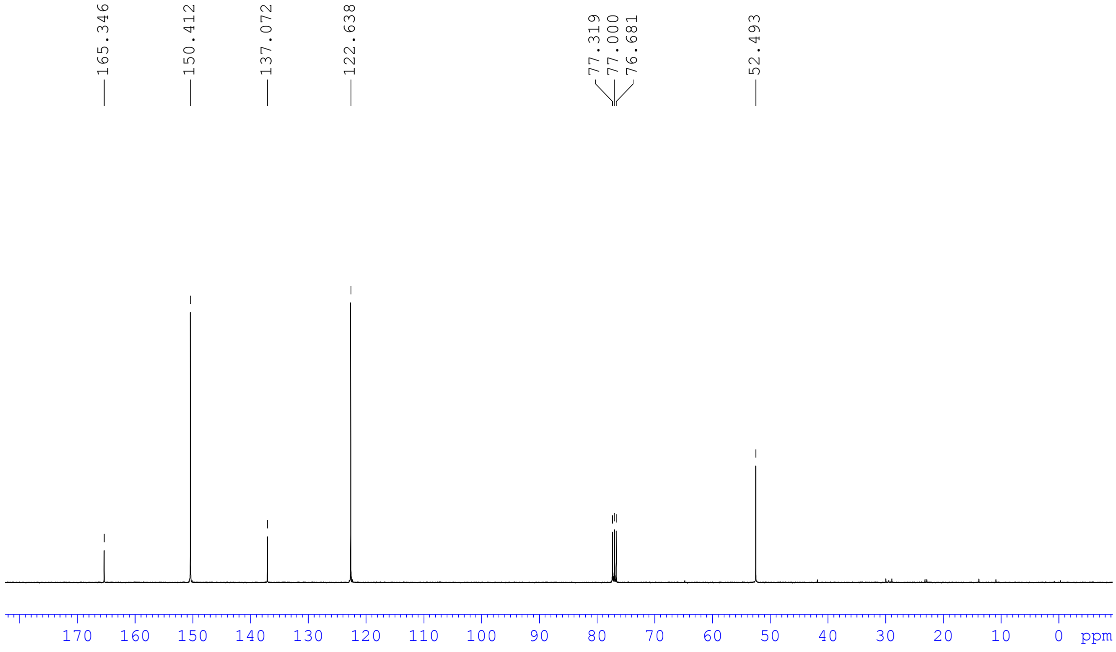
165.346
150.412
137.072
122.638
77.319
77.000
76.681
52.493
170 160 150 140 130 120 110 100 90 80 70 60 50 40 30 20 10 0 ppm

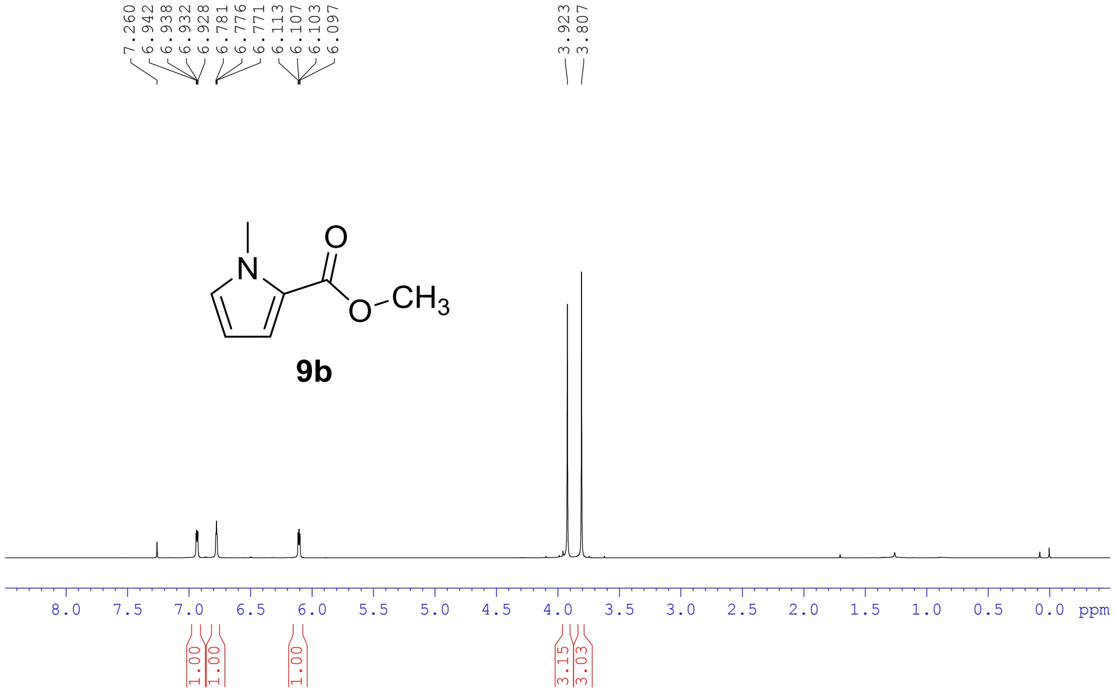
7.260
6.942
6.938
6.932
6.928
6.781
6.776
6.771
6.113
6.107
6.103
6.097
3.923
3.807
N
O
O
CH3
9b
8.0 7.5 7.0 6.5 6.0 5.5 5.0 4.5 4.0 3.5 3.0 2.5 2.0 1.5 1.0 0.5 0.0 ppm
1.00
1.00
1.00
3.15
3.03

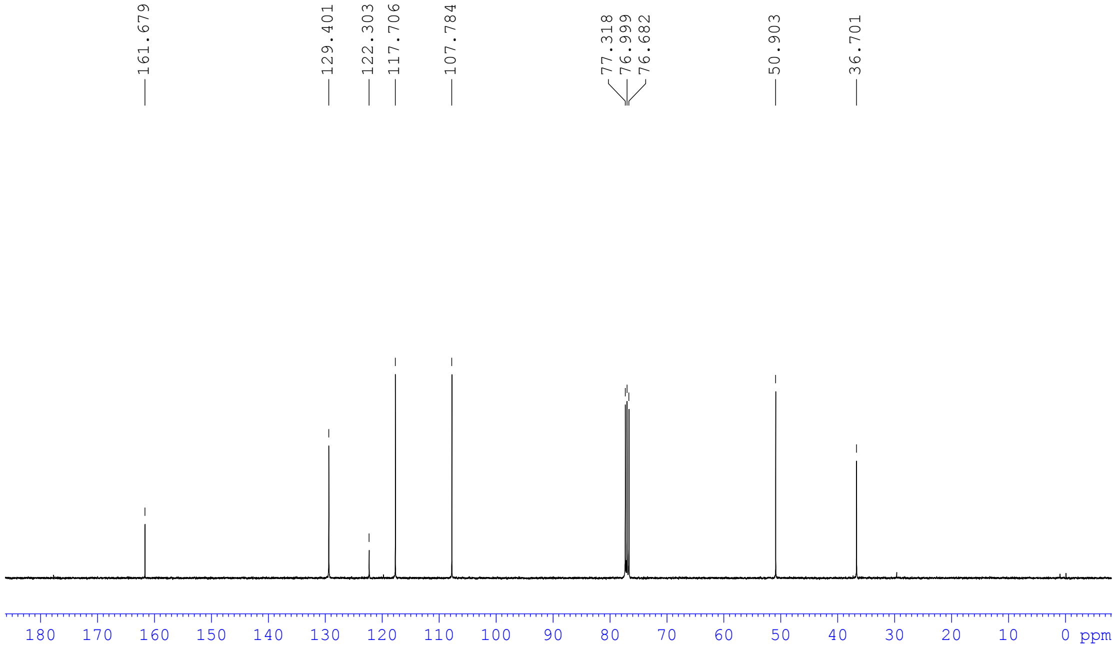
161.679
129.401
122.303
117.706
107.784
77.318
76.999
76.682
50.903
36.701
180 170 160 150 140 130 120 110 100 90 80 70 60 50 40 30 20 10 0 ppm

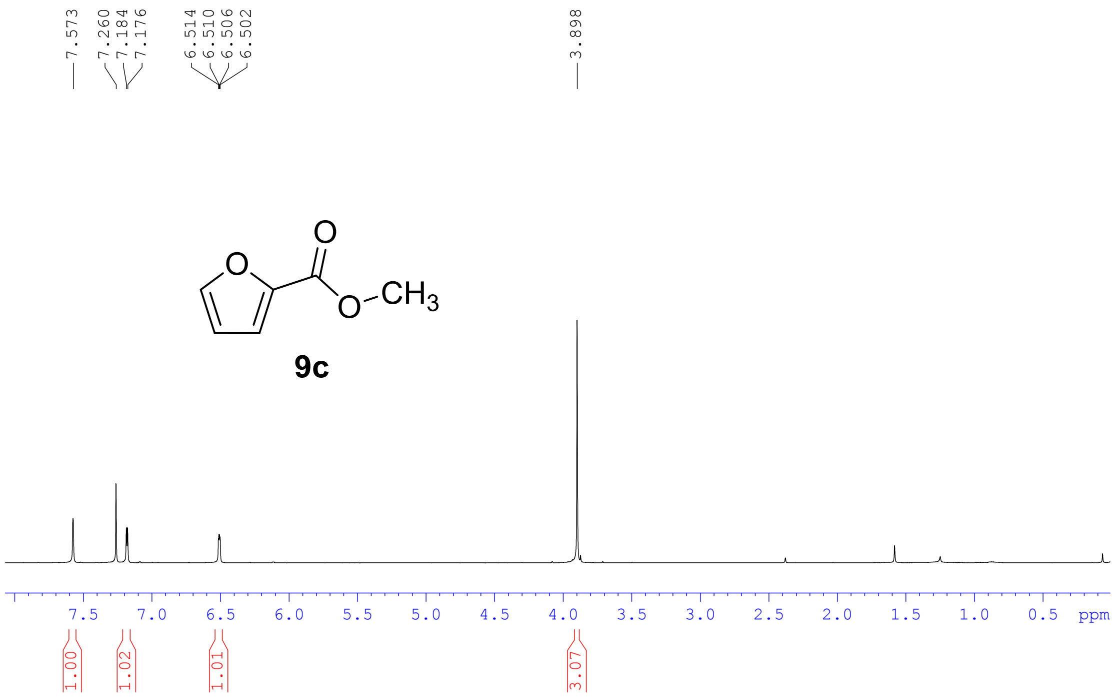

O
O
CH3
9c
7.573
7.260
7.184
7.176
6.514
6.510
6.506
6.502
3.898
7.5 7.0 6.5 6.0 5.5 5.0 4.5 4.0 3.5 3.0 2.5 2.0 1.5 1.0 0.5 ppm
1.00
1.02
1.01
3.07

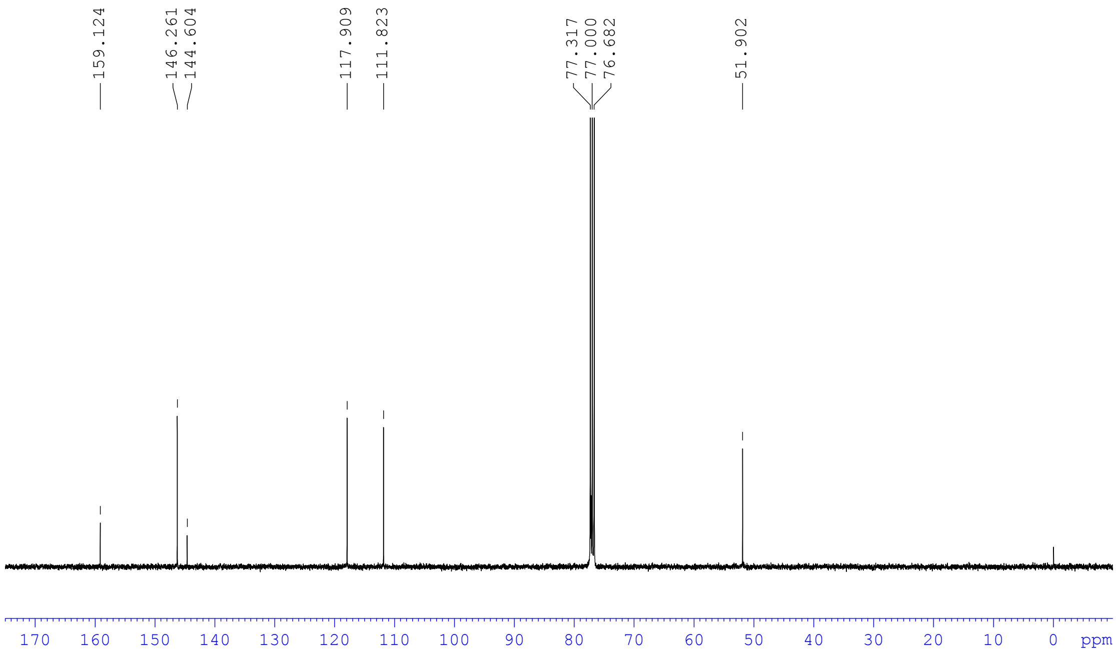

159.124
146.261
144.604
117.909
111.823
77.317
77.000
76.682
51.902
170 160 150 140 130 120 110 100 90 80 70 60 50 40 30 20 10 0 ppm

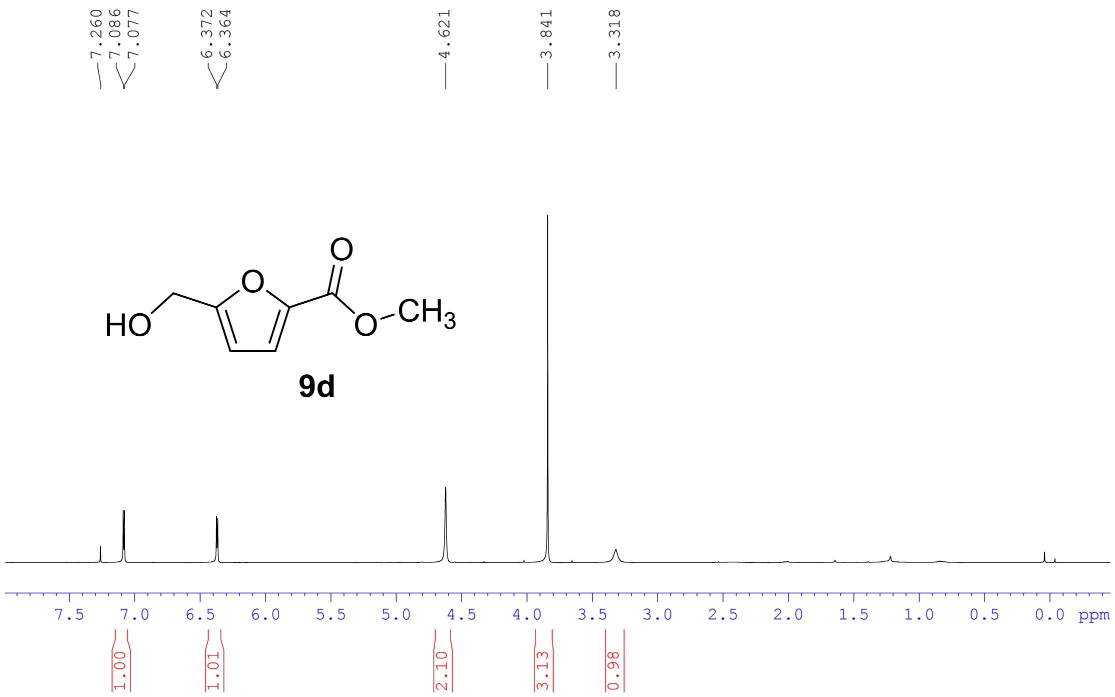
7.260
7.086
7.077
6.372
6.364
4.621
3.841
3.318
HO
O
O
O
CH3
9d
7.5 7.0 6.5 6.0 5.5 5.0 4.5 4.0 3.5 3.0 2.5 2.0 1.5 1.0 0.5 0.0 ppm
1.00
1.01
2.10
3.13
0.98

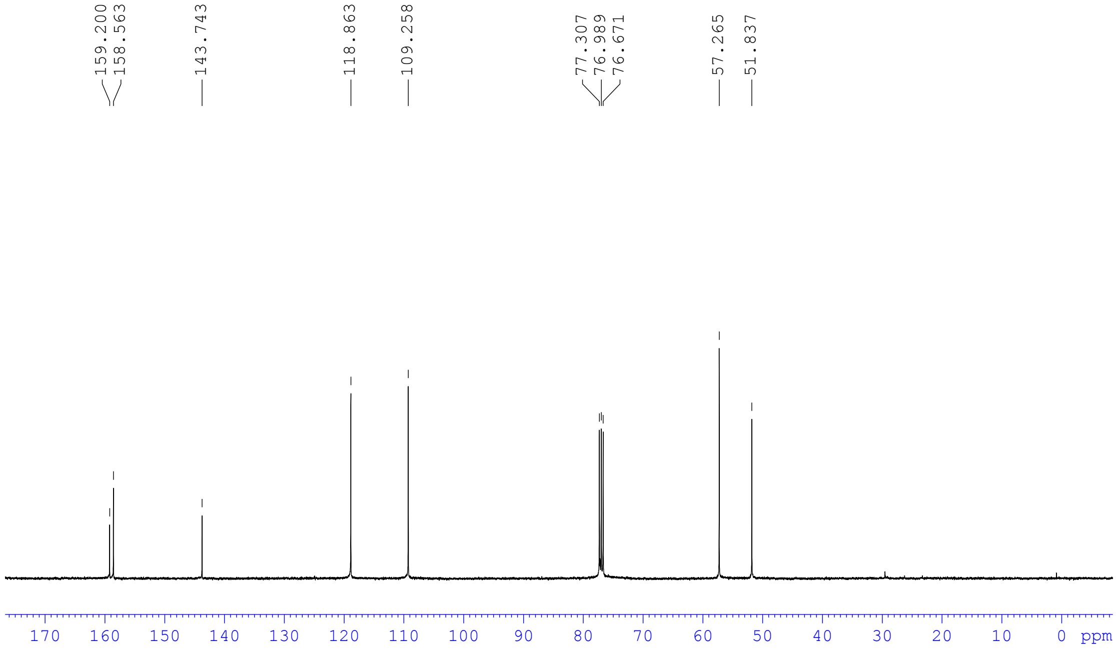
159.200
158.563
143.743
118.863
109.258
77.307
76.989
76.671
57.265
51.837
170 160 150 140 130 120 110 100 90 80 70 60 50 40 30 20 10 0 ppm

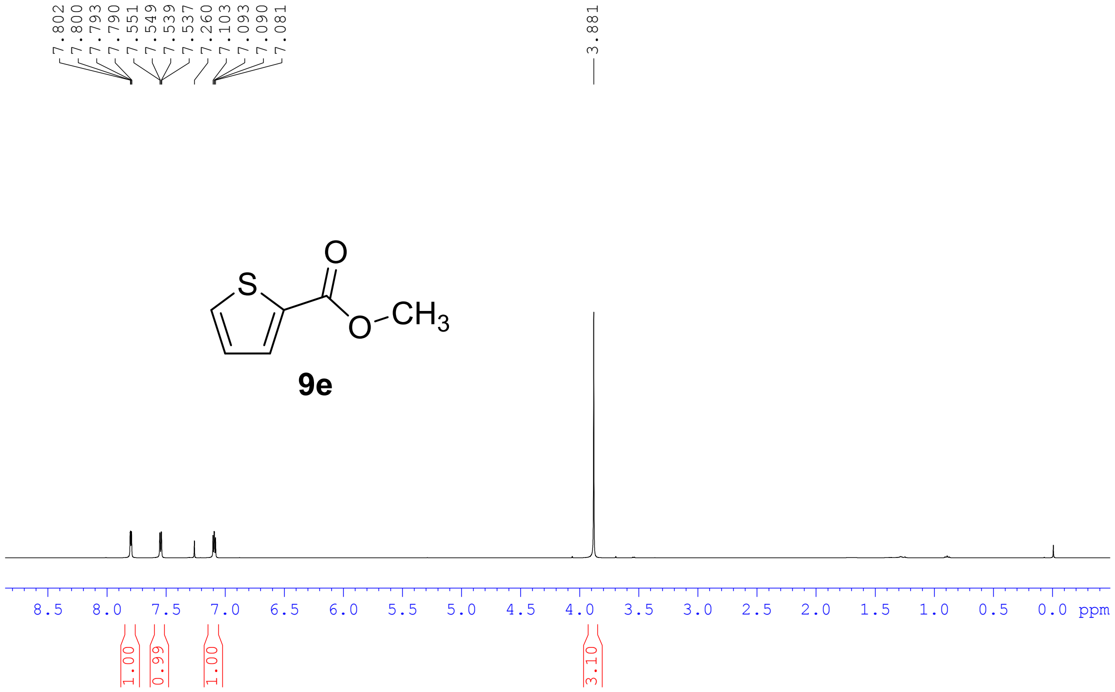
7.802
7.800
7.793
7.790
7.551
7.549
7.539
7.537
7.260
7.103
7.093
7.090
7.081
3.881
S
O
O
CH3
9e
8.5 8.0 7.5 7.0 6.5 6.0 5.5 5.0 4.5 4.0 3.5 3.0 2.5 2.0 1.5 1.0 0.5 0.0 ppm
1.00
0.99
1.00
3.10

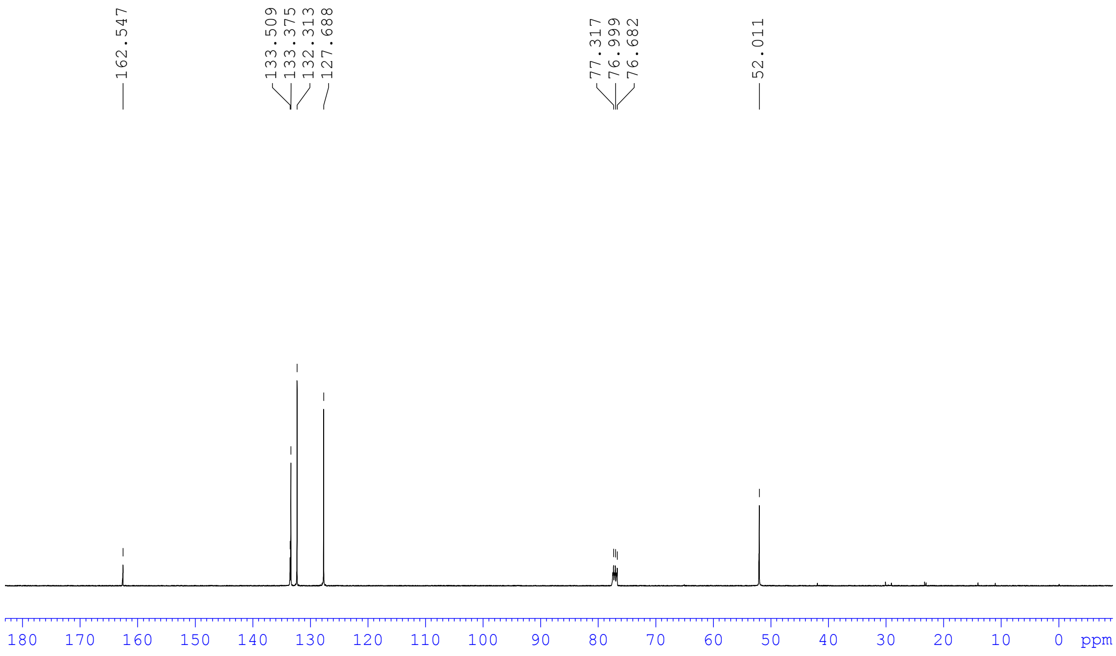
162.547
133.509
133.375
132.313
127.688
77.317
76.999
76.682
52.011
180 170 160 150 140 130 120 110 100 90 80 70 60 50 40 30 20 10 0 ppm

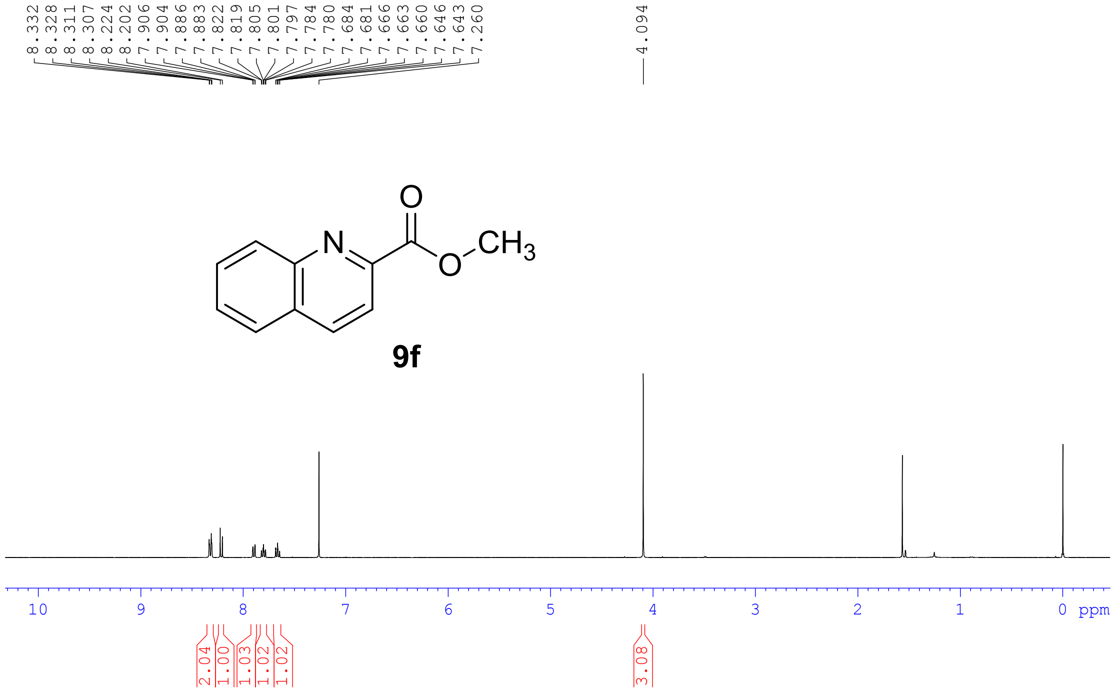
8.332
8.328
8.311
8.307
8.224
8.202
7.906
7.904
7.886
7.883
7.822
7.819
7.805
7.801
7.797
7.784
7.780
7.684
7.681
7.666
7.663
7.660
7.646
7.643
7.260
4.094
N
O
O
CH3
9f
2.04
1.00
1.03
1.02
1.02
3.08
10
9
8
7
6
5
4
3
2
1
0 ppm

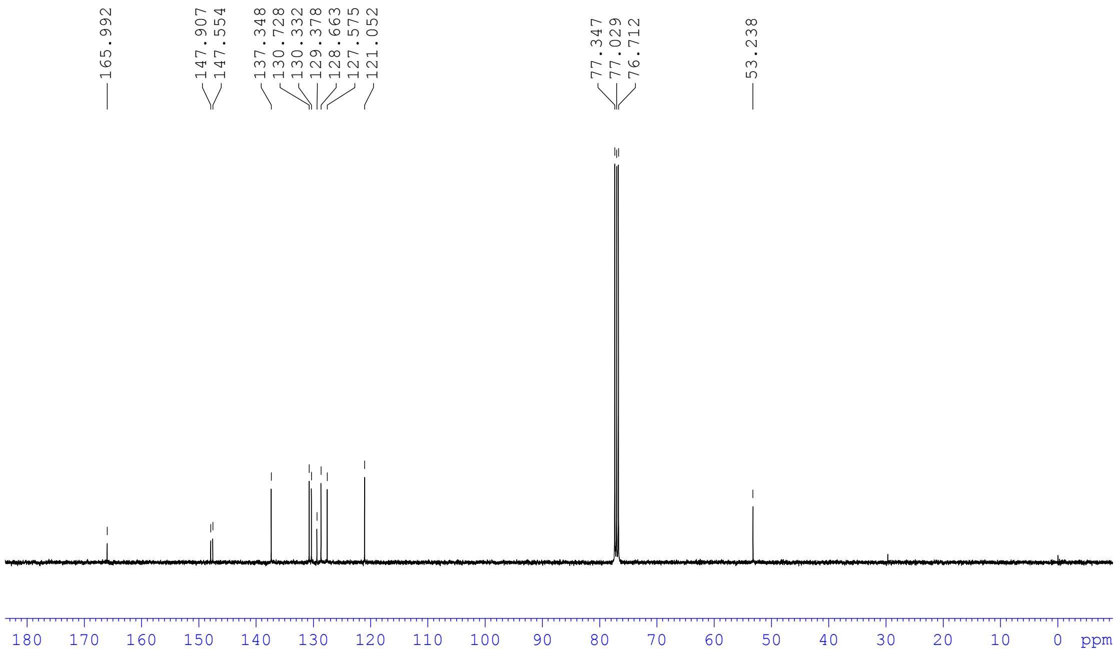
165.992
147.907
147.554
137.348
130.728
130.332
129.378
128.663
127.575
121.052
77.347
77.029
76.712
53.238
180
170
160
150
140
130
120
110
100
90
80
70
60
50
40
30
20
10
0 ppm

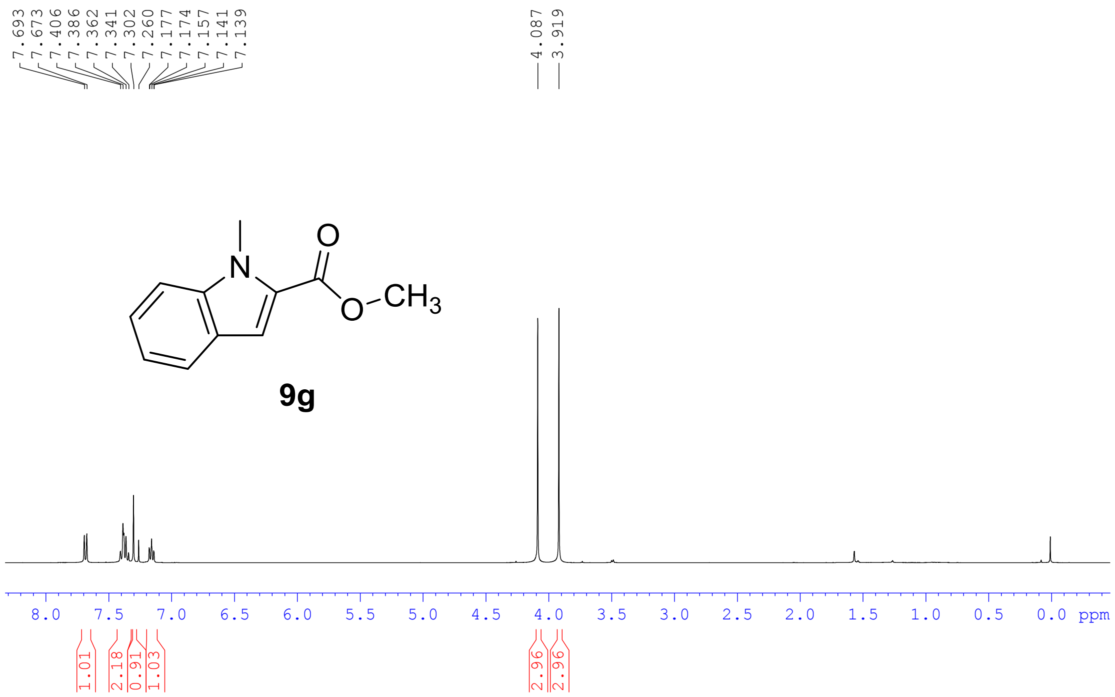
7.693 7.673 7.406 7.386 7.362 7.341 7.302 7.260 7.177 7.174 7.157 7.141 7.139
4.087 3.919
N
O
O
CH3
9g
8.0 7.5 7.0 6.5 6.0 5.5 5.0 4.5 4.0 3.5 3.0 2.5 2.0 1.5 1.0 0.5 0.0 ppm
1.01 2.18 0.91 1.03 2.96 2.96

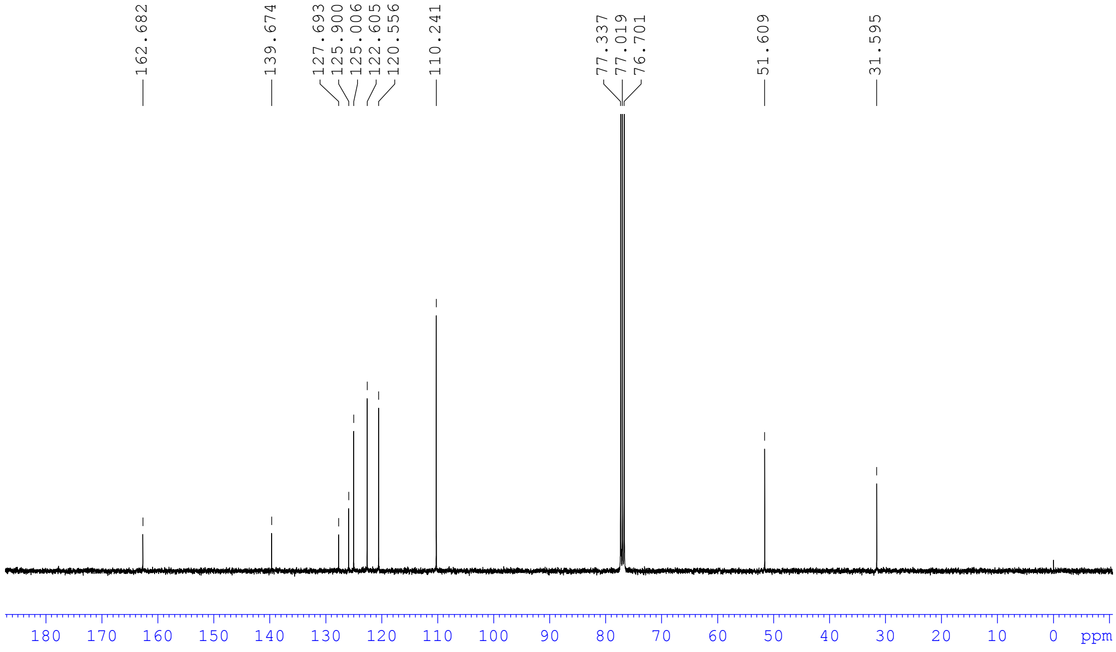
162.682 139.674 127.693 125.900 125.006 122.605 120.556 110.241 77.337 77.019 76.701 51.609 31.595
180 170 160 150 140 130 120 110 100 90 80 70 60 50 40 30 20 10 0 ppm

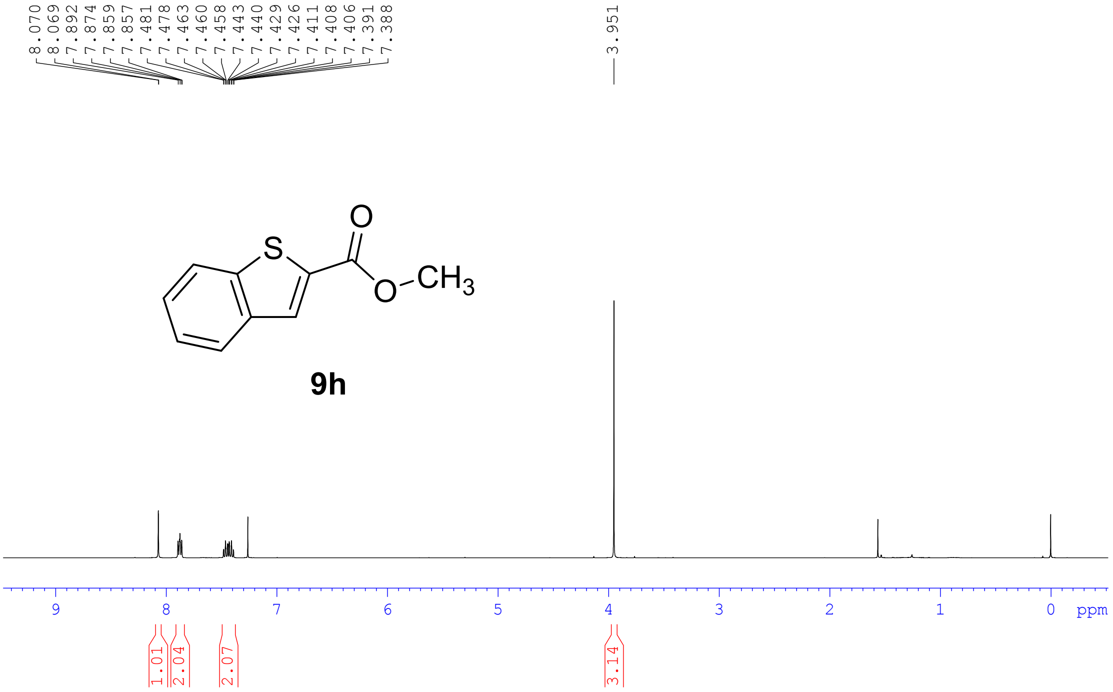
8.070
8.069
7.892
7.874
7.859
7.857
7.481
7.478
7.463
7.460
7.458
7.443
7.440
7.429
7.426
7.411
7.408
7.406
7.391
7.388
3.951
S
O
O
CH3
9h
9 8 7 6 5 4 3 2 1 0 ppm
1.01
2.04
2.07
3.14

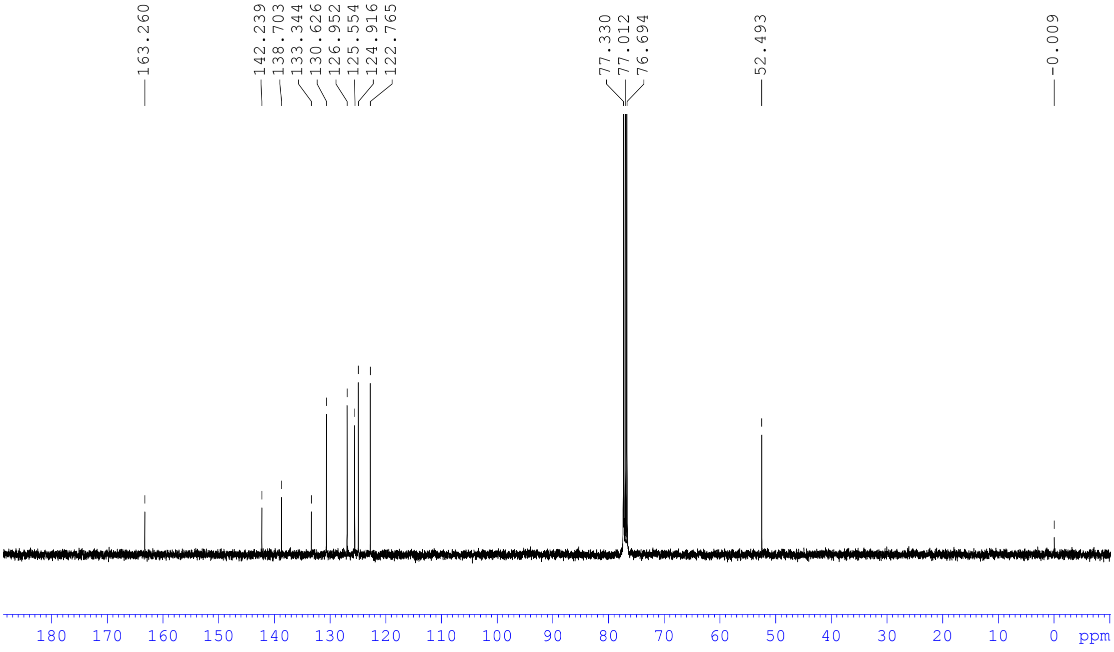
163.260
142.239
138.703
133.344
130.626
126.952
125.554
124.916
122.765
77.330
77.012
76.694
52.493
-0.009
180 170 160 150 140 130 120 110 100 90 80 70 60 50 40 30 20 10 0 ppm

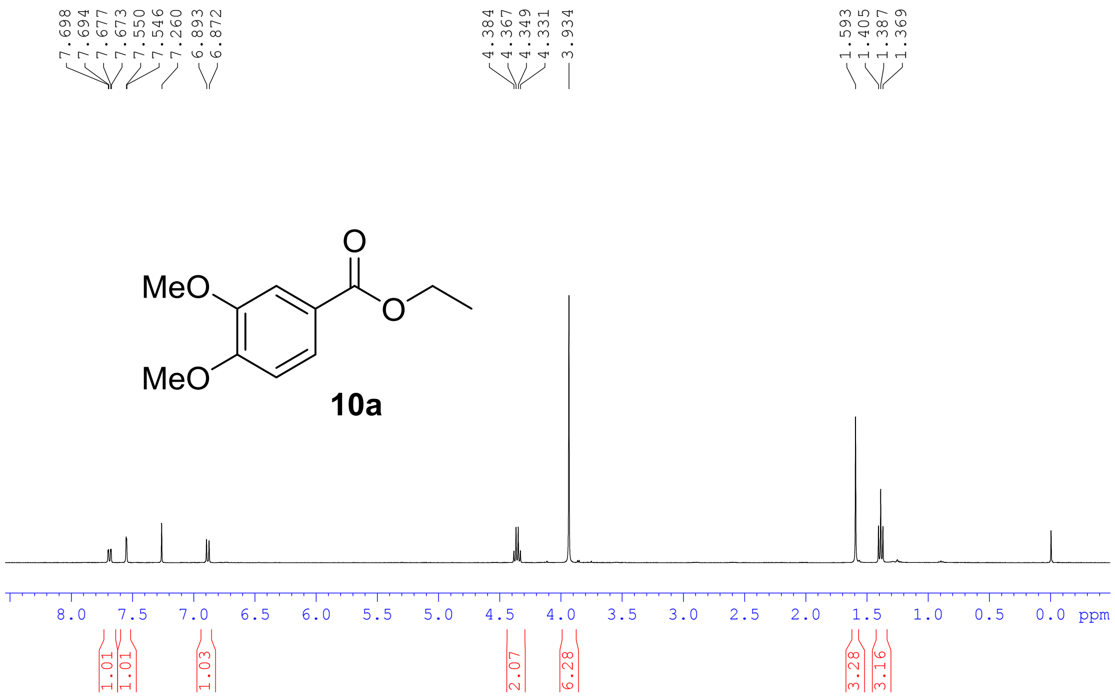
7.698
7.694
7.677
7.673
7.550
7.546
7.260
6.893
6.872
4.384
4.367
4.349
4.331
3.934
1.593
1.405
1.387
1.369
MeO
MeO
O
O
10a
8.0 7.5 7.0 6.5 6.0 5.5 5.0 4.5 4.0 3.5 3.0 2.5 2.0 1.5 1.0 0.5 0.0 ppm
1.01
1.01
1.03
2.07
6.28
3.28
3.16

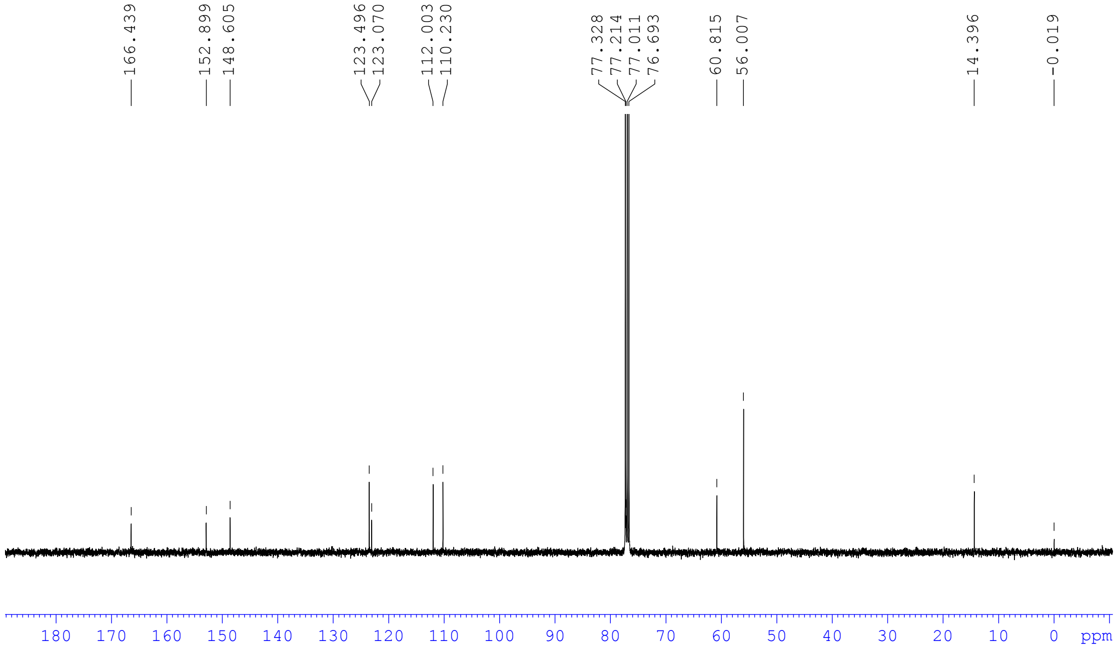
166.439
152.899
148.605
123.496
123.070
112.003
110.230
77.328
77.214
77.011
76.693
60.815
56.007
14.396
-0.019
180 170 160 150 140 130 120 110 100 90 80 70 60 50 40 30 20 10 0 ppm

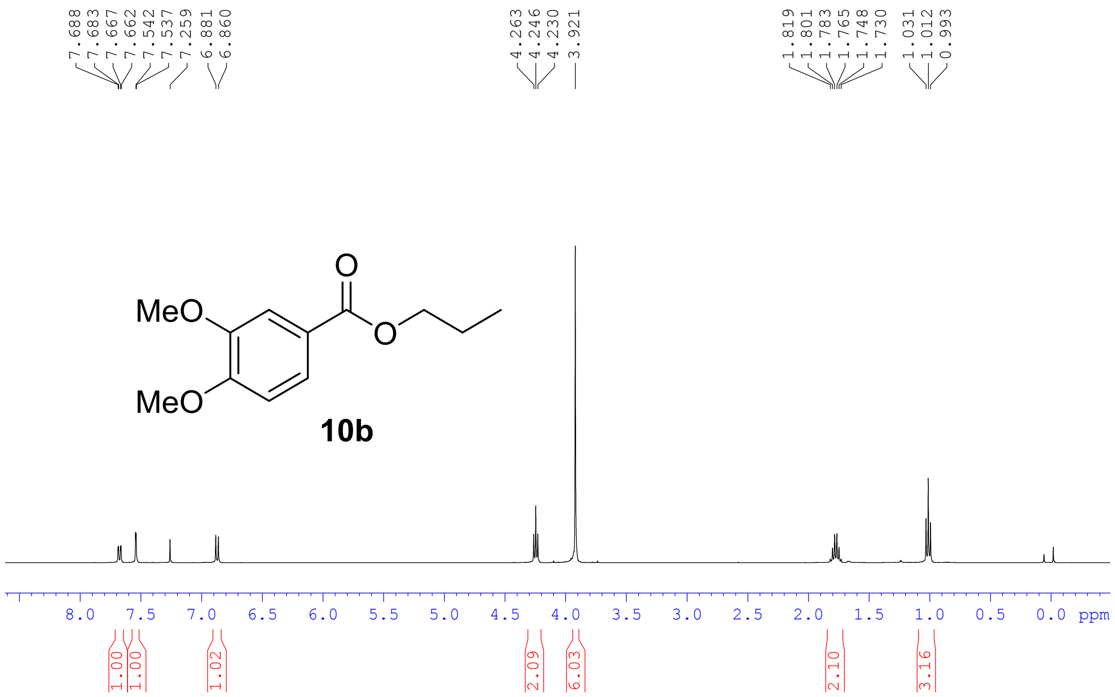
7.688
7.683
7.667
7.662
7.542
7.537
7.259
6.881
6.860
4.263
4.246
4.230
3.921
1.819
1.801
1.783
1.765
1.748
1.730
1.031
1.012
0.993
MeO
MeO
O
O
10b
8.0 7.5 7.0 6.5 6.0 5.5 5.0 4.5 4.0 3.5 3.0 2.5 2.0 1.5 1.0 0.5 0.0 ppm
1.00
1.00
1.02
2.09
6.03
2.10
3.16

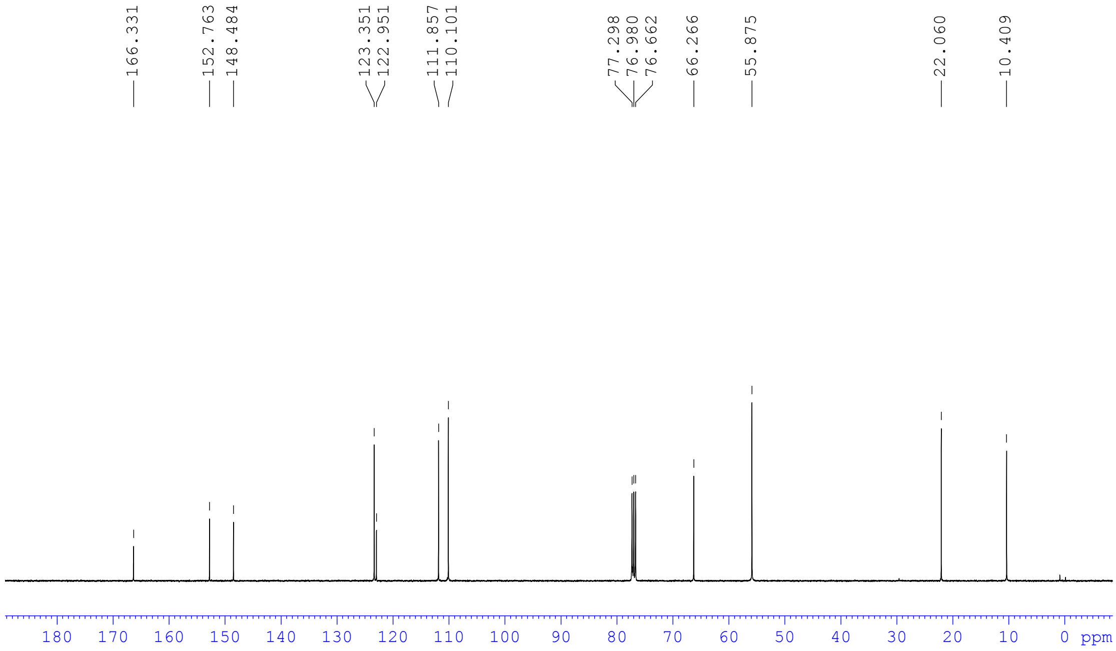
166.331
152.763
148.484
123.351
122.951
111.857
110.101
77.298
76.980
76.662
66.266
55.875
22.060
10.409
180 170 160 150 140 130 120 110 100 90 80 70 60 50 40 30 20 10 0 ppm

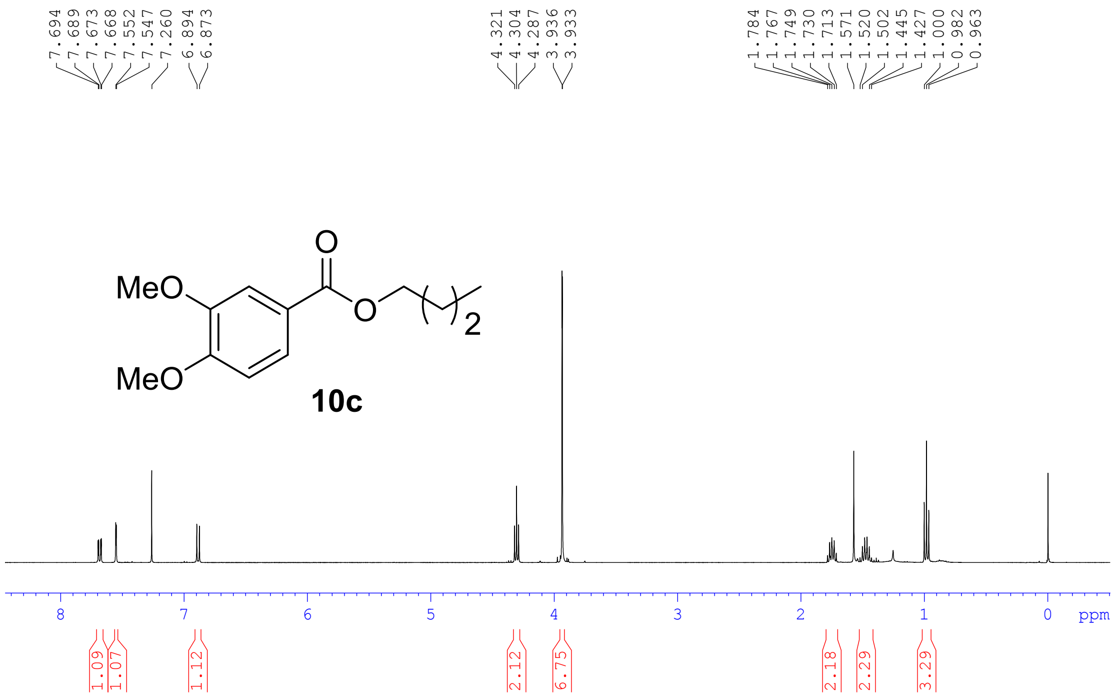
7.694
7.689
7.673
7.668
7.552
7.547
7.260
6.894
6.873
4.321
4.304
4.287
3.936
3.933
1.784
1.767
1.749
1.730
1.713
1.571
1.520
1.502
1.445
1.427
1.000
0.982
0.963
MeO
MeO
O
O
2
10c
8
7
6
5
4
3
2
1
0
ppm
1.09
1.07
1.12
2.12
6.75
2.18
2.29
3.29

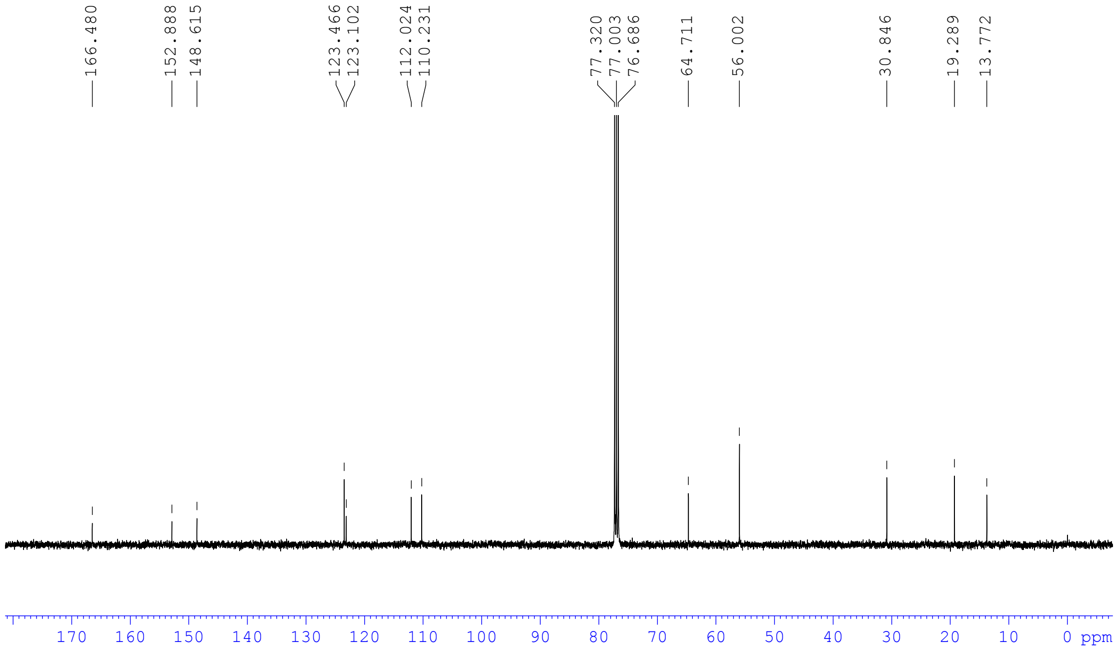
166.480
152.888
148.615
123.466
123.102
112.024
110.231
77.320
77.003
76.686
64.711
56.002
30.846
19.289
13.772
170
160
150
140
130
120
110
100
90
80
70
60
50
40
30
20
10
0 ppm

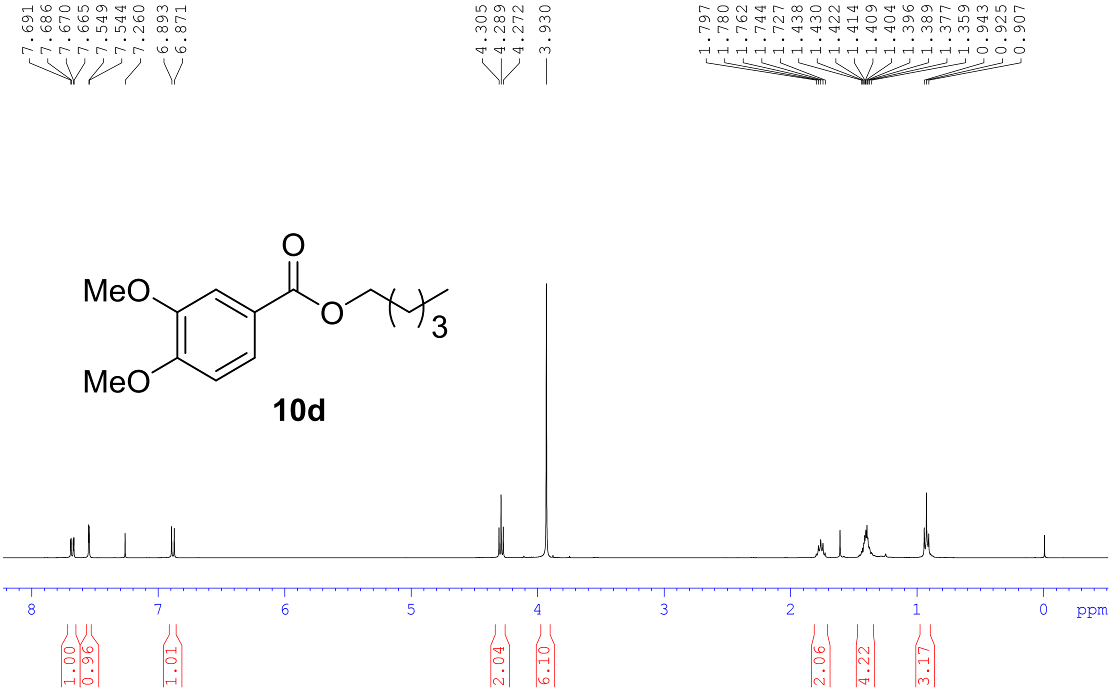

MeO
MeO
O
O
3
7.691
7.686
7.670
7.665
7.549
7.544
7.260
6.893
6.871
4.305
4.289
4.272
3.930
1.797
1.780
1.762
1.744
1.727
1.438
1.430
1.422
1.414
1.409
1.404
1.396
1.389
1.377
1.359
0.943
0.925
0.907
8 7 6 5 4 3 2 1 0 ppm
1.00
0.96
1.01
2.04
6.10
2.06
4.22
3.17

10d

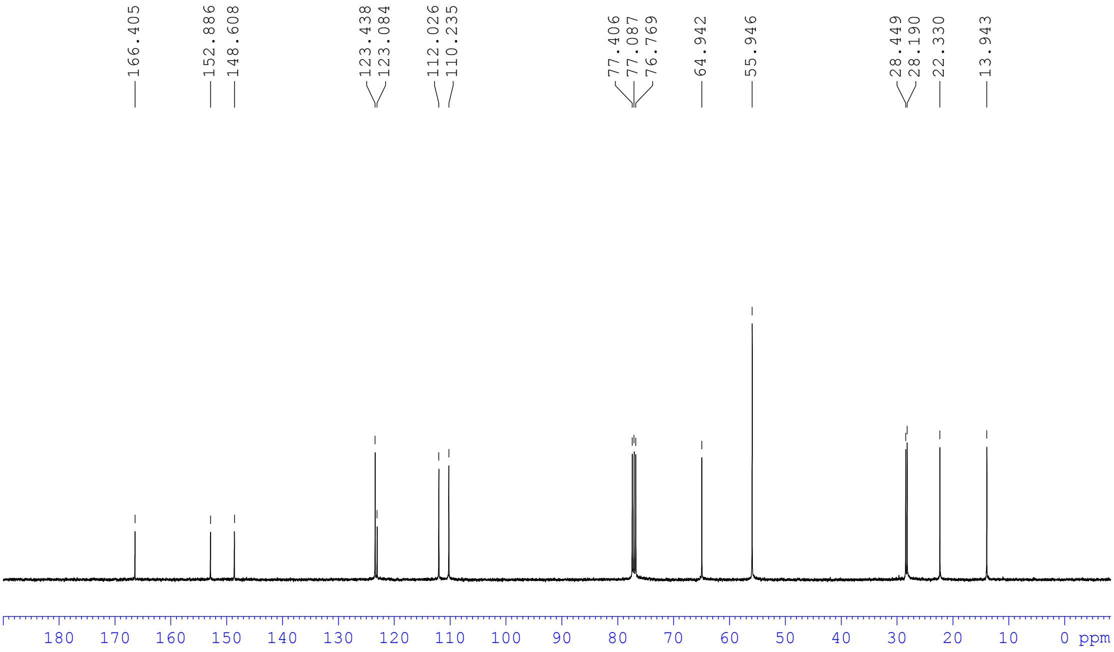

166.405
152.886
148.608
123.438
123.084
112.026
110.235
77.406
77.087
76.769
64.942
55.946
28.449
28.190
22.330
13.943
180 170 160 150 140 130 120 110 100 90 80 70 60 50 40 30 20 10 0 ppm

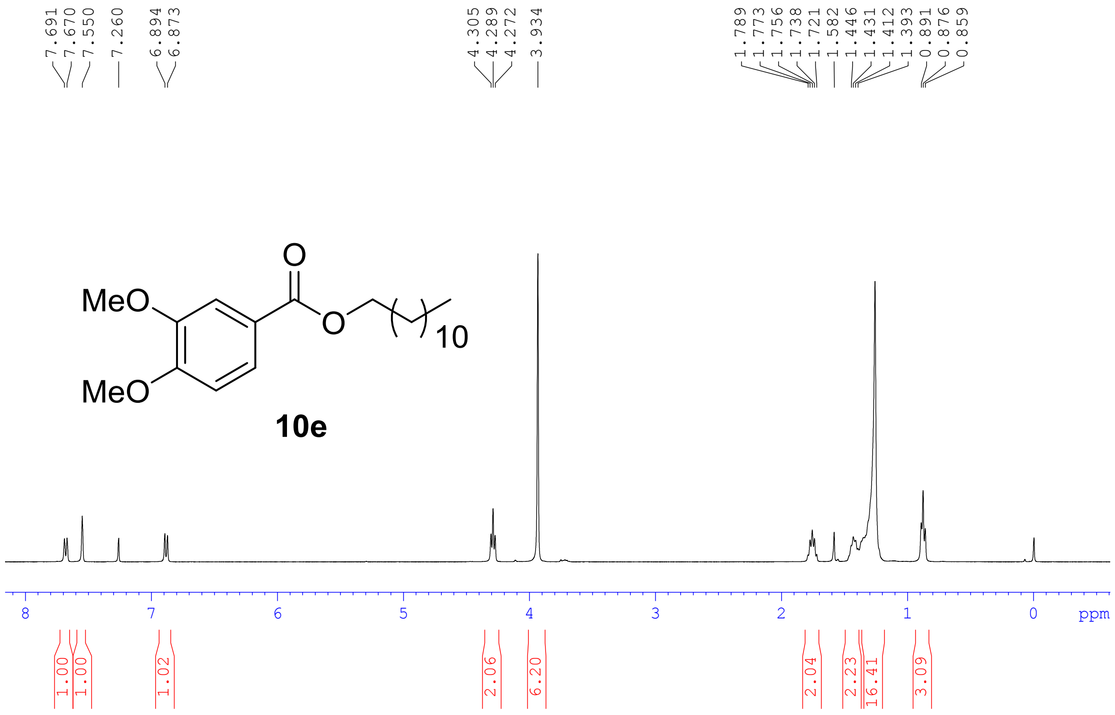
7.691
7.670
7.550
7.260
6.894
6.873
4.305
4.289
4.272
3.934
1.789
1.773
1.756
1.738
1.721
1.582
1.446
1.431
1.412
1.393
0.891
0.876
0.859
MeO
MeO
O
O
10
10e
8
7
6
5
4
3
2
1
0
ppm
1.00
1.00
1.02
2.06
6.20
2.04
2.23
16.41
3.09

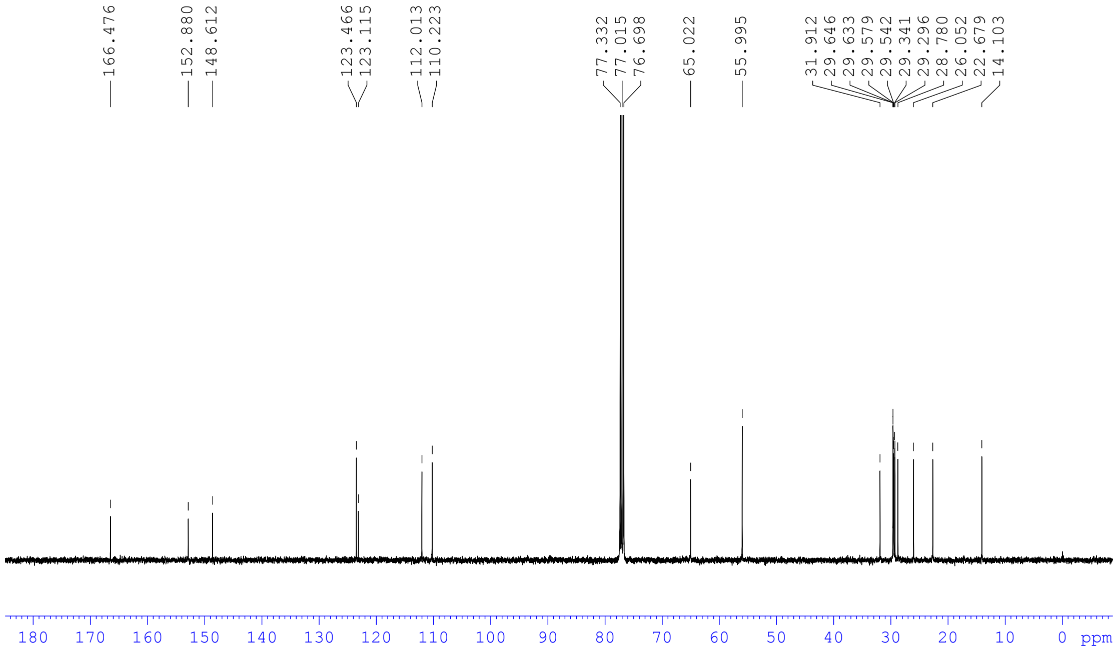
166.476
152.880
148.612
123.466
123.115
112.013
110.223
77.332
77.015
76.698
65.022
55.995
31.912
29.646
29.633
29.579
29.542
29.341
29.296
28.780
26.052
22.679
14.103
180
170
160
150
140
130
120
110
100
90
80
70
60
50
40
30
20
10
0
ppm

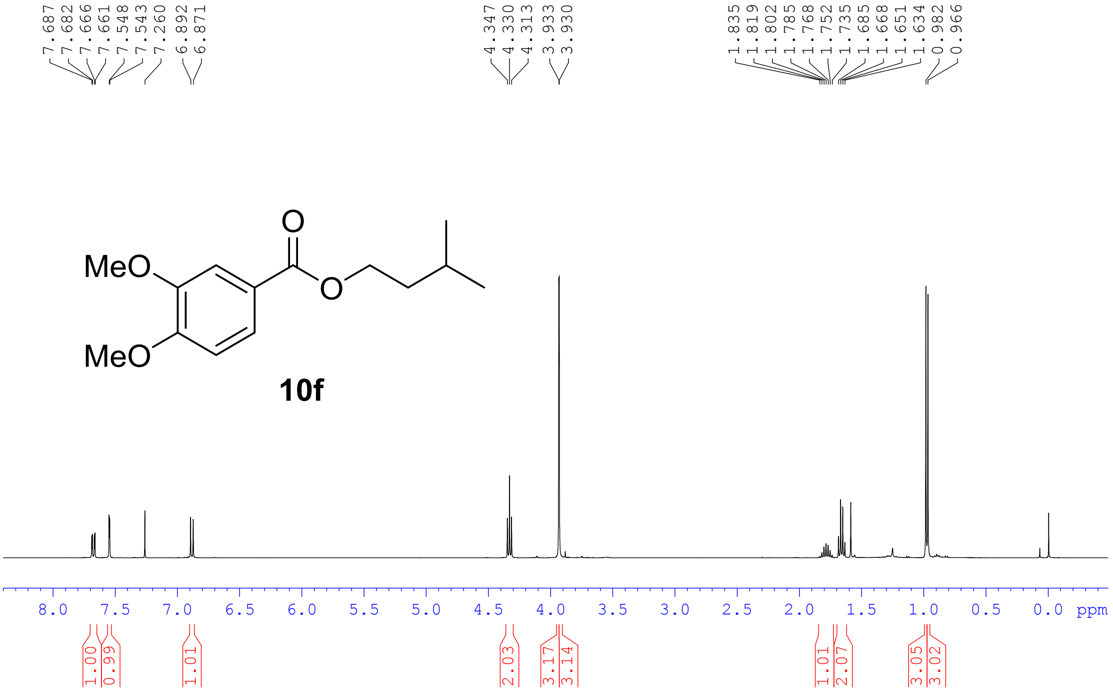
7.687
7.682
7.666
7.661
7.548
7.543
7.260
6.892
6.871
4.347
4.330
4.313
3.933
3.930
1.835
1.819
1.802
1.785
1.768
1.752
1.735
1.685
1.668
1.651
1.634
0.982
0.966
MeO
MeO
O
O
10f
8.0
7.5
7.0
6.5
6.0
5.5
5.0
4.5
4.0
3.5
3.0
2.5
2.0
1.5
1.0
0.5
0.0
ppm
1.00
0.99
1.01
2.03
3.17
3.14
1.01
2.07
3.05
3.02

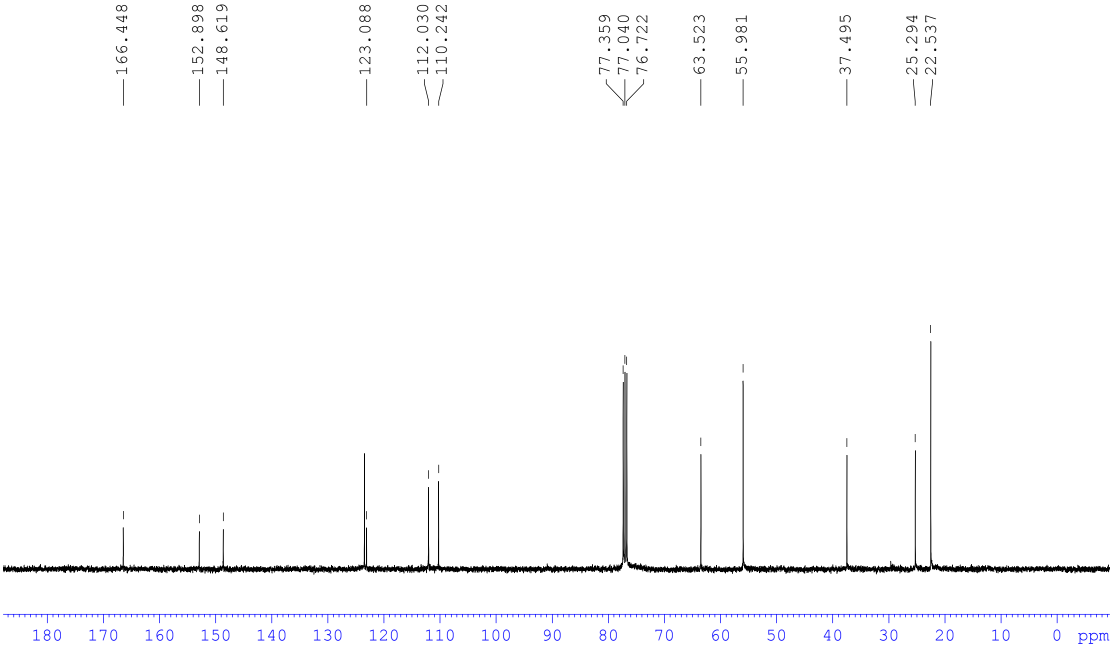
166.448
152.898
148.619
123.088
112.030
110.242
77.359
77.040
76.722
63.523
55.981
37.495
25.294
22.537
180
170
160
150
140
130
120
110
100
90
80
70
60
50
40
30
20
10
0
ppm

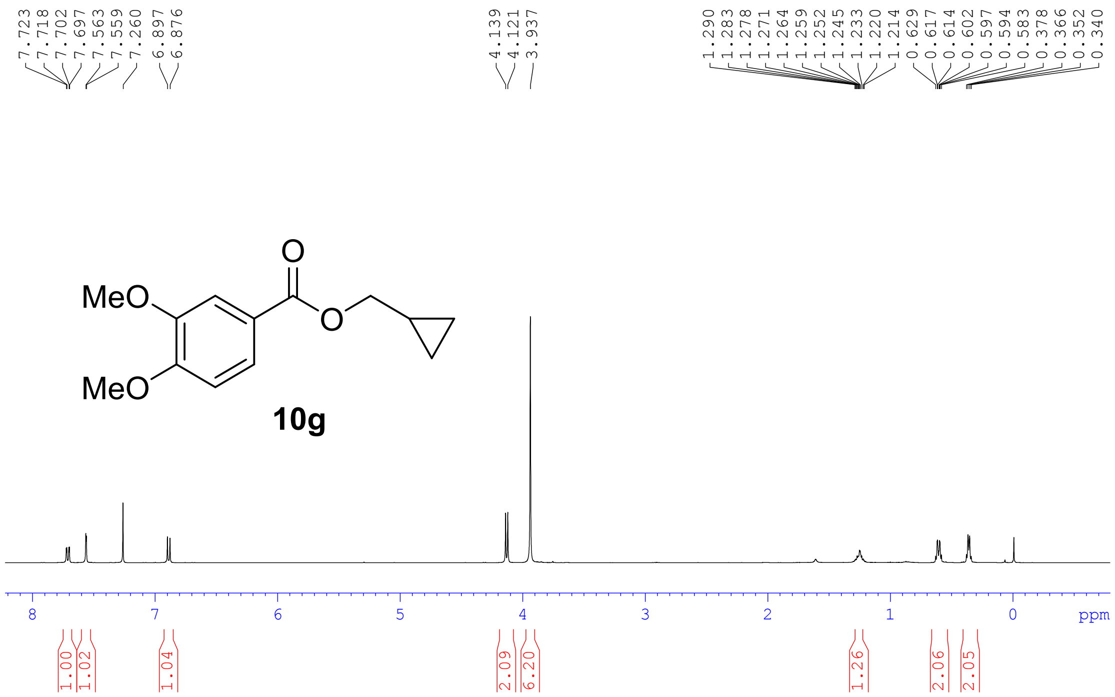

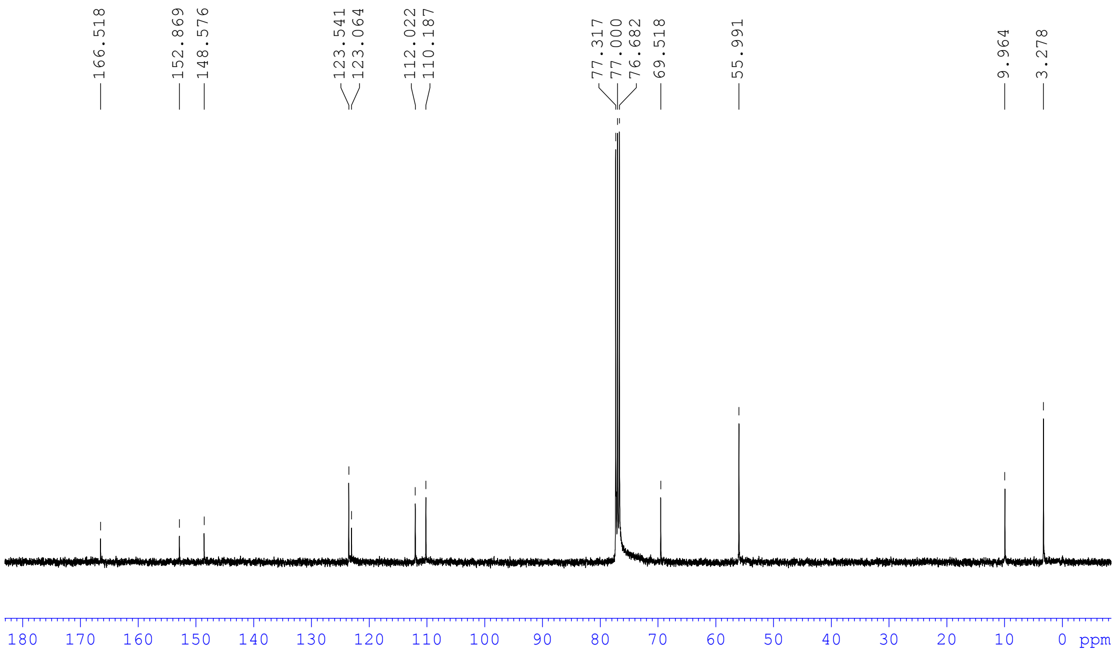

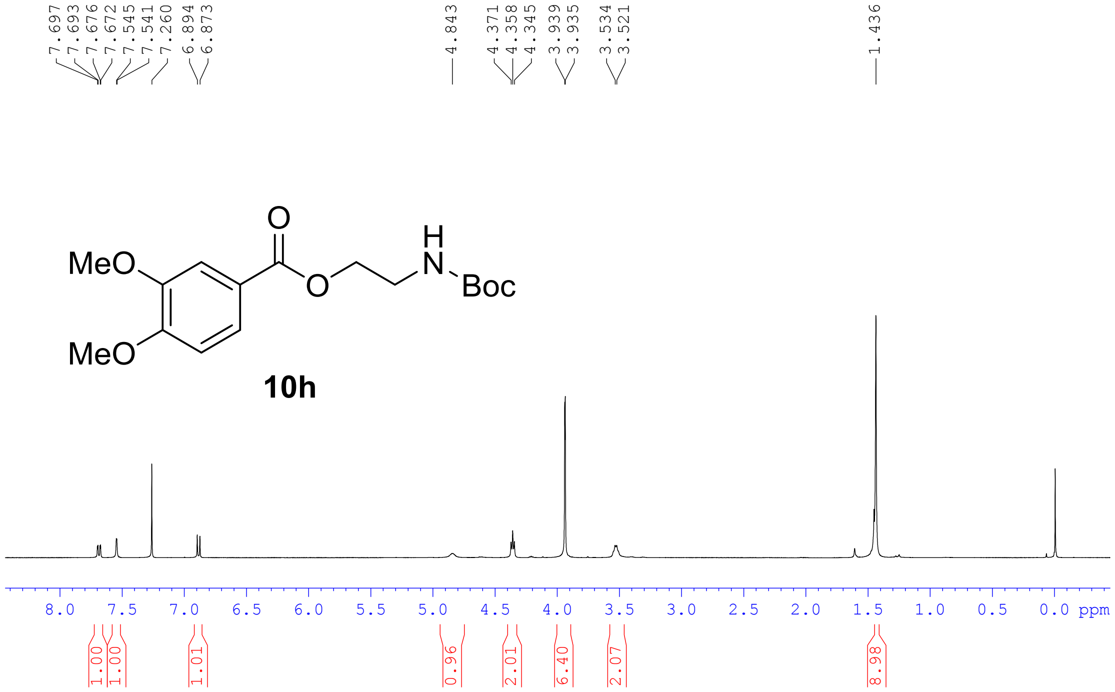
7.697
7.693
7.676
7.672
7.545
7.541
7.260
6.894
6.873
4.843
4.371
4.358
4.345
3.939
3.935
3.534
3.521
1.436
MeO
MeO
O
O
H
N
Boc
10h
8.0 7.5 7.0 6.5 6.0 5.5 5.0 4.5 4.0 3.5 3.0 2.5 2.0 1.5 1.0 0.5 0.0 ppm
1.00
1.00
1.01
0.96
2.01
6.40
2.07
8.98

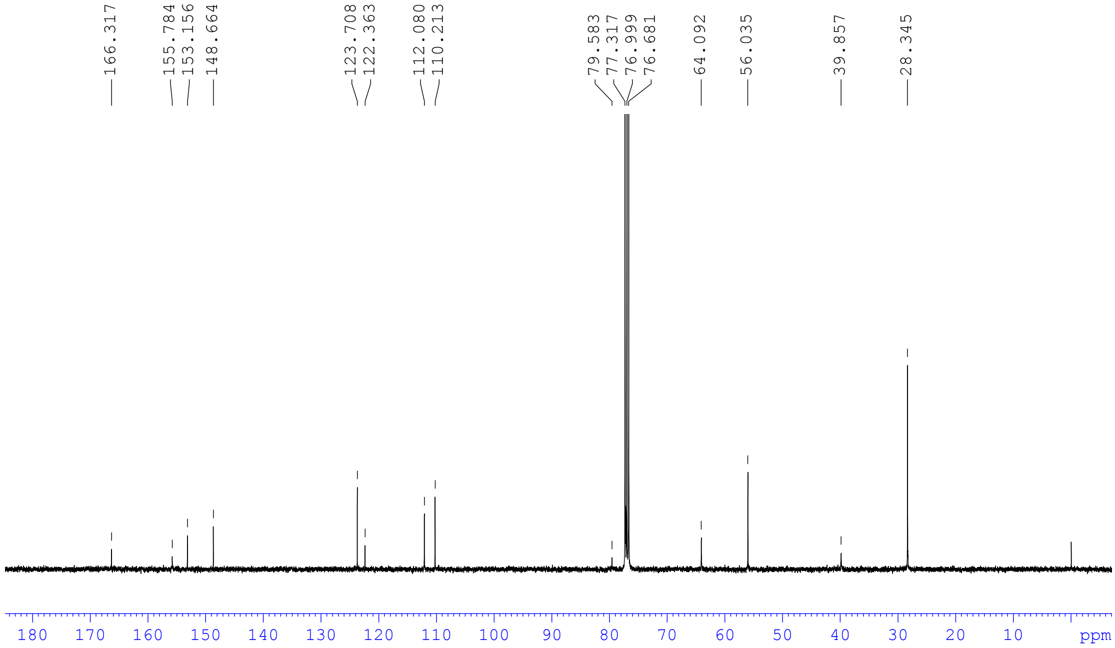
166.317
155.784
153.156
148.664
123.708
122.363
112.080
110.213
79.583
77.317
76.999
76.681
64.092
56.035
39.857
28.345
180 170 160 150 140 130 120 110 100 90 80 70 60 50 40 30 20 10 ppm

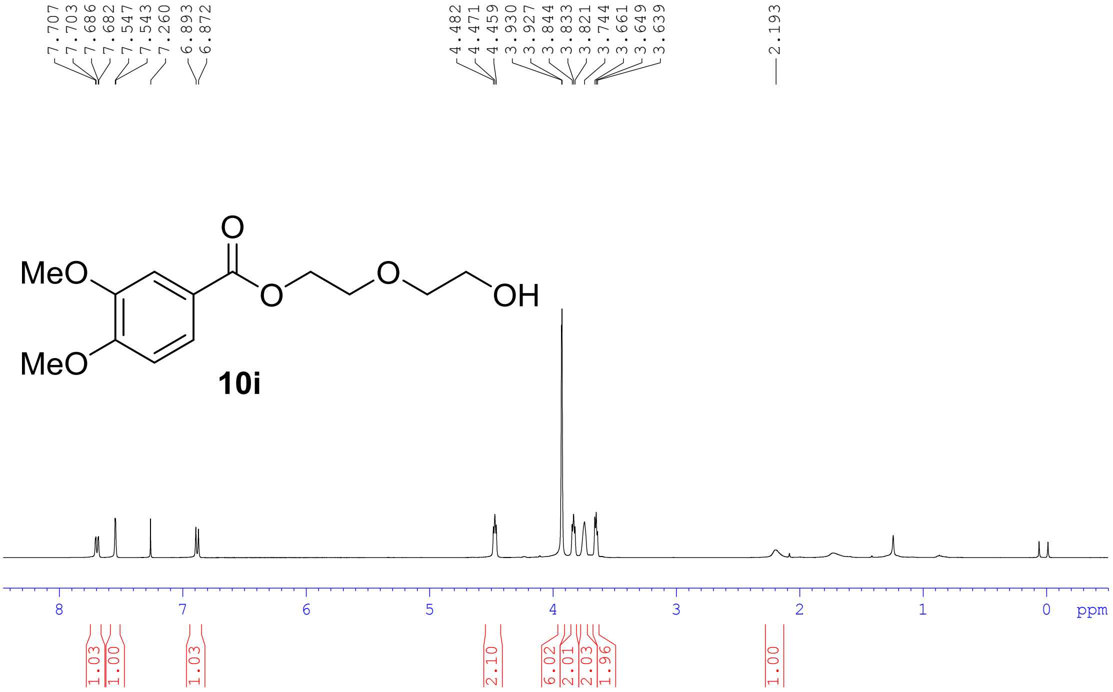
7.707
7.703
7.686
7.682
7.547
7.543
7.260
6.893
6.872
4.482
4.471
4.459
3.930
3.927
3.844
3.833
3.821
3.744
3.661
3.649
3.639
2.193
MeO
MeO
O
O
O
OH
10i
8
7
6
5
4
3
2
1
0
ppm
1.03
1.00
1.03
2.10
6.02
2.01
2.03
1.96
1.00

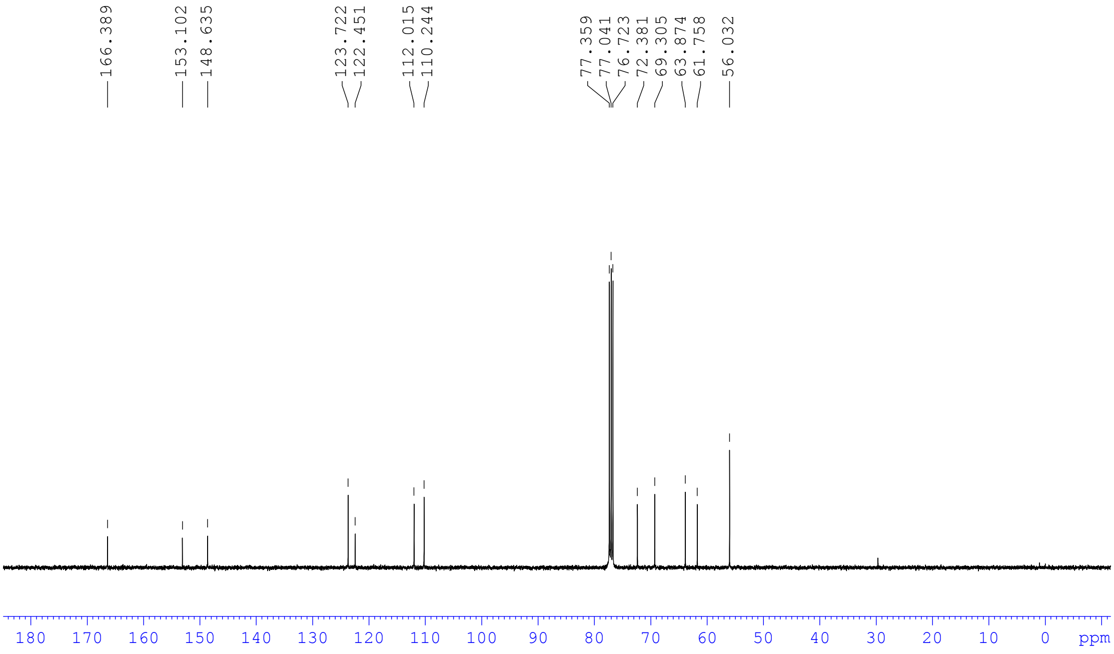
166.389
153.102
148.635
123.722
122.451
112.015
110.244
77.359
77.041
76.723
72.381
69.305
63.874
61.758
56.032
180
170
160
150
140
130
120
110
100
90
80
70
60
50
40
30
20
10
0
ppm

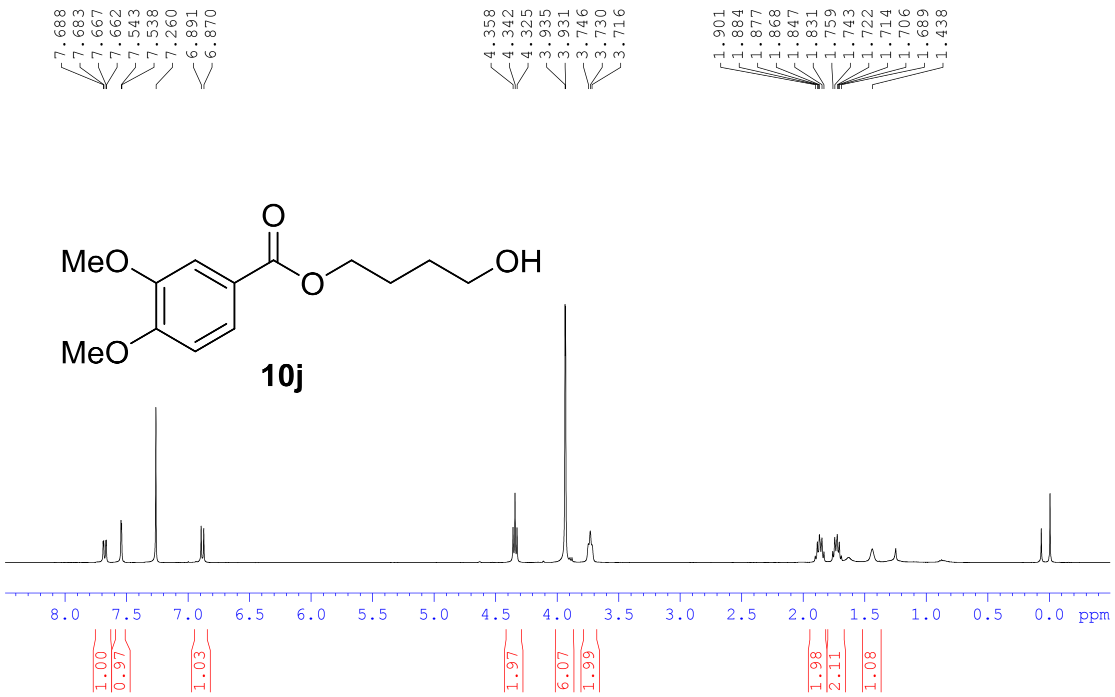
7.688
7.683
7.667
7.662
7.543
7.538
7.260
6.891
6.870
4.358
4.342
4.325
3.935
3.931
3.746
3.730
3.716
1.901
1.884
1.877
1.868
1.847
1.831
1.759
1.743
1.722
1.714
1.706
1.689
1.438
MeO
MeO
O
O
OH
10j
8.0 7.5 7.0 6.5 6.0 5.5 5.0 4.5 4.0 3.5 3.0 2.5 2.0 1.5 1.0 0.5 0.0 ppm
1.00
0.97
1.03
1.97
6.07
1.99
1.98
2.11
1.08

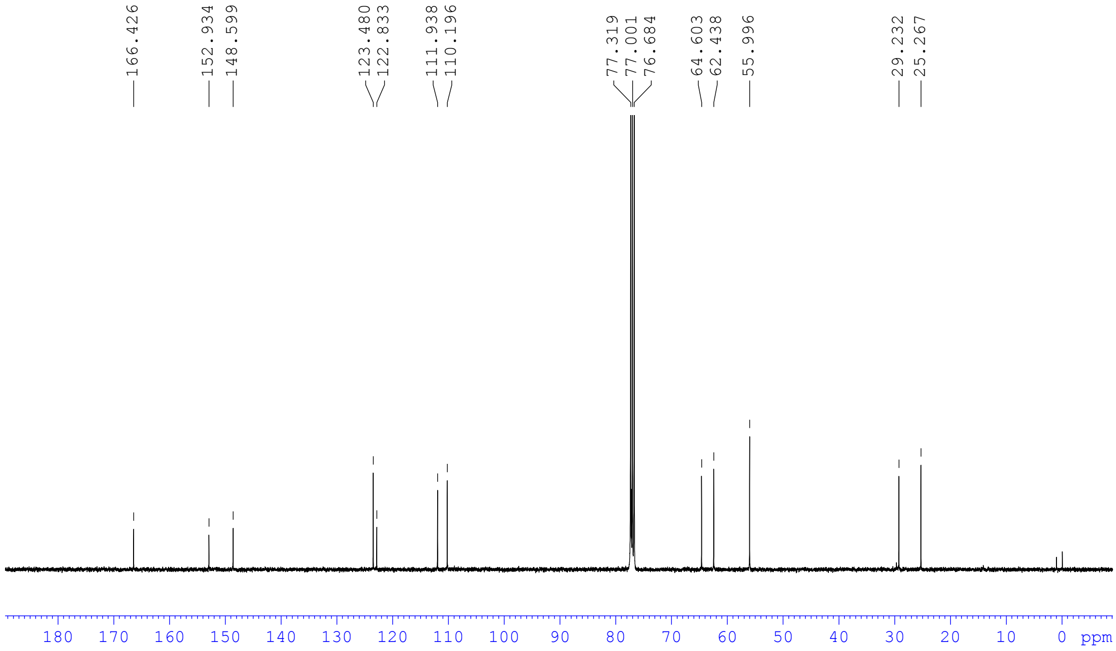
166.426
152.934
148.599
123.480
122.833
111.938
110.196
77.319
77.001
76.684
64.603
62.438
55.996
29.232
25.267
180 170 160 150 140 130 120 110 100 90 80 70 60 50 40 30 20 10 0 ppm

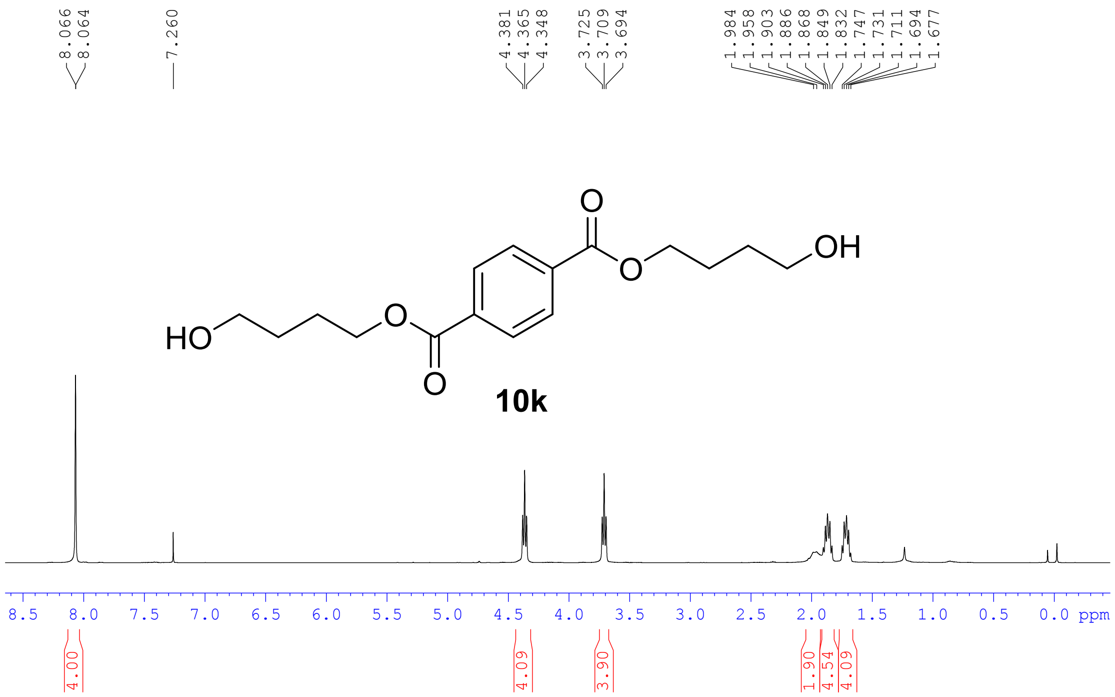
8.066
8.064
7.260
4.381
4.365
4.348
3.725
3.709
3.694
1.984
1.958
1.903
1.886
1.868
1.849
1.832
1.747
1.731
1.711
1.694
1.677
HO
O
O
O
O
OH
10k
8.5 8.0 7.5 7.0 6.5 6.0 5.5 5.0 4.5 4.0 3.5 3.0 2.5 2.0 1.5 1.0 0.5 0.0 ppm
4.00
4.09
3.90
1.90
4.54
4.09

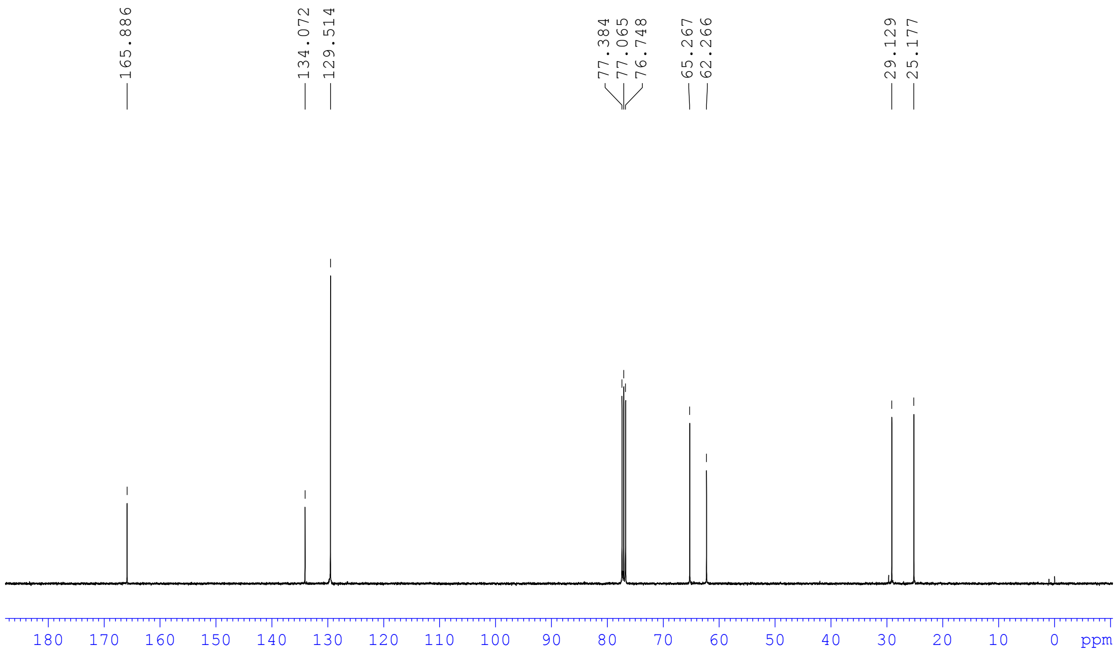
165.886
134.072
129.514
77.384
77.065
76.748
65.267
62.266
29.129
25.177
180 170 160 150 140 130 120 110 100 90 80 70 60 50 40 30 20 10 0 ppm

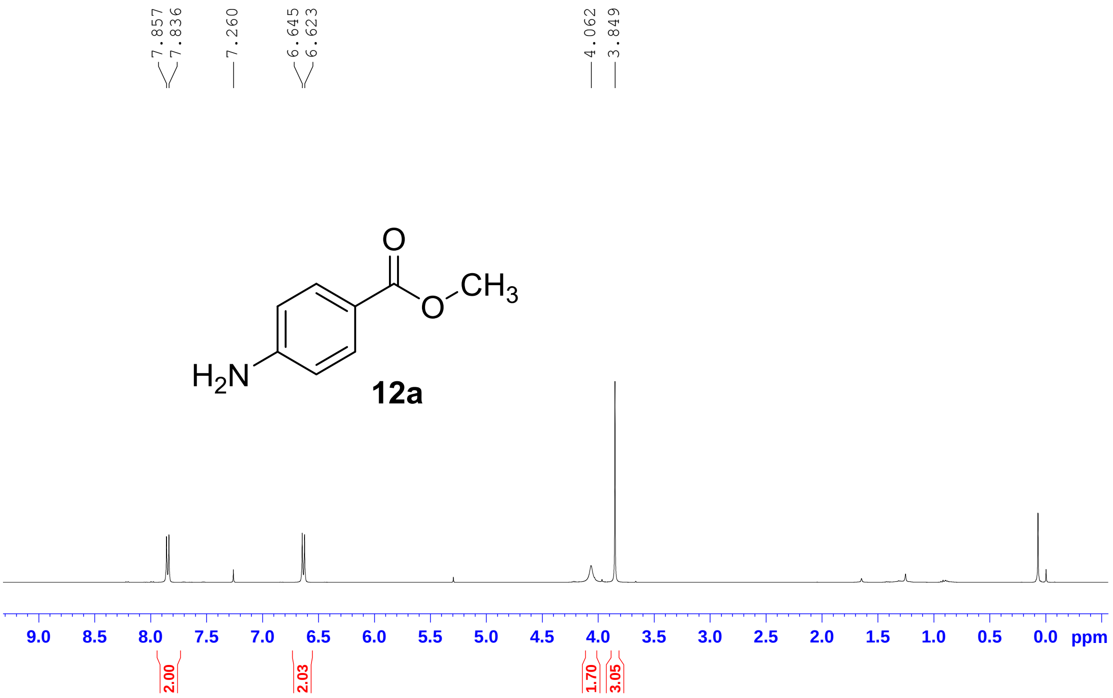

7.857
7.836
7.260
6.645
6.623
4.062
3.849
O
CH3
O
H2N
9.0 8.5 8.0 7.5 7.0 6.5 6.0 5.5 5.0 4.5 4.0 3.5 3.0 2.5 2.0 1.5 1.0 0.5 0.0 ppm
2.00
2.03
1.70
3.05

12a

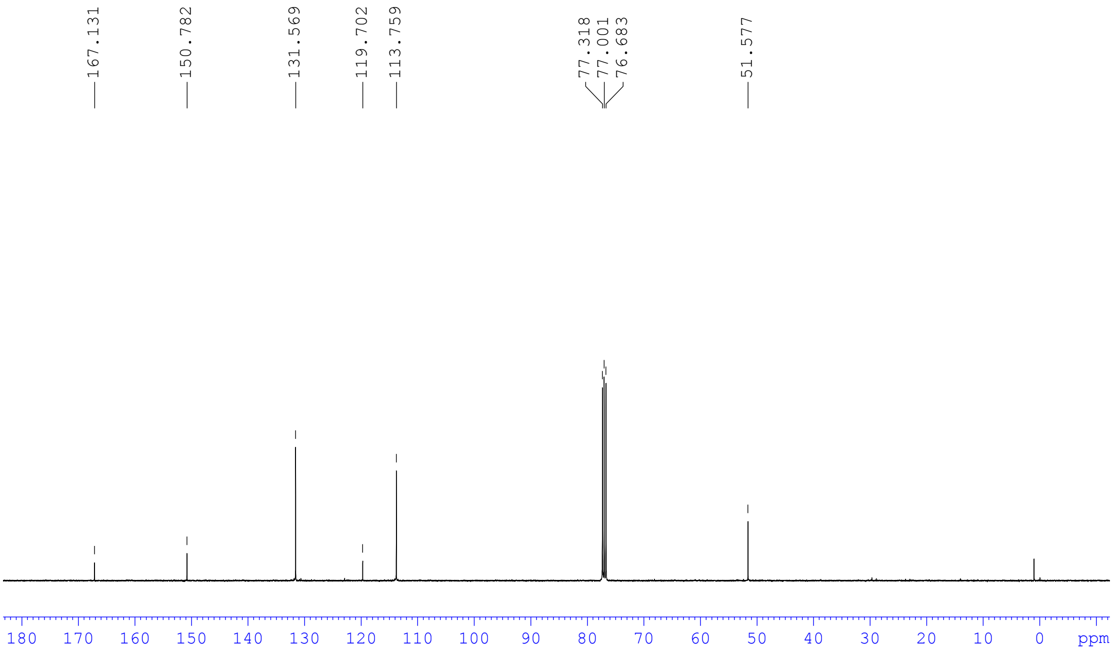

167.131
150.782
131.569
119.702
113.759
77.318
77.001
76.683
51.577
180 170 160 150 140 130 120 110 100 90 80 70 60 50 40 30 20 10 0 ppm

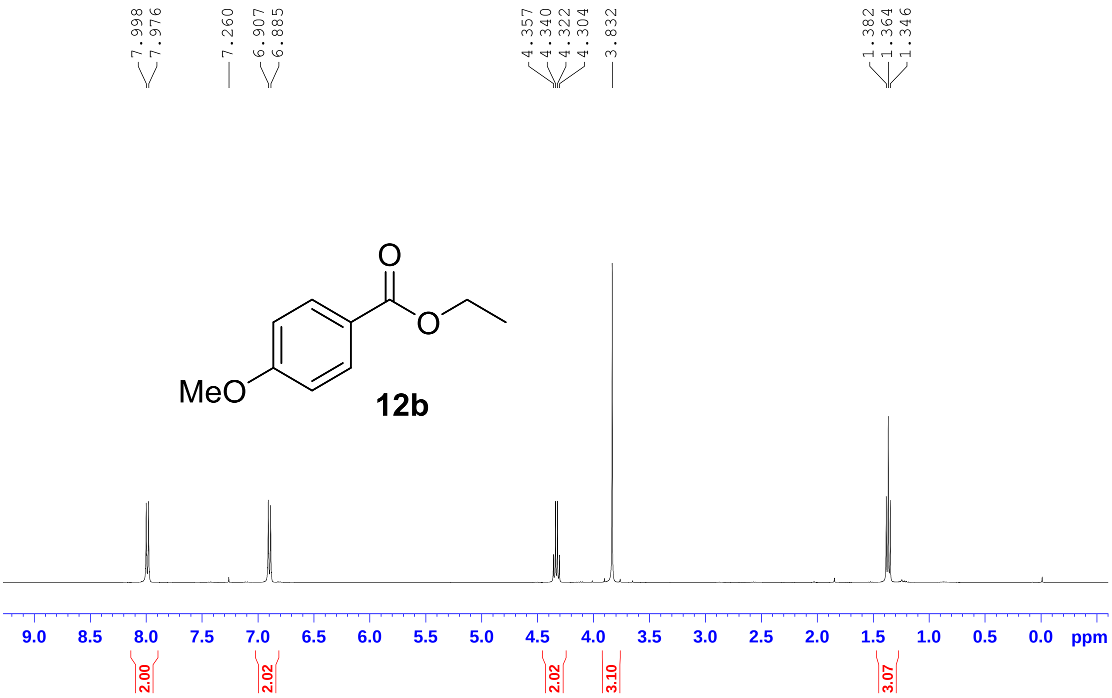
7.998
7.976
7.260
6.907
6.885
4.357
4.340
4.322
4.304
3.832
1.382
1.364
1.346
MeO
12b
9.0 8.5 8.0 7.5 7.0 6.5 6.0 5.5 5.0 4.5 4.0 3.5 3.0 2.5 2.0 1.5 1.0 0.5 0.0 ppm
2.00
2.02
2.02
3.10
3.07

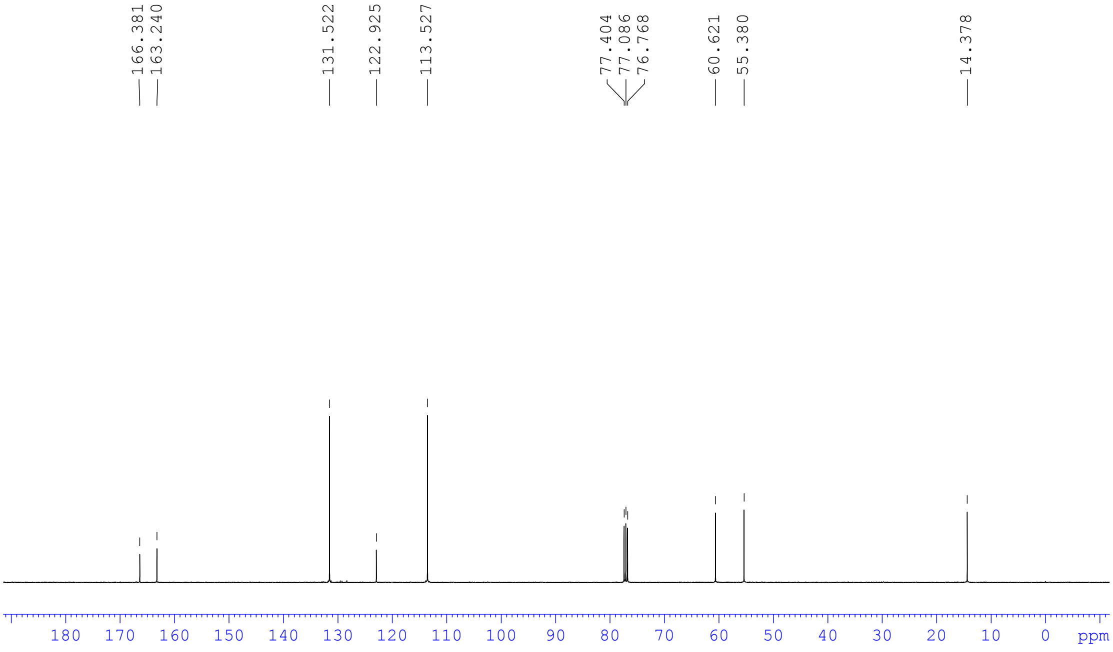
166.381
163.240
131.522
122.925
113.527
77.404
77.086
76.768
60.621
55.380
14.378
180 170 160 150 140 130 120 110 100 90 80 70 60 50 40 30 20 10 0 ppm

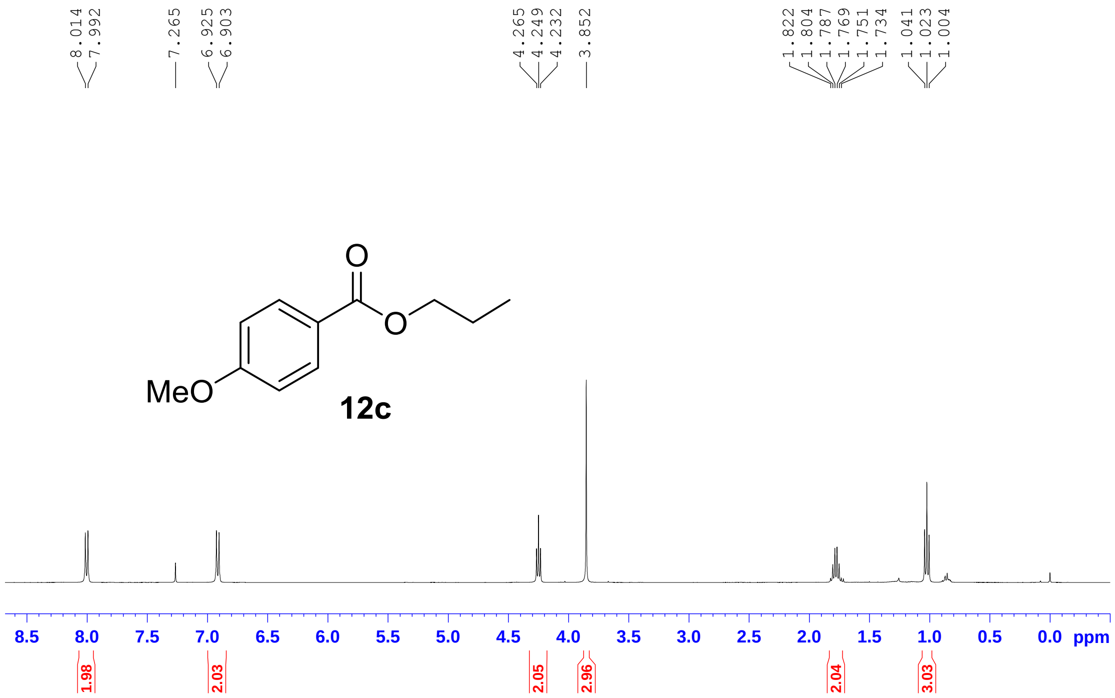
8.014
7.992
7.265
6.925
6.903
4.265
4.249
4.232
3.852
1.822
1.804
1.787
1.769
1.751
1.734
1.041
1.023
1.004
MeO
O
O
12c
8.5 8.0 7.5 7.0 6.5 6.0 5.5 5.0 4.5 4.0 3.5 3.0 2.5 2.0 1.5 1.0 0.5 0.0 ppm
1.98
2.03
2.05
2.96
2.04
3.03

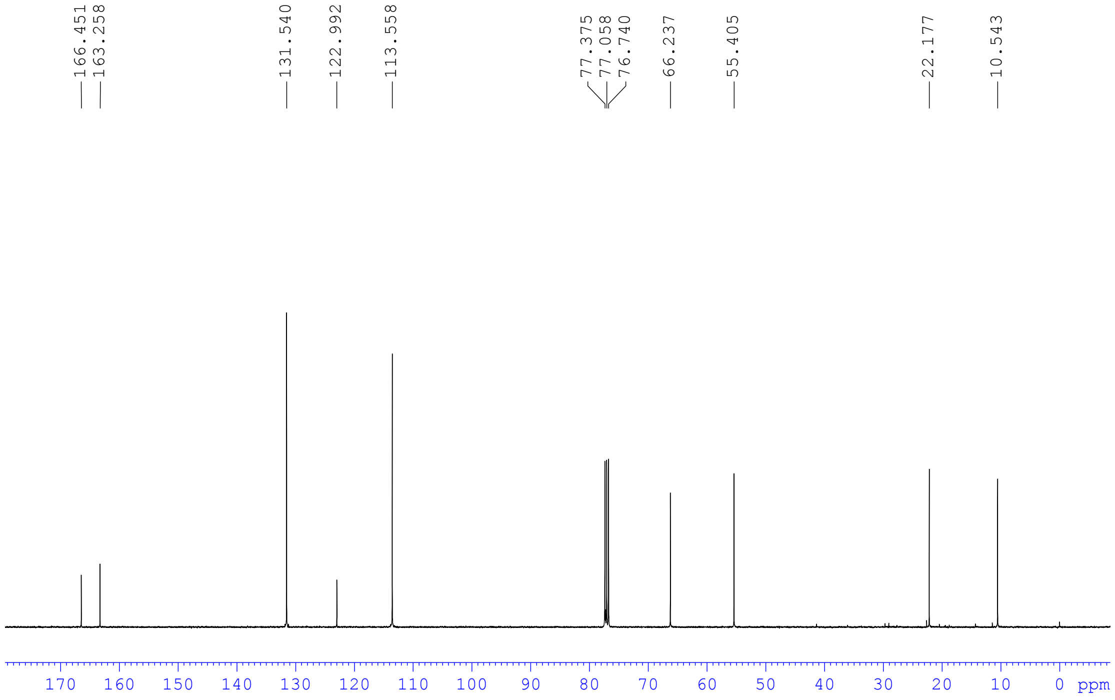
166.451
163.258
131.540
122.992
113.558
77.375
77.058
76.740
66.237
55.405
22.177
10.543
170 160 150 140 130 120 110 100 90 80 70 60 50 40 30 20 10 0 ppm

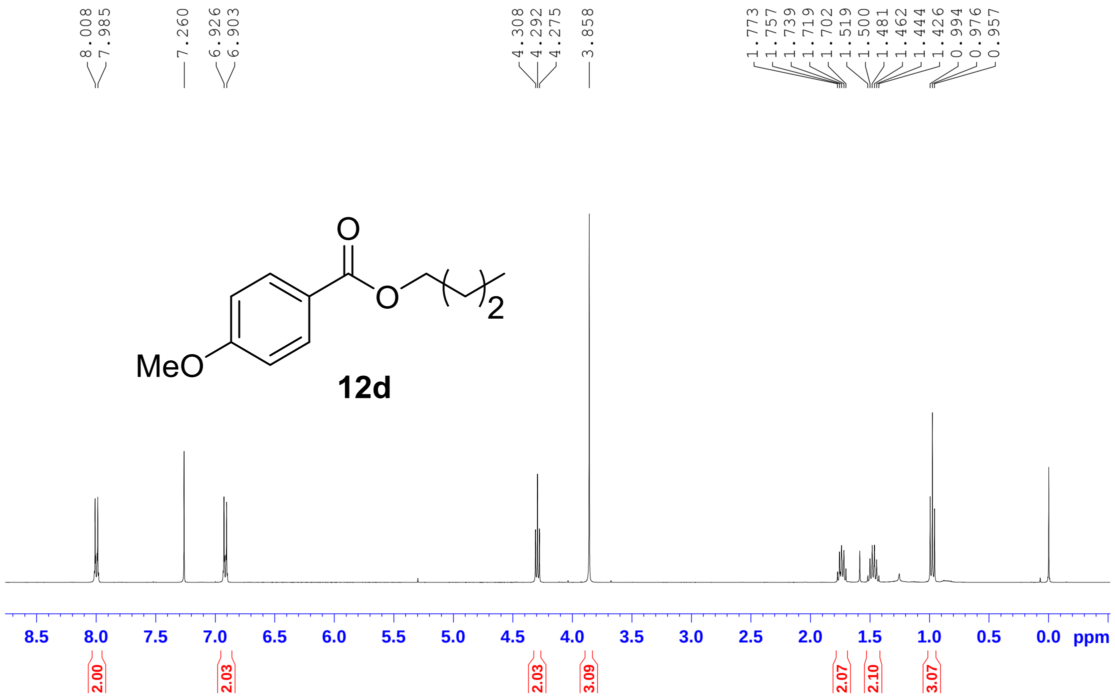
8.008
7.985
7.260
6.926
6.903
4.308
4.292
4.275
3.858
1.773
1.757
1.739
1.719
1.702
1.519
1.500
1.481
1.462
1.444
1.426
0.994
0.976
0.957
O
O
2
MeO
12d
8.5 8.0 7.5 7.0 6.5 6.0 5.5 5.0 4.5 4.0 3.5 3.0 2.5 2.0 1.5 1.0 0.5 0.0 ppm
2.00
2.03
2.03
3.09
2.07
2.10
3.07

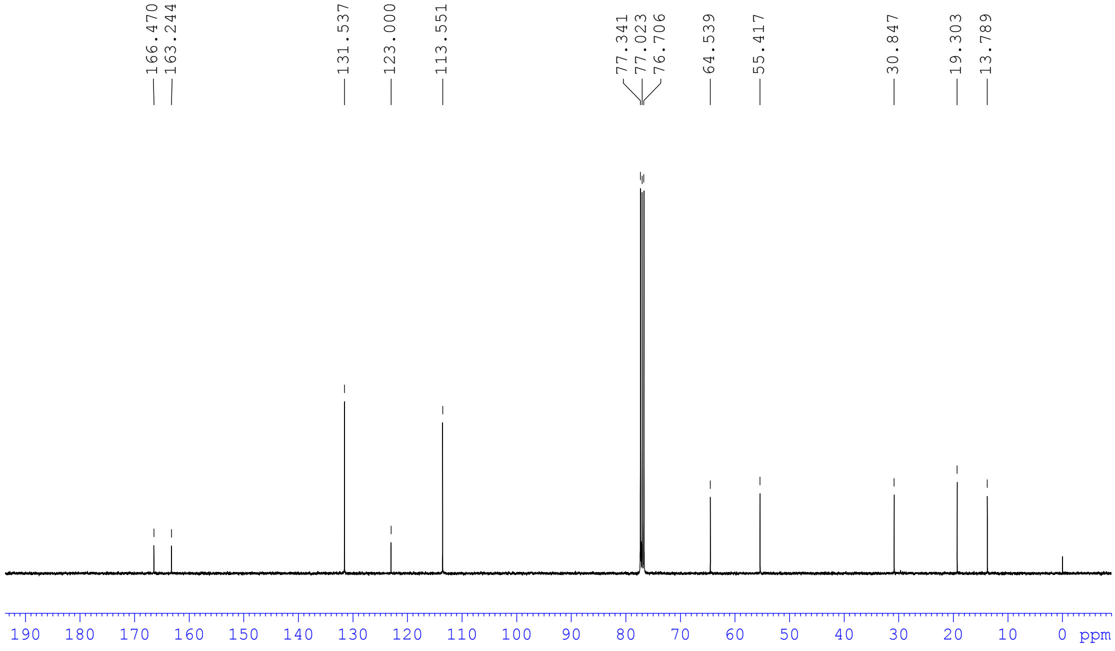
166.470
163.244
131.537
123.000
113.551
77.341
77.023
76.706
64.539
55.417
30.847
19.303
13.789
190 180 170 160 150 140 130 120 110 100 90 80 70 60 50 40 30 20 10 0 ppm

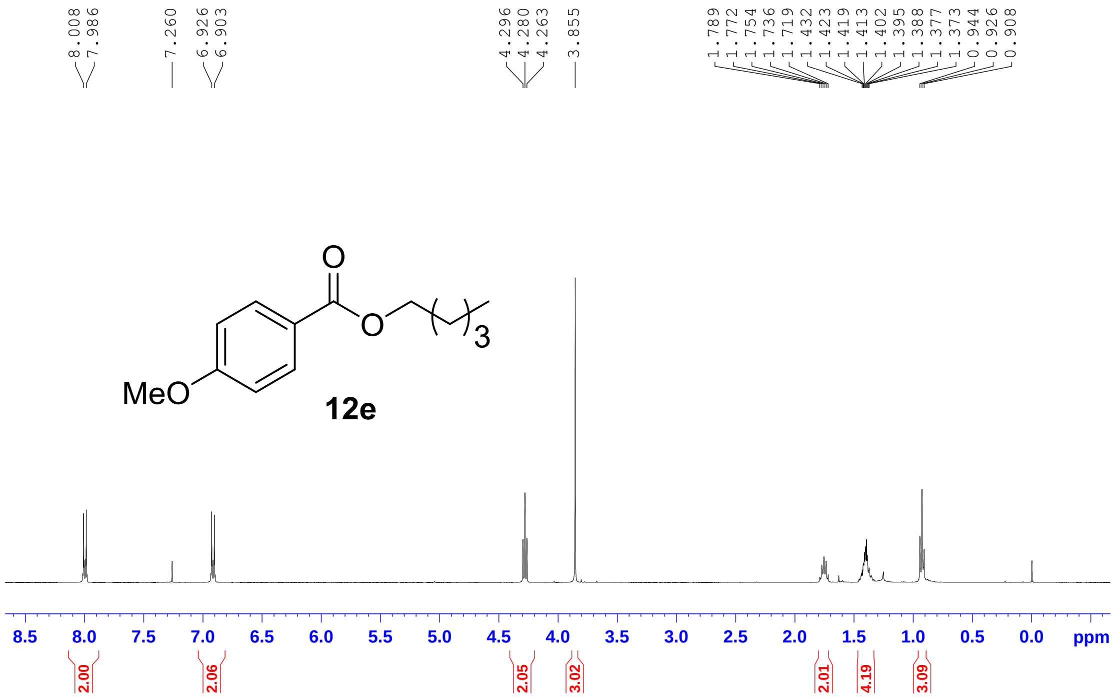
8.008
7.986
7.260
6.926
6.903
4.296
4.280
4.263
3.855
1.789
1.772
1.754
1.736
1.719
1.432
1.423
1.419
1.413
1.402
1.395
1.388
1.377
1.373
0.944
0.926
0.908
O
O
3
MeO
12e
8.5
8.0
7.5
7.0
6.5
6.0
5.5
5.0
4.5
4.0
3.5
3.0
2.5
2.0
1.5
1.0
0.5
0.0
ppm
2.00
2.06
2.05
3.02
2.01
4.19
3.09

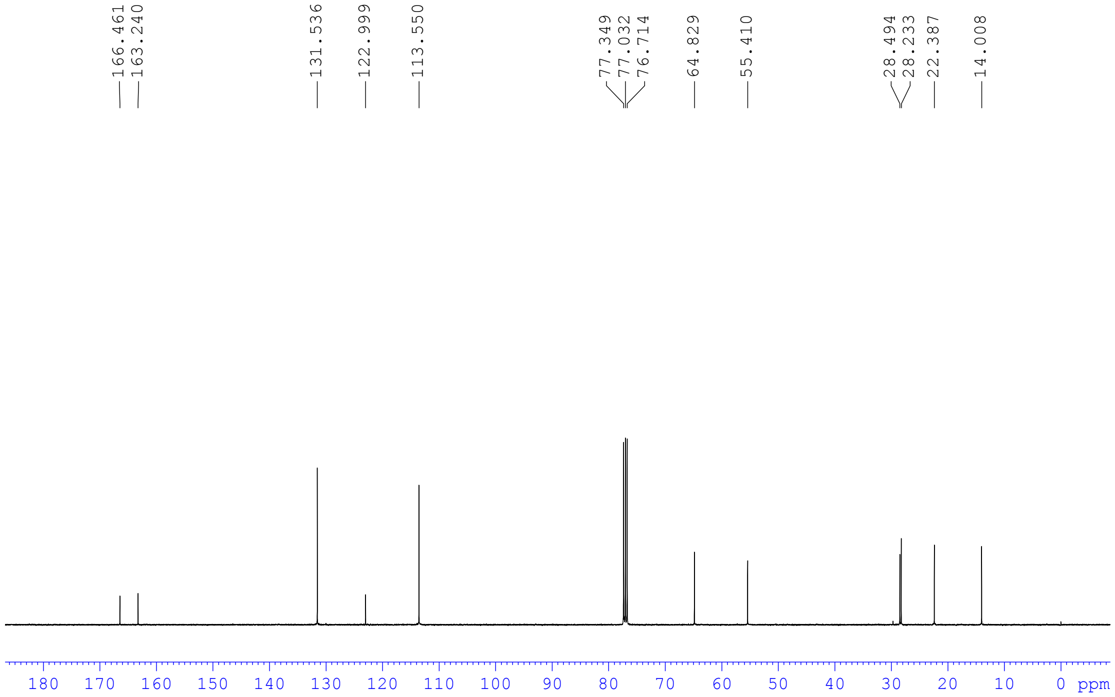
166.461
163.240
131.536
122.999
113.550
77.349
77.032
76.714
64.829
55.410
28.494
28.233
22.387
14.008
180
170
160
150
140
130
120
110
100
90
80
70
60
50
40
30
20
10
0
ppm

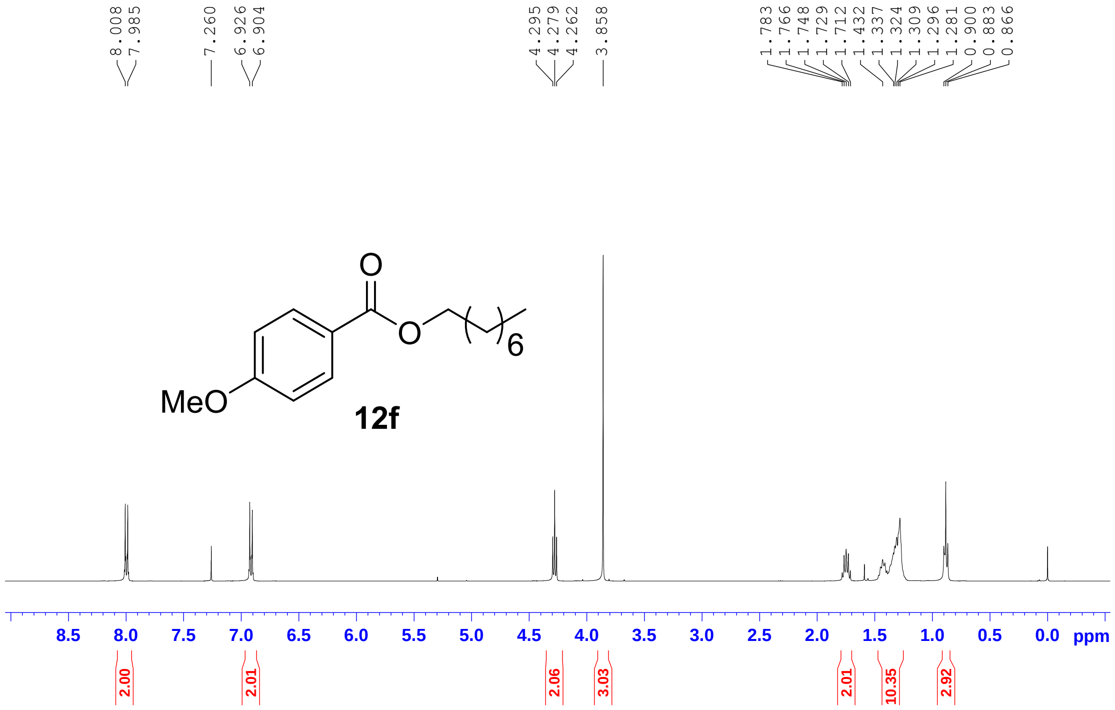
8.008
7.985
7.260
6.926
6.904
4.295
4.279
4.262
3.858
1.783
1.766
1.748
1.729
1.712
1.432
1.337
1.324
1.309
1.296
1.281
0.900
0.883
0.866
O
O
6
MeO
12f
8.5 8.0 7.5 7.0 6.5 6.0 5.5 5.0 4.5 4.0 3.5 3.0 2.5 2.0 1.5 1.0 0.5 0.0 ppm
2.00
2.01
2.06
3.03
2.01
10.35
2.92

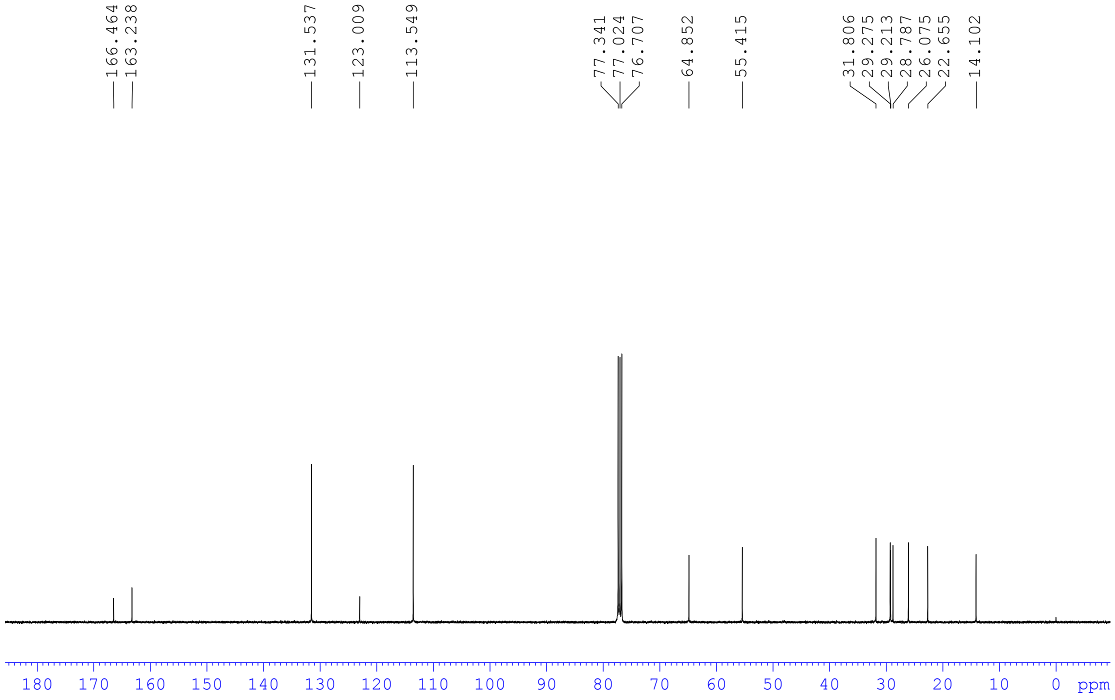
166.464
163.238
131.537
123.009
113.549
77.341
77.024
76.707
64.852
55.415
31.806
29.275
29.213
28.787
26.075
22.655
14.102
180 170 160 150 140 130 120 110 100 90 80 70 60 50 40 30 20 10 0 ppm

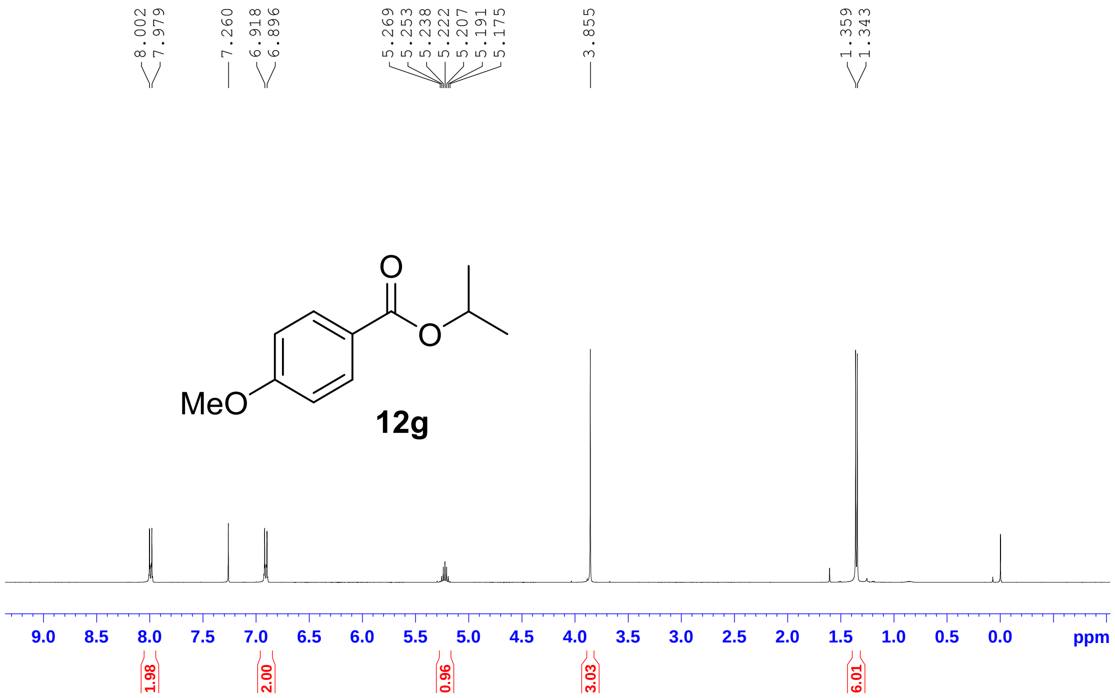
8.002
7.979
7.260
6.918
6.896
5.269
5.253
5.238
5.222
5.207
5.191
5.175
3.855
1.359
1.343
MeO
12g
1.98
2.00
0.96
3.03
6.01
ppm

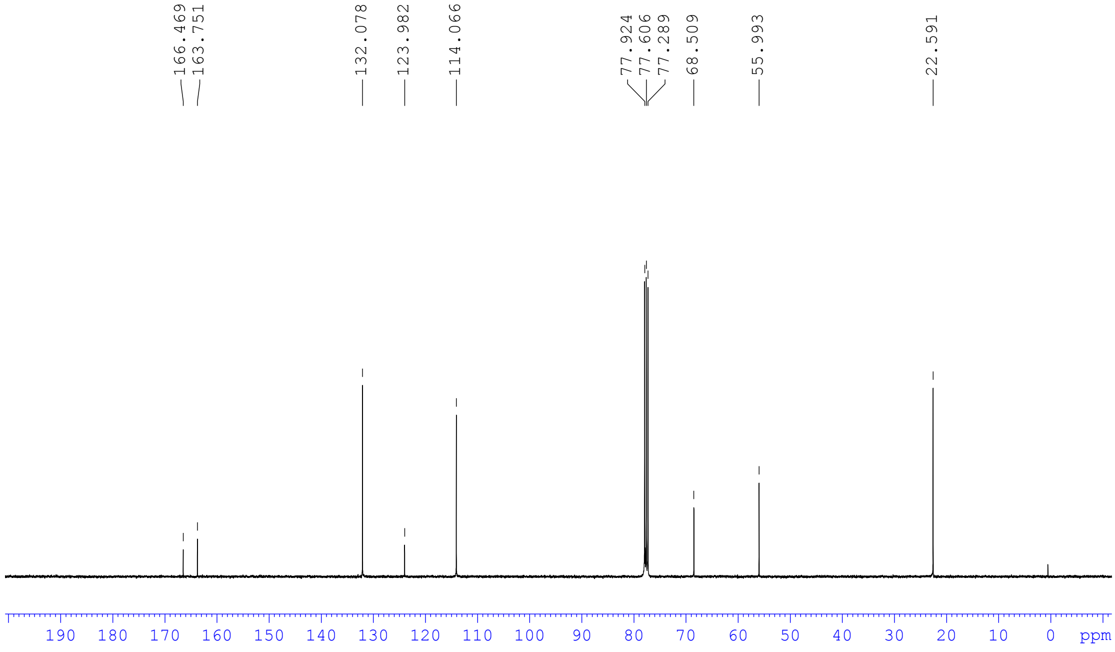
166.469
163.751
132.078
123.982
114.066
77.924
77.606
77.289
68.509
55.993
22.591
ppm

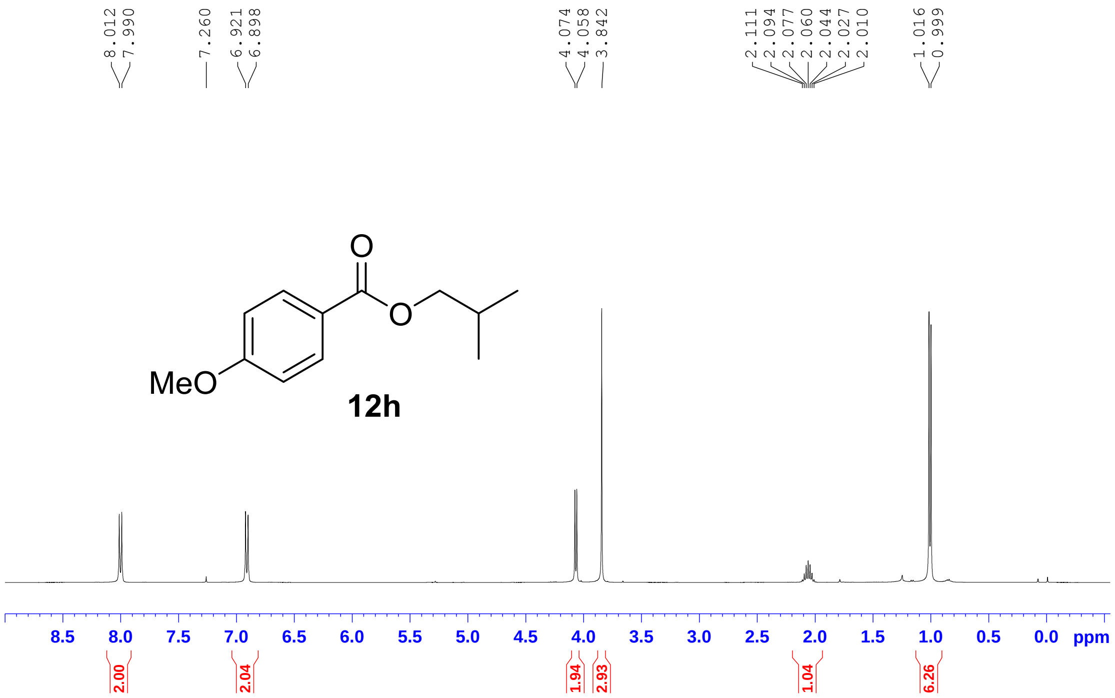
8.012
7.990
7.260
6.921
6.898
4.074
4.058
3.842
2.111
2.094
2.077
2.060
2.044
2.027
2.010
1.016
0.999
8.5 8.0 7.5 7.0 6.5 6.0 5.5 5.0 4.5 4.0 3.5 3.0 2.5 2.0 1.5 1.0 0.5 0.0 ppm
2.00
2.04
1.94
2.93
1.04
6.26

12h

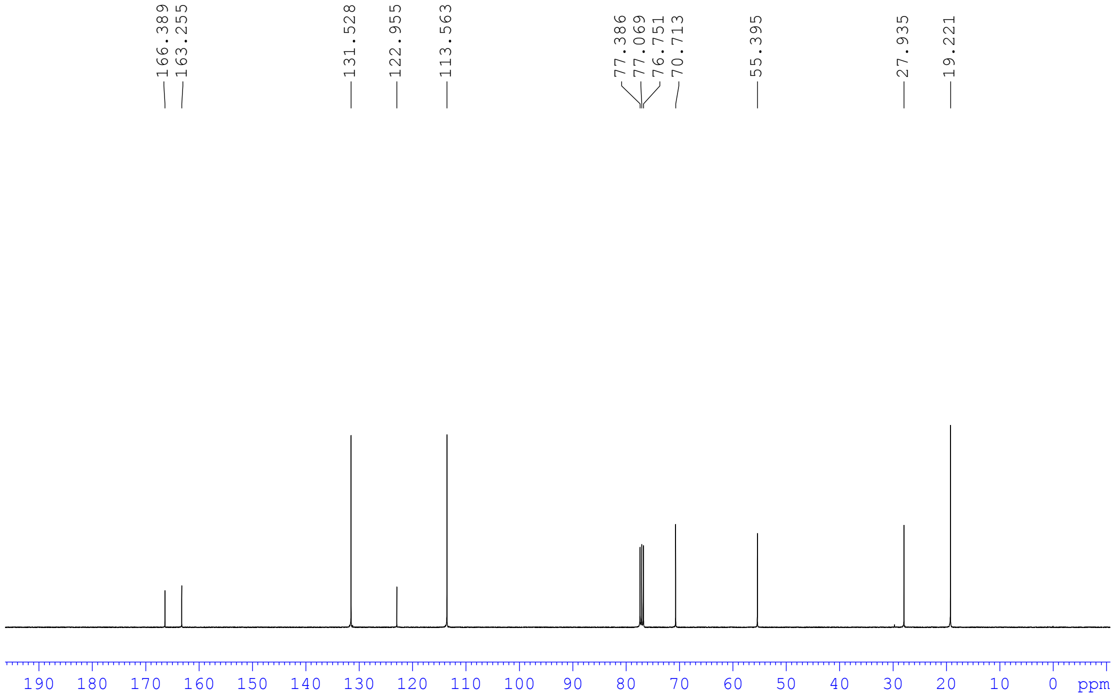
166.389
163.255
131.528
122.955
113.563
77.386
77.069
76.751
70.713
55.395
27.935
19.221
190 180 170 160 150 140 130 120 110 100 90 80 70 60 50 40 30 20 10 0 ppm

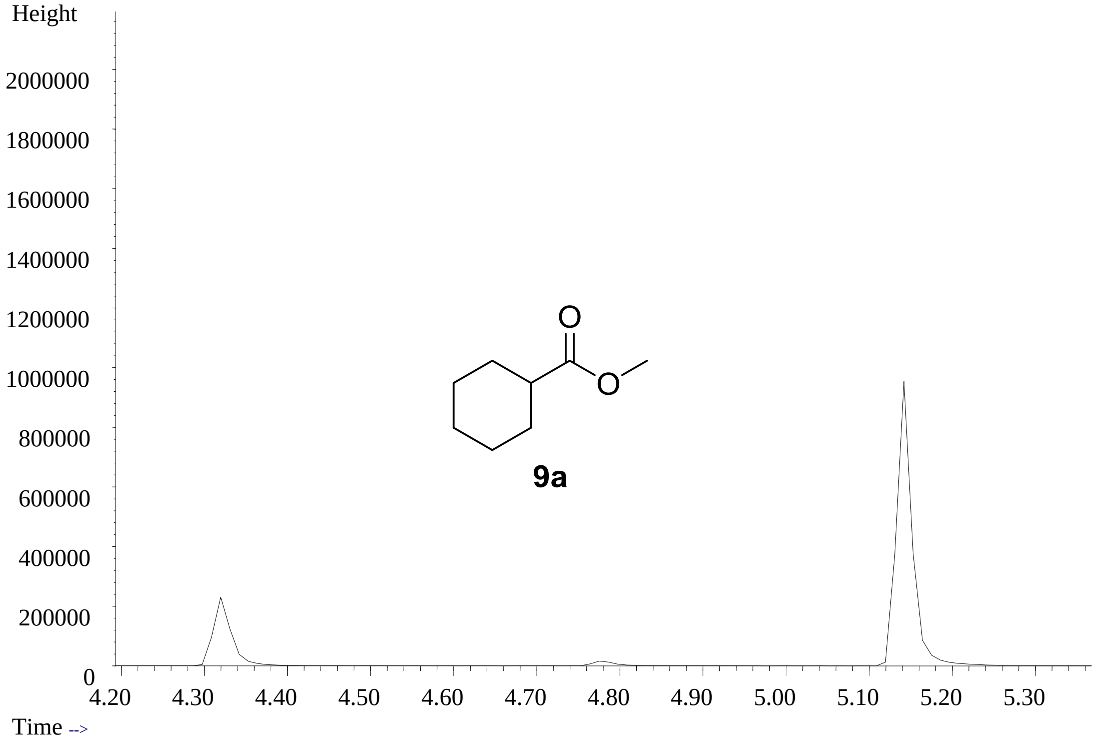

	Time	Area	Area%
1	4.320	348437	21.2
2	4.775	32484	2.0
3	5.142	1260619	76.8

1: cyclohexanecarbaldehyde, 2: cyclohexylmethanol, 3: methyl cyclohexanecarboxylate.

14. Crystallographic data for complex 11*

<table>
<tr>
<td>
C_{15} H_{11} Cl_3N_3Rh
M = 442.53
0.30 x 0.30 x 0.20 mm^3
Monoclinic, space group P21/n
a = 8.5110(10) Å
b = 13.8850(10) Å
c = 13.7120(10) Å
α = 90 °
β = 105.412(9) °.
γ = 90 °
V = 1562.1(2) Å^3
Z = 4
D_c = 1.882g/cm^3
T = 298(2)K
$2\theta_{max}$ = 51.2 °
16682 reflections collected,
2935 unique
Multiscan absorption correction
R_{int} = 0.0829
Final $GooF$ = 1.067
$R1$ = 0.0645
$wR2$ = 0.1591
199 parameters, 0 restraints
μ = 1.603 mm^{-1}
</td>
<td>
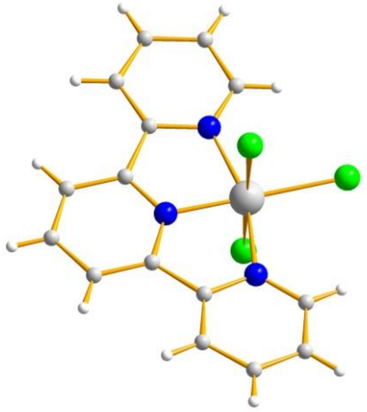
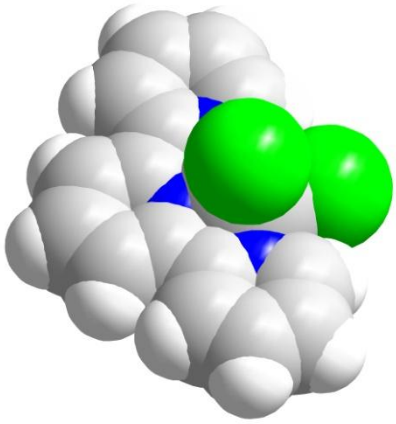
</td>
</tr>
</table>

*The structure of **11** has been determined, see: F. P. Pruchnik, P. Jakimowicz, Z. Ciunik, J. Zakrzewska-Czerwinska, A. Opolski, J.Wietrzyk, E. Wojdat, *Inorg. Chim. Acta*. **2002**, *334*, 59.

Table 1. Crystal data and structure refinement for **11**.

Identification code	11	
Empirical formula	$C_{15}H_{11}Cl_3N_3Rh$	
Formula weight	442.53	
Temperature	293(2) K	
Wavelength	0.71073 Å	
Crystal system	Monoclinic	
Space group	P2(1)/n	
Unit cell dimensions	a = 8.5110(10) Å	= 90°.

	b = 13.8850(10) Å	= 105.412(9)°.
	c = 13.7120(10) Å	= 90°.
Volume	1562.1(2) $Å^3$	
Z	4	
Density (calculated)	1.882 Mg/m^3	
Absorption coefficient	1.603 mm^{-1}	
F(000)	872	
Crystal size	0.30 x 0.30 x 0.20 mm^3	
Theta range for data collection	3.31 to 25.60 °.	
Index ranges	-10<=h<=10, -16<=k<=16, -16<=l<=16	
Reflections collected	16682	
Independent reflections	2935 [R(int) = 0.0829]	
Completeness to theta = 25.60 °	99.7 %	
Absorption correction	Semi-empirical from equivalents	
Max. and min. transmission	0.7398 and 0.6448	
Refinement method	Full-matrix least-squares on F^2	
Data / restraints / parameters	2935 / 0 / 199	
Goodness-of-fit on F^2	1.067	
Final R indices [I>2sigma(I)]	R1 = 0.0645, wR2 = 0.1591	
R indices (all data)	R1 = 0.1123, wR2 = 0.1852	
Largest diff. peak and hole	1.259 and -1.112 e.$Å^{-3}$	

Table 2. Atomic coordinates (x 10^4) and equivalent isotropic displacement parameters ($Å^2$x 10^3) for w. U(eq) is defined as one third of the trace of the orthogonalized U^{ij} tensor.

	x	y	z	U(eq)
Rh(1)	4743(1)	1336(1)	7694(1)	43(1)
Cl(1)	2270(3)	1113(2)	6460(2)	60(1)
Cl(3)	3292(4)	1618(2)	8907(2)	75(1)
Cl(2)	7198(4)	1539(2)	8917(2)	75(1)
C(1)	4454(11)	3515(7)	7541(8)	56(2)
C(15)	4479(12)	-672(7)	8515(7)	57(2)
C(8)	7431(14)	706(10)	5255(9)	77(3)
N(2)	5866(8)	1097(5)	6686(5)	41(2)
C(13)	5150(14)	-2080(8)	7728(9)	73(3)
C(6)	6178(11)	1802(7)	6133(7)	53(2)
C(11)	5619(11)	-519(7)	7152(7)	51(2)

C(2)	4644(13)	4407(7)	7120(10)	70(3)
C(12)	5701(12)	-1517(7)	7068(9)	64(3)
C(10)	6202(11)	148(6)	6508(7)	48(2)
N(1)	4926(9)	2708(5)	7188(5)	48(2)
C(9)	7010(14)	-55(8)	5789(8)	69(3)
C(4)	5862(13)	3617(8)	5968(9)	67(3)
N(3)	4972(8)	-124(5)	7853(5)	48(2)
C(7)	7011(12)	1624(9)	5417(7)	64(3)
C(5)	5643(12)	2750(7)	6407(7)	55(2)
C(14)	4576(14)	-1669(8)	8449(10)	74(3)
C(3)	5361(14)	4445(8)	6336(10)	76(3)

Table 3. Bond lengths [Å] and angles [°] for **11**.

Rh(1)-N(2)	1.907(7)
Rh(1)-N(3)	2.043(7)
Rh(1)-N(1)	2.048(8)
Rh(1)-Cl(2)	2.323(3)
Rh(1)-Cl(1)	2.345(3)
Rh(1)-Cl(3)	2.353(3)
C(1)-N(1)	1.326(12)
C(1)-C(2)	1.393(14)
C(1)-H(1)	0.9300
C(15)-N(3)	1.335(11)
C(15)-C(14)	1.392(15)
C(15)-H(15)	0.9300
C(8)-C(7)	1.358(16)
C(8)-C(9)	1.385(16)
C(8)-H(8)	0.9300
N(2)-C(6)	1.308(12)
N(2)-C(10)	1.384(10)
C(13)-C(14)	1.341(16)
C(13)-C(12)	1.370(16)
C(13)-H(13)	0.9300
C(6)-C(7)	1.378(13)
C(6)-C(5)	1.474(14)
C(11)-N(3)	1.345(11)
C(11)-C(12)	1.394(13)

C(11)-C(10)	1.455(13)
C(2)-C(3)	1.371(16)
C(2)-H(2)	0.9300
C(12)-H(12)	0.9300
C(10)-C(9)	1.373(13)
N(1)-C(5)	1.367(11)
C(9)-H(9)	0.9300
C(4)-C(3)	1.369(16)
C(4)-C(5)	1.381(13)
C(4)-H(4)	0.9300
C(7)-H(7)	0.9300
C(14)-H(14)	0.9300
C(3)-H(3)	0.9300

N(2)-Rh(1)-N(3)	81.5(3)
N(2)-Rh(1)-N(1)	79.5(3)
N(3)-Rh(1)-N(1)	161.0(3)
N(2)-Rh(1)-Cl(2)	91.0(2)
N(3)-Rh(1)-Cl(2)	90.1(2)
N(1)-Rh(1)-Cl(2)	89.8(2)
N(2)-Rh(1)-Cl(1)	88.9(2)
N(3)-Rh(1)-Cl(1)	89.3(2)
N(1)-Rh(1)-Cl(1)	90.7(2)
Cl(2)-Rh(1)-Cl(1)	179.39(10)
N(2)-Rh(1)-Cl(3)	178.5(2)
N(3)-Rh(1)-Cl(3)	98.2(2)
N(1)-Rh(1)-Cl(3)	100.7(2)
Cl(2)-Rh(1)-Cl(3)	90.56(11)
Cl(1)-Rh(1)-Cl(3)	89.60(10)
N(1)-C(1)-C(2)	121.4(10)
N(1)-C(1)-H(1)	119.3
C(2)-C(1)-H(1)	119.3
N(3)-C(15)-C(14)	119.2(10)
N(3)-C(15)-H(15)	120.4
C(14)-C(15)-H(15)	120.4
C(7)-C(8)-C(9)	120.9(10)
C(7)-C(8)-H(8)	119.5
C(9)-C(8)-H(8)	119.5

C(6)-N(2)-C(10)	121.8(8)
C(6)-N(2)-Rh(1)	120.6(6)
C(10)-N(2)-Rh(1)	117.4(6)
C(14)-C(13)-C(12)	120.0(11)
C(14)-C(13)-H(13)	120.0
C(12)-C(13)-H(13)	120.0
N(2)-C(6)-C(7)	120.1(9)
N(2)-C(6)-C(5)	113.3(8)
C(7)-C(6)-C(5)	126.4(10)
N(3)-C(11)-C(12)	120.3(10)
N(3)-C(11)-C(10)	116.4(8)
C(12)-C(11)-C(10)	123.3(9)
C(3)-C(2)-C(1)	118.9(10)
C(3)-C(2)-H(2)	120.5
C(1)-C(2)-H(2)	120.5
C(13)-C(12)-C(11)	118.6(11)
C(13)-C(12)-H(12)	120.7
C(11)-C(12)-H(12)	120.7
C(9)-C(10)-N(2)	119.3(9)
C(9)-C(10)-C(11)	128.5(9)
N(2)-C(10)-C(11)	112.3(8)
C(1)-N(1)-C(5)	119.4(8)
C(1)-N(1)-Rh(1)	127.3(7)
C(5)-N(1)-Rh(1)	113.3(6)
C(10)-C(9)-C(8)	118.1(10)
C(10)-C(9)-H(9)	120.9
C(8)-C(9)-H(9)	120.9
C(3)-C(4)-C(5)	118.7(11)
C(3)-C(4)-H(4)	120.7
C(5)-C(4)-H(4)	120.7
C(15)-N(3)-C(11)	121.1(8)
C(15)-N(3)-Rh(1)	126.9(7)
C(11)-N(3)-Rh(1)	112.0(6)
C(8)-C(7)-C(6)	119.5(10)
C(8)-C(7)-H(7)	120.3
C(6)-C(7)-H(7)	120.3
N(1)-C(5)-C(4)	121.3(10)
N(1)-C(5)-C(6)	113.2(8)

C(4)-C(5)-C(6)	125.5(10)
C(13)-C(14)-C(15)	120.7(11)
C(13)-C(14)-H(14)	119.6
C(15)-C(14)-H(14)	119.6
C(4)-C(3)-C(2)	120.3(10)
C(4)-C(3)-H(3)	119.9
C(2)-C(3)-H(3)	119.9
